# Shedding light on dark taxa: exploring a cryptic diversity of parasitoid wasps affected by artificial light at night

**DOI:** 10.1038/s41598-025-88111-3

**Published:** 2025-02-20

**Authors:** Manuel Dietenberger, Andreas Jechow, Manuela Sann, Franz Hölker

**Affiliations:** 1 Leibniz Institute of Freshwater Ecology and Inland Fisheries (IGB), Müggelseedamm 310, 12587 Berlin, Germany; 2 Institute of Biology, Freie Universität Berlin, Königin-Luise-Straße 1-3, 14195 Berlin, Germany; 3 Chair of Nature Conservation and Landscape Ecology, Albert-Ludwigs-Universität Freiburg, Stefan-Meier-Str.76, 79104 Freiburg, Germany; 4 Department of Engineering, Brandenburg University of Applied Sciences, Magdeburger Str. 50, 14770 Brandenburg an der Havel, Germany; 5 Natural History Museum Bern, Bernastraße 15, Bern, 3005 Switzerland

**Keywords:** Insect decline, Light pollution, Parasitoids, Street lights, Artificial light at night, Shielding, Biological control, Ecology, Community ecology, Conservation biology, Sequencing

## Abstract

Artificial light at night (ALAN) contributes to the globally observed insect decline. ALAN attracts nocturnal insects from their native ecosystems and disturbs their functions in the food web. Road lights in this context are ubiquitous and relevant ALAN sources that are often not considered in conservation approaches. In a previous study we showed that shielded LED road lights are suited to be part of conservation measures by effectively reducing the attraction of nocturnal insects. Here we show that this positive effect holds true for parasitoid wasps in an experimental BACI design (Before-After-Control-Impact). Combining morphological with molecular and phylogenetic analyses, we identified 106 individuals (62 morphotypes) of a minimum of 45 genera out of 13 Hymenoptera families. We were able to identify 21 species, 11 of which are newly reported in Southern Germany (Baden-Württemberg). Further combining knowledge on life history and host appearance from our data and the literature, we discuss potential impacts of ALAN ranging from an influence on nocturnal pollination via parasitoid pressure on moth species and biological control of invasive pest species to tritrophic interactions between primary and secondary parasitoids. We conclusively think that shielded LED road lights will reduce the ecological impact of ALAN on parasitoid wasps in a large and undescribed number of taxa with different host associations, likely affecting associated ecosystem functions such as biological control.

**Supplementary Information:**

The online version contains supplementary material available at 10.1038/s41598-025-88111-3.

## Introduction

### Artificial light and parasitoid wasps

Natural light-dark cycles, defined as daily, annual and lunar cycles that are stable across geological and thus evolutionary time scales, are crucial for synchronisation of many physiological and behavioural processes^1,2^. In this context, it is hardly surprising that artificial light at night (ALAN) has been identified as one of the causes of the worldwide insect decline^3,4^. This decline will likely have severe effects on energy/material flows and consequently on important ecosystem functions and services associated with insects (e.g. biological control, plant pollination, primary production, decomposition, nutrient cycling^5–7^). Among Hymenoptera, parasitoid wasps represent a hyper-diverse functional group with a wide range of undescribed species, so called “dark-taxa”, and host organisms, many of them playing important beneficial roles in integrated pest management^8–10^. While the sparse research in the field of ALAN and biological control focuses on model systems and population dynamics (e.g. plant-aphid-parasitoid) which are important in many temperate terrestrial ecosystems^11^ and play a major role in the control of economically relevant pest species^12^, few studies investigate naturally occurring parasitoid communities. Further, many studies investigating direct behavioural responses of parasitoids to light are conducted under confined artificial laboratory conditions^13^ and lack reference to field conditions with numerous relevant factors for parasitoids that can influence their behaviour (e.g. light competition, host abundance, olfactory cues, natural variability of abiotic parameters).

### The attraction effect

While in pest control (e.g. light trapping in greenhouses) attraction is often a desired effect^8^, the attraction of insects to ALAN constitutes a major problem in natural environments. Many artificial light sources attract insects, withdrawing them from their neighbouring habitats and ecological functions in the food web^14,15^. By affecting host and parasitoid species and life stages differently, this will likely have an influence on top-down control in insect food webs with unknown consequences for associated ecosystem functions^16^. Mitigation strategies to reduce insect attraction are therefore urgently needed. This may be especially relevant at transition zones between protected areas and human settlements, where public outdoor lighting represents a constantly present nocturnal disturbance and intervention, often not considered in conservation approaches at state-level^17^. While there are different parameters (e.g. timing and duration of illumination, the spectral composition of the light source, the light intensity, the geometry of the light emission), that could be integrated into conservation measures, uncertainty remains about the best strategies for ensuring road safety and good visibility from a human perspective while minimising the attraction radius for nocturnal insects^18^.

### Experimental design

**Fig. 1:**
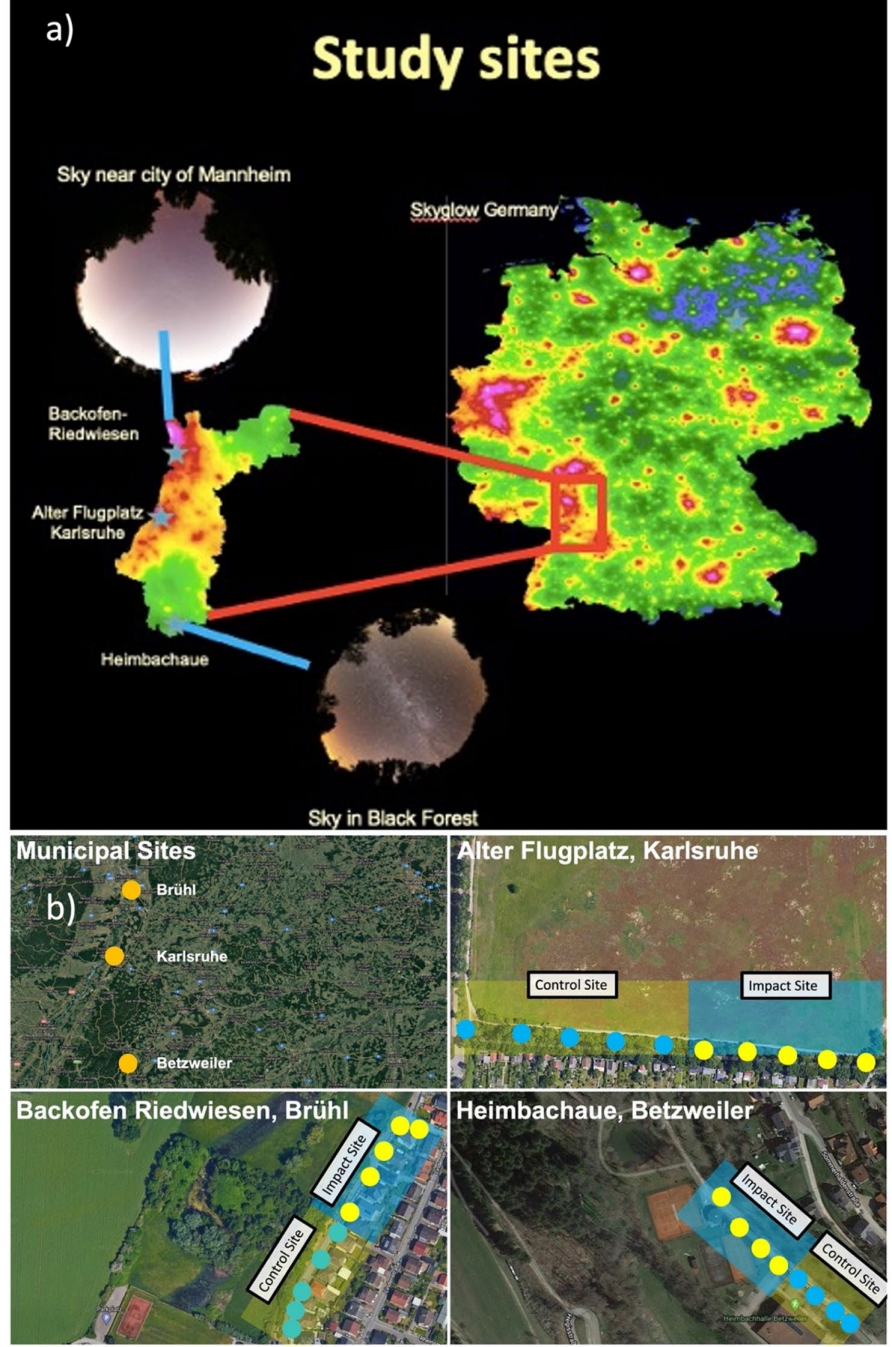
Municipal study sites. (**a**) Municipal study sites in Southern Germany (federal state of Baden-Württemberg) selected from different levels of astronomical light pollution (skyglow), different grades of urbanisation and different habitats. Map shows data from a skyglow model from Falchi et al.^21^ and was produced with QGIS (Version 3.1.0, www.qgis.org). (**b**) Municipal study sites in Baden-Württemberg and road lights equipped with insect traps. Control luminaires (blue circles) remained untransformed (= 3 Control sites). Impact luminaires (yellow circles) were converted in Winter 2021–2022 (= 3 Impact sites). Study site 1: Alter Flugplatz Karlsruhe, grassland, urban, *n* = 5 luminaires per control/impact site (conversion from conventional LED 4000 K to tailored LED 4000 K). Study site 2: Backofen Riedwiesen Brühl, Rhine plain, suburban, *n* = 5 luminaires per control/impact site (conversion from conventional HPS cylinder 2000 K to tailored LED 2700). Study site 3: Heimbachaue Betzweiler, river plain/forest, rural, *n* = 4 luminaires per control/impact site (conversion from conventional bell-shaped HPS 2000 K to tailored LED 2700/2000K). Maps data: Google, © 2022 CNES / Airbus, Maxar technologies.

In our experimental BACI-Setup (Before-After-control-Impact), we aimed to test the effect of spatially confining light emission when converting different less shielded conventional luminaires to tailored LED luminaires. For this purpose, we monitored the attraction effect of individual luminaires on nocturnal insects with flight interception traps over a period of two years (2021–2022). This was conducted at three municipal streets (study sites) close to nature reserves in Southern Germany, representing a gradient in skyglow (ALAN scattered back in the atmosphere) and urbanisation (urban, suburban, rural). At each study site, paired control and impact sites with similar numbers of replicate subplots (luminaires) were selected (Fig. 1). With this experimental design, control sites were not experimental controls to explain environmental variability, but were chosen to be highly correlated with the impact sites, reflecting covariates to reduce unexplained temporal variation^19^. At the impact sites, we converted different types of road lights with less shielded emission (LED 4000 K, high pressure sodium (HPS) 2000 K) to tailored and shielded LED luminaires (LED 4000 K, 2700 K, 2000 K) after one year. The new lights constitute a novel approach to reduce insect attraction via a tailored light emission on the target area and an external shield around the LED panels, reducing unintentional spill light in adjacent areas and the visibility of luminaire heads from a distance (described in detail in Dietenberger et al. 2024 ^20^). The tailored and shielded lights represent a highly optimised solution for local site conditions (street width, luminaire height, spacing), being in line with EU lighting norms (EN 13201).

### Hypotheses

These transitions from various conventional less shielded luminaires to tailored and shielded LEDs allowed us to test two hypotheses. Compared to different conventional luminaires without optimised light distribution, **H1)** tailored and shielded road lights reduce the number of attracted parasitoid wasps (abundance), and **H2)** tailored and shielded road lights reduce the number of attracted species among parasitoid wasps (species richness). This was possible by combining morphological, molecular and phylogenetic analyses of a “dark taxa” - parasitoid wasps. To our knowledge, no other study evaluated the attraction of mostly very small-sized (in this study ranging from 0.5 mm to 17 mm in body length) parasitoid wasps to public outdoor lighting under field conditions on this level of taxonomic resolution. In a last step we investigated reported host associations of the identified parasitoid species from the literature. We combined this with species data on Lepidoptera abundances in our experimental set up (as an important host group). In this context, we preliminary assumed that **H3)** road lights will affect whole host-parasitoid-complexes by attracting adult life stages of both, parasitoids and related host species.

## Results

### Light treatments

In a total of 55 sampling nights over two years (10 luminaires in 17 nights at site 1, 10 luminaires in 15 nights at site 2, 8 luminaires in 23 nights at site 3), a total of 411 hymenopteran insects out of 18 families/subfamilies were caught at three sites (Supporting Information Table S1, Figure S1). Most Hymenoptera were winged individuals of the family Formicidae (62%), 1% Others, 11% Vespinae (*Vespula* and *Vespa*) and 26% belonged to families that exhibit a parasitoid lifestyle. The latter were subject to DNA-Analysis (see section Barcoding). In 2021, we collected 26 individuals (22 parasitoid species), in 2022 we collected 80 individuals (46 parasitoid species). In addition, 88 species of *Lepidoptera* (potential hosts) were identified morphologically. This data is available via the DOI included at the end of this article (File 1).

**Fig. 2:**
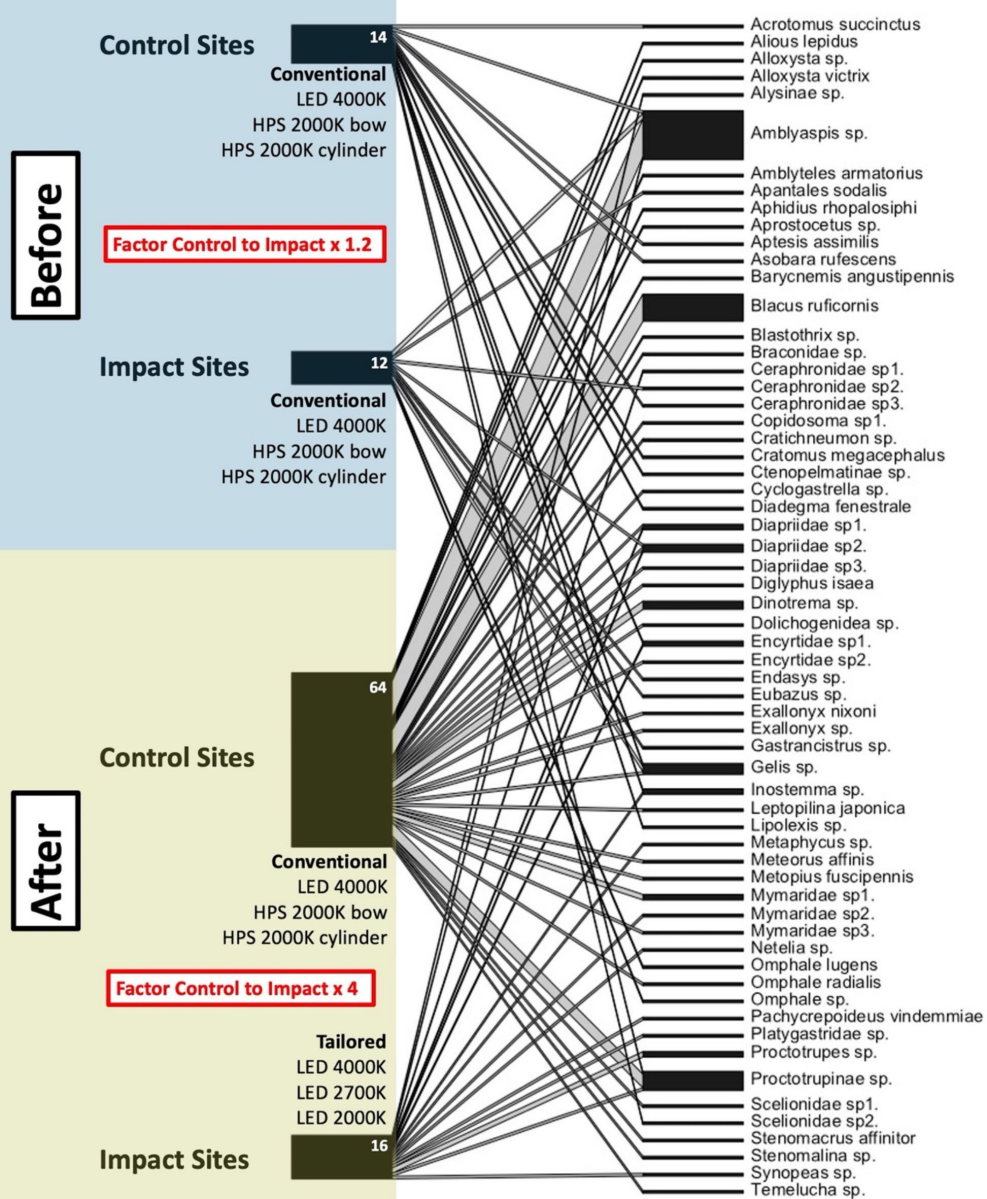
Bipartite Species-Site-Network. Left bars represent total parasitoid abundance at Control and Impact Sites before and after the conversion to tailored and shielded LEDs at the Impact Sites. Right bars show parasitoid taxa. Numbering of sp.-species refers to different morphotypes. Width of bars corresponds to abundance (white numbers). Before (2021): *n* = 26 samplings (Site 1: Alter Flugplatz, five luminaires per control and impact site, eight samplings; Site 2: Backofen Riedwiesen, five luminaires per control and impact site, eight samplings; Site 3: Heimbachaue, four luminaires per control and impact site, ten samplings). After (2022): *n* = 29 samplings (Site 1: Alter Flugplatz, five luminaires per control and impact site, nine samplings; Site 2: Backofen Riedwiesen, five luminaires per control and impact site; seven samplings; Site 3: Heimbachaue, four luminaires per control and impact site, 13 samplings).

**Fig. 3:**
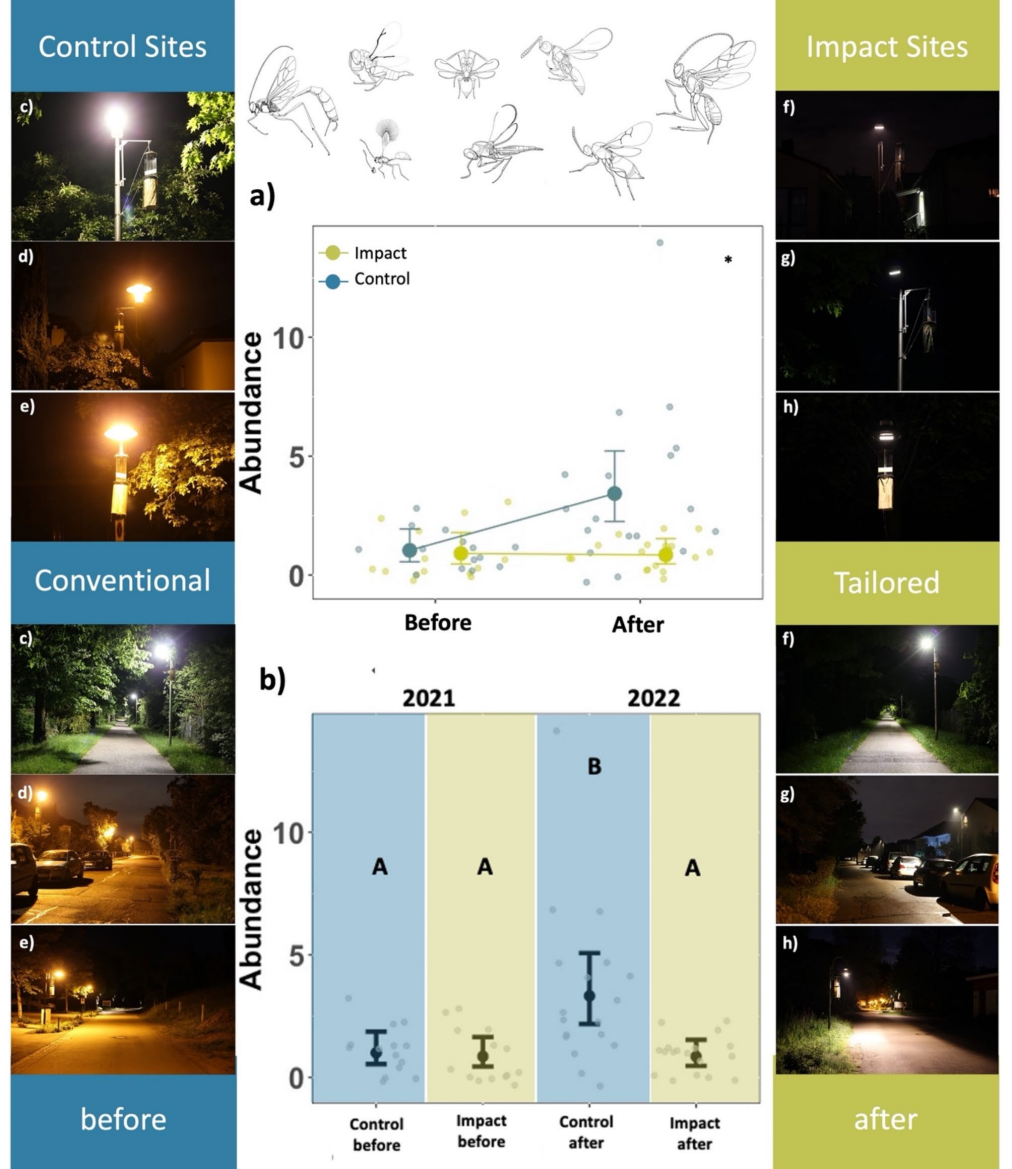
Results of generalised linear models on parasitoid abundance. (**a**) The BACI-effect on the number of parasitoids per road light (Abundance, points) between control and impact sites (colours) before and after the intervention. Error bars show model predictions (points) and 95% Confidence intervals. (**b**) Pairwise differences in the total number of parasitoids per road light (Abundance) between treatment groups (control before, control after, impact before, impact after). In each group, there were 14 luminaires. Letters refer to a Tukey post-hoc test. Different letters indicate significant differences. 2021 (Before): *n* = 26 samplings, (Site 1: eight samplings, Site 2: eight samplings, Site 3: ten samplings; 2022 (After): *n* = 29 samplings (Site 1: nine samplings, Site 2: seven samplings, Site 3: 13 samplings). Treatment groups: Control sites (before): (conventional LED 4000 K (**c**), HPS 2000 K (**d**, **e**)); Control sites (after): (conventional LED 4000 K, HPS 2000 K), Impact sites (before): (conventional LED 4000 K, HPS 2000 K); Impact sites after (tailored and shielded LED 4000 K (**f**)/HPS 2700 K (**g**, **h**) in 2022). Error bars show model predictions (points) and 95% Confidence intervals. Parasitoid drawings derived from Goulet et al. (1993).

Since parasitoids were mostly singletons and the overall abundance very low (106), we compared abundances per luminaire between control and impact sites (before and after) visually in a network plot (Fig. 2) and for individual sites (Supporting Information Figure S2). When modelling parasitoid abundance in a generalised linear model, we observed that the abundance (*p* = 0.01*) was affected by the interaction of period (before-after) and treatment (control-impact) (the BACI effect) (Fig. 3a, Supporting Information Table S2). The estimated BACI effect (mean **C**ontrol **A**fter - mean **C**ontrol **B**efore ‐ (mean **I**mpact **A**fter ‐ mean **I**mpact **B**efore) of the model was 2.45 ± 0.77.

Parasitoid abundance was similarly affected by the treatment group (combination of period and treatment = 4 treatment groups). Models showed that only the conventional luminaires at the control sites 2022 (**A**fter) attracted significantly more parasitoids wasps (*p* < 0.001 ***) (Fig. 3b, Supporting Information Table S3) compared to conventional luminaires at the control sites 2021 (**B**efore). In contrast, a similarly observed increase was less pronounced and not significant at the tailored and shielded luminaires at the impact sites 2022 (after) (*p* = 0.682). A pairwise comparison further revealed that only the conventional control luminaires (**A**fter) attracted significantly more parasitoids than all other treatment groups and that there were no further differences between the individual treatment groups. (Supporting Information Table S4).

**Fig. 4:**
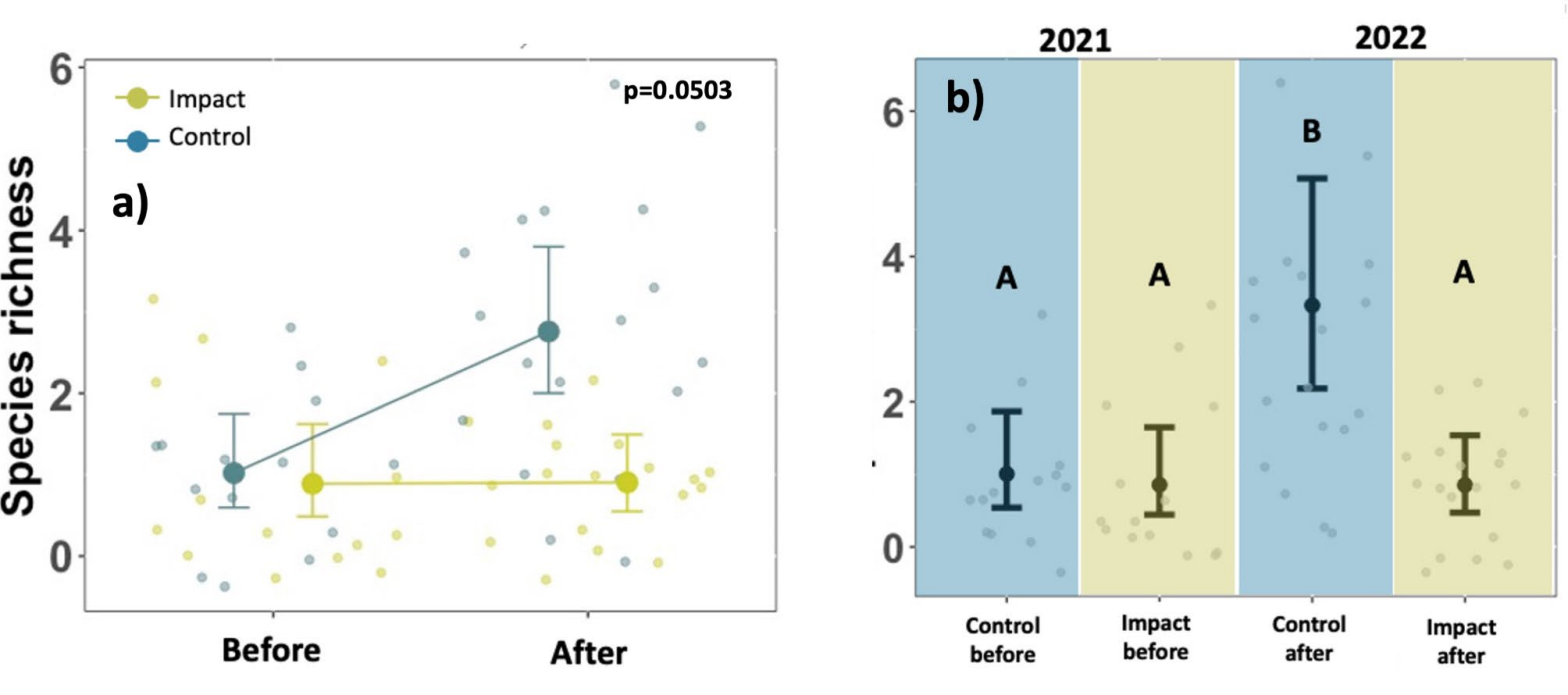
Results of generalised linear models on parasitoid species richness. (**a**) The BACI-effect on the number of parasitoid species per road lights (species richness, points) between control and impact sites (colours) before and after the intervention. Error bars show model predictions (points) and 95% Confidence intervals. (**b**) Pairwise differences in the total number of parasitoid species per road light (species richness) between individual treatment groups (control before, control after, impact before, impact after). Letters refer to a Tukey post-hoc test. Different letters indicate significant differences. In each group there were 14 luminaires. 2021: *n* = 26 samplings (eight samplings Site 1, eight samplings Site 2, ten samplings Site 3; 2022: *n* = 29 samplings (nine samplings Site 1, seven samplings Site 2, 13 samplings Site 3). Treatment groups: Control sites (before): (conventional LED 4000 K, HPS 2000 K); Control sites (after): (conventional LED 4000 K, HPS 2000 K), Impact sites (before): (conventional LED 4000 K, HPS 2000 K); Impact sites after (tailored and shielded LED 4000 K/2700K/2000K in 2022).

The species richness of parasitoids was only marginally affected by the BACI interaction term (*p* = 0.0503) (Fig. 4a, Supporting Information Table S5). The estimated BACI effect of the model was 1.72 ± 0.63. The treatment groups also had a significant effect on the number of parasitoid species (species richness). The statistical models showed that only conventional luminaires at the control sites 2022 (after) attracted significantly more parasitoid wasp species (*p* = 0.0153 **) (Fig. 4a, Supporting Information Table S6) in 2022 compared to conventional luminaires in the year before. This increase was less pronounced and not significant at the new tailored and shielded luminaires at the impact sites in 2022 (after) (*p* = 0.736). The pairwise comparison further revealed that only the conventional control luminaires (**A**fter) attracted significantly more parasitoid species than all other treatment groups and that there were no further differences between the individual treatment groups. (Supporting Information Table S4). (Fig. 4b, Supporting Information Table S7).

### DNA barcoding

A total of 106 individuals, belonging to 13 families within the Hymenoptera reported to show a parasitoid lifestyle, were investigated. When comparing the morphological and COI-based phylogeny, our results show that the examined parasitoid individuals represent 62 different morphotypes of at least 45 genera out of 13 Hymenoptera families (Supporting Information Figures S3-S155, Table S8). We successfully barcoded 95 out of 106 specimens and determined the remaining individuals (11) morphologically to family and/or genus level. From 95 sequences, 24 CO1 sequences were determined to species level (Supporting Information Table S9), representing 21 different species. The remaining individuals were identified on genus (53 individuals) or family (18 individuals) level. The COI-based phylogenetic tree confirmed our identifications (Supporting information Figure S156). Due to low sequence length, we discarded 5 of 95 sequences here. Additionally, we identified 3 species with no COI reference sequence in Germany (Global Biodiversity Information Facility (GBIF)) (Supporting Information Table S10). Further, 14 of the species identified here have not been reported on a molecular basis in the federal state of Baden-Württemberg (GBIF). Of these, 11 species (*Aliolus Lepidus (Haliday 1835)*,* Apanteles sodalis (Haliday 1834)*,* Aphidius rhopalosiphi (De Stefani Perez 1902)*,* Asobara rufescens (Forster 1862)*,* Blacus ruficornis (Nees 1811)*,* Diglyphus isaea (Walker 1838)*,* Exallonyx nixoni (Townes 1981)*,* Meteorus affinis (Wesmael 1835)*,* Omphale lugens (Nees 1834)*,* Omphale radialis (Thomson 1878*), *Stenomacrus affinitor (Aubert 1981)* were not reported for Baden-Württemberg elsewhere in the available literature (Supporting Information Table S10). Host associations reported from the literature can be seen in Supporting Information Table S10.

## Discussion

### Light treatments

The analyses of this Before-After-Impact-Control experiment showed that the applied intervention (the transformation from different conventional lighting techniques to spatially optimised LED road lighting with shielding) resulted in a reduced attraction of parasitoid wasps in terms of abundance at three municipal streets close to nature reserves in Southern Germany (supporting H1). In addition, the conventional luminaires at the control and impact sites were not significantly different in terms of parasitoid attraction before the intervention, representing an important prerequesite for a proper evaluation of this simulated conversion scenario.

These results over two seasons were obtained from a broad spectrum of environmental conditions, grades of urbanisation and different conventional lighting contexts and involved different light intensities and spectra. We therefore think that the observed reduction in the attraction of parasitoid wasps, which have the potential to be used as sensitive indicator species of environmental change^22^, strongly supports the results of our previous study^20^. We showed that the new tailored and shielded LED lights have a reducing effect on the overall attraction of nocturnal flying arthropods after the intervention at the individual study sites by greatly reducing the area in which insects can get confused. The reduced attraction of parasitoid wasps in this study extends these insights and additionally points out the value of reducing spill light and visibility of luminaire heads from a distance. This will likely reduce an influence on host-parasitoid interactions and subsequently on population dynamics of parasitoids and associated ecosystem functions, such as biological control, as Sanders et al. have been shown in semi-field experiments^23^. Their study showed that community response to ALAN in an aphid-parasitoid complex is intensity-dependent, suggesting that additional context-dependent dimming of tailored and shielded road lights could be a further improvement.

In addition, the new tailored and shielded luminaires at the impact sites in 2022 were not significantly different from overall low parasitoid abundances attracted to conventional luminaires at control and impact sites in the year before (2021), indicating that the tailored lighting design compensated for a seasonal increase in parasitoid abundance from 2021 to 2022. The factor from control to impact sites changed from x1.2 before to x4 after the intervention.

Although parasitoid species were mostly singletons and the species richness was only marginally impacted by the “BACI-effect”, a pairwise comparison of the number of species (morphotypes) between control and impact sites before and after the conversion at the impact sites gave additional insights, indicating that tailoring and shielding of luminaires also reduced the number of attracted parasitoid species compared to different conventional luminaires (supporting H2). The attraction of different species to ALAN is related to different photosensitivities, but the observed reduction in the number of species in this study is most likely related to an overall reduced parasitoid abundance at tailored and shielded LEDs^24^. Site 3 (rural) in the Black Forest showed the highest number of parasitoids (30 species). Of 53 parasitoids in 2022, only seven individuals (13%), representing seven different species (23%) were attracted to the new tailored luminaires at the impact site in 2022. In summary, the tailored lighting approach investigated in this study reduced distracting and misleading visual cues and the number of affected individuals and species and is therefore expected to be beneficial for ecosystem services associated with parasitoid wasps in different host associations, which may be especially relevant close to protected areas, where light pollution is often not integrated into conservation approaches. We therefore propose that a tailored light distribution of neighbouring road lights should be a part of future mitigation strategies.

### Barcoding

Integrative taxonomy is becoming ever more significant in biodiversity research as scientists are increasingly tackling taxonomically challenging groups such as chironomids or parasitoid wasps^25^. By using such an approach, we investigated the attraction of parasitoid wasps to road lights, which has provided valuable insights into a largely undescribed diversity of species affected by ALAN in Baden-Württemberg. The comparison of a COI-based phylogeny and morphological data allowed us to identify and discuss ALAN-induced impacts of 21 different insect species, of which 11 were not previously reported in Baden-Württemberg. In the following, the potential impacts of ALAN on eight parasitoid species whose life history, host association and distribution data are available, are briefly discussed:

### Lepidoptera parasitoids

***Apanteles sodalis*(*Braconidae)*** is a koinobiont endoparasite of lepidopteran larvae^26^, emerging at Site 2 in the upper rhine plain in September 2021 at conventional HPS 2000 K luminaires. A potential lepidopteran host species is *Archips rosana* (*Tortricidae*), a relevant pest of different soft fruits^27^. Potential *Tortricidae* hosts at conventional HPS at Site 2 were *Pandemis heparana* and *Eucosma metzneriana* emerging in mid-June 2022.

***Diadegma fenestrale (Ichneumonidae)*** was attracted to conventional LED 4000 K in late September 2021 at Site 1 in Karlsruhe. *Diadegma fenestrale* is known to attack the larvae of *Plutella xylostella*, one of the most relevant lepidopteran pest species of brassicaceous crops and has been introduced as a biocontrol agent in several countries in Europe^28,29^. *Plutella xylostella* (microlepidoptera) has a cosmopolitan distribution and was attracted to conventional LED 4000 K at Site 1 in September 2022.

***Amblyteles armatorius (Ichneumonidae)*** has a wide distribution across Europe (GBIF). The larvae of this species emerge from the pupae of two lepidopteran genera (*Xestia* and *Noctua*)^30^. *Amblyteles armatorius* was attracted to road lights at Site 1 (conventional LED 4000 K) in October 2022. Likewise, four potential host species *(X. c-nigrum*,* X. stigmata*,* X. xantographa* and *N. Pronuba)* were attracted to conventional LED 4000 K at Site 1 in August in 2021, July 2022 and September 2021 and 2022 ^31,32^.

In summary, the observed attraction effect of ALAN on adult life stages of several Lepidoptera parasitoids and hosts points to an influence of road lights on whole host-parasitoid-complexes (supporting H3). Subsequently, this suggests a large-scale influence of public outdoor lighting on parasitoid pressure on Lepidoptera populations. This could influence nocturnal pollination by moths and therefore plant reproductive success, which may cascade to the diurnal pollinator community^15,23^. We showed that tailored and shielded road lights effectively reduced unwanted light emission and parasitoid attraction in different real traffic and lighting scenarios, while fulfilling EU-norms. This will most likely also reduce an impact on Lepidoptera populations via parasitism under different environmental and lighting contexts and should be integrated into future lighting concepts.

### Potential Spotted Wing Drosophila (SWD) Parasitoids

***Pachycrepoideus vindemmiae *(*Pteromalidae*)** is an idiobiont ectoparasitic wasp attacking pupape of different cylorrhaphous flies^31^. It was one of the few parasitoids in total (16 of 80 in total at three sites in 2022 = 20%) that was attracted to the new tailored and shielded luminaires, in this case tailored LED 4000 K at the urban Site 1. Its potential as a biocontrol agent has been tested in several European countries, since it is one of the main parasitoids of the invasive Asian fruit fly *Drosophila Suzukii* Matsumara 1931 (SWD*)* in Europe, an economically important pest of soft fruits^32–34^. SWD was first detected in Italy and Spain in 2005 ^35^. In Germany, SWD was first reported in 2011 in a monitoring program of the Julius-Kühn-Institute (JKI), including collection sites in the upper rhine plain^36^. The successful invasiveness of SWD may be related to the absence of well adapted specific antagonists in the invaded ecosystem, but knowledge on the regulative efficiency of German natural enemy communities is scarce^37^. This is especially investigated for the upper Rhine plain by studies from Englert & Hertz (2017)^37^. In the laboratory, they reared natural enemies from fruit baited traps collected at JKI Dossenheim. SWD uses a wide range of habitats and host plants (soft/stone fruits), crops as well as and ornamentals^36,38^. Another main parasitoid of SWD in Europe is *Trichopria sp.* (*cf. drosophilae*) (*Diapriidae*)^34,37^. Undetermined *Diapriidae sp. (cf. Trichopria).* emerged at conventional LED 4000 K at Site 1 (urban) and tailored LED 2700 K at Site 2 (suburban).

Another potential SWD parasitoid is ***Asobara rufescens*** *(Braconidae)*. *Asobara rufescens* is reported from the Palearctic Region, but has been considered a junior synonym of *Asobara tabida* (Nees, 1834), which occurs in the Nearctic, Palearctic, Oriental, and Oceanic regions^39^. It appeared at conventional HPS 2000 K at Site 2 (suburban) in September 2022. The appearance of *Pachycrepoideus vindemmiae*,* Diapriidae sp. (*cf. *Trichopria).* and *Asobara rufescens* (or *Asobara tabida*) as potential natural enemies of SWD in this study corresponds with other European reports^33,37^. Interestingly, we observed another potential parasitoid of SWD, similarly originating from Asia and first reported in Europe from Italy in 2022 ^40^ and from Germany in 2023 ^41^, at the suburban Site 2:

***Leptopilina japonica* (*Figitidae*)** was first reported in several sites in southern and western Germany in 2023, potentially reflecting a case of unintentional biological control in Germany^41^. *Leptopilina japonica* was attracted in mid-August 2022 to conventional HPS 2000 K at Site 2 (suburban). Comparing sequences with BOLD database, we found our specimen to be conspecific with a specimen (BOLD process ID: GBHYG1714-23) from the study of Martin et al. (2023). Martin et al. (2023) caught *L. japonica* with sweep nets in consecutive years (2021–2022) in Bonn (North Rhine-Westphalia), but also from infested raspberries in Dossenheim in 2021 (Baden-Württemberg) and conclude that adventive populations may already be established^41^. The appearance of *L. japonica* in this study at Site 2 (approximately 10 km from JKI Dossenheim) supports this conclusion. To our knowledge, it is the first molecular report of an adult individuum collected under field conditions in the upper Rhein plain (GBIF). The species could have the potential to be used in integrated pest management of invasive SWD in a natural enemy naive environment. In summary, the appearance of different SWD parasitoids across different sites in Baden-Württemberg points to a large-scale impact of public outdoor lighting on adult life stages of potential biological control agents of pest species. In any case, we showed that tailored and shielded LED luminaires significantly reduced overall parasitoid attraction, which most likely reduces an impact on host interactions of biocontrol agents. However, further ALAN field research is needed here.

### Tritrophic interactions

In Europe, *Sitobium avenae*, *Rhopalosiphium padi* and *Metopolophium dirhodum* are among the most important aphid cereal pest species. These three aphid species are frequently attacked by a group of primary parasitoids (mostly *Braconidae*, *Aphidiinae*)^42^. These natural enemies can exert top-down control (biological control) on cereal aphid pest populations^43^, while in turn being attacked by secondary hyperparasitoids, influencing primary parasitoid mortality and consequently the levels of biological control^44^. For example, *Aphidius rhopalosiphi* is among the primary aphid parasitoids and was attracted to conventional HPS (2000 K) in June 2022 at Site 2 (suburban). Its antagonist the hyperparasitoid *Alloxysta victrix* (Figitidae) was attracted to conventional HPS 2000 K in June 2022 at Site 3 (rural)^45^. Despite the presence of the latter two species at different sites, their appearance on two consecutive nights in June 2022 (28.06.22/29.06.22) suggests a potential influence of road lights on tritrophic interactions by removing single individuals from their habitats. We showed that tailored and shielded LED luminaires reduce the attraction of parasitoid wasps, which is therefore expected to reduce an impact on tritrophic parasitoid complexes.

## Material & methods

### Light treatments

In a first step, nature reserves in Southern Germany were identified by categorisation into light pollution classes regarding sky brightness using night light satellite data of the “day-night band” (day and night band) of the instrument “visible infrared imaging radiometer suite” (VIIRS) on board of the Suomi-NPP satellite for recording upward directed light. For being able to make general statements on the suitability of a transition to tailored and shielded LED in aspects of insect conservation, protected areas (*n* = 3) were selected from different habitat-, landscape- and light pollution contexts (Fig. 1). A site-specific monitoring and a conversion concept were developed for the locations (e.g. number of luminaires, selection of masts/luminaires, position in relation to the nature reserve, conversion options) (Supporting Information Table S11). From April to October 2021–2022, a total of 28 flight interception traps were mounted close to the luminaire heads of individual luminaires at three differentially illuminated sites (Site 1 Alter Flugplatz LED 4000 K (*n* = 10), Site 2 Backofen Riedwiesen, high pressure sodium (HPS) cylinder luminaires 2000 K (*n* = 10), Site 3 Heimbachaue HPS bell-shaped luminaires 2000 K (*n* = 8). To ensure that the planned before-and-after comparison allows reliable statements to be made regarding the impact of light transformation on nocturnal insects, control investigations with the existing conventional lighting were carried out at all locations (Fig. 1). These control sites were as similar as possible (development, vegetation, traffic, lighting environment etc.) to the upgraded impact sites before the upgrade. Half of the trap-equipped lights at each location were converted to tailored and shielded LED luminaires between November 2021 and April 2022 (Site 1 Alter Flugplatz LED 4000 K, Site 2 Backofen Riedwiesen LED 2700 K, Heimbachaue LED 2700 K), while the other served as controls in 2022. Once on a monthly schedule, insects were sampled on consecutive nights (first night Site 1, second night Site 2, third night Site 3) close to half-moon (Supporting Information Table S11). For this purpose, containers with 80% alcohol were screwed to the bottom of the traps for the period between sunset and sunrise. During the winter season, the insect traps were dismantled and serviced to be reinstalled after the conversion. To rule out that the differences in colour temperature between conventional (HPS 2000 K) luminaires and the tailored and shielded luminaires (LED 2700 K) at Site 2 + 3 obscures the effect of an optimised light distribution, we additionally performed control experiments with the tailored and shielded luminaires and a reduced blue light content (LED, 2000 K) at Site 3. For this purpose, we attached amber foil to the exit window of the luminaire and adjusted illuminance to the same level as the control luminaires (2000 K, Em = 5.2 lx). From June to September, the tailored and shielded luminaires were modified in this way for the duration of one week after sampling with the original configuration (LED, 2700 K) and prior to five additional sampling events.

### Identification

Microscopes and binoculars were used for morphological identification. Depending on taxon, animals were identified to order, family, genus, or species level^46^. In preparation for molecular DNA-analyses (see next section), parasitoid Hymenoptera were transferred to 99.9% alcohol and send to AIM Gmbh Leipzig. We evaluated all specimens morphologically using different taxonomic keys (Supporting Information Identification literature). To evaluate sampling efficiency and to get an estimate on the minimum number of species, the individuals were assigned to morphotypes, representing different species based on morphological criteria. In a separate taxonomic examination, Lepidoptera (constituting potential host species with partly close relationships to foraging plants) were determined to species-level by Dr. Robert Trusch and Michael Falkenberg, Naturkundemuseum Karlsruhe). Lepidoptera are stored in the Lepidoptera collection at Naturkundemuseum Karlsruhe. Subsequently, we screened available literature and online literature resources (Google Scholar, Web of Science, Global Biodiversity Information Facility (GBIF)) to gain insight into species distribution and ecology as well as reliable molecular reports from Germany and Baden-Württemberg of the identified taxa.

### Barcoding

Target DNA sequences of the mitochondrial cytochrome C oxidase I (CO1) gene fragment of 106 parasitoid individuals were generated and analysed at Advanced Identification Methods (AIM GmbH, Leipzig, Germany^47^). Particularly, genomic DNA (gDNA) was extracted from whole individuals or parts stored in 99.9% alcohol using the Qiagen DNeasy^®^ Tissue Kits (Qiagen, Hilden, Germany). The gDNA was then amplified in a polymerase chain reaction (PCR) with 2 established primer combinations: LCO1490-JJ_M13f and HCO-1490-JJ_M13r ^48^ / dgLCO_M13f and dgHCO_M13r^49^). The generated PCR products were then processed on two ABI 3730 capillary sequencers with 50 cm capillary length. All raw DNA sequences were assembled and edited using Sequencher 4.10.1 (Gene Codes, Ann Arbor, MI, USA). Editing for each gene sequence was performed in MEGA v5.1 ^50^ and searched against the Barcode of life data system v3 (BOLD) and the National Centre for Biotechnology Information (NCBI) database using the Nucleotide collection (nt/nr) database of the Basic Local Alignment Search Tool BLAST^51,52^. For species identification, we defined a threshold of a 97% match with the reference sequence and compared the best hit with the next three hits for consistency. All involved specimens are stored as vouchers at Leibniz-Institute for Freshwater Ecology and Inland Fisheries, Müggelseedamm 310, 12784 Berlin.

### Phylogenetic analysis

Complimentary, we inferred a COI phylogenetic tree using the maximum likelihood optimality criterion as implemented in the software IQ-TREE version 1.6.12 ^53,54^ with default parameters. Particularly, for the phylogenetic data analyses all processed COI sequences were aligned using Mafft version 7 ^55^ before running IQ-TREE. The inferred tree was rooted by selecting the two *Ceraphronidae* specimens as phylogenetic outgroup.

### Statistics

Statistical analysis was carried out with R Studio Software Version 1.2.5033. In a first step, luminaires were assigned to site (paired control and treatment site at three study sites) (Compare Fig. 1). The control sites refer to the untransformed conventional control luminaires, the impact sites refer to luminaires that were converted in winter 2021–2022. Abundances were calculated by counting the number of parasitoid individuals per individual luminaire and year. Species richness was calculated by counting the number of species (individual morphotypes treated as species) per luminaire and year. Because we observed a general effect of the year (the 3.1-fold total parasitoid abundance in 2022 (after) compared to 2021 (before)), we proceeded modelling parasitoid abundance in a combined generalised linear model over three sites (to achieve robust sample sizes)^58^ in a before-after-control-impact (BACI) design^19^ (package “glmmTMB”^56^). Distribution test and model selection included “shapiro.test”, ”fitdistr” (“MASS” package^57^), “lmer”, ”anova” (“lmerTest” package^58^) and “simulateResiduals”, “testDispersion”, “testQuantiles”,”testOutliers”,”testZeroInflation” of the “DHARMA” package (residual diagnostics for hierarchical (multi-level/mixed) regression models^59^). Hypothesis testing was carried out using the “anova” function of the “stats” package^60^, backward selection of fixed effects and comparing AIC. We constructed GLMMs to determine how the abundance and species richness of parasitoid wasps (dependant variables) were affected by period (before-after) and treatment (control-impact) along with their interaction (the BACI effect) (fixed effects)^61^. We used a poisson distribution and set the zeroinflation argument to 1. We designated 2021 as “before” and 2022 as “after,” and the two sites per study site as “impact” and “control” sites. We included the study site and the year as random effects. We additionally used an observation-level (luminaire) random effect. To be able to make pairwise comparison between all combinations of period and treatment, we combined these predictors into a single fixed effect (= treatment group) in models with the identical structure of random effects^19,61,62^. All models were checked for overdispersion, zeroinflation and autocorrelation of residuals. Post-hoc tests (“emmeans” package, method ”Tukey”^63^) were computed to reveal the differences between individual treatment groups. Plotting was done with “ggplot”^64^.

## Electronic supplementary material

Below is the link to the electronic supplementary material.

Supplementary Material 1

## Data Availability

All data supporting the results is accessible via DOI 10.6084/m9.figshare.26206229; Files names: File 1: Lepidoptera.xlsx: Identification results Lepidoptera; File 2: Identification results and Fasta sequences.xlsx: The source data behind Supporting Information Table S8, S9; File 3: Parasitoids_wide_format.csv: The source data behind Figs. 3 and 4; File 4: Parasitoids_long_format.csv: The source data behind Fig. 2; File 5: R-script Parasitoids.txt: R-Code statistical analysis.
