# Supplementary material for: Shedding light on dark taxa: exploring a cryptic diversity of parasitoid wasps affected by artificial light at night
Source: Sci Rep. 2025 Feb 20;15:6237. doi: 10.1038/s41598-025-88111-3 (PMC11842737; doi:10.1038/s41598-025-88111-3)
Supplement: Supplementary file 1 — Supplementary Material 1 [file 41598_2025_88111_MOESM1_ESM.pdf]

Supporting Information

Manuscript: Shedding light on Dark taxa: Exploring a cryptic diversity of parasitoid wasps affected by Artificial Light at Night (ALAN)

Corresponding Author: Manuel Dietenberger. Leibniz-Institute of Freshwater Ecology and Inland Fisheries. Department 2 Community and Ecosystem Ecology. Light Pollution and Ecophysiology. Müggelseedamm 310. 12587 Berlin. Germany

E-Mail: manuel.dietenberger@igb-berlin.de

Tables: 11

Figures: 156

Table S1: Total number of Hymenoptera per family/subfamily attracted to road lights at three municipal sites from 2021-2022 in Southern Germany (Baden-Württemberg). Taxa with parasitoid lifestyle are printed in bold. Site 1 Alter Flugplatz, n=17 samplings (conversion from conventional LED 4000K to tailored LED 4000K), Site 2 Backofen Riedwiesen, n=15 samplings (conversion from conventional HPS cylinder 2000K to tailored LED 2700K). Site 3 Heimbachaue, n=23 samplings (conversion from conventional bell-shaped HPS 2000K to tailored LED 2700K/2000K).

Subfamily	Site 1	Site 2	Site 3	TOTAL
Apidae	1	0	0	1
Braconidae	**2**	**4**	**17**	**23**
Ceraphronidae	**2**	**1**	**0**	**3**
Crabronidae	2	0	0	2
Diapriidae	**2**	**1**	**3**	**6**
Encyrtidae	**3**	**1**	**2**	**6**
Eulophidae	**1**	**1**	**3**	**5**
Figitidae	**0**	**1**	**2**	**3**
Formicidae	161	61	28	250
Ichneumonidae	**7**	**5**	**4**	**16**
Mymaridae	**0**	**1**	**3**	**4**
Pirenidae	**0**	**1**	**0**	**1**
Platygastridae	**1**	**4**	**17**	**22**
Pompilidae	0	1	1	2
Proctotrupidae	**0**	**2**	**9**	**11**
Pteromalidae	**2**	**2**	**0**	**4**
Scelionidae	**2**	**0**	**0**	**2**
Sphecidae	0	1	0	1
Vespinae	8	31	10	49
**TOTAL**	**194**	**118**	**99**	**411**

Table S2: Generalised linear model showing the BACI effect on the total number of parasitoid wasps per road light (Abundance) 14 luminaires per groups. **2021**: n = 26 samplings (8 samplings Site 1 Alter Flugplatz, 8 samplings Site 2 Backofen Riedwiesen, 10 samplings Site 3 Heimbachaue; **2022**: n = 29 samplings (9 samplings Site 1 Alter Flugplatz, 7 samplings Site 2 Backofen Riedwiesen, Site 3 Heimbachaue 13 samplings). Treatment groups: Control sites (before): (conventional LED 4000K, HPS 2000K); Control sites (after): (conventional LED 4000K, HPS 2000K), Impact sites (before): (conventional LED 4000K, HPS 2000K); Impact sites after (tailored and shielded LED 4000K/2700K/2000K in 2022). P-values lower than 0.05 are printed in bold.

**Abundance ~Group**	**Estimate**	**SE**	**Z**	**P**
Intercept	1.2321	0.2146	5.741	9.44e-09 ***
Period (before/after)	-1.1962	0.3106	-3.851	**0.000118 *** **
Site (control/impact)	-1.4020	0.2852	-4.915	**8.87e-07 *** **
Period:Site	1.2665	0.4964	2.552	**0.010725 ***

Table S3: Generalised linear model showing the effect of the treatment group on the total number of parasitoid wasps per road light (Abundance) 14 luminaires per group. **2021**: n = 26 samplings (8 samplings Site 1 Alter Flugplatz, 8 samplings Site 2 Backofen Riedwiesen, 10 samplings Site 3 Heimbachaue; **2022**: n = 29 samplings (9 samplings Site 1 Alter Flugplatz, 7 samplings Site 2 Backofen Riedwiesen, Site 3 Heimbachaue 13 samplings). Treatment groups: Control sites (before): (conventional LED 4000K, HPS 2000K); Control sites (after): (conventional LED 4000K, HPS 2000K), Impact sites (before): (conventional LED 4000K, HPS 2000K); Impact sites after (tailored and shielded LED 4000K/2700K/2000K in 2022). P-values lower than 0.05 are printed in bold.

**Abundance ~Group**	**Estimate**	**SE**	**Z**	**P**
Control Before	0.005289	0.315992	1.271	0.986645
Impact Before	-0.160893	0.432054	0.678	0.709602
Control After	1.197170	0.2856	0.348528	**0.000593 *****
Impact After	-0.166899	0.2999	0.407114	0.681837

Table S4: Pairwise comparison (Tukey post hoc test of Generalised linear model) showing differences in the total number of parasitoid wasps per road light (Abundance) between treatment groups in a combined analysis over three municipal sites. 14 luminaires per group. **2021**: n = 26 samplings (8 samplings Site 1 Alter Flugplatz, 8 samplings Site 2 Backofen Riedwiesen, 10 samplings Site 3 Heimbachaue; **2022**: n = 29 samplings (9 samplings Site 1 Alter Flugplatz, 7 samplings Site 2 Backofen Riedwiesen, Site 3 Heimbachaue 13 samplings). Treatment groups: Control sites (before): (conventional LED 4000K, HPS 2000K); Control sites (after): (conventional LED 4000K, HPS 2000K), Impact sites (before): (conventional LED 4000K, HPS 2000K); Impact sites after (tailored and shielded LED 4000K/2700K/2000K in 2022). P-values lower than 0.05 are printed in bold.

**contrast**	Estimate	SE	Df	t-ratio	p-value
Control before- Impact before	0.160892942	0.4320538	56	0.37239098	0.9821998970
Control before - Control after	- 1.197170407	0.3485282	56	-3.43493086	**0.0060112231**
Control before - Impact after	0.166899144	0.4071136	56	0.40995718	0.9765212216
Impact before - Control after	- 1.358063349	0.3637251	56	- 3.73376290	**0.0024371413**
Impact before - Impact after	0.006006202	0.4205650	56	0.01428127	0.9999989400
Control after- Impact after	1.364069551	0.3213565	56	4.24472385	**0.0004716123**

Table S5: Generalised linear model showing the BACI effect on the total number of parasitoid wasp species per road light (Abundance) 14 luminaires per groups. **2021**: n = 26 samplings (8 samplings Site 1 Alter Flugplatz, 8 samplings Site 2 Backofen Riedwiesen, 10 samplings Site 3 Heimbachaue; **2022**: n = 29 samplings (9 samplings Site 1 Alter Flugplatz, 7 samplings Site 2 Backofen Riedwiesen, Site 3 Heimbachaue 13 samplings). Treatment groups: Control sites (before): (conventional LED 4000K, HPS 2000K); Control sites (after): (conventional LED 4000K, HPS 2000K), Impact sites (before): (conventional LED 4000K, HPS 2000K); Impact sites after (tailored and shielded LED 4000K/2700K/2000K in 2022). P-values lower than 0.05 are printed in bold.

**Abundance ~Group**	**Estimate**	**SE**	**Z**	**P**
Intercept	1.0144	0.1636	6.200	5.66e-10 ***
Period (before/after)	- 0.9944	0.3089	- 3.219	**0.001286 ****
Site (control/impact)	- 1.1148	0.2938	- 3.795	**0.000148 *****
Period:Site	0.9762	0.4987	1.958	0.050280

Table S6: Generalised linear model showing the effect of the treatment group on the total number of parasitoid wasp species per road light (Species richness) 14 luminaires per group. **2021**: n = 26 samplings (8 samplings Site 1 Alter Flugplatz, 8 samplings Site 2 Backofen Riedwiesen, 10 samplings Site 3 Heimbachaue; **2022**: n = 29 samplings (9 samplings Site 1 Alter Flugplatz, 7 samplings Site 2 Backofen Riedwiesen, Site 3 Heimbachaue 13 samplings). Treatment groups: Control sites (before): (conventional LED 4000K, HPS 2000K); Control sites (after): (conventional LED 4000K, HPS 2000K), Impact sites (before): (conventional LED 4000K, HPS 2000K); Impact sites after (tailored and shielded LED 4000K/2700K/2000K in 2022). P-values lower than 0.05 are printed in bold.

Species richness ~Group	Estimate	SE	Z	P
Control Before	8.045e-05	2.697e-01	0.000	0.99976
Impact After	-1.542e-01	3.934e-01	- 0.392	0.69517
Control Before	9.742e-01	3.074e-01	3.170	**0.00153****
Impact After	-1.244e-01	3.690e-01	- 0.337	0.73600

Table S7: Pairwise comparison (Tukey post hoc test of Generalised linear model) showing differences in the total number of parasitoid wasp species per road light (Species richness) between treatment groups in a combined analysis over three municipal sites. 14 luminaires per group. **2021**: n = 26 samplings (8 samplings Site 1 Alter Flugplatz, 8 samplings Site 2 Backofen Riedwiesen, 10 samplings Site 3 Heimbachaue; **2022**: n = 29 samplings (9 samplings Site 1 Alter Flugplatz, 7 samplings Site 2 Backofen Riedwiesen, Site 3 Heimbachaue 13 samplings). Treatment groups: Control sites (before): (conventional LED 4000K, HPS 2000K); Control sites (after): (conventional LED 4000K, HPS 2000K), Impact sites (before): (conventional LED 4000K, HPS 2000K); Impact sites after (tailored and shielded LED 4000K/2700K/2000K in 2022). P-values lower than 0.05 are printed in bold.

**contrast**	Estimate	SE	Df	t-ratio	p-value
Control before- Impact before	0.15415154	0.3933980	56	0.39184623	0.979385097
Control before - Control after	- 0.97421003	0.3073598	56	- 3.16960750	**0.012760656**
Control before - Impact after	0.12440110	0.3689671	56	0.33716043	0.986662119
Impact before - Control after	- 1.12836157	0.3261516	56	- 3.45962322	**0.005537354**
Impact before - Impact after	- 0.02975044	0.3847616	56	- 0.07732174	0.999832184
Control after- Control after	1.09861113	0.2886752	56	3.80570000	**0.001922537**

Table S8: Parasitoids attracted to road lights at three municipal sites in Southern Germany (Baden-Württemberg) from 2021-2022.

Superfamily	Family	Genus	Species/ Morphotypes	Luminaire	Municipal Site	Starting date	Sanger_Seq ID	Sample ID
Ceraphronoidea	*Ceraphronidae*	*Ceraphronidae*	*Ceraphronidae sp1.*	Tailored LED 4000K	Alter Flugplatz	220829	SQ_2022_057_037	KA_290822_C12A
			Ceraphronidae sp2.	Conventional LED 4000K	Alter Flugplatz	210927	SQ_2022_057_058	KA_270921_C9B
			Ceraphronidae sp3.	Conventional HPS 2000K	Backofen Riedwiesen	210902	SQ_2022_057_093	BR_020921_C25A
Chalcidoidea	*Encyrtidae*	*Blastothrix*	*Blastothrix sp.*	Conventional LED 4000K	Alter Flugplatz	220719	SQ_2022_057_013	KA_190722_C16A
		Copidosoma	*Copidosoma sp.*	Conventional HPS 2000K	Heimbachaue	220629	SQ_2022_057_001	HA_290622_C6A
			Encyrtidae sp1.	Conventional LED 4000K	Alter Flugplatz	210927	SQ_2022_057_034	KA_270921_C17A
			Encyrtidae sp1.	Tailored LED 2700K	Backofen Riedwiesen	220916	SQ_2022_057_049	BR_160922_C22A
			Encyrtidae sp2.	Conventional HPS 2000K	Heimbachaue	220629	SQ_2022_057_003	HA_290622_C7B
		Metaphycus	*Metaphycus sp.*	Tailored LED 4000K	Alter Flugplatz	220719	SQ_2022_057_028	KA_190722_C13A
Chalcidoidea	*Eulophidae*	*Aprostocetus*	*Aprostocetus sp.*	Conventional HPS 2000K	Backofen Riedwiesen	220816	SQ_2022_057_085	BR_160822_C27B
		Diglyphus	*Diglyphus isaea*	Conventional HPS 2000K	Heimbachaue	220601	SQ_2022_057_002	HA_010622_C5A
		Omphale	*Omphale lugens*	Conventional HPS 2000K	Heimbachaue	210612	SQ_2022_057_045	HA_120621_C1B
		Omphale	*Omphale radialis*	Conventional HPS 2000K	Heimbachaue	220824	SQ_2022_057_039	HA_240822_C8A
		Omphale	*Omphale sp.*	Conventional LED 4000K	Alter Flugplatz	210531	SQ_2022_057_022	KA_310521_C10A

Superfamily	Family	Genus	Species	Luminaire	Municipal Site	Starting date	Sanger_Seq ID	Sample ID
Chalcidoidea	*Mymaridae*	*Mymaridae*	*Mymaridae sp1.*	Conventional HPS 2000K	Heimbachaue	220824	SQ_2022_057_025	HA_240822_C5A
			Mymaridae sp1.	Conventional HPS 2000K	Heimbachaue	220824	SQ_2022_057_086	HA_240822_C7A
			Mymaridae sp2.	Tailored LED 2700K	Backofen Riedwiesen	220816	SQ_2022_057_027	KA_160822_C22A
			Mymaridae sp3.	Conventional HPS 2000K	Heimbachaue	220817	SQ_2022_057_074	HA_180822_C7A
Chalcidoidea	*Pirenidae*	*Gastrancistrus*	*Gastrancistrus sp.*	Conventional HPS 2000K	Backofen Riedwiesen	210902	SQ_2022_057_035	BR_020921_C22B
Chalcidoidea	*Pteromalidae*	*Cratomus*	*Cratomus megacephalus*	Conventional HPS 2000K	Backofen Riedwiesen	210602	SQ_2022_057_010	BR_020621_C24A
		Cyclogastrella	*Cyclogastrella sp.*	Conventional HPS 2000K	Backofen Riedwiesen	220816	SQ_2022_057_073	BR_160822_C27A
		Pachycrepoideus	*Pachycrepoideus vindemmiae*	Tailored LED 4000K	Alter Flugplatz	221017	SQ_2022_057_061	KA_171022_C9A
		Stenomalina	*Stenomalina sp.*	Conventional LED 4000K	Alter Flugplatz	220627	SQ_2022_057_015	KA_270622_C14A
Cynipoidea	*Figitidae*	*Alloxysta*	*Alloxysta sp.*	Conventional HPS 2000K	Heimbachaue	220518	SQ_2022_057_038	HA_180522_C6A
			Alloxysta victrix	Conventional HPS 2000K	Heimbachaue	220629	SQ_2022_057_050	HA_290622_C7A
		Leptopilina	*Leptopilina japonica*	Conventional HPS 2000K	Backofen Riedwiesen	220816	SQ_2022_057_062	BR_160822_C24A
Diaprionidea	*Diapriidae*		*Diapriidae sp1.*	Conventional HPS 2000K	Heimbachaue	220824	SQ_2022_057_066	HA_240822_C7H
			Diapriidae sp1.	Tailored LED 2000K	Heimbachaue	220726	SQ_2022_057_090	KA_260722_C2A
			Diapriidae sp2.	Conventional HPS 2000K	Heimbachaue	220824	SQ_2022_057_031	HA_240822_C7I

Superfamily	Family	Genus	Species	Luminaire	Municipal Site	Starting date	Sanger_Seq ID	Sample ID
Diaprionidea	*Diapriidae*		*Diapriidae sp2.*	Conventional LED 4000K	Alter Flugplatz	210927	SQ_2022_057_046	KA_270921_C9A
			Diapriidae sp3.	Conventional LED 4000K	Alter Flugplatz	220627	SQ_2022_057_078	KA_270622_C15B
			Diapriidae sp2.	Tailored LED 2700K	Backofen Riedwiesen	220816	SQ_2022_057_019	BR_160822_C21B
Ichneumonoidea	*Braconidae*	*Alious*	*Alious lepidus*	Conventional HPS 2000K	Backofen Riedwiesen	220628	SQ_2022_057_068	BR_280622_C27A
			Alysiinae sp.	Tailored LED 2000K	Heimbachaue	220726	SQ_2022_057_095	HA_260722_C4A
		Apantales	*Apantales sodalis*	Conventional HPS 2000K	Backofen Riedwiesen	210902	SQ_2022_057_023	BR_020921_C22A
Ichneumonoidea	*Braconidae*	*Aphidius*	*Aphidius rhopalosiphi*	Conventional HPS 2000K	Backofen Riedwiesen	220628	SQ_2022_057_048	BR_280622_C26A
		Asobara	*Asobara rufescens*	Conventional HPS 2000K	Backofen Riedwiesen	210902	SQ_2022_057_047	BR_020921_C24A
		Blacus	*Blacus ruficornis*	Conventional HPS 2000K	Heimbachaue	220629	SQ_2022_057_008	HA_290622_C5A
			Blacus ruficornis	Conventional HPS 2000K	Heimbachaue	220629	SQ_2022_057_012	HA_290622_C8A
			Blacus ruficornis	Conventional HPS 2000K	Heimbachaue	220726	SQ_2022_057_044	HA_260722_C7C
			Blacus ruficornis	Conventional HPS 2000K	Heimbachaue	220824	SQ_2022_057_072	HA_240822_C7J
			Blacus ruficornis	Conventional HPS 2000K	Heimbachaue	220629	SQ_2022_057_098	HA_290622_C6B
			Blacus ruficornis	Conventional HPS 2000K	Heimbachaue	220721	SQ_2022_057_032	HA_210722_C6C
			Blacus ruficornis	Conventional HPS 2000K	Heimbachaue	220721	SQ_2022_057_043	HA_210722_C6A

Superfamily	Family	Genus	Species	Luminaire	Municipal Site	Starting date	Sanger_Seq ID	Sample ID
Ichneumonoidea	*Braconidae*	*Blacus*	*Blacus ruficornis*	Conventional HPS 2000K	Heimbachaue	220721	SQ_2022_057_091	HA_210722_C6B
			Blacus ruficornis	Conventional HPS 2000K	Heimbachaue	220726	SQ_2022_057_067	HA_260722_C7B
			Blacus ruficornis	Conventional HPS 2000K	Heimbachaue	220726	SQ_2022_057_079	HA_260722_C8B
			Braconidae sp.	Conventional HPS 2000K	Heimbachaue	220726	SQ_2022_057_020	HA_260722_C5A
		Dinotrema	*Dinotrema sp.*	Conventional HPS 2000K	Heimbachaue	220629	SQ_2022_057_024	HA_290622_C8B
			Dinotrema sp.	Conventional HPS 2000K	Heimbachaue	220721	SQ_2022_057_055	HA_210722_C8A
			Dinotrema sp.	Conventional LED 4000K	Alter Flugplatz	220719	SQ_2022_057_092	KA_190722_C17A
		Dolichogenidea	*Dolichogenidea sp.*	Conventional HPS 2000K	Heimbachaue	220629	SQ_2022_057_036	HA_290622_C8C
		Eubazus	*Eubazus sp.*	Conventional LED 4000K	Alter Flugplatz	210927	SQ_2022_057_082	KA_270921_C10A
		Lipolexis	*Lipolexis sp.*	Conventional HPS 2000K	Heimbachaue	210612	SQ_2022_057_069	HA_120621_C5A
		Meteorus	*Meteorus affinis*	Conventional HPS 2000K	Heimbachaue	220726	SQ_2022_057_084	HA_260722_C5B
Ichneumonoidea	*Ichneumonidae*	*Acrotomus*	*Acrotomus succinctus*	Conventional LED 4000K	Alter Flugplatz	210927	SQ_2022_057_101	KA_270921_C14A
		Amblyteles	*Amblyteles armatorius*	Conventional LED 4000K	Alter Flugplatz	221017	SQ_2022_057_100	KA_171022_C15A
		Aptesis	*Aptesis assimilis*	Conventional HPS 2000K	Backofen Riedwiesen	210902	SQ_2022_057_081	BR_020921_C27A
		Barycnemis	*Barycnemis angustipennis*	Conventional HPS 2000K	Heimbachaue	220831	SQ_2022_057_104	HA_310822_C8A

Superfamily	Family	Genus	Species	Luminaire	Municipal Site	Starting date	Sanger_Seq ID	Sample ID
Ichneumonoidea	*Ichneumonidae*	*Cratichneumon*	*Cratichneumon sp.*	Conventional HPS 2000K	Backofen Riedwiesen	220830	SQ_2022_057_105	BR_300822_C27A
			Ctenopelmatinae sp.	Conventional LED 4000K	Alter Flugplatz	210927	SQ_2022_057_097	KA_270921_C16A
		Diadegma	*Diadegma fenestrale*	Conventional LED 4000K	Alter Flugplatz	210927	SQ_2022_057_070	KA_270921_C15A
		Endasys	*Endasys sp.*	Conventional HPS 2000K	Heimbachaue	210818	SQ_2022_057_106	HA_180821_C1A
		Gelis	*Gelis sp.*	Conventional HPS 2000K	Backofen Riedwiesen	210602	SQ_2022_057_059	BR_020621_C23A
			Gelis sp.	Conventional HPS 2000K	Heimbachaue	210804	SQ_2022_057_009	HA_040821_C2A
			Gelis sp.	Conventional LED 4000K	Alter Flugplatz	210816	SQ_2022_057_011	KA_160821_C18A
			Gelis sp.	Conventional LED 4000K	Alter Flugplatz	220915	SQ_2022_057_056	KA_150922_C15A
		Metopius	*Metopius fuscipennis*	Conventional HPS 2000K	Backofen Riedwiesen	220830	SQ_2022_057_103	BR_300822_C26A
		Netelia	*Netelia sp.*	Tailored LED 2000K	Heimbachaue	220726	SQ_2022_057_102	HA_260722_C1A
		Stenomacrus	*Stenomacrus affinitor*	Conventional HPS 2000K	Backofen Riedwiesen	220628	SQ_2022_057_060	BR_280622_C24A
		Temelucha	*Temelucha sp.*	Conventional LED 4000K	Alter Flugplatz	220627	SQ_2022_057_099	KA_270622_C15A
Platygastroidea	*Platygastridae*	*Amblyaspis*	*Amblyaspis sp.*	Conventional HPS 2000K	Backofen Riedwiesen	210602	SQ_2022_057_071	BR_020621_C22A
			Amblyaspis sp.	Conventional HPS 2000K	Backofen Riedwiesen	220830	SQ_2022_057_064	BR_300822_C28A
			Amblyaspis sp.	Conventional HPS 2000K	Heimbachaue	210612	SQ_2022_057_033	HA_120621_C1A

Superfamily	Family	Genus	Species	Luminaire	Municipal Site	Starting date	Sanger_Seq ID	Sample ID
Platygastroidea	*Platygastridae*	*Amblyaspis*	*Amblyaspis sp.*	Conventional HPS 2000K	Heimbachaue	210831	SQ_2022_057_021	HA_310821_C5A
			Amblyaspis sp.	Conventional HPS 2000K	Heimbachaue	220705	SQ_2022_057_005	HA_050722_C7A
			Amblyaspis sp.	Conventional HPS 2000K	Heimbachaue	220705	SQ_2022_057_088	HA_050722_C5A
			Amblyaspis sp.	Conventional HPS 2000K	Heimbachaue	220726	SQ_2022_057_006	HA_260722_C8A
			Amblyaspis sp.	Conventional HPS 2000K	Heimbachaue	220824	SQ_2022_057_029	HA_240822_C5C
			Amblyaspis sp.	Conventional HPS 2000K	Heimbachaue	220824	SQ_2022_057_030	HA_240822_C7E
			Amblyaspis sp.	Conventional HPS 2000K	Heimbachaue	220824	SQ_2022_057_041	HA_240822_C6D
			Amblyaspis sp.	Conventional HPS 2000K	Heimbachaue	220824	SQ_2022_057_042	HA_240822_C7F
			Amblyaspis sp.	Conventional HPS 2000K	Heimbachaue	220824	SQ_2022_057_053	HA_240822_C7B
			Amblyaspis sp.	Conventional HPS 2000K	Heimbachaue	220824	SQ_2022_057_054	HA_240822_C7G
			Amblyaspis sp.	Conventional HPS 2000K	Heimbachaue	220824	SQ_2022_057_065	HA_240822_C7C
			Amblyaspis sp.	Conventional HPS 2000K	Heimbachaue	220824	SQ_2022_057_077	HA_240822_C7D
			Amblyaspis sp.	Conventional HPS 2000K	Heimbachaue	220824	SQ_2022_057_089	HA_240822_C5D
			Amblyaspis sp.	Conventional LED 4000K	Alter Flugplatz	220531	SQ_2022_057_040	KA_310522_C17A
			Amblyaspis sp.	Tailored LED 2700K	Backofen Riedwiesen	220816	SQ_2022_057_018	BR_160822_C21A

Superfamily	Family	Genus	Species	Luminaire	Municipal Site	Starting date	Sanger_Seq ID	Sample ID
Platygastroidea	*Platygastridae*	*Inostemma*	*Inostemma sp.*	Conventional HPS 2000K	Backofen Riedwiesen	210602	SQ_2022_057_083	BR_020621_C24B
			Inostemma sp.	Tailored LED 2700K	Heimbachaue	220817	SQ_2022_057_052	HA_180822_C2A
			Platygastridae sp.	Tailored LED 2700K	Heimbachaue	220518	SQ_2022_057_017	HA_180522_C1A
	Scelionidae	*Synopeas*	*Synopeas sp.*	Tailored LED 2000K	Heimbachaue	220824	SQ_2022_057_076	HA_240822_C3A
		Scelionidae	*Scelionidae sp1.*	Conventional LED 4000K	Alter Flugplatz	220719	SQ_2022_057_014	KA_190722_C14A
			Scelionidae sp2.	Conventional LED 4000K	Alter Flugplatz	210630	SQ_2022_057_094	KA_300621_C16A
Proctotrupoidea	*Proctotrupidae*	*Exallonyx*	*Exallonyx nixoni*	Conventional HPS 2000K	Heimbachaue	220726	SQ_2022_057_051	HA_260722_C6A
		Proctotrupes	*Exallonyx sp.*	Conventional HPS 2000K	Heimbachaue	220726	SQ_2022_057_007	HA_260722_C7A
			Proctotrupes sp.	Tailored LED 2700K	Backofen Riedwiesen	220628	SQ_2022_057_080	BR_280622_C23A
			Proctotrupes sp.	Tailored LED 2700K	Backofen Riedwiesen	220628	SQ_2022_057_107	BR_280622_C19A
			Proctotrupinae sp.	Conventional HPS 2000K	Heimbachaue	210612	SQ_2022_057_057	HA_120621_C7A
			Proctotrupinae sp.	Conventional HPS 2000K	Heimbachaue	220831	SQ_2022_057_026	HA_310822_C6A
			Proctotrupinae sp.	Conventional HPS 2000K	Heimbachaue	220824	SQ_2022_057_016	HA_240822_C5B
			Proctotrupinae sp.	Conventional HPS 2000K	Heimbachaue	220824	SQ_2022_057_063	HA_240822_C6A
			Proctotrupinae sp.	Conventional HPS 2000K	Heimbachaue	220824	SQ_2022_057_075	HA_240822_C6B

Superfamily	Family	Genus	Species	Luminaire	Municipal Site	Starting date	Sanger_Seq ID	Sample ID
Proctotrupoidea	*Proctotrupidae*		*Proctotrupinae sp.*	Conventional HPS 2000K	Heimbachaue	220824	SQ_2022_057_087	HA_240822_C6C
			Proctotrupinae sp.	Tailored LED 2000K	Heimbachaue	220824	SQ_2022_057_004	HA_240822_C2A

Table S9: List of identified species examined both morphologically and molecularly. Abbreviations: AF=Alter Flugplatz, BR=Backofen Riedwiesen, HA=Heimbachaue, IGB=Leibniz Institute Berlin, Müggelseedamm 310, AIM=Advanced Identification GmbH Leipzig

Locality	Species	Specimen-ID	Luminaire	AIM-ID	Collection Date	Sequencer	Sequence length (bp)	Depository	Sequence Data repository
AF	*Acrotomus succinctus (Gravenhorst 1829)*	KA_270921_C14A	Conventional LED 4000K	SQ_2022_057_101	27.09.21	AIM	603	IGB	10.6084/m9.figshare.26206229
BR	*Aliolus lepidus (Haliday 1835)*	BR_280622_C27A	Conventional HPS 2000K cylinder	SQ_2022_057_068	28.06.22	AIM	654	IGB	10.6084/m9.figshare.26206229
HA	*Alloxysta victrix (Westwood 1833)*	HA_290622_C7A	Conventional HPS 2000K bow-shaped	SQ_2022_057_050	29.06.22	AIM	637	IGB	10.6084/m9.figshare.26206229
AF	*Amblyteles armatorius (Forster 1771)*	KA_171022_C15A	Conventional LED 4000K	SQ_2022_057_100	17.10.22	AIM	606	IGB	10.6084/m9.figshare.26206229
BR	*Apanteles sodalis (Haliday 1834)*	BR_020921_C22A	Conventional HPS 2000K cylinder	SQ_2022_057_023	02.09.21	AIM	587	IGB	10.6084/m9.figshare.26206229
BR	*Aphidius rhopalosiphi (De Stefani Perez 1902)*	BR_280622_C26A	Conventional HPS 2000K cylinder	SQ_2022_057_048	28.06.22	AIM	639	IGB	10.6084/m9.figshare.26206229
BR	*Aptesis assimilis (Gravenhorst 1829)*	BR_020921_C27A	Conventional HPS 2000K cylinder	SQ_2022_057_081	02.09.21	AIM	625	IGB	10.6084/m9.figshare.26206229
BR	*Asobara rufescens (Forster 1862)*	BR_020921_C24A	Conventional HPS 2000K cylinder	SQ_2022_057_047	03.09.21	AIM	614	IGB	10.6084/m9.figshare.26206229

Locality	Species	Specimen-ID	Luminaire	AIM-ID	Collection Date	Sequencer	Sequence length (bp)	Depository	Sequence Data repository
HA	*Barycnemis angustipennis (Holmgren 1860)*	HA_310822_C8A	Conventional HPS 2000K bow-shaped	SQ_2022_057_104	31.08.22	AIM	579	IGB	10.6084/m9.figshare.26206229
HA	*Blacus ruficornis (Nees 1811)*	HA_290622_C5A	Conventional HPS 2000K bow-shaped	SQ_2022_057_008	29.06.22	AIM	635	IGB	10.6084/m9.figshare.26206229
HA	*Blacus ruficornis (Nees 1811)*	HA_290622_C8A	Conventional HPS 2000K bow-shaped	SQ_2022_057_012	29.06.22	AIM	598	IGB	10.6084/m9.figshare.26206229
HA	*Blacus ruficornis (Nees 1811)*	HA_260722_C7C	Conventional HPS 2000K bow-shaped	SQ_2022_057_044	26.07.22	AIM	307	IGB	10.6084/m9.figshare.26206229
HA	*Blacus ruficornis (Nees 1811)*	HA_240822_C7J	Conventional HPS 2000K bow-shaped	SQ_2022_057_072	24.08.22	AIM	634	IGB	10.6084/m9.figshare.26206229
BR	*Cratomus megacephalus (Fabricius 1793)*	BR_020621_C24A	Conventional HPS 2000K cylinder	SQ_2022_057_010	02.06.21	AIM	644	IGB	10.6084/m9.figshare.26206229
AF	*Diadegma fenestrale (Holgren 1860)*	KA_270921_C15A	Conventional LED 4000K	SQ_2022_057_070	27.09.21	AIM	647	IGB	10.6084/m9.figshare.26206229
HA	*Diglyphus isaea (Walker 1838*	HA_010622_C5A	Conventional HPS 2000K bow-shaped	SQ_2022_057_002	01.06.22	AIM	646	IGB	10.6084/m9.figshare.26206229
HA	*Exallonyx nixoni (Townes 1981)*	HA_260722_C6A	Conventional HPS 2000K bow-shaped	SQ_2022_057_051	26.07.22	AIM	301	IGB	10.6084/m9.figshare.26206229

Loc ality	Species	Specimen -ID	Luminaire	AIM-ID	Collect ion Date	Sequen cer	Sequen ce length (bp)	Depository	Sequence Data repository
BR	*Leptopilina japonica (Novković & Kimura 2011)*	BR_16082 2_C24A	Conventional HPS 2000K cylinder	SQ_2022 _057_06 2	16.08.2 2	AIM	598	IGB	10.6084/m9. figshare.262 06229
HA	*Meteorus affinis (Wesmael 1835)*	HA_2607 22_C5B	Conventional HPS 2000K bow-shaped	SQ_2022 _057_08 4	26.07.2 2	AIM	618	IGB	10.6084/m9. figshare.262 06229
BR	*Metopius fuscipennis (Wesmael 1849)*	BR_30082 2_C26A	Conventional HPS 2000K cylinder	SQ_2022 _057_10 3	30.08.2 2	AIM	612	IGB	10.6084/m9. figshare.262 06229
HA	*Omphale lugens (Nees 1834)*	HA_1206 21_C1B	Conventional HPS 2000K bow-shaped	SQ_2022 _057_04 5	12.06.2 1	AIM	573	IGB	10.6084/m9. figshare.262 06229
HA	*Omphale radialis (Thomson 1878)*	HA_2408 22_C8A	Conventional HPS 2000K bow-shaped	SQ_2022 _057_03 9	24.08.2 2	AIM	278	IGB	10.6084/m9. figshare.262 06229
AF	*Pachycrepoideus vindemmiae (Rondani 1875)*	KA_1710 22_C9A	Tailored LED 4000K	SQ_2022 _057_06 1	12.10.2 2	AIM	297	IGB	10.6084/m9. figshare.262 06229
BR	*Stenomacrus affinitor (Aubert 1981)*	BR_28062 2_C24A	Conventional HPS 2000K cylinder	SQ_2022 _057_06 0	28.06.2 2	AIM	633	IGB	10.6084/m9. figshare.262 06229

Table S10: Identified species, distribution in Germany and Baden-Württemberg. Species not previously reported in Baden-Württemberg in the literature or the Global Biodiversity Information Facility (GBIF) are printed in bold and shaded in grey.

Family/Subfamily	Species	Host	Presence/ Absence Germany GBIF	Presence/Absence Baden-Württemberg GBIF	Other Reports
Ichneumonidae Tryphoninae	*Acrotomus succinctus*	*Symphyta* [1]	(+)	(+)	[2] (Germany: Baden-Württemberg); [3] (Britain); [4] (Switzerland); [5] (Netherlands)
Braconidae Brachistinae	***Aliolus lepidus***	**Coleoptera** [6]	**(+)**	**(-)**	[7] **(Germany);** [6] **(Bulgaria, Czech Republic, Germany);** [8] **(Germany: Schleswig-Holstein, Lower Saxony)**
Figitidae Charipinae	*Alloxysta victrix*	*Hyperparasitoid Aphidiinae* [8]	(+)	(-)	[9] (Germany: Baden-Württemberg); [10] (Norway); [8] (Germany: Schleswig-Holstein, Lower Saxony)
Ichneumonidae Ichneumoninae	*Amblyteles armatorius*	*Lepidoptera* [11] *Xestia, Noctua*	(+)	(+)	[4] (Switzerland); [3](Britain)
Braconidae Microgastrinae	***Apanteles sodalis***	***Lepidoptera*** [1] ***Tortricidae***	**(-)**	**(-)**	[7] **(Germany); Aydoğdu (2014)** [12] **(Turkey)**
Braconidae Aphidiinae	***Aphidius rhopalosiphi***	***Aphididae*** [13]	**(+)**	**(-)**	[7] **(Germany);** [8] **(Germany: Schleswig-Holstein, Lower Saxony)**
Ichneumonidae Cryptinae	*Aptesis assimilis*	Symphyta [14]	(+)	(+)	[15] (Germany: Baden-Württemberg); [3] (Britain)
Braconidae Alysiinae	***Asobara rufescens***	**Diptera** [16]	**(+)**	**(-)**	[7] **(Germany);** [16] **(North-America)**
Braconidae Tersilochinae	*Barycnemis angustipennis*	Coleoptera [17]	(+)	**(+)**	[15] (Germany: Baden-Württemberg); Klopfstein (2019) [4] (Switzerland); [3] (Britain)
Braconidae Brachistinae	***Blacus ruficornis***	**Coleoptera** [14]	**(+)**	**(-)**	[7] **(Germany);** [18] **(Britain, Germany: Bavaria)**
Pteromalidae Pteromalinae	*Cratomus megacephalus*	Unknown [19]	(+)	(+)	[20] (Germany), [21] (Germany); [19] (Poland [22] (Romania)

Family/Subfamily	Species	Host	Presence/ Absence Germany GBIF	Presence/Absence Baden-Württemberg GBIF	Other Reports
Ichneumonidae Campopleginae	*Diadegma fenestrale*	*Lepidoptera* [23,24] *Plutella xylostella*	(+)	**(-)**	[3] (Britain); [4] (Switzerland); [25] (Germany: Baden-Württemberg)
Eulophidae Eulophinae	***Diglyphus isaea***	***Diptera*** [26]	**(+)**	**(-)**	[20] **(Germany);** [26] **(Netherlands)**
Proctotrupidae Proctotrupinae	***Exallonyx nixoni***	**Unknown** [14,27]	**(+)**	**(-)**	[28] **(Iran);** [27] **(Britain)**
Figitidae Eucoilinae	*Leptopilina japonica*	*Diptera* [29]	(+)	(+)	[29] (Germany: Baden-Württemberg); [30] (Italy); [16] (North-America)
Braconidae Euphorinae	***Meteorus affinis***	***Lepidoptera*** [31]	**(-)**	**(-)**	[7] **(Germany);** [31]**, Stigenberg & Shaw (2013) (Europe, Korea, China, Russia)**
Ichneumonidae Metopiinae	*Metopius fuscipennis*	*Lepidoptera* [14]	(+)	(+)	[4] (Switzerland); [15] (Germany: Baden-Württemberg)
Eulophidae Entedoniae	***Omphale lugens***	***Cecidomyiidae (Diptera)*** [32]	**(+)**	**(-)**	[20] **(Germany);** [33] **(Netherlands)**
Eulophidae Entedoniae	***Omphale radialis***	***Cecidomyiidae (Diptera)*** [32]	**(+)**	**(-)**	[20] **(Germany);** [33] **(Netherlands, France, Germany: Northrhine-Westphalia, Rheinland-Pfalz)**
Pteromalidae Pachyneurinae	*Pachycrepoideus vindemmiae*	*Diptera* [34]	**(-)**	**(-)**	[35] (Germany: Schleswig-Holstein); [20] (Germany); [34] (Germany: Baden-Württemberg); [36] (Switzerland); [16] (North-America)
Ichneumonidae Orthocentrinae	***Stenomacrus affinitor***	**Unknown** [1]	**(+)**	**(-)**	[37] **(Finland);** [38]**(Iran)**

Table S11: Classification of existing lighting before (2021) and after (2022) conversion of half of the luminaires at three municipal sites in Baden-Württemberg.

	**Site 1 Alter Flugplatz. Karlsruhe**	**Backofen Riedwiesen. Brühl**	**Heimbachaue. Betzweiler**
**Coordinates**	49.02107968828992. 8.37779407517844	49.41185086276461. 8.519436152709618	48.363683514004585. 8.481609229369308
**Habitat**	Dry and sandy Oligotrophic grasslands	Damp meadows. Wet floodplains	Wet Floodplain vegetation. Semi-arid grassland
**Traffic usage**	Cycle path. No motorised vehicles. pedestrians	Residential street <30km/h. Motorised vehicles + sidewalk	marketplace + parking lot. Slow Motorised vehicles
**Pollution class**	Extreme	Very high	Low
**Light regime**	Urban	Peri-urban	Rural
		**2021**	
**Luminaire**	Trilux Lumega 700	Abele Geiger Trend	Hess Barcelona
**Illuminant**	LED	HPS	HPS
**Mean (lux)**	9.5	3.1	5.1
**Height (m)**	4.5	3.5	3.5
**Spectrum (K)**	4000	2000	2000
**Number**	10	10	8
**Sample events**	8	8	10 (3 nights with part-time-illumination (technical problems)
		**2022**	
**Luminaire**	Selux Tal + shield	Selux Tal + shield	Selux Beta + shield
**Illuminant**	LED	LED	LED
**Mean (lux)**	9.2	3.1	5.2
**Height (m)**	4.5	3.5	3.5
**Spectrum (K)**	4000	2700	2700
**Number**	5	5	4
**Controls**	5	5	4
**Sample events**	9	7	8 (+ 5 samplings with 2000K)

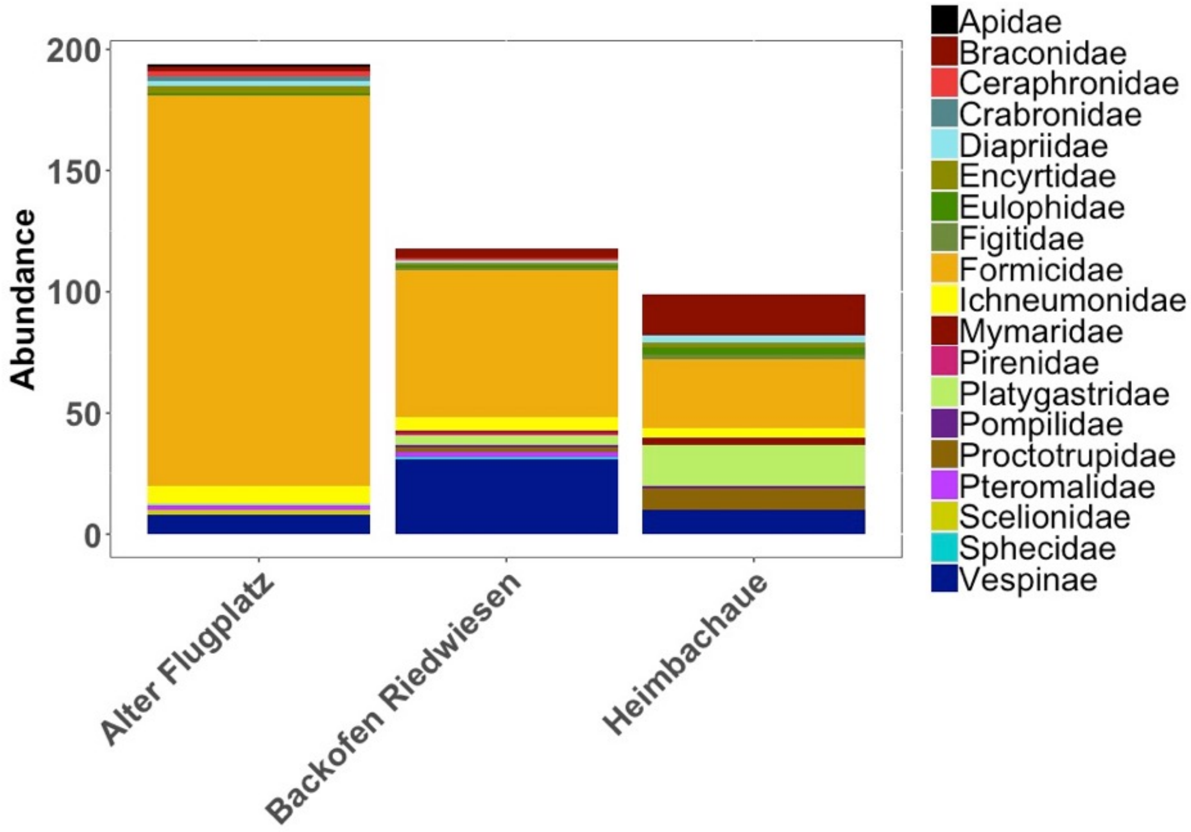

Figure S1: Total number of *Hymenoptera* per family/subfamily (colour coded) attracted to road lights at three municipal sites from 2021-2022 in Southern Germany (Baden-Württemberg). Site 1 Alter Flugplatz n=17 samplings, 10 luminaires per treatment (conversion from conventional LED 4000K to tailored LED 4000K), Site 2 Backofen Riedwiesen n=15 samplings, 10 luminaires per treatment (conversion from conventional HPS cylinder 2000K to tailored LED 2700K). Site 3 Heimbachaue = 23 samplings, 4 luminaires per treatment (conversion from conventional bell-shaped HPS 2000K to tailored LED 2700K (includes 5 control samplings with tailored LED in a 2000K configuration).

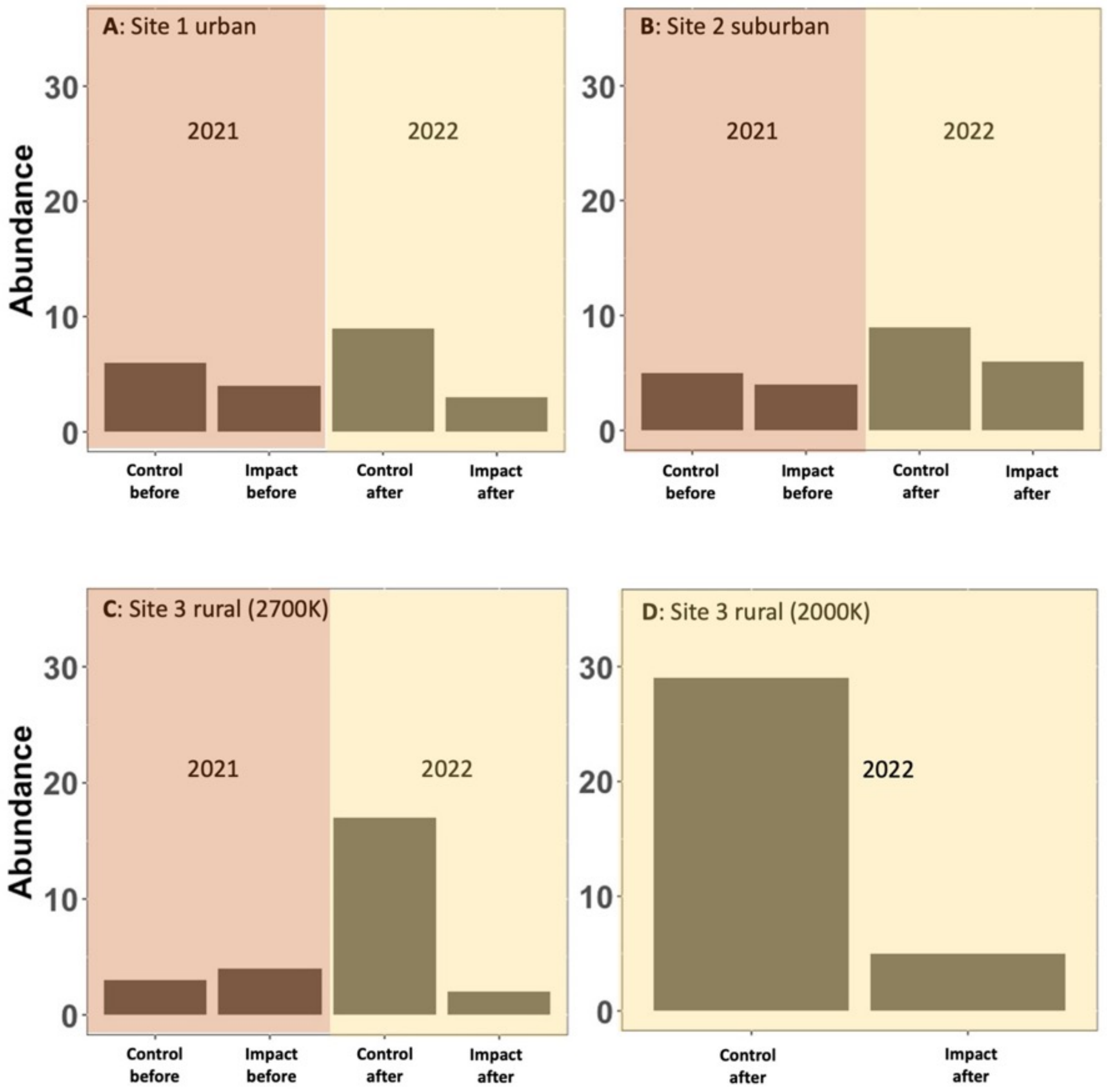

Figure S2: Total number of parasitoid wasps (bars) per control/impact sites before (2021) and after (2022) conversion of the luminaires at the impact sites to tailored and shielded LED. **A**: Site 1 Alter Flugplatz, urban, n=8 samplings 2021,9 samplings 2022, 5 luminaires per control/impact (conversion from conventional LED 4000K to tailored LED 4000K), B**:** Site 2 Backofen Riedwiesen n=7 samplings 2021, 8 samplings 2022, 5 luminaires per control/impact (conversion from conventional HPS cylinder 2000K to tailored LED 2700K). **C**: Site 3 Heimbachaue n=10 samplings 2021, 8 samplings 2022, 4 luminaires per control/impact (conversion from conventional bell-shaped HPS 2000K to tailored LED 2700K, **D**: Site 3

Heimbachaue: 5 samplings, 4 luminaires per control/impact (HPS 2000K vs. tailored LED 2000K).

Acrotomus succinctus (Ichneumonidae)

Sanger_ID: SQ_2022_057_101
Data_ID: KA_270921_C14A (conventional LED 4000K)

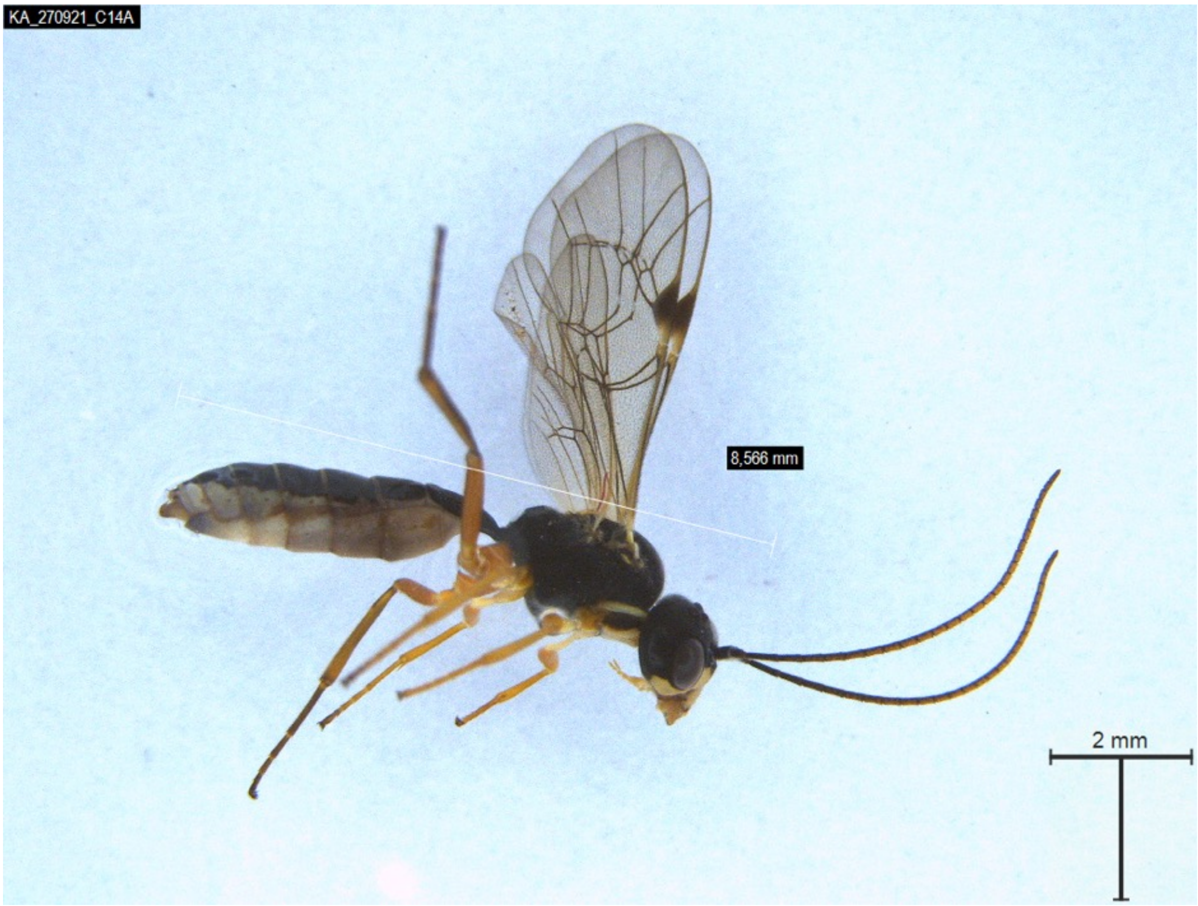

Figure S3*: Acrotomus succinctus*

SPECIES | ACCEPTED

Acrotomus succinctus (Gravenhorst, 1829)

Published in: Gravenhorst, J.L.C. Ichneumonologia Europaea. Pars II. Vratislaviae. 989 pp. (1829).
source: Taxapad Ichneumonoidea

Ichneumonid wasp In English **Basionym:** *Tryphon succinctus* Gravenhorst, 1829

260 OCCURRENCES 1 INFRASPECIES

OVERVIEW METRICS REFERENCE TAXON

16 OCCURRENCES WITH IMAGES

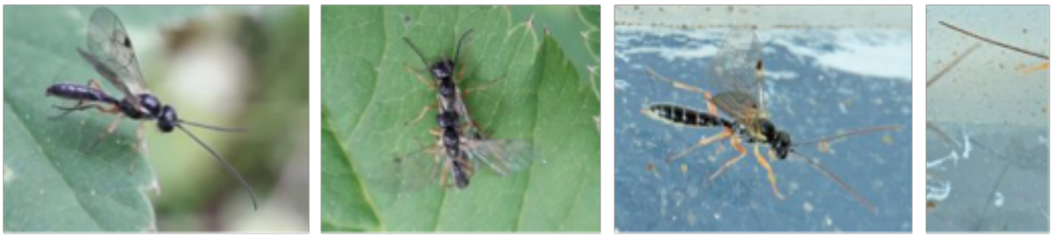

154 GEOREFERENCED RECORDS

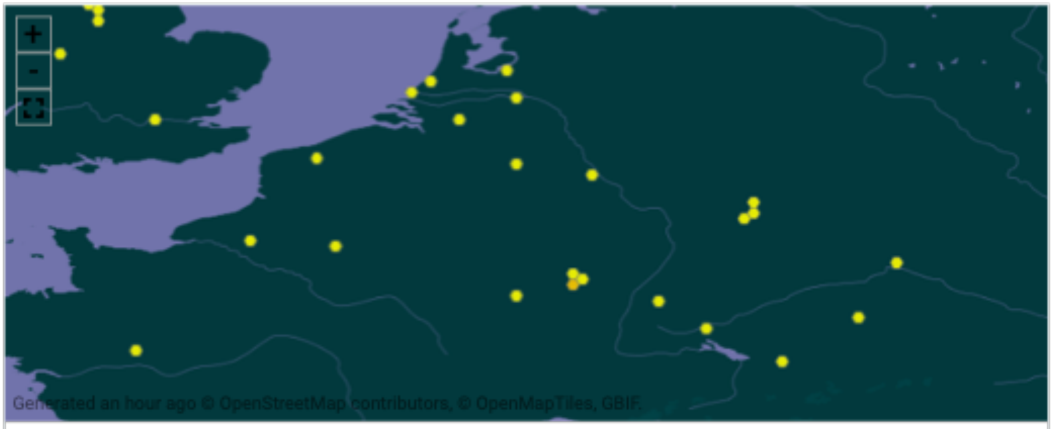

Any year 1915 - 2022 EXPLORE AREA

RECORDED AS INTRODUCED IN 1 COUNTRY OR ISLAND

Recorded as introduced in	According to	Evidence of impact	Occurrences in GBIF
United States of America	Global Register of Introduced and Invasive Species - United States (Contiguous) (ver.2.0, 2022)	No	Unknown

NAME USAGES APPLIED TO OCCURRENCES IN GBIF

Figure S4: Global Biodiversity Information Facility (GBIF) Webpage *Acrotomus Succinctus*

Aliolus lepidus (Braconidae)

Sanger_ID: SQ_2022_057_068
Data_ID: BR_280622_C27A (conventional HPS 2000K)

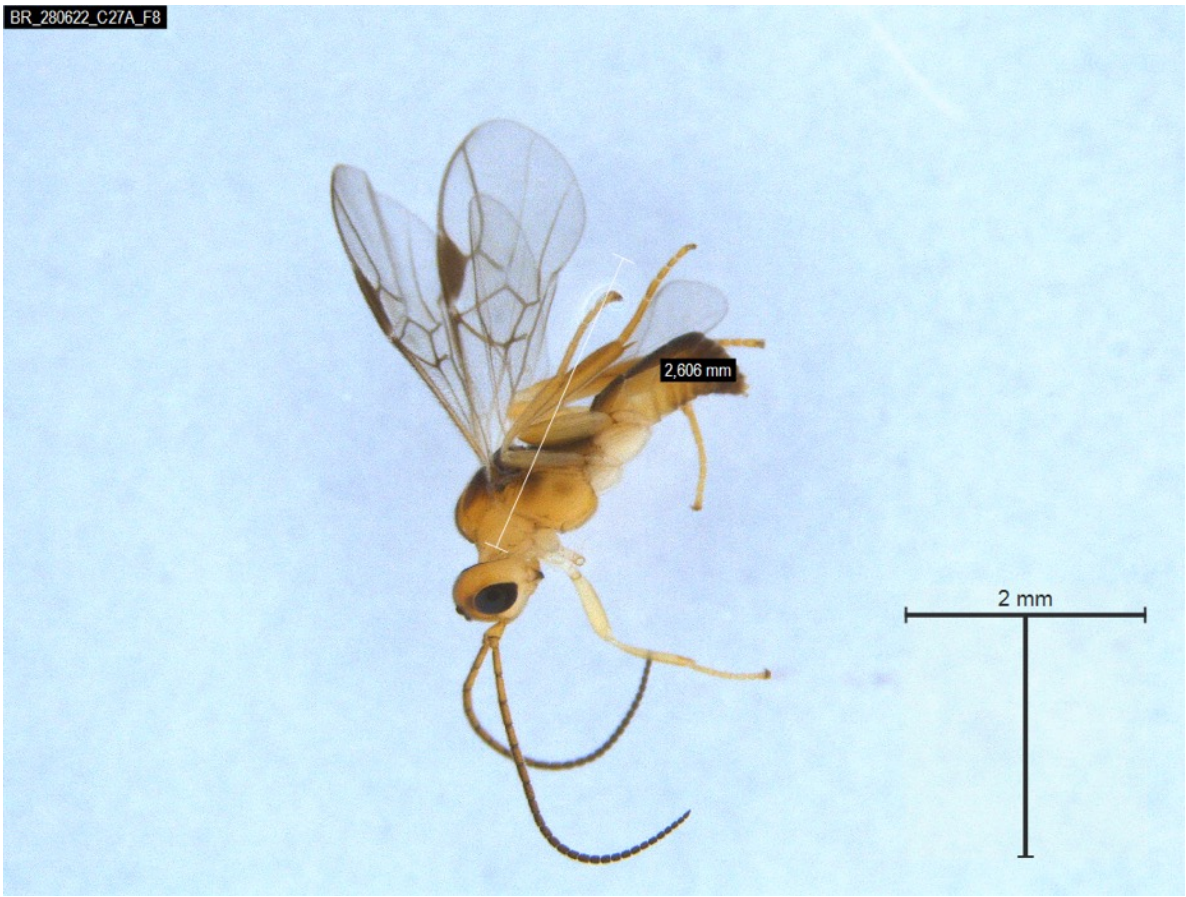

Figure S5*:* *Aliolus lepidus*

16/11/2023, 14:22 Aliolus lepidus

SPECIES | ACCEPTED

Aliolus lepidus

In: GBIF Backbone Taxonomy

10 OCCURRENCES 1 INFRASPECIES

OVERVIEW METRICS

2 GEOREFERENCED RECORDS

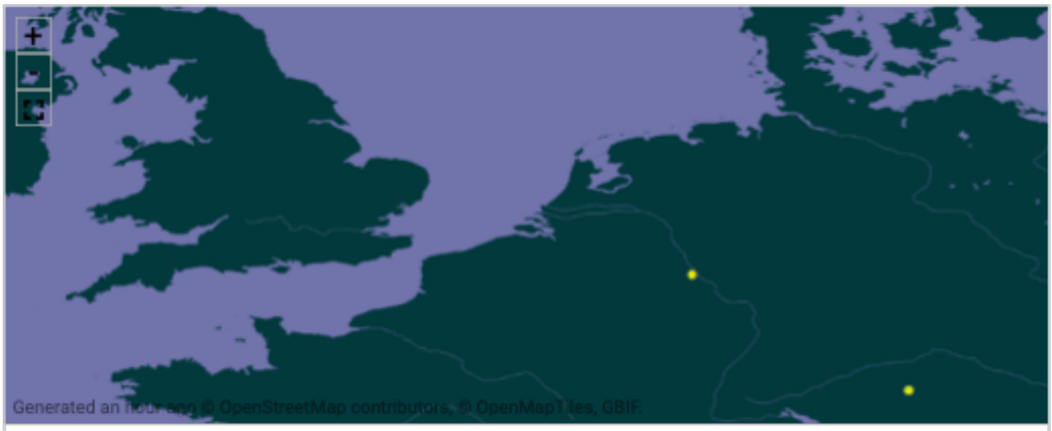

Any year 2014 - 2017 EXPLORE AREA

APPEARS IN 4 CHECKLIST DATASETS:

GBIF Backbone Taxonomy
As *Aliolus lepidus*

NCBI Taxonomy
As *Aliolus lepidus*

The European Nucleotide Archive (ENA) taxonomy
As *Aliolus lepidus*

International Barcode of Life project (iBOL) Barcode Index Numbers (BINs)
As *Aliolus lepidus*

APPEARS IN 2 OCCURRENCE DATASETS:

International Barcode of Life project (iBOL)

https://www.gbif.org/species/10090350 1/2

Figure S6: Global Biodiversity Information Facility (GBIF) Webpage *Aliolus lepidus*

Alloxysta sp. (Figitidae)

Sanger_ID: SQ_2022_057_038
Data_ID: HA_180522_C6A (conventional HPS 2000K)

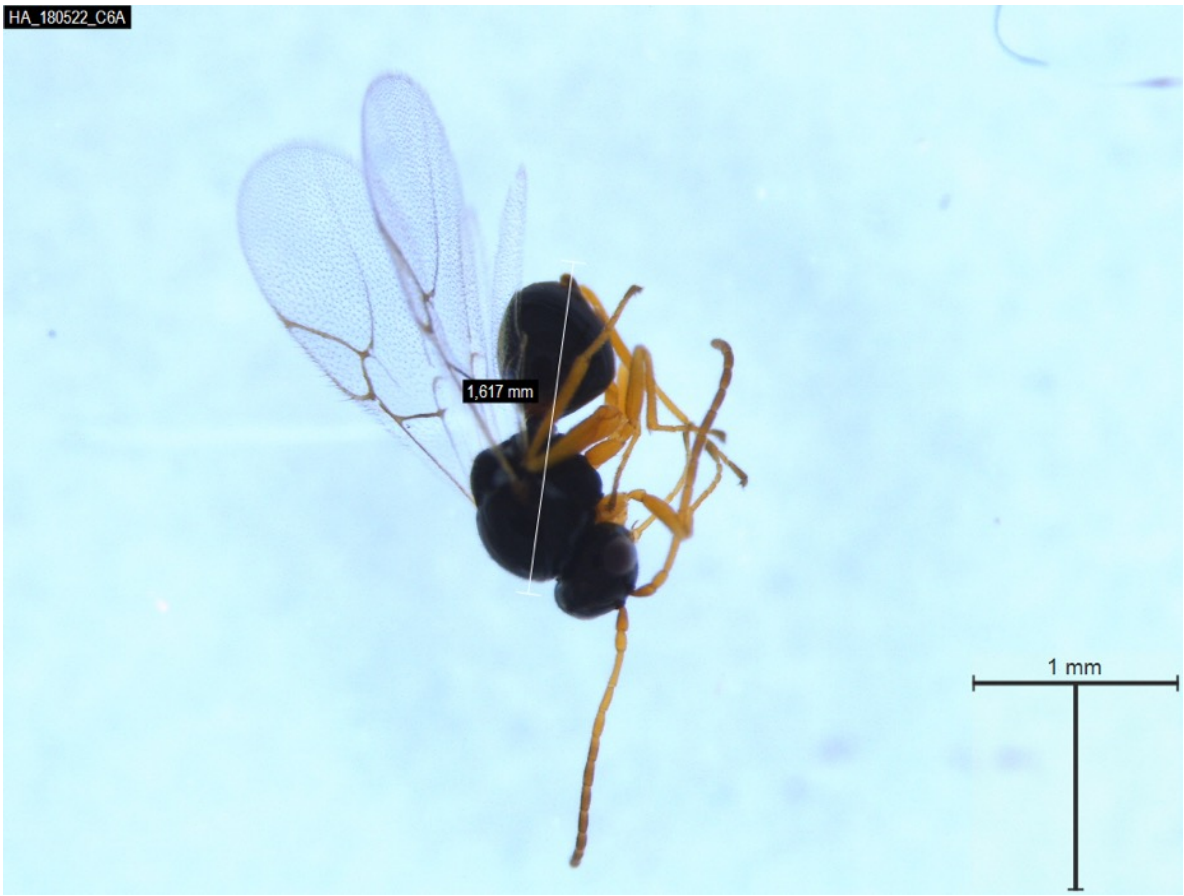

Figure S7: *Alloxysta sp.*

GENUS | ACCEPTED

Alloxysta Förster, 1869

Published in: Förster, Arnold. 1869. Ueber die Gallwespen. Verhandlungen der Kaiserlich-Königlichen Zoologisch-Botanischen Gesellschaft in Wien 19: 327-370.
source: A list of the terrestrial fungi, flora and fauna of Madeira and Selvagens archipelagos

5,292 OCCURRENCES 116 SPECIES

OVERVIEW 5 TREATMENTS METRICS REFERENCE TAXON

97 OCCURRENCES WITH IMAGES

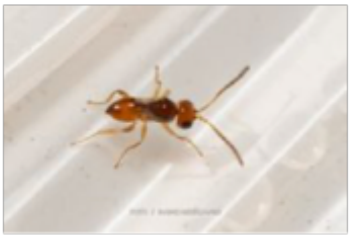
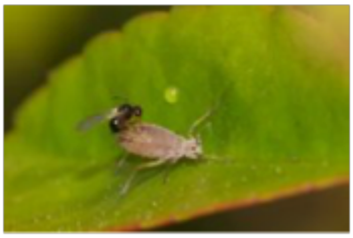
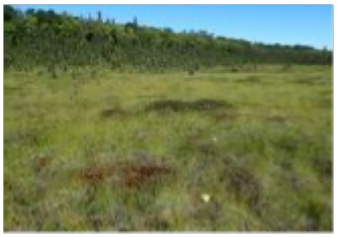

1,299 GEOREFERENCED RECORDS

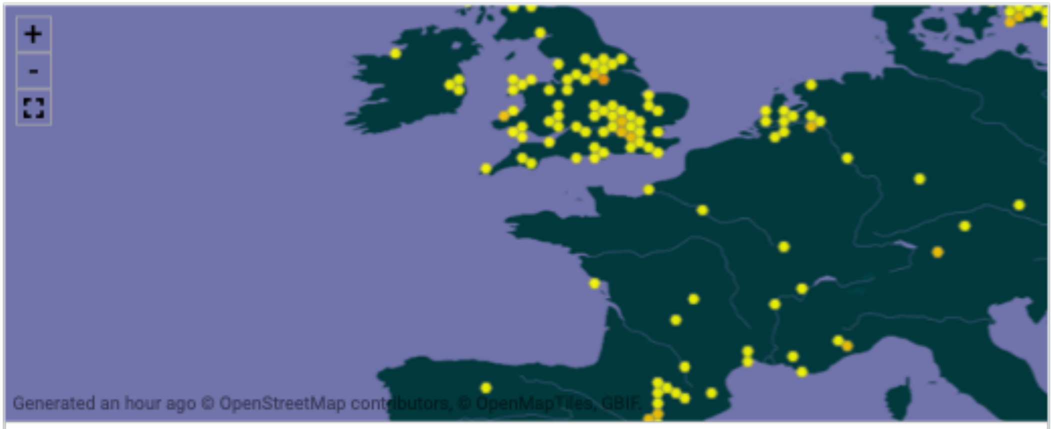

Any year 1800 - 2023 EXPLORE AREA

APPEARS IN 26 CHECKLIST DATASETS:

GBIF Backbone Taxonomy
As *Alloxysta* Förster, 1869

NCBI Taxonomy
As *Alloxysta*

Figure S8: Global Biodiversity Information Facility (GBIF) Webpage *Alloxysta*

Alloxysta victrix (Figitidae)

Sanger_ID: SQ_2022_057_050
Data_ID: HA_290622_C7A (conventional HPS 2000K)

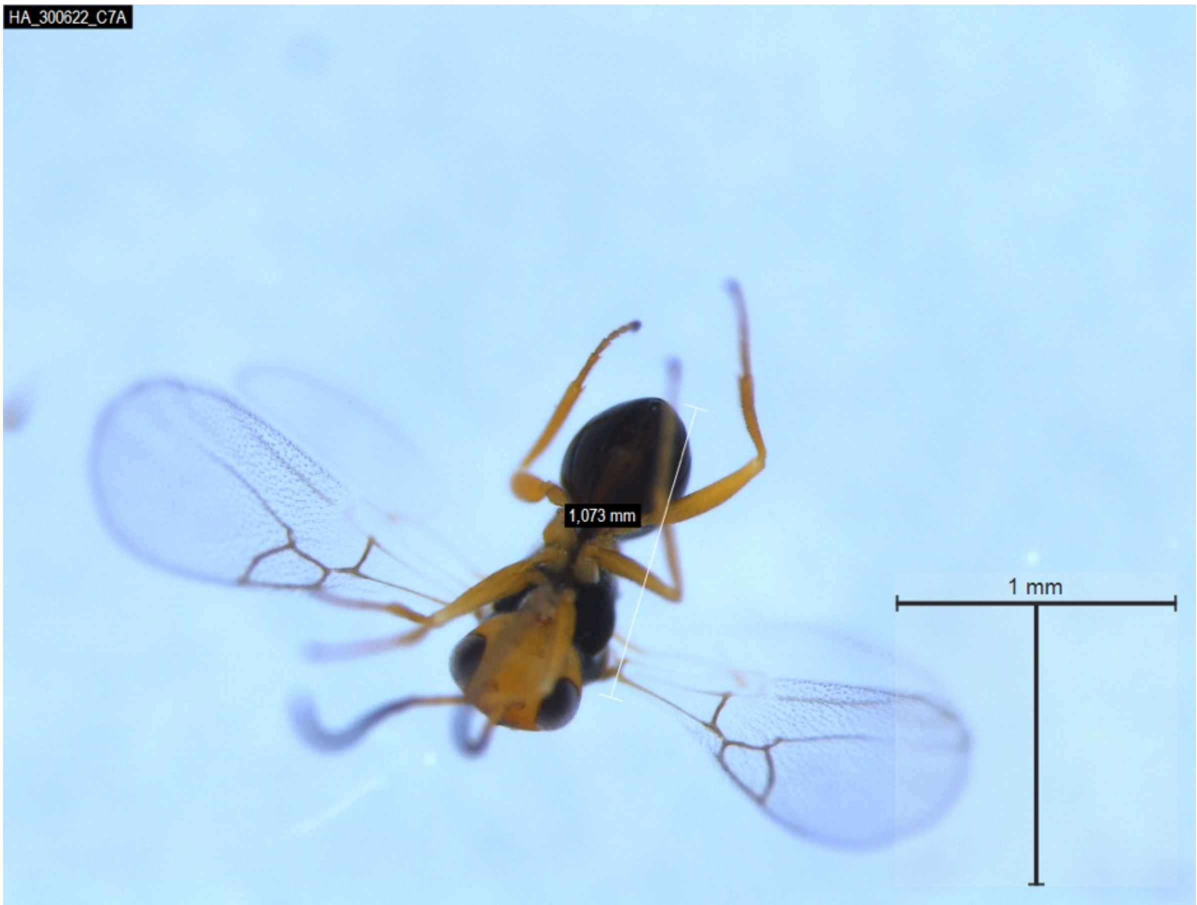

Figure S9: *Alloxysta victrix*

SPECIES | ACCEPTED

Alloxysta victrix (Westwood, 1833)

source: A list of the terrestrial fungi, flora and fauna of Madeira and Selvagens archipelagos

Basionym: *Allotria victrix* Westwood, 1833

669 OCCURRENCES 15 INFRASPECIES

OVERVIEW 3 TREATMENTS METRICS REFERENCE TAXON

8 OCCURRENCES WITH IMAGES

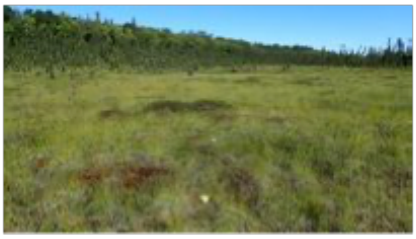

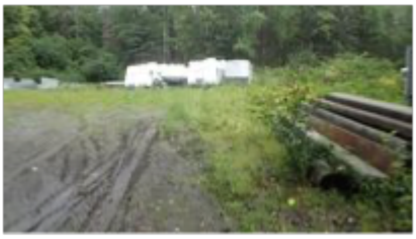

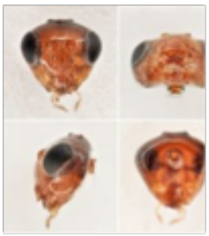

292 GEOREFERENCED RECORDS

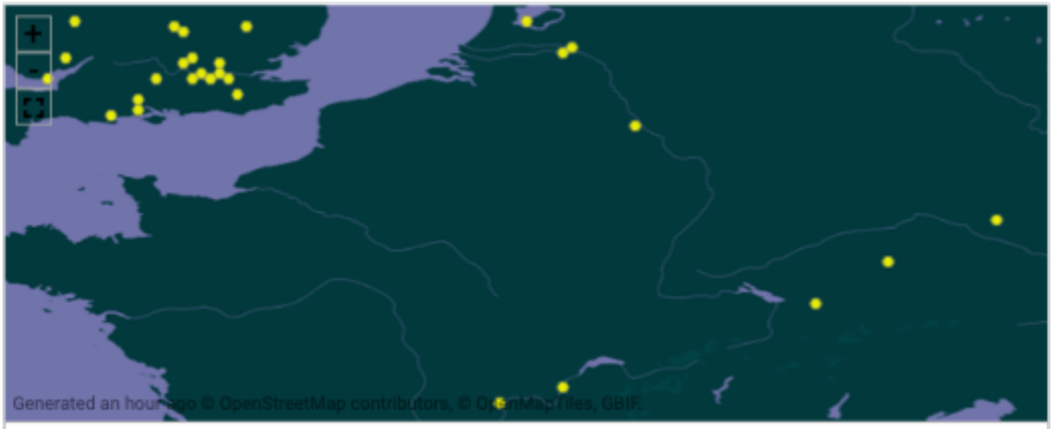

Any year 1868 - 2023 EXPLORE AREA

TYPE SPECIMENS

HOLOTYPE of Alloxysta victrix (Westwood, 1833) B. M. Type Hym..
03BD87FFFF9DA00FF307F89EF32D6CD4.mc.3B7C3CB4FF9AA00FF1A5FEECF46D6F9F
Source: Revision of the types of species of Alloxysta described by Cameron and Fergusson (Hymenoptera: Figitidae: Charipinae) and deposited in the Natural History Museum (London), including a key to the fauna of Great Britain

LECTOTYPE of Alloxysta victrix (Westwood, 1833) NMV Entomology T21211
Source: Museums Victoria provider for OZCAM

LECTOTYPE of Alloxysta victrix (Westwood, 1833) QM Entomology T207019

Figure S10: Global Biodiversity Information Facility (GBIF) Webpage *Alloxysta victrix*

Amblyaspis sp. (Platygastridae)

GENUS | ACCEPTED

Amblyaspis Förster, 1856

Published in: Foerster, Arnold. 1856. Hymenopterologische Studien. II. Heft. Chalcidiae und Proctotrupii. Ernst ter Meer, Aachen.: 1-152.

In: GBIF Backbone Taxonomy

1,122 OCCURRENCES 65 SPECIES

OVERVIEW 3 TREATMENTS METRICS

9 OCCURRENCES WITH IMAGES

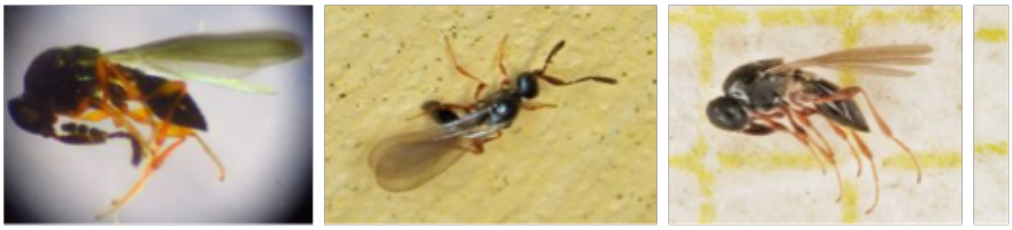

241 GEOREFERENCED RECORDS

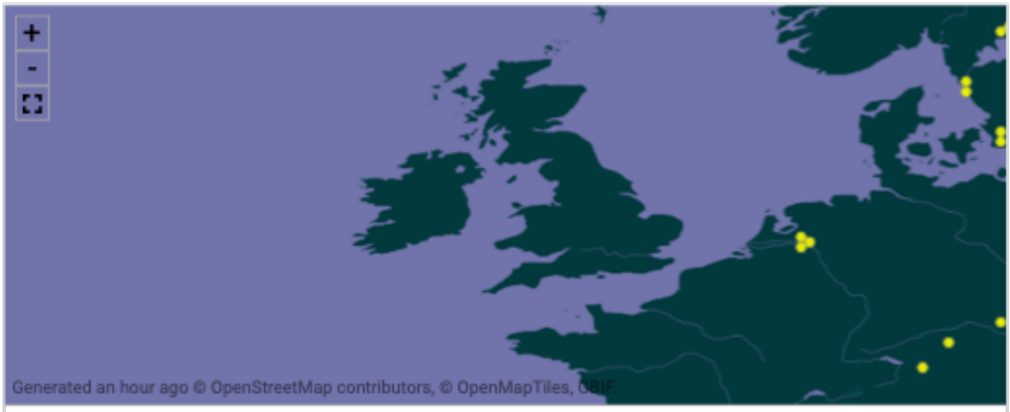

Any year 1870 - 2023 EXPLORE AREA

APPEARS IN 22 CHECKLIST DATASETS:

GBIF Backbone Taxonomy
As *Amblyaspis* Förster, 1856

Catalogue of Life Checklist
As *Amblyaspis* Förster, 1856

Figure S11: Global Biodiversity Information Facility (GBIF) Webpage *Amblyaspis*

Sanger_ID: SQ_2022_057_005
Data_ID: HA_050722_C7A (conventional HPS 2000K)

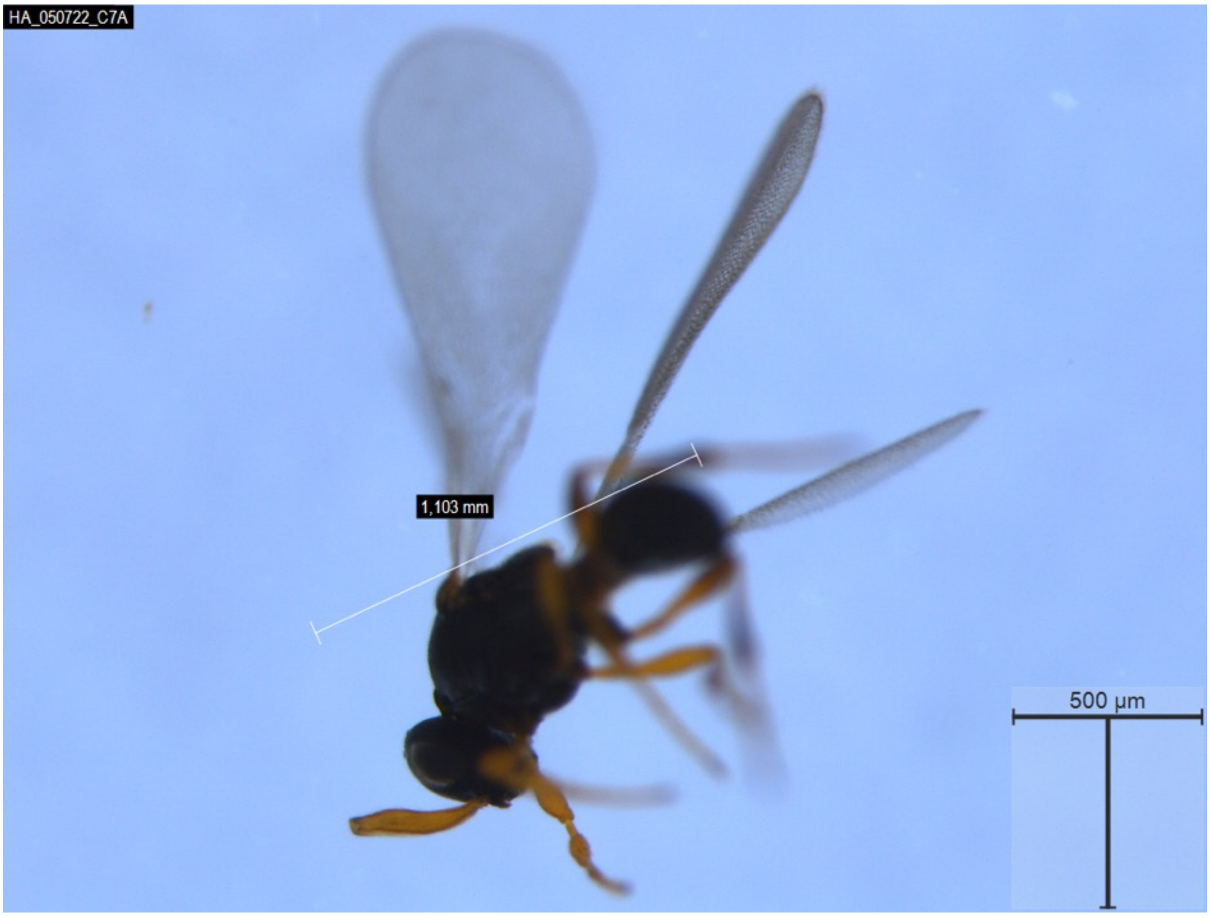

Figure S12: *Amblyaspis sp.*

Sanger-ID: SQ_2022_057_006
Data-ID: HA_260722_C8A (conventional HPS 2000K)

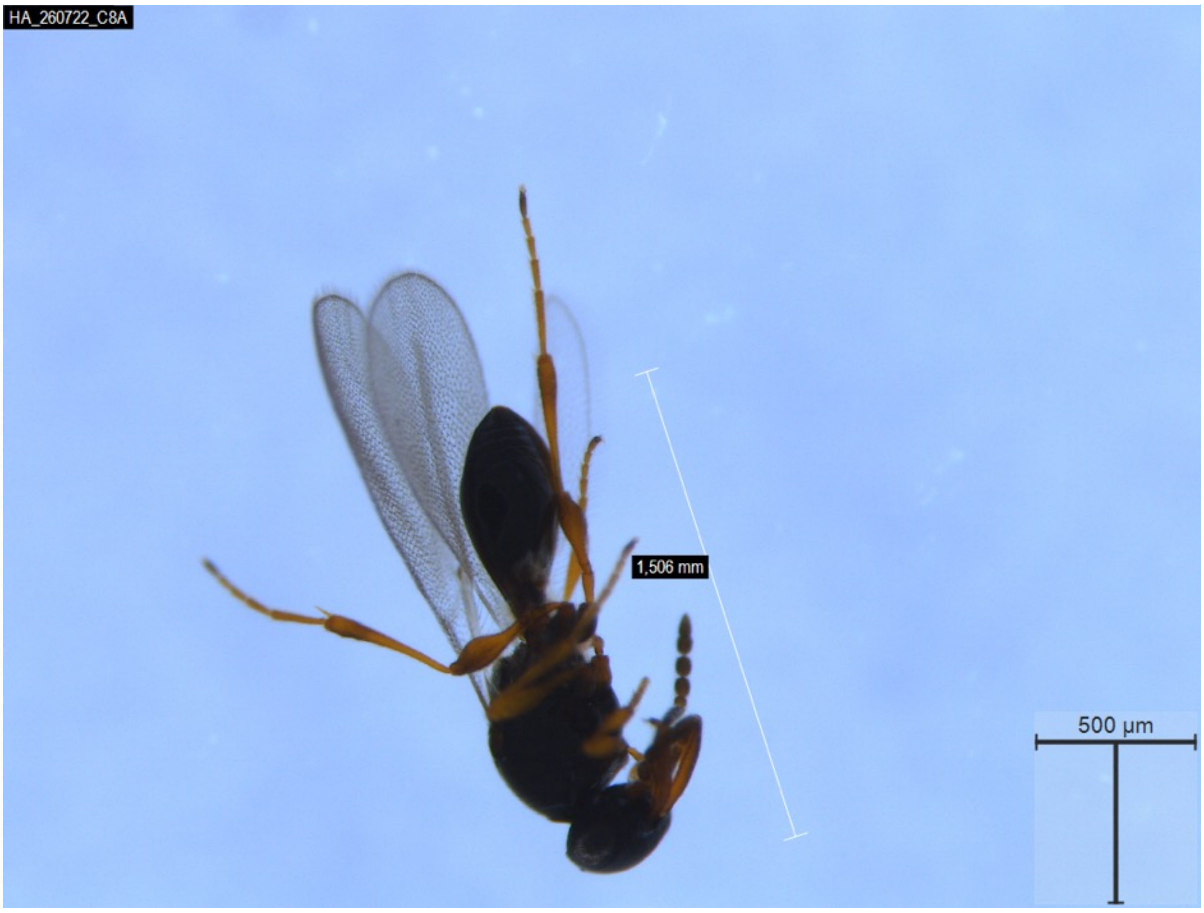

Figure S13: *Amblyaspis sp.*

Sanger-ID: SQ_2022_057_018
Data-ID: BR_160822_C21A (conventional HPS 2000K)

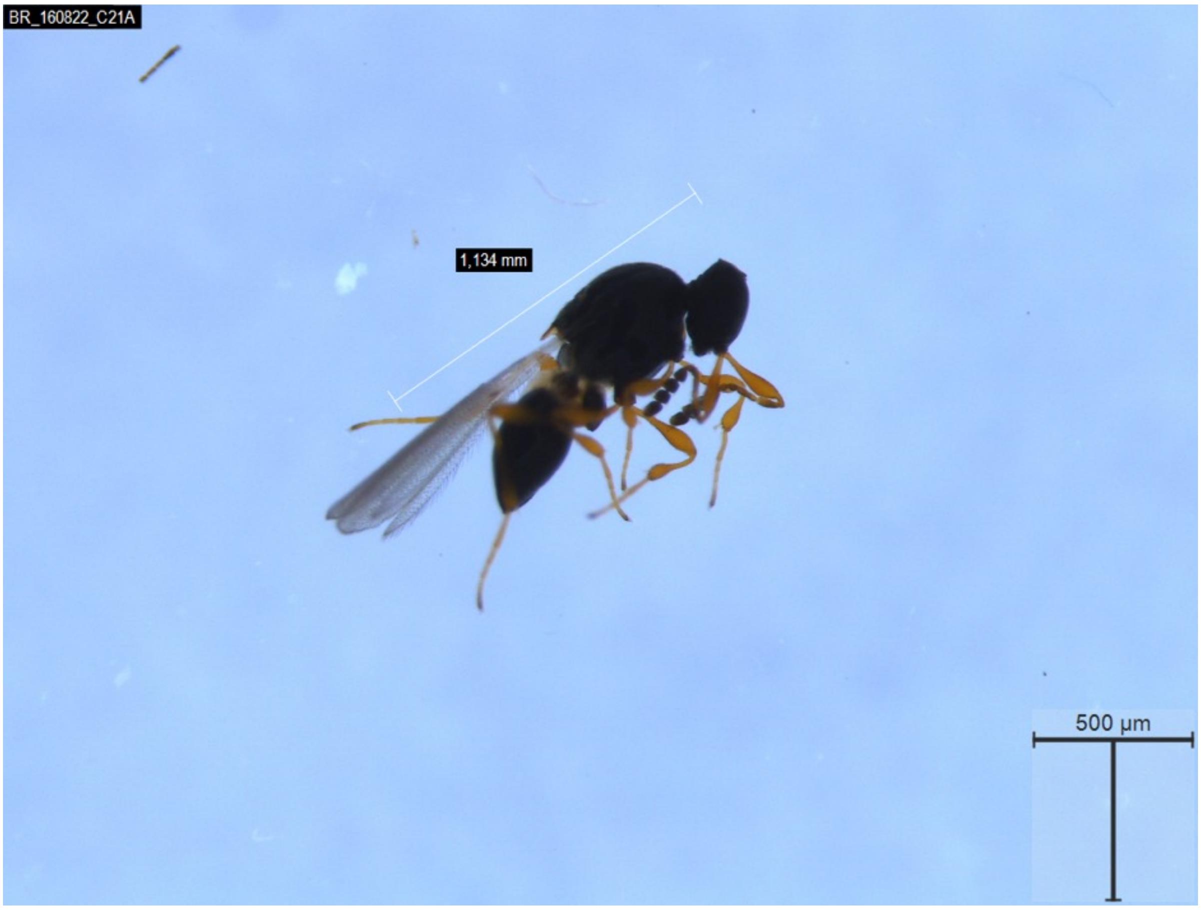

Figure S14: *Amblyaspis sp.*

Sanger-ID: SQ_2022_057_021
Data-ID: HA_310821_C5A (conventional HPS 2000K)

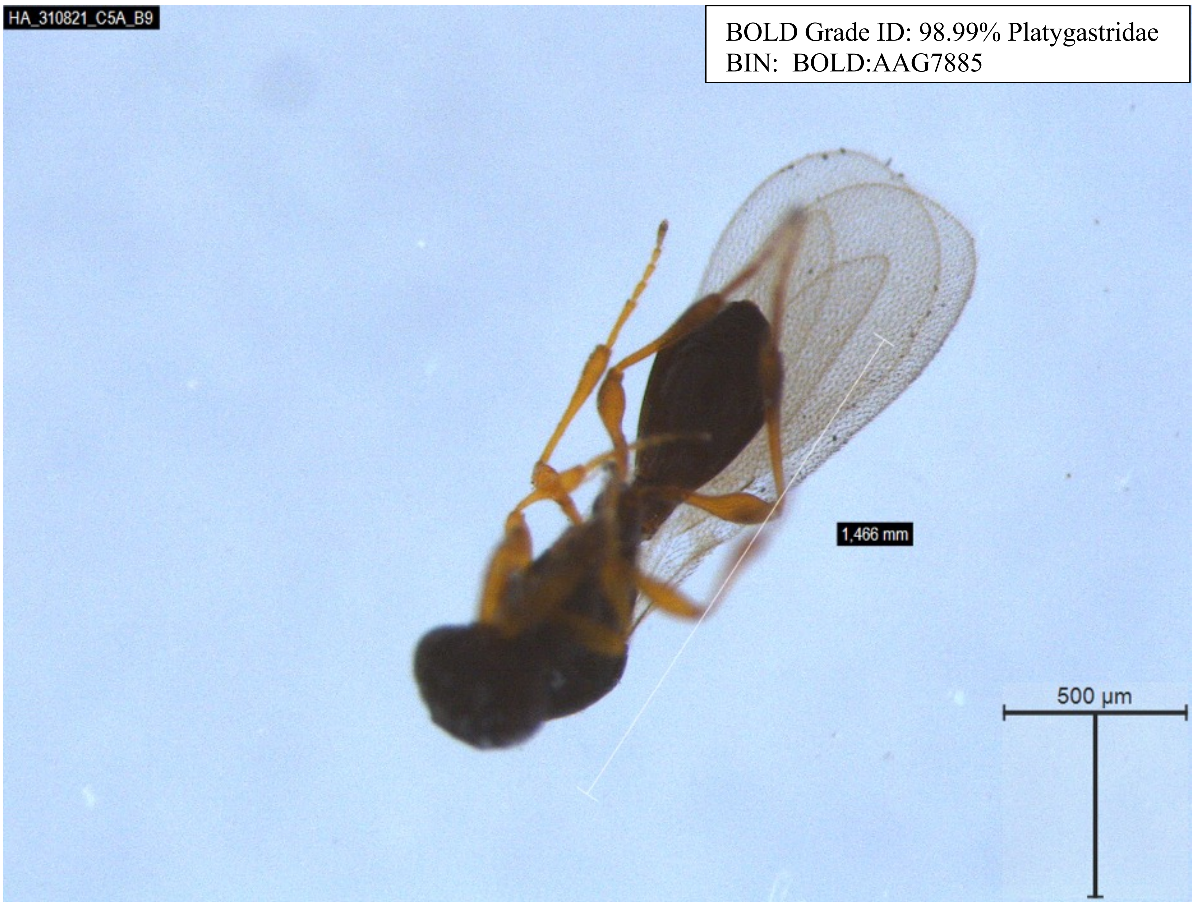

Figure S15: *Amblyaspis sp.*

Sanger-ID: SQ_2022_057_029
Data-ID: HA_240822_C5C (conventional HPS 2000K)

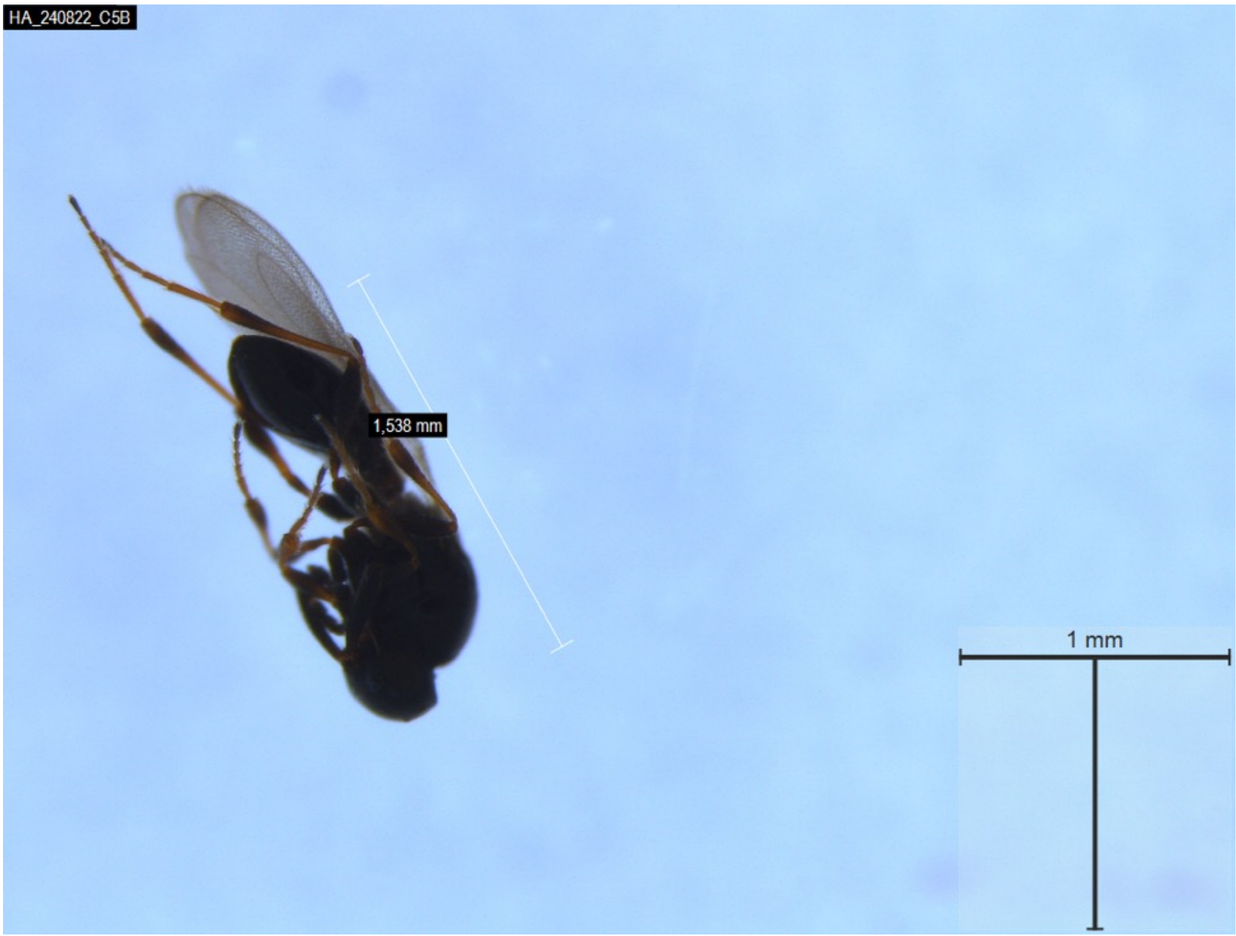

Figure S16: *Amblyaspis sp.*

Sanger-ID: SQ_2022_057_030
Data-ID: HA_240822_C7E (conventional HPS 2000K)

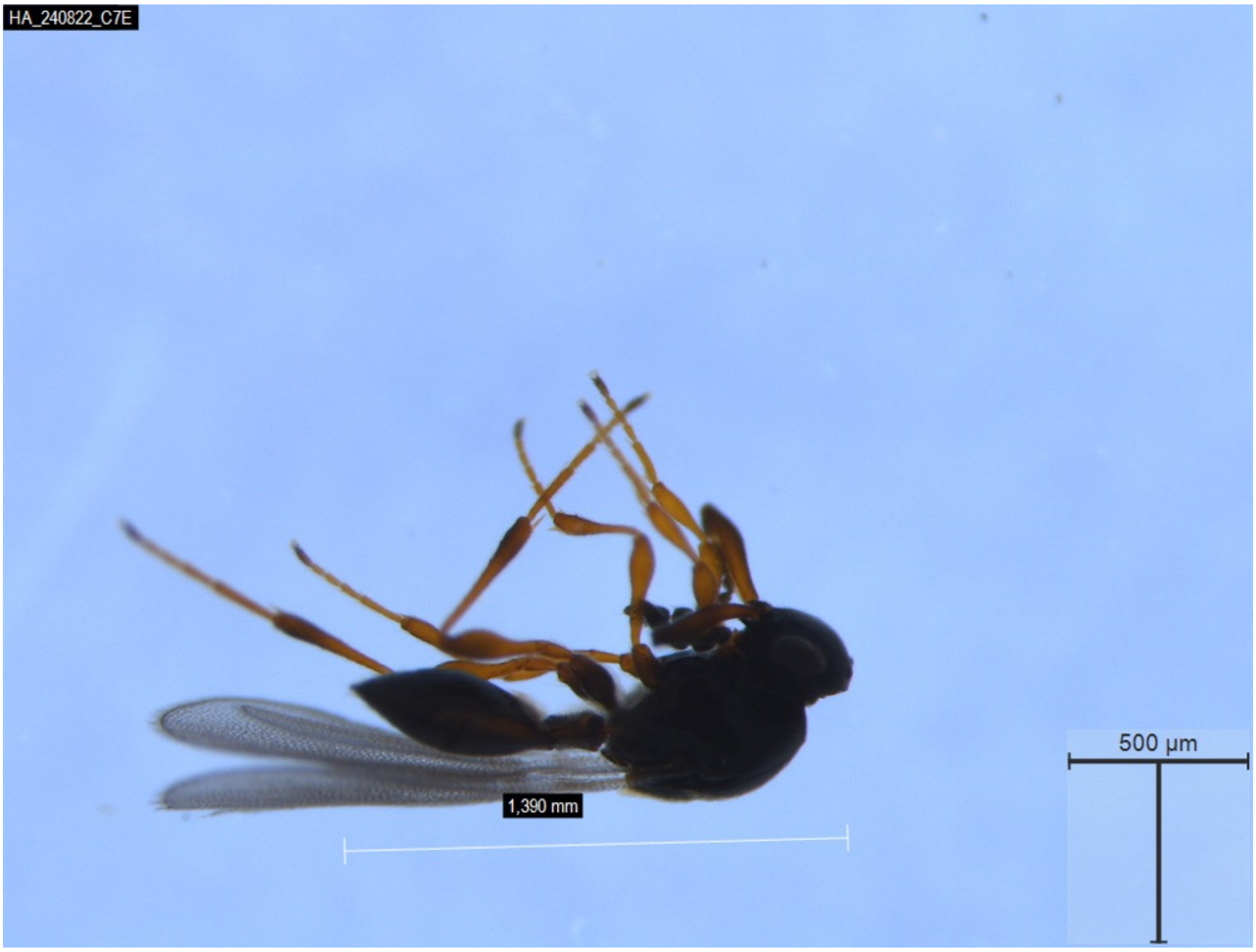

Figure S17: *Amblyaspis sp.*

Sanger-ID: SQ_2022_057_033
Data-ID: HA_120621_C1A (conventional HPS 2000K)

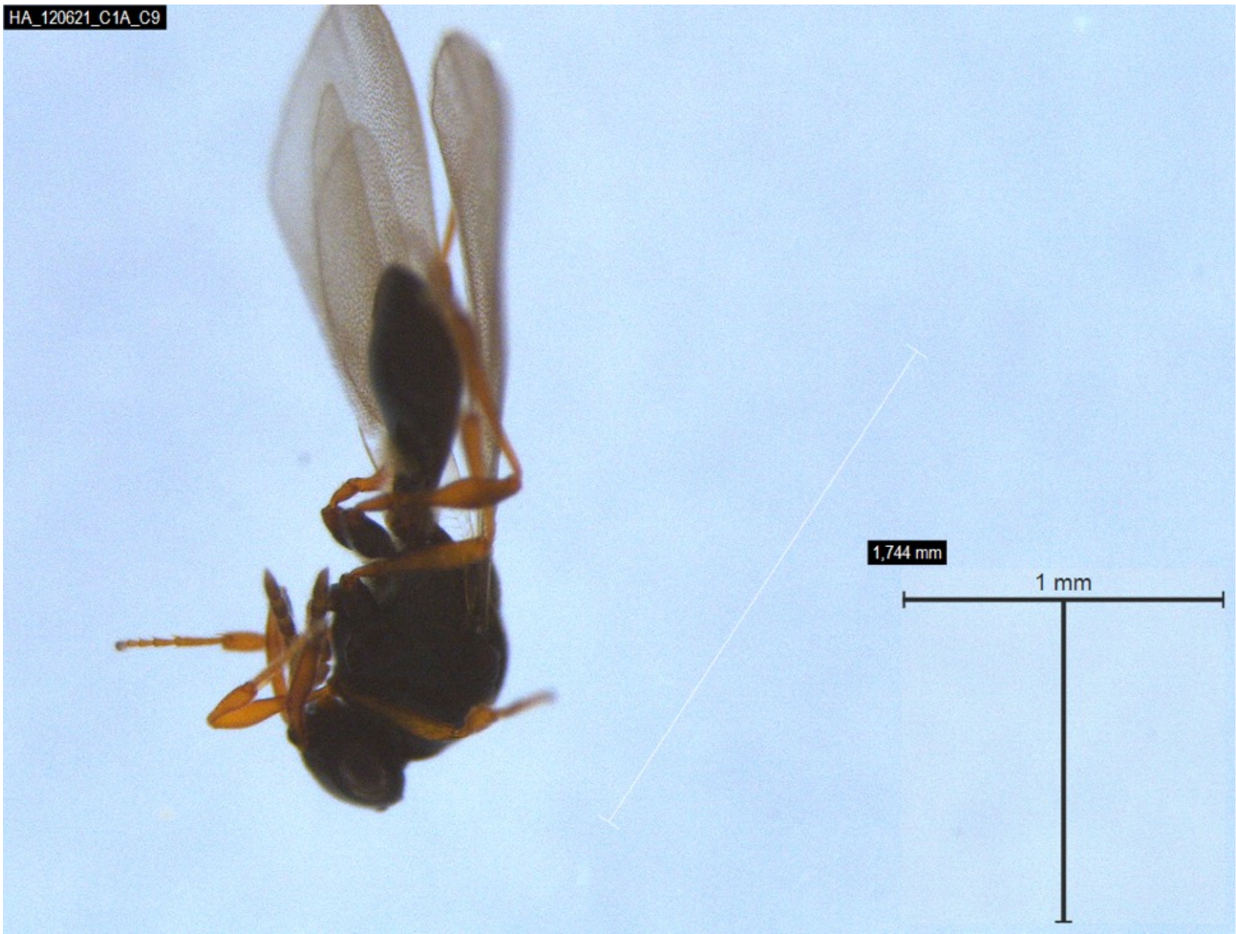

Figure S18: *Amblyaspis sp.*

Sanger-ID: SQ_2022_057_040
Data-ID: KA_310522_C17A (conventional LED 4000K)

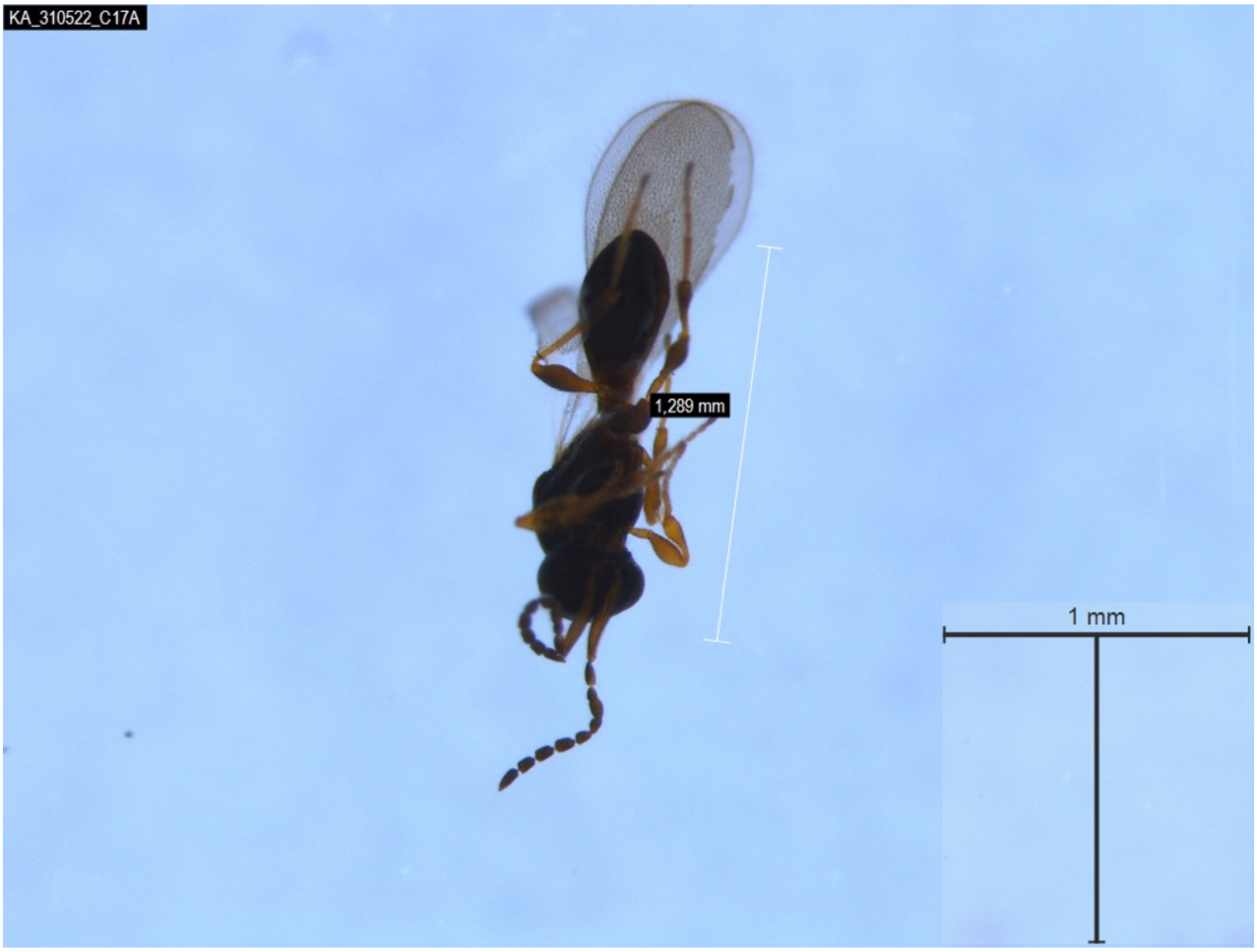

Figure S19: *Amblyaspis sp.*

Sanger-ID: SQ_2022_057_041
Data-ID: HA_240822_C6D (conventional HPS 2000K)

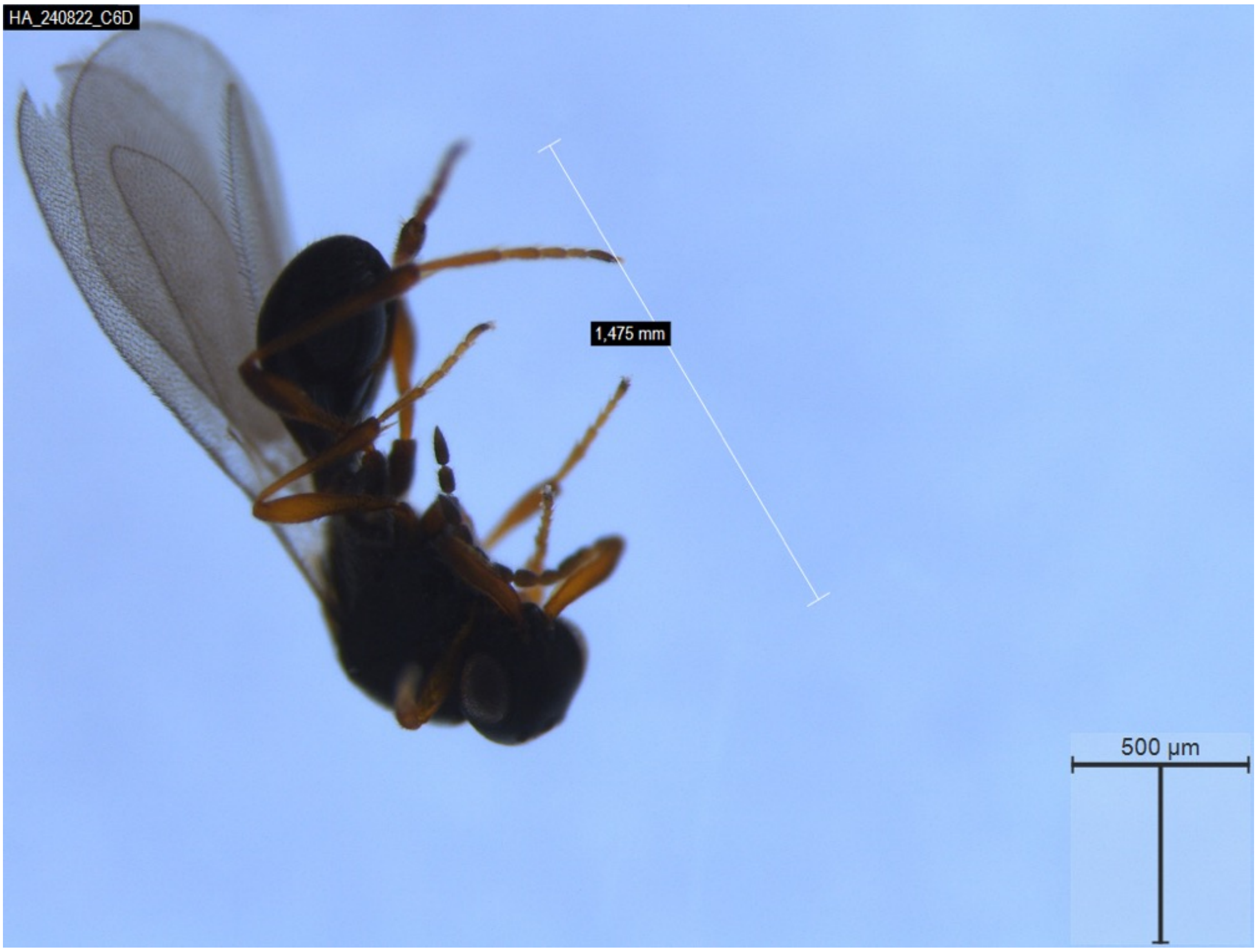

Figure S20: *Amblyaspis sp.*

Sanger-ID: SQ_2022_057_042
Data-ID: HA_240822_C7F (conventional HPS 2000K)

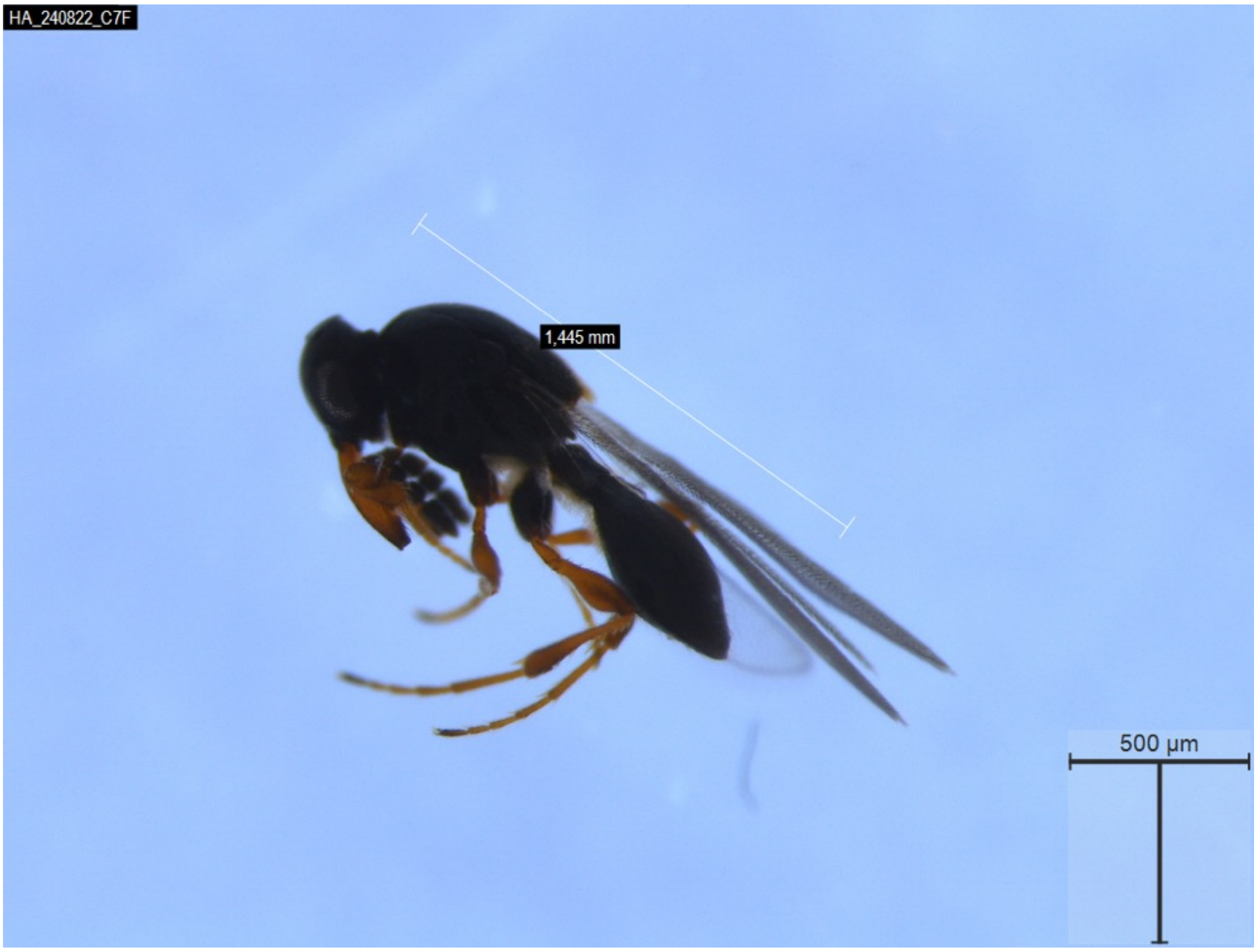

Figure S21 *Amblyaspis sp.*

Sanger-ID: SQ_2022_057_053
Data-ID: HA_240822_C7B (conventional HPS 2000K)

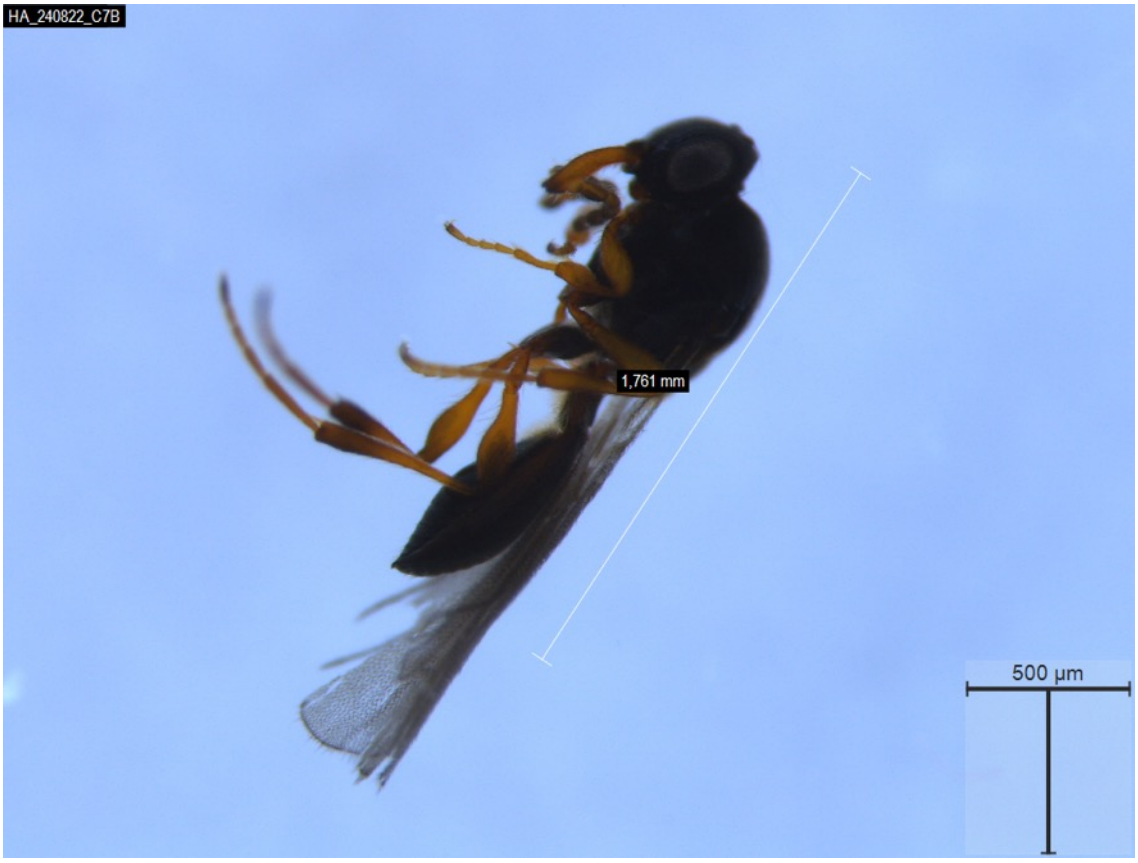

Figure S22: *Amblyaspis sp.*

Sanger-ID: SQ_2022_057_054
Data-ID: HA_240822_C7G (conventional HPS 2000K)

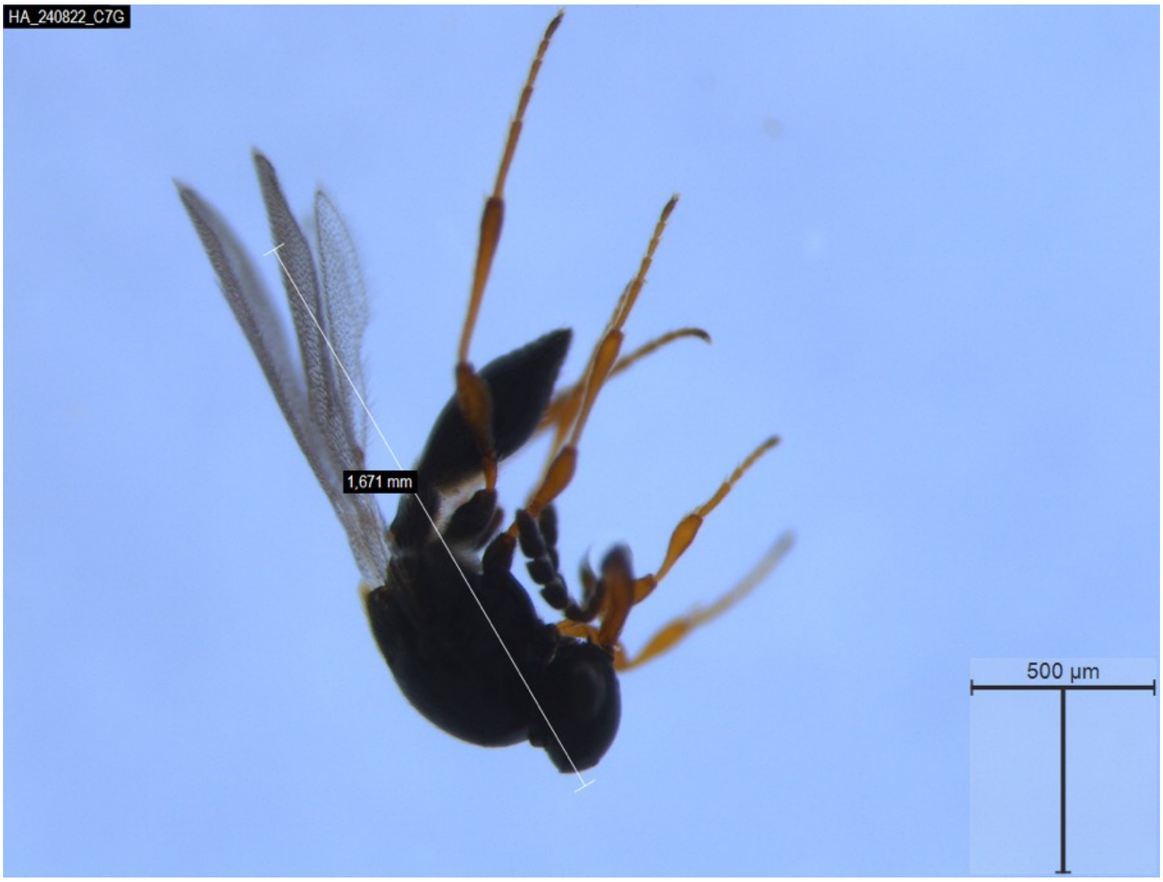

Figure S23: *Amblyaspis sp.*

Sanger-ID: SQ_2022_057_064
Data-ID: BR_300822_C28A (conventional HPS 2000K)

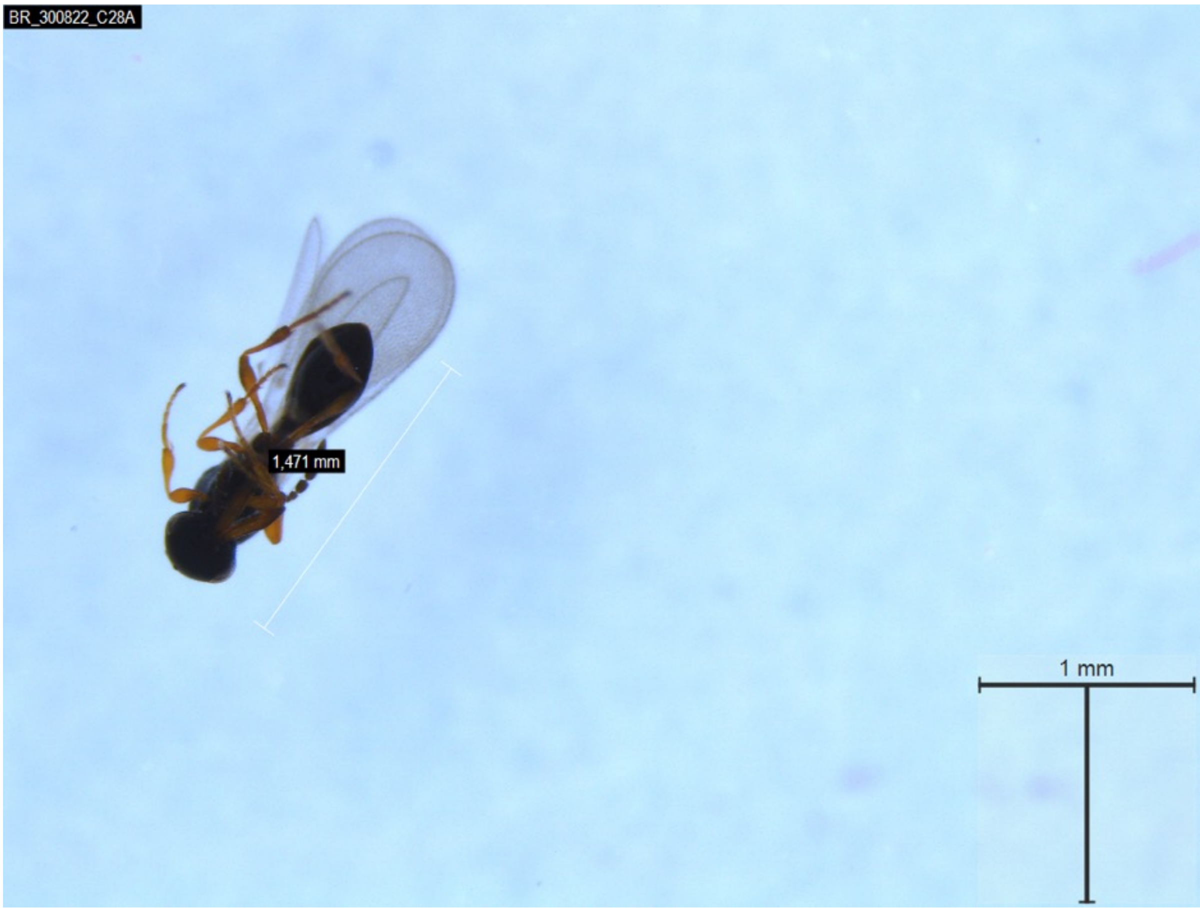

Figure S24 *Amblyaspis sp.*

Sanger-ID: SQ_2022_057_065
Data-ID: HA_240822_C7C (conventional HPS 2000K)

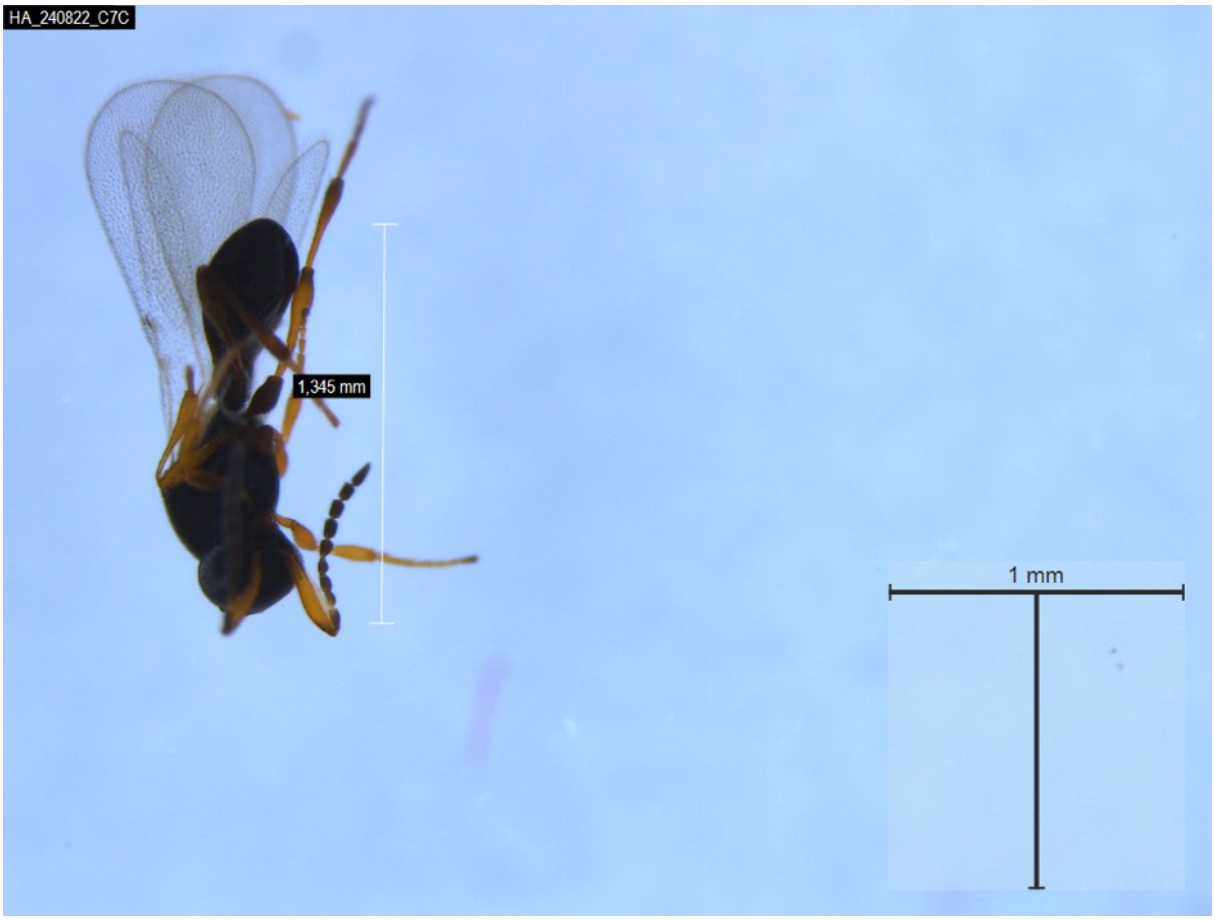

Figure S25: *Amblyaspis sp.*

Sanger-ID: SQ_2022_057_071
Data-ID: BR_020621_C22A (conventional HPS 2000K)

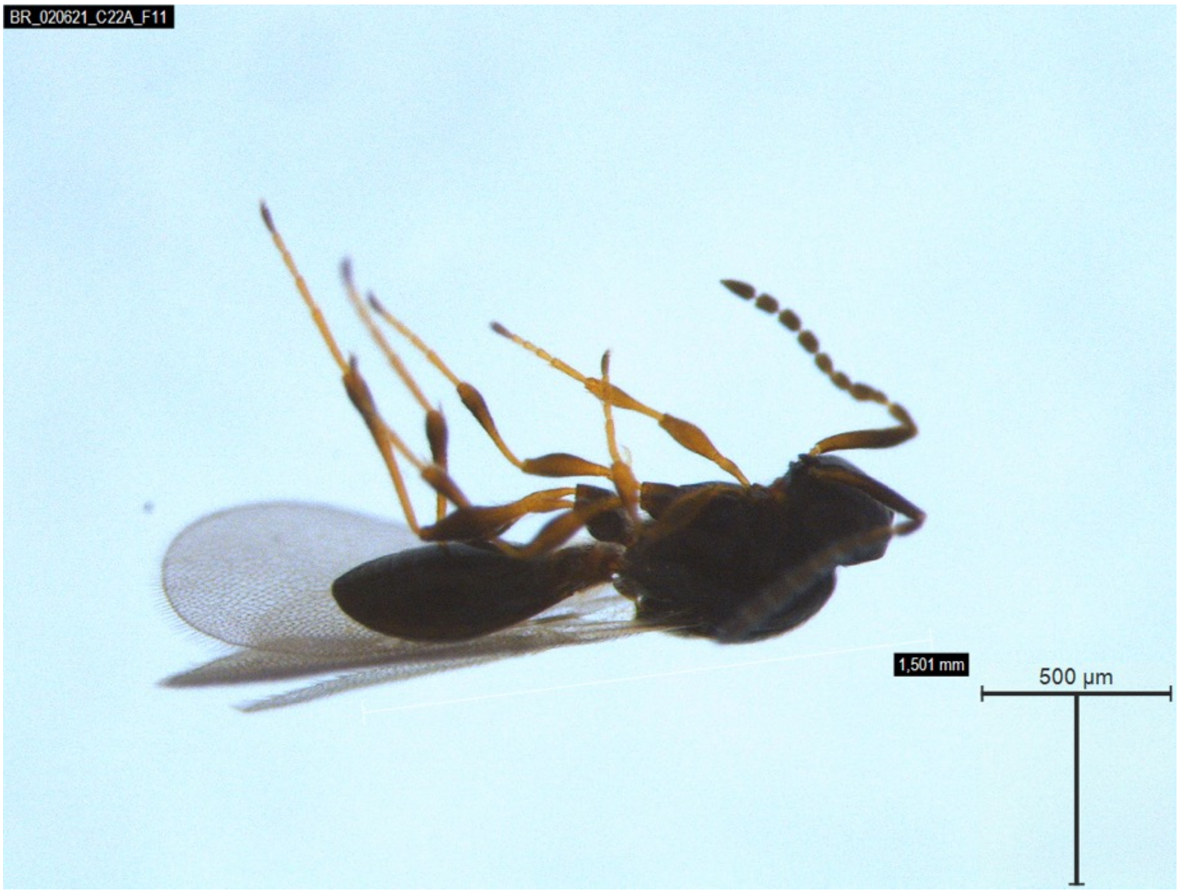

Figure S26: *Amblyaspis sp.*

Sanger-ID: SQ_2022_057_077
Data-ID: HA_240822_C7D (conventional HPS 2000K)

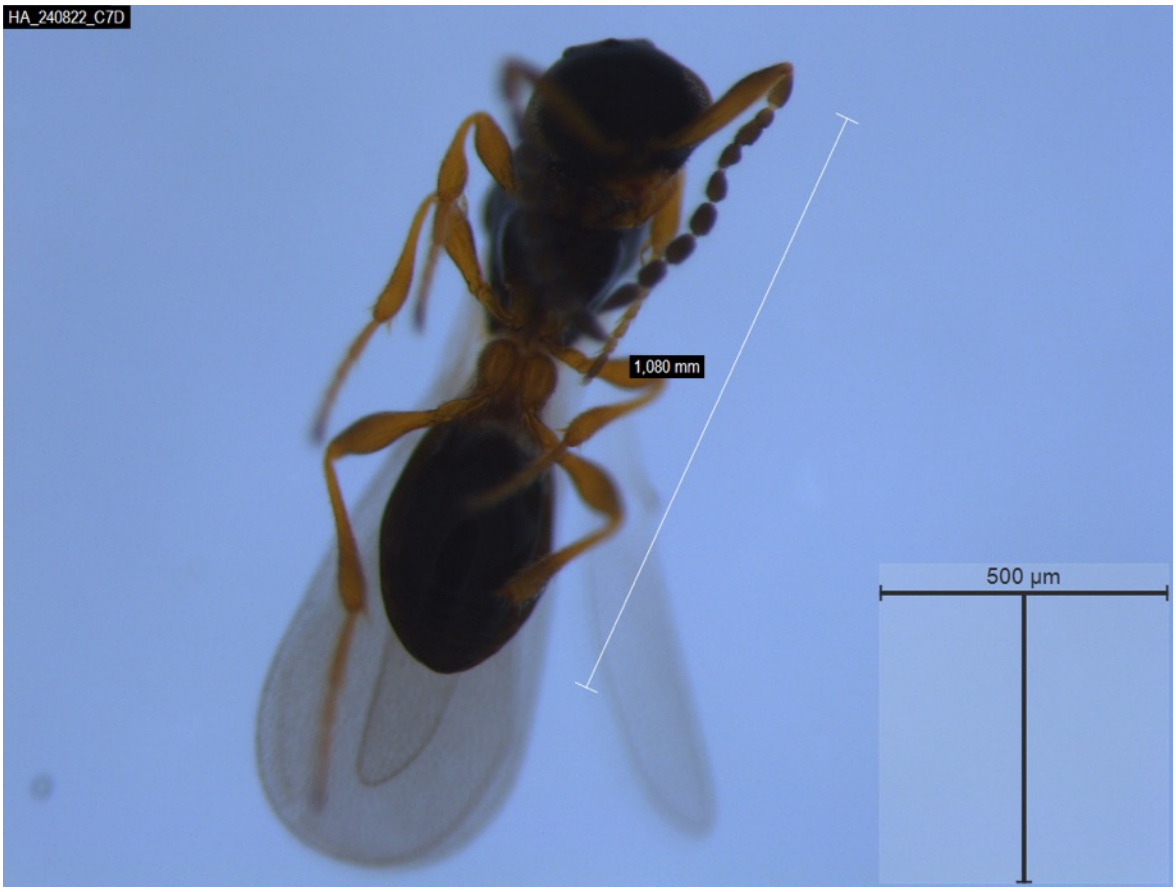

Figure S27: *Amblyaspis sp.*

Sanger-ID: SQ_2022_057_088
Data-ID: HA_050722_C5A (conventional HPS 2000K)

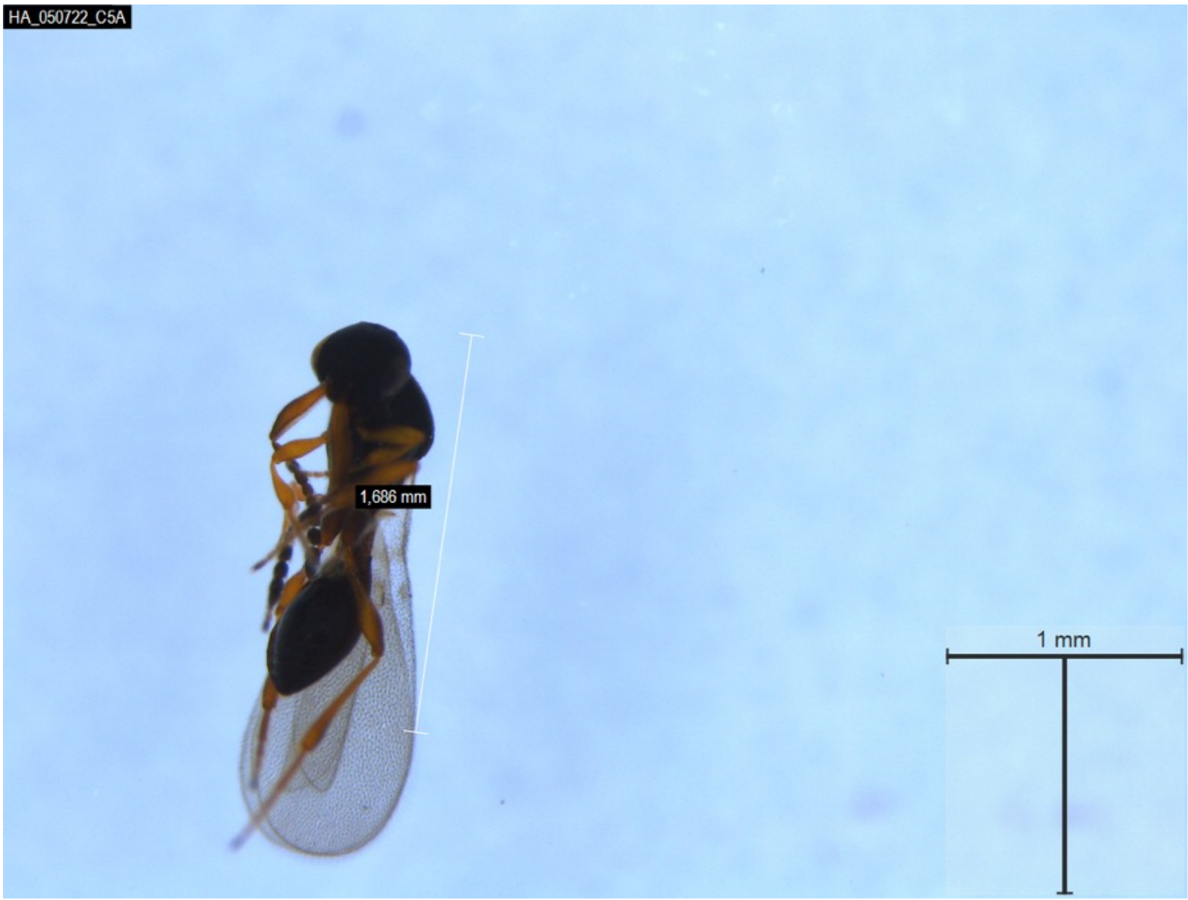

Figure S28: *Amblyaspis sp.*

Sanger-ID: SQ_2022_057_089
Data-ID: HA_240822_C5D (conventional HPS 2000K)

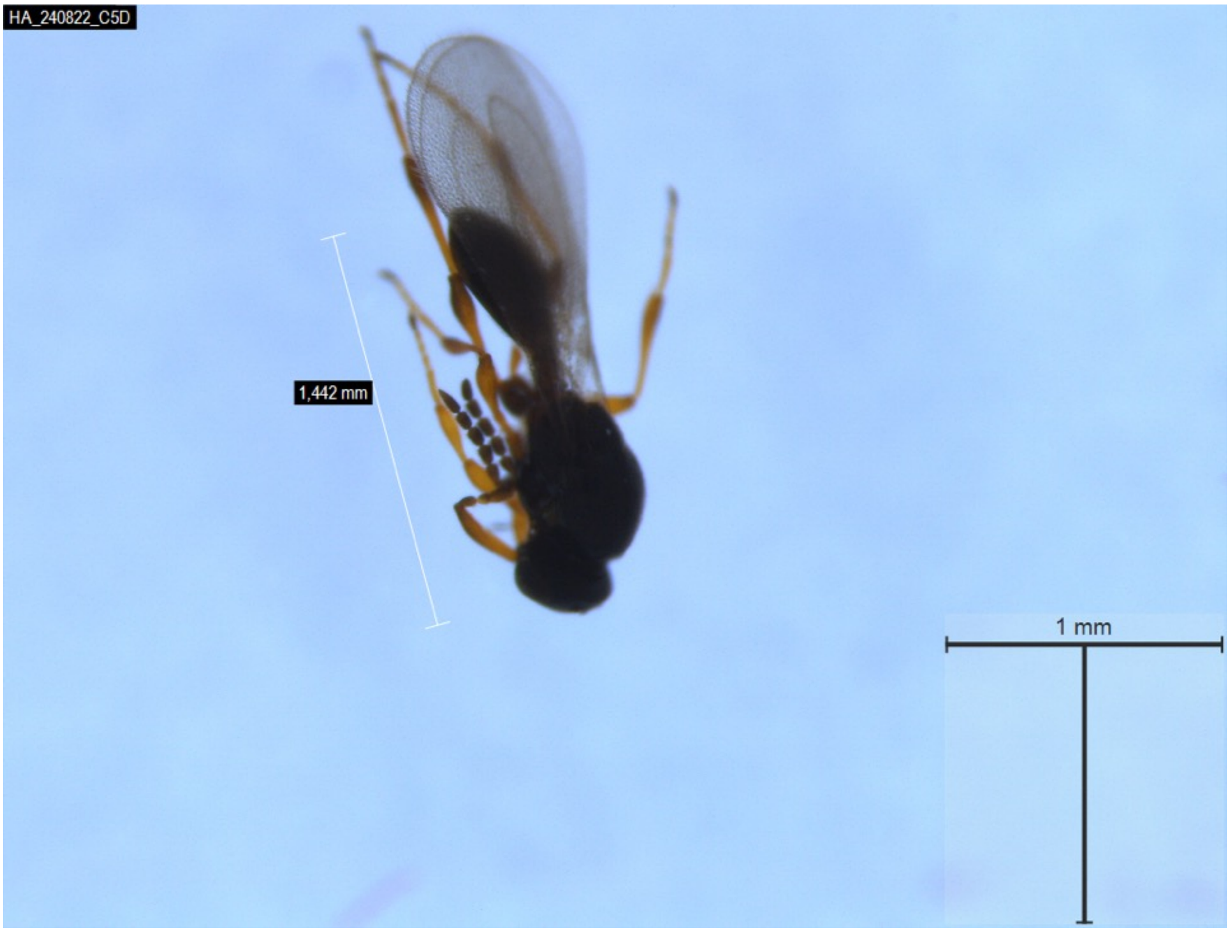

Figure S29 *Amblyaspis sp.*

Amblyteles armatorius (Ichneumonidae)

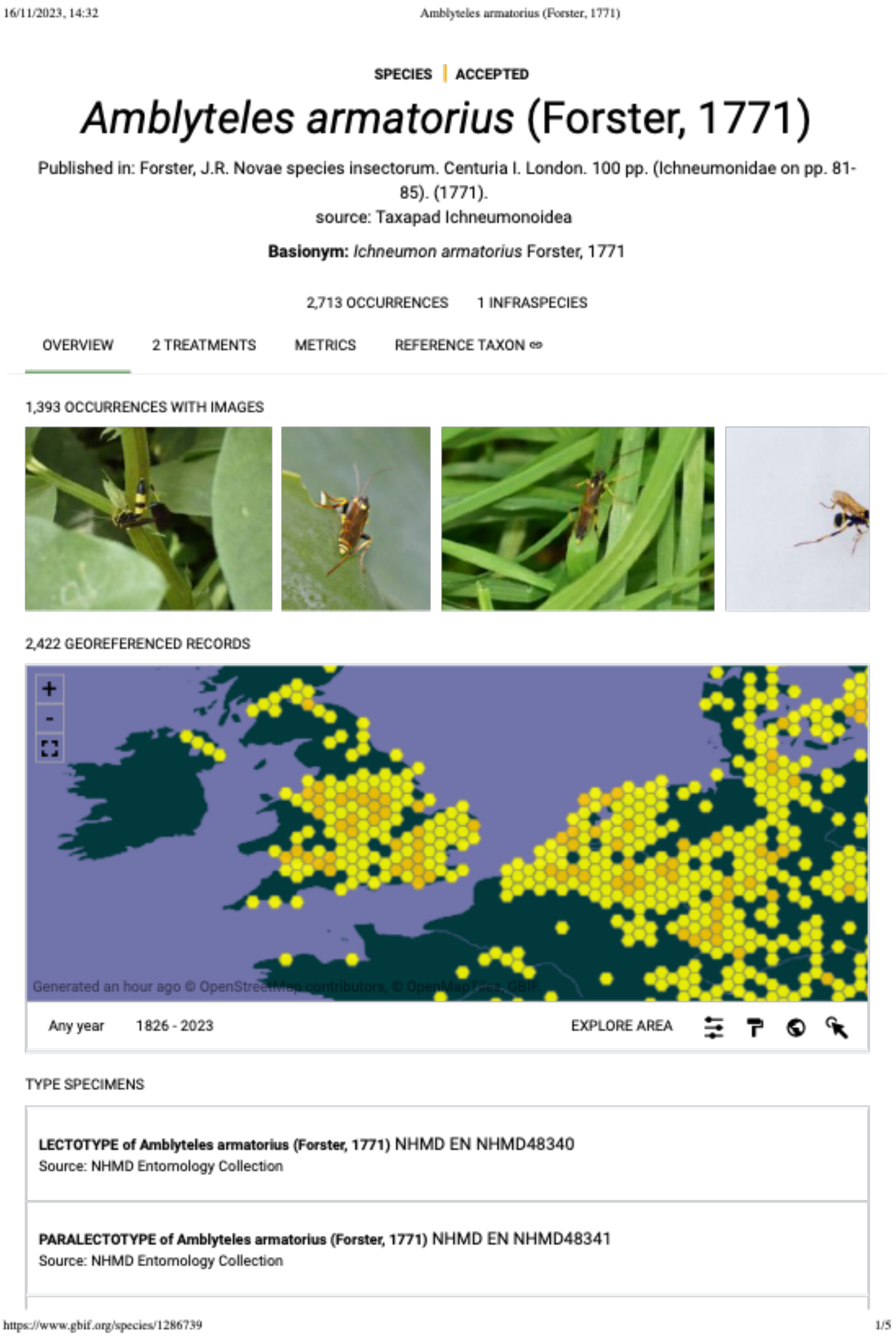

16/11/2023, 14:32 Amblyteles armatorius (Forster, 1771)

SPECIES | ACCEPTED

Amblyteles armatorius (Forster, 1771)

Published in: Forster, J.R. Novae species insectorum. Centuria I. London. 100 pp. (Ichneumonidae on pp. 81-85). (1771).
source: Taxapad Ichneumonoidea

Basionym: *Ichneumon armatorius* Forster, 1771

2,713 OCCURRENCES 1 INFRASPECIES

OVERVIEW 2 TREATMENTS METRICS REFERENCE TAXON

1,393 OCCURRENCES WITH IMAGES

2,422 GEOREFERENCED RECORDS

Generated an hour ago © OpenStreetMap contributors, © OpenMapTiles, GBIF.

Any year 1826 - 2023 EXPLORE AREA

TYPE SPECIMENS

LECTOTYPE of Amblyteles armatorius (Forster, 1771) NHMD EN NHMD48340
Source: NHMD Entomology Collection

PARALECTOTYPE of Amblyteles armatorius (Forster, 1771) NHMD EN NHMD48341
Source: NHMD Entomology Collection

https://www.gbif.org/species/1286739 1/5

Figure S30: Global Biodiversity Information Facility (GBIF) Webpage *Amblyteles armatorius*

Sanger_ID: SQ_2022_057_100
Data_ID: KA_171022_C15A (conventional LED 4000K)

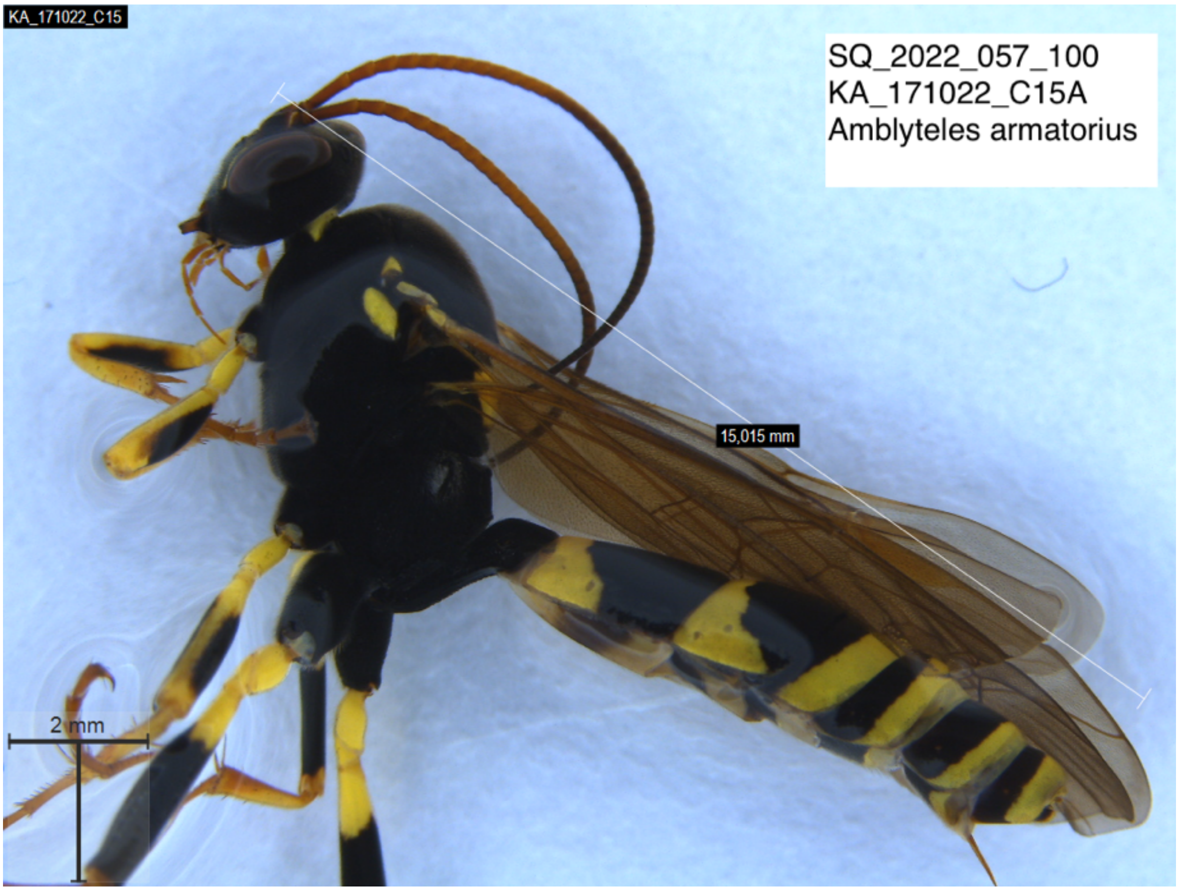

Figure S31 *Amblyteles armatorius*

Apanteles sodalis (Braconidae)

Sanger_ID: SQ_2022_057_023
Data_ID: BR_020921_C22A (conventional HPS 2000K)

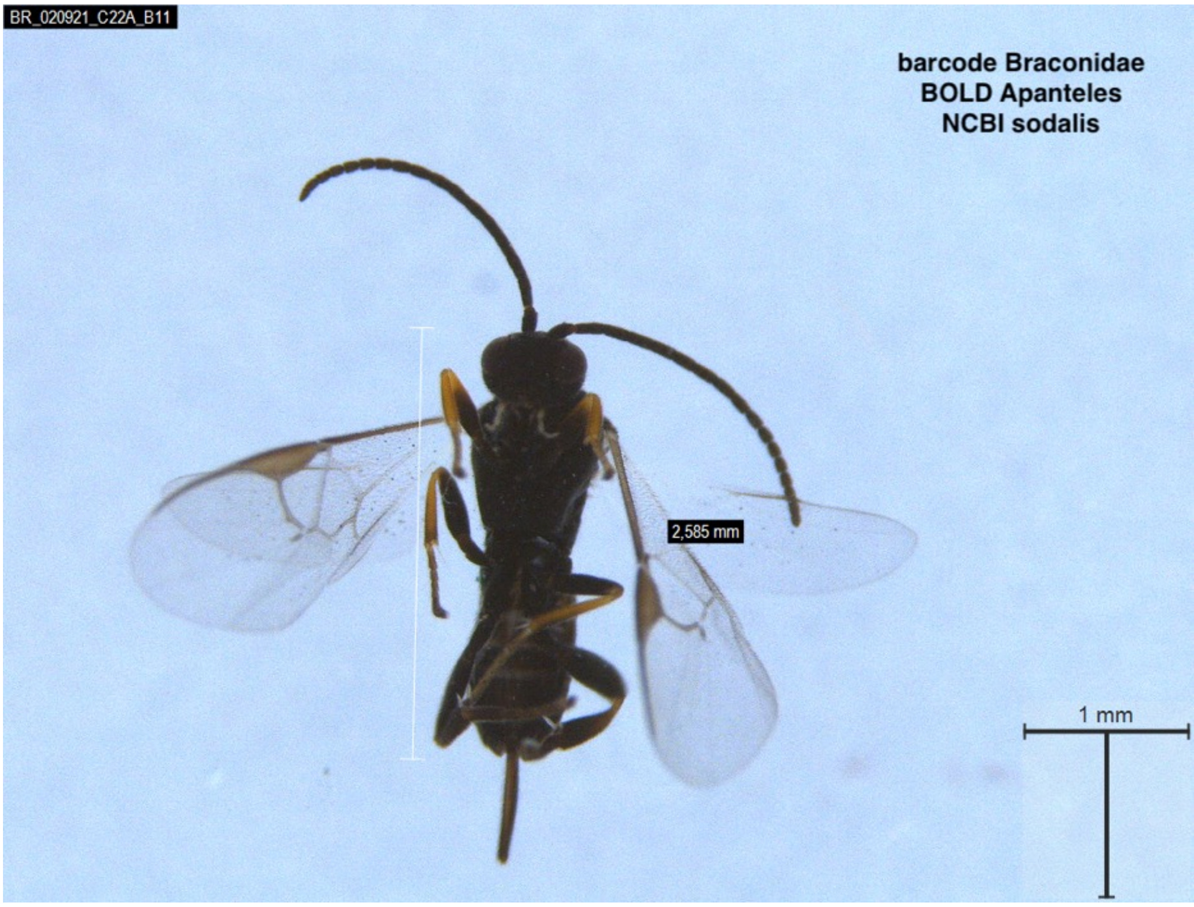

Figure S32: *Apanteles sodalis*

SPECIES | ACCEPTED

Apanteles sodalis (Haliday, 1834)

Published in: Haliday, A.H. Essay on parasitic Hymenoptera. Entomological Magazine. 2(iii):225-259. (1834).

source: Taxapad Ichneumonoidea

Braconid wasp In English **Basionym:** *Microgaster sodalis* Haliday, 1834

59 OCCURRENCES 4 INFRASPECIES

OVERVIEW 2 TREATMENTS METRICS REFERENCE TAXON

5 OCCURRENCES WITH IMAGES

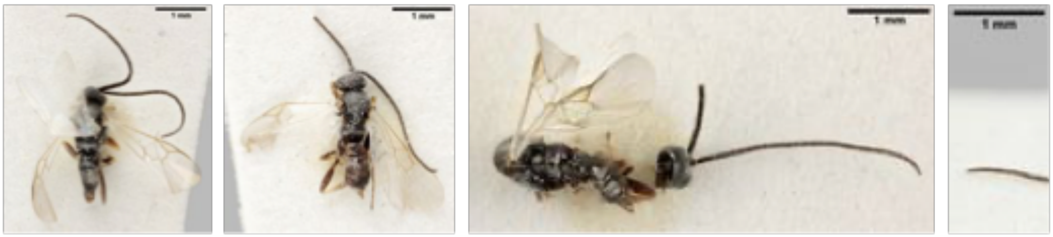

6 GEOREFERENCED RECORDS

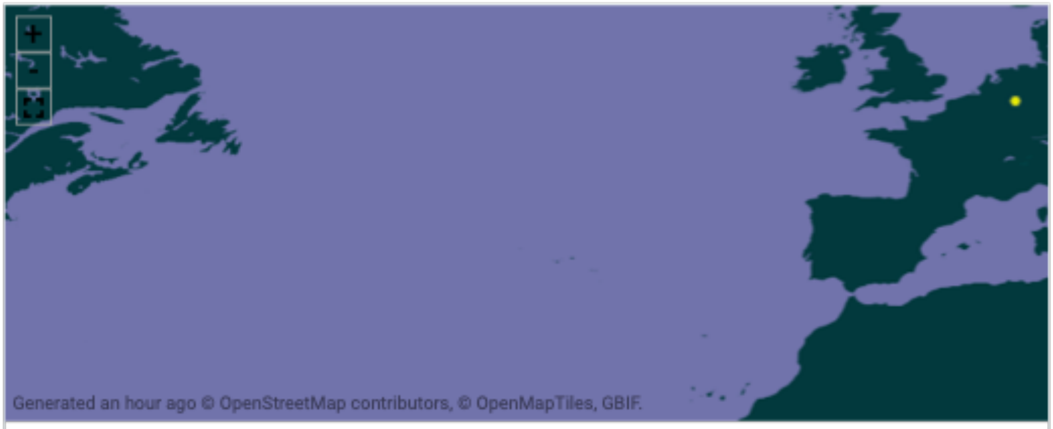

Any year 1942 - 1954 EXPLORE AREA

RECORDED AS INTRODUCED IN 1 COUNTRY OR ISLAND

Recorded as introduced in	According to	Evidence of impact	Occurrences in GBIF
United States of America	Global Register of Introduced and Invasive Species - United States (Contiguous) (ver.2.0, 2022)	No	Unknown

TYPE SPECIMENS

Figure S33: Global Biodiversity Information Facility (GBIF) Webpage *Apanteles sodalis*

Aphidius rhopalosiphi (Braconidae)

Sanger_ID: SQ_2022_057_048
Data_ID: BR_280622_C26A (conventional HPS 2000K)

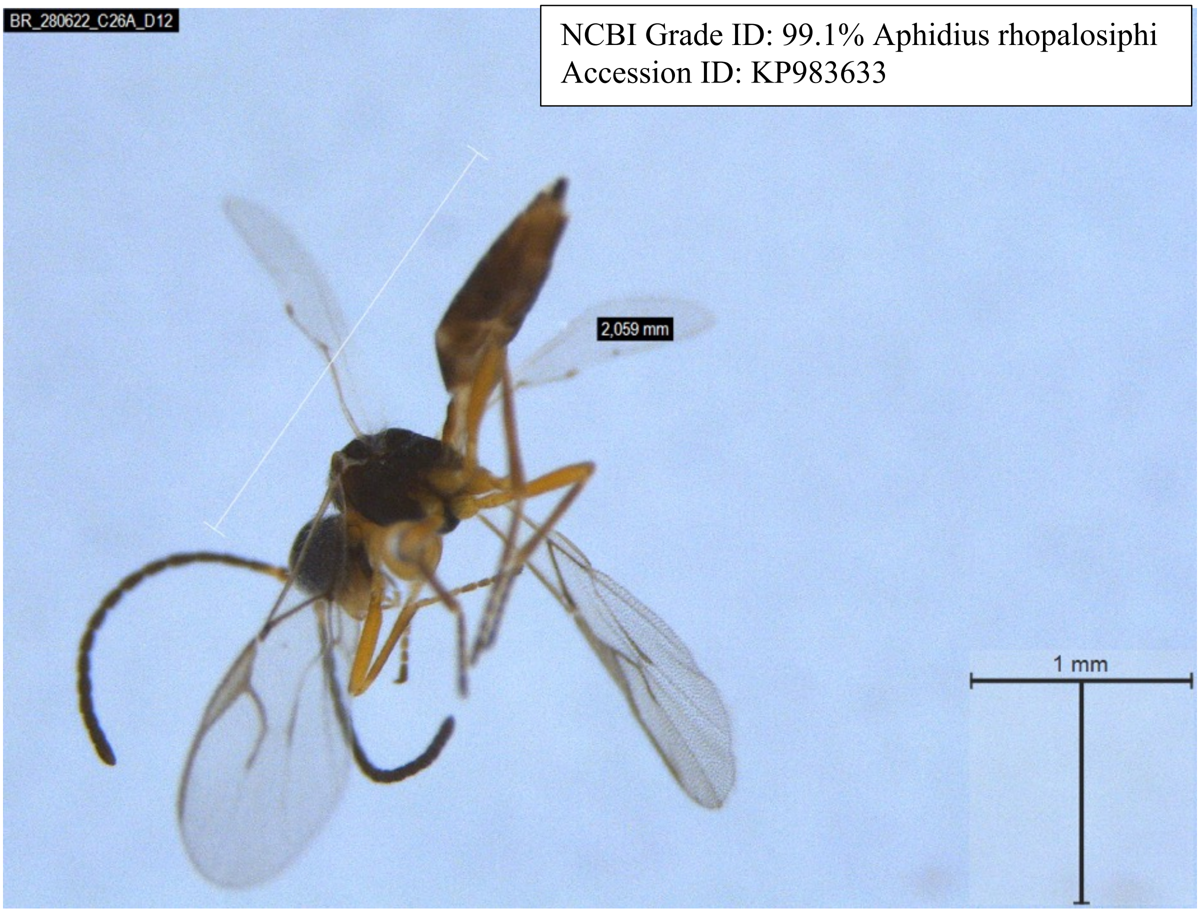

Figure S34: *Aphidius rhopalosiphi*

SPECIES | ACCEPTED

Aphidius rhopalosiphi de Stefani-Perez, 1902

Published in: De Stefani-Perez, T. Osservazioni biologische sopra un Braconide aquatico, Giardinaia urinator, a descrizione di due altri Imenotteri nuovi. Zoologische Jahrbucher Abteilung fur Systematik. 15:625-634. (1902).
source: Taxapad Ichneumonoidea

916 OCCURRENCES 1 INFRASPECIES

OVERVIEW 4 TREATMENTS METRICS REFERENCE TAXON

2 OCCURRENCES WITH IMAGES

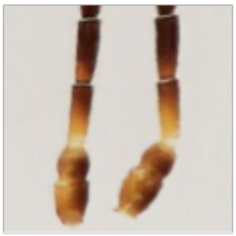

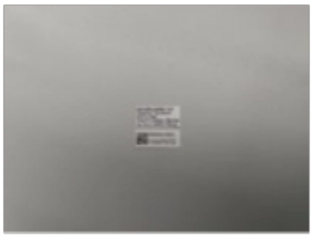

211 GEOREFERENCED RECORDS

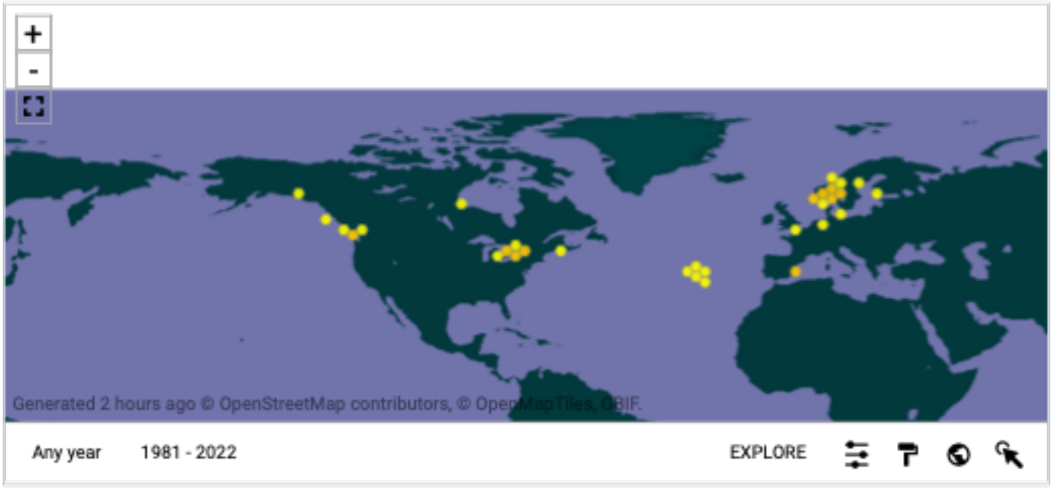

NAME USAGES APPLIED TO OCCURRENCES IN GBIF

Figure S35: Global Biodiversity Information Facility (GBIF) Webpage *Aphidius rhopalosiphi*

Aprostocetus sp. (Eulophidae)

Sanger_ID: SQ_2022_057_085
Data_ID: BR_160822_C27B (conventional HPS 2000K)

No photo available

BOLD Grade ID: 96.99% Aprostocetus<br>BIN: BOLD:ADS2460

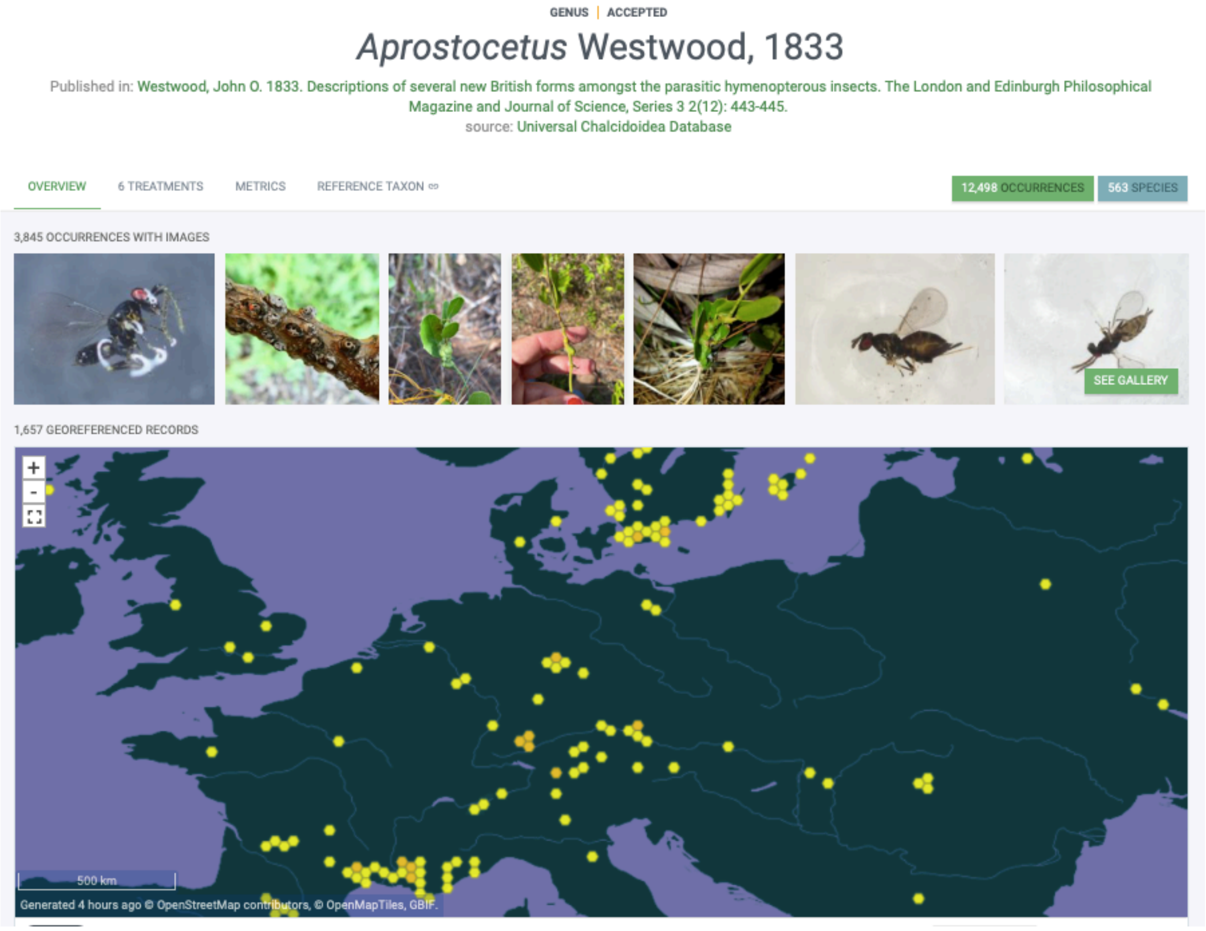

Figure S36: Global Biodiversity Information Facility (GBIF) Webpage *Aprostocetus* 18.12.23

Aptesis assimilis (Ichneumonidae)

Sanger_ID: SQ_2022_057_081
Data_ID: BR_020921_C27A (conventional HPS 2000K)

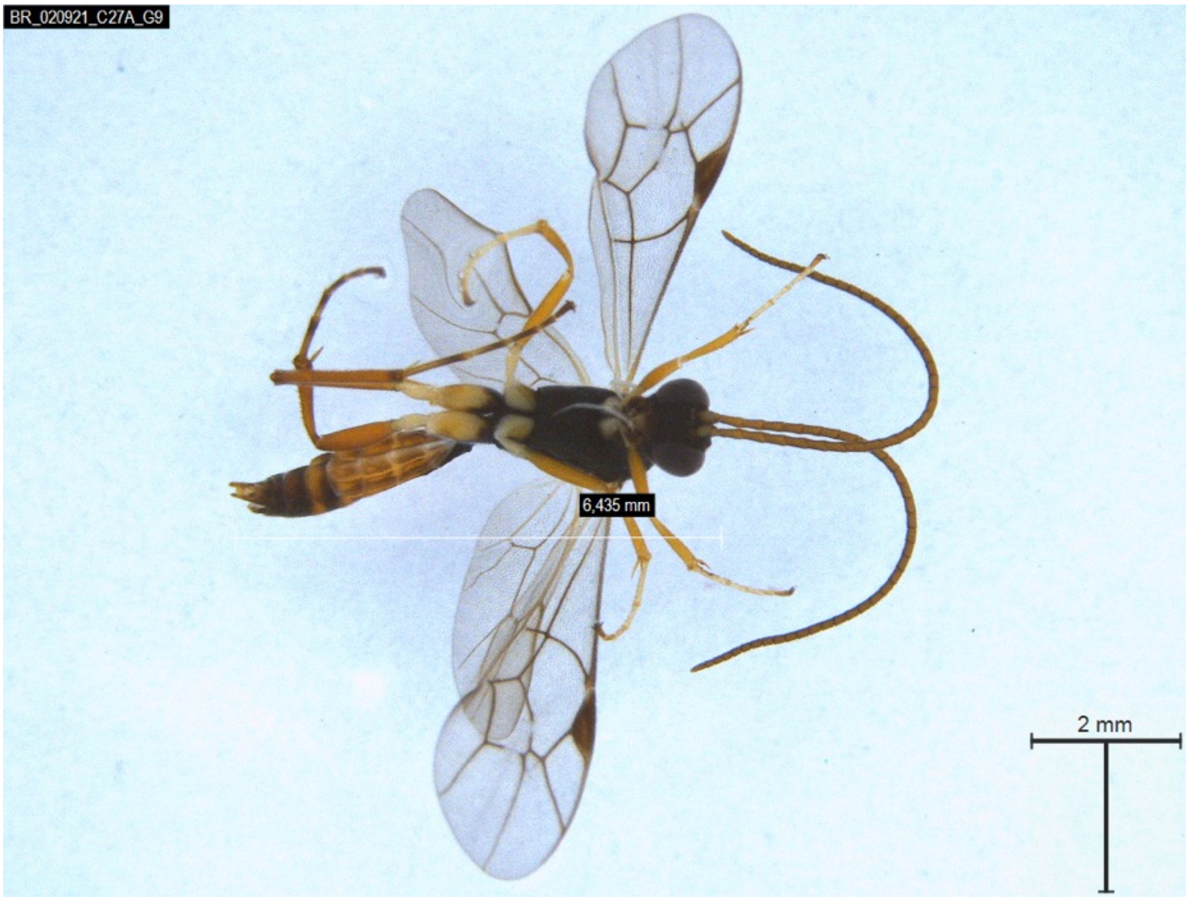

Figure S37 *Aptesis Assimilis*

SPECIES | ACCEPTED

Aptesis assimilis (Gravenhorst, 1829)

Published in: Gravenhorst, J.L.C. Ichneumonologia Europaea. Pars II. Vratislaviae. 989 pp. (1829).
source: Taxapad Ichneumonoidea

Basionym: *Phygadeuon assimilis* Gravenhorst, 1829

49 OCCURRENCES 1 INFRASPECIES

OVERVIEW 1 TREATMENT METRICS REFERENCE TAXON

27 GEOREFERENCED RECORDS

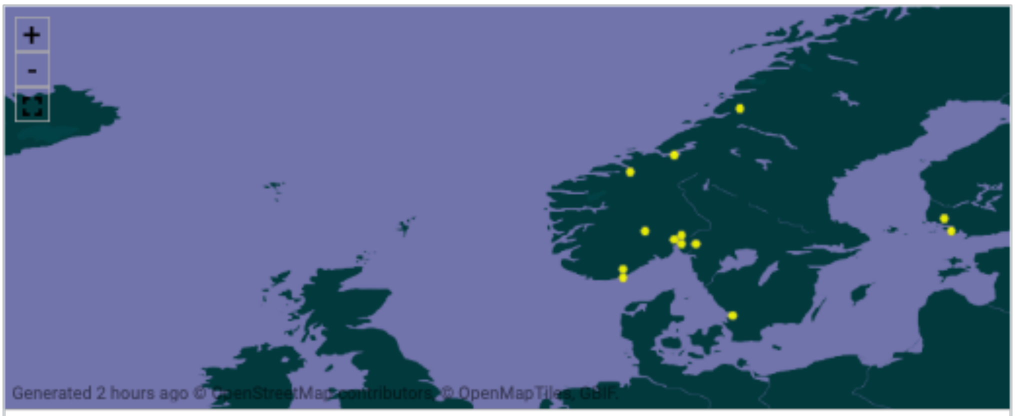

Any year 1909 - 2017 EXPLORE AREA

NAME USAGES APPLIED TO OCCURRENCES IN GBIF

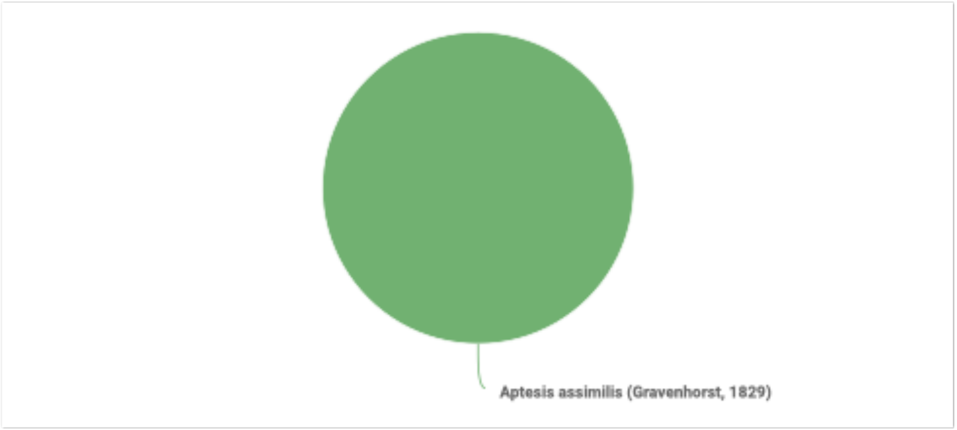

DESCRIPTION

DESCRIPTION

Figure S38: Global Biodiversity Information Facility (GBIF) Webpage *Aptesis assimilis*

Asobara rufescens (Braconidae)

Sanger_ID: SQ_2022_057_047
Data_ID: BR_020921_C24A (conventional HPS 2000K)

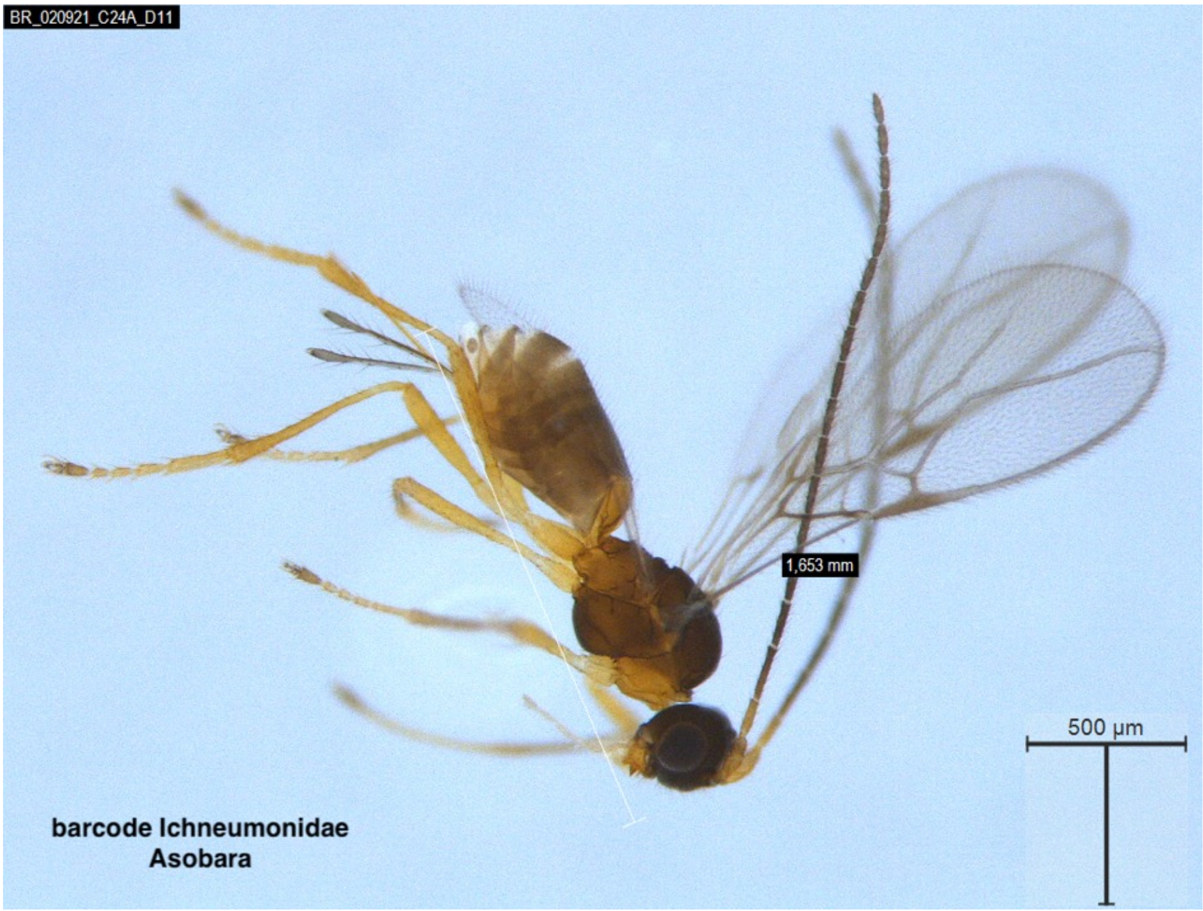

Figure S39: *Asobara rufescens*

SPECIES | ACCEPTED

Asobara rufescens (Forster, 1862)

Published in: Forster, A. Synopsis der Familien und Gattungen der Braconiden. Verhandlungen des Naturhistorischen Vereins der Preussischen Rheinlande und Westfalens. 19:225-288. (1862).
source: Taxapad Ichneumonoidea

Basionym: *Spanista rufescens* Foerster, 1862

158 OCCURRENCES 2 INFRASPECIES

OVERVIEW 2 TREATMENTS METRICS REFERENCE TAXON

2 OCCURRENCES WITH IMAGES

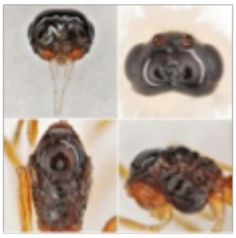

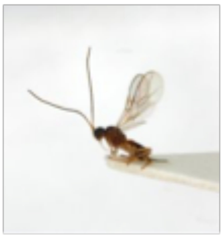

81 GEOREFERENCED RECORDS

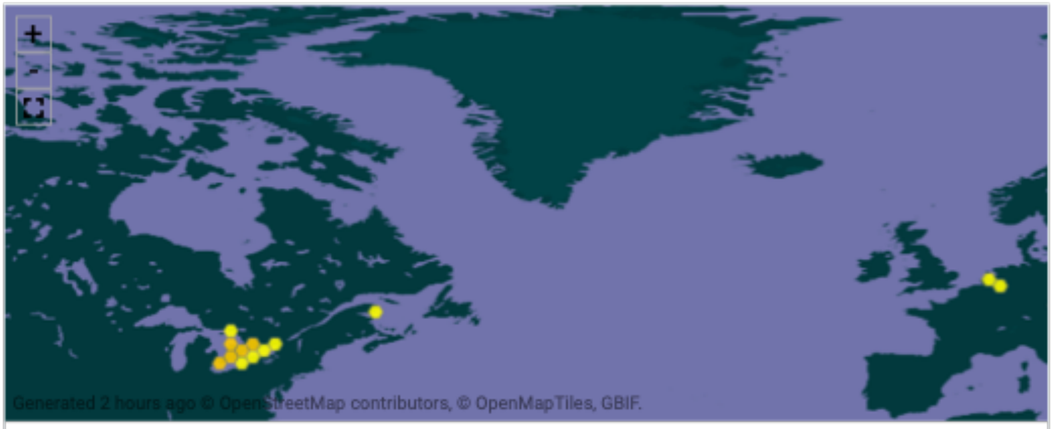

Any year 2008 - 2021 EXPLORE

NAME USAGES APPLIED TO OCCURRENCES IN GBIF

Figure S40: Global Biodiversity Information Facility (GBIF) Webpage *Asobara rufescens*

Barycnemis angustipennis (Ichneumonidae)

Sanger_ID: SQ_2022_057_104
Data_ID: HA_310822_C8A (conventional HPS 2000K)

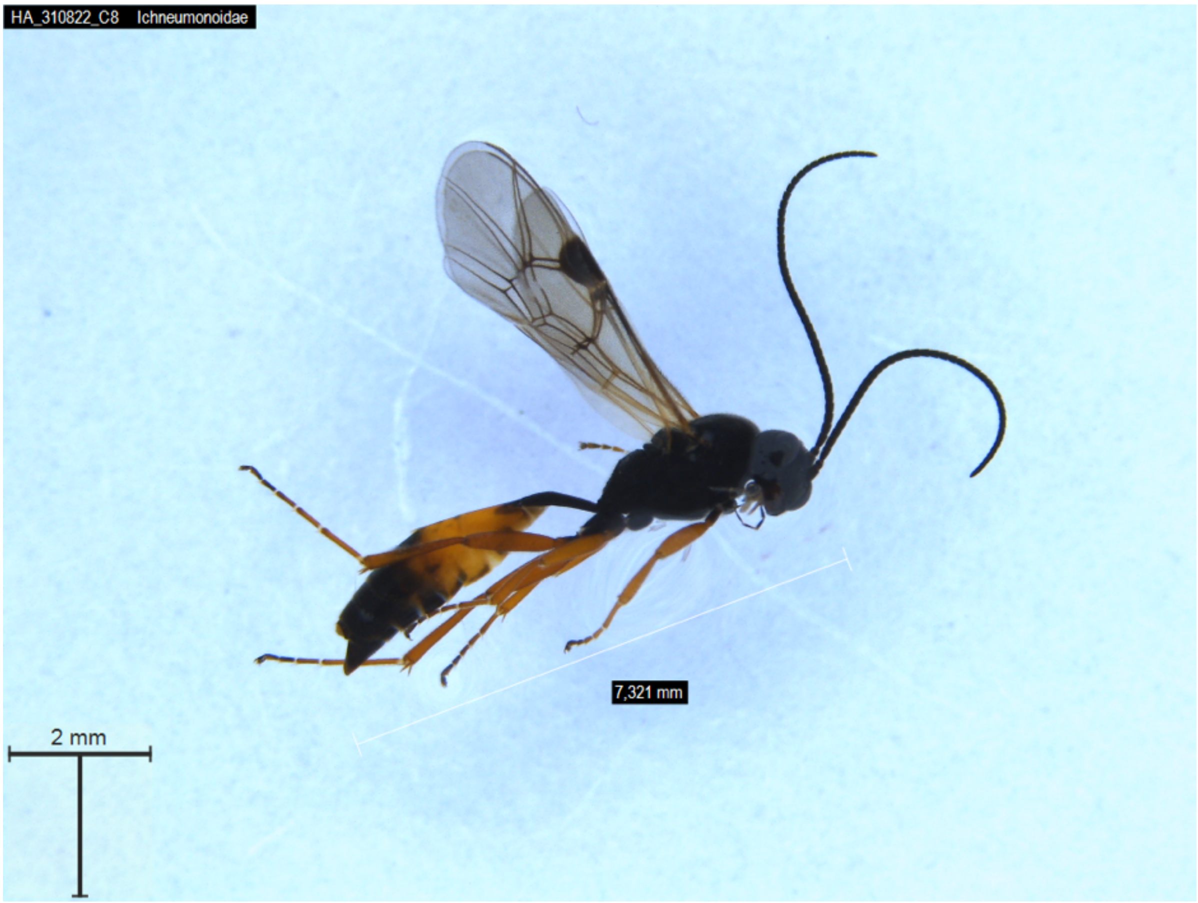

Figure S41: Barycnemis angustipennis

SPECIES | ACCEPTED

Barycnemis angustipennis (Holmgren, 1860)

Published in: Holmgren, A.E. Forsok till uppstallning och beskrifning af de i Sverige funna Ophionider. (Monographia Ophionidum Sueciae). Kongliga Svenska Vetenskapsakademiens Handlingar. 2(8):1-158. (1860).
source: Taxapad Ichneumonoidea

Basionym: *Porizon angustipennis* Holmgren, 1860

59 OCCURRENCES 1 INFRASPECIES

OVERVIEW 1 TREATMENT METRICS REFERENCE TAXON

15 GEOREFERENCED RECORDS

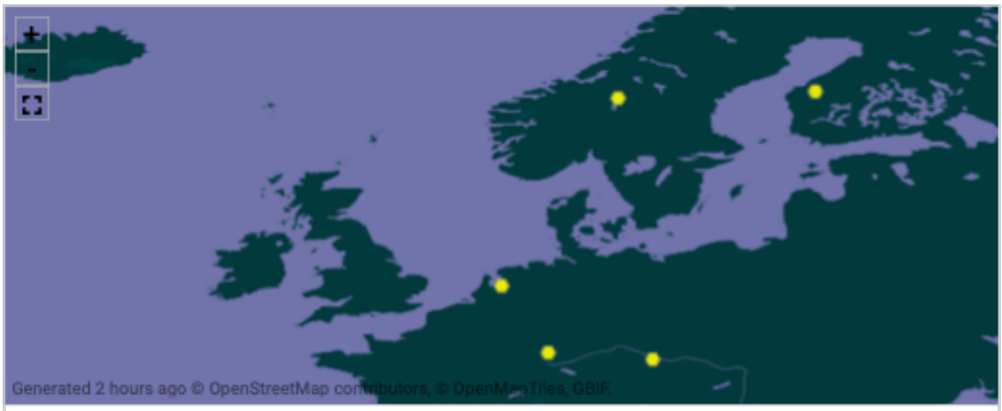

Any year 1934 - 2011 EXPLORE AREA

TYPE SPECIMENS

LECTOTYPE of Barycnemis angustipennis (Holmgren, 1860) Carl Henrik Boheman Sweden. NRM NHRS NHRS-HEVA000012175
Source: Entomological Collections (NHRS), Swedish Museum of Natural History (NRM)

NAME USAGES APPLIED TO OCCURRENCES IN GBIF

Figure S42: Global Biodiversity Information Facility (GBIF) Webpage *Barycnemis angustipennis*

Braconidae sp. (Braconidae)

Sanger_ID: SQ_2022_057_020
Data_ID: HA_260722_C5A (conventional HPS 2000K)

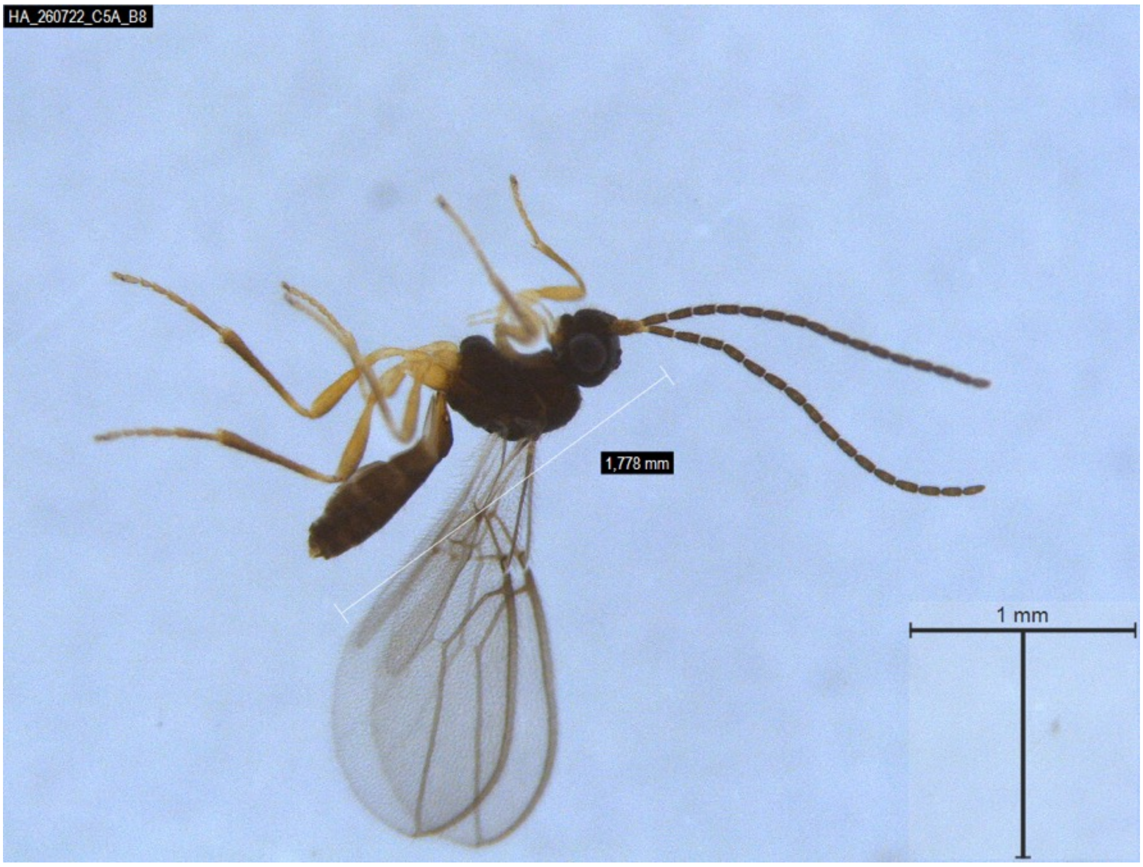

Figure S43: *Braconidae sp.*

Blacus ruficornis (Braconidae)

16/11/2023, 15:37 Blacus ruficornis (Nees, 1811)

SPECIES | ACCEPTED

Blacus ruficornis (Nees, 1811)

Published in: Nees von Esenbeck, C.G. Ichneumonides Adsciti, in Genera et Familias Divisi. Magazin Gesellschaft Naturforschender Freunde zu Berlin. 5(1811). 37 pp. (1811).
source: Taxapad Ichneumonoidea

Basionym: *Bracon ruficornis* Nees, 1811

77 OCCURRENCES 3 INFRASPECIES

OVERVIEW METRICS REFERENCE TAXON

8 OCCURRENCES WITH IMAGES

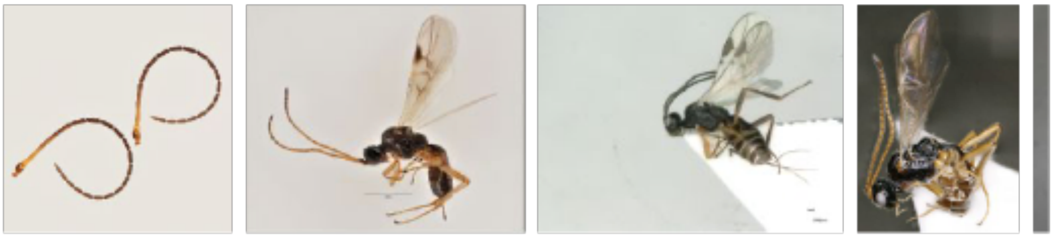

44 GEOREFERENCED RECORDS

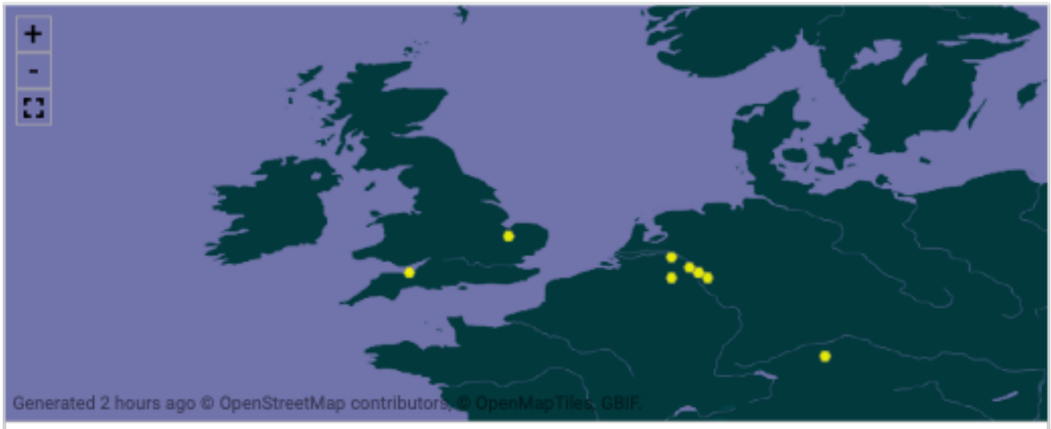

Any year 1885 - 2022 EXPLORE AREA

TYPE SPECIMENS

HOLOTYPE of Blacus dentatus Hellén, 1958 Hellén, Wolter Finland. MZH http://id.luomus.fi/GL.3314
Source: Hymenoptera Eastern Fennoscandia (Luomus)

TYPE of Blacus ruficornis (Nees, 1811) RBINS-Scientific Heritage urn:catalog:RBINS:TVC EVCT.1183
Source: RBINS DaRWIN

https://www.gbif.org/species/1252745 1/4

Figure S44: Global Biodiversity Information Facility (GBIF) Webpage *Blacus ruficornis*

Sanger_ID: SQ_2022_057_008
Data_ID: HA_290622_C5A (conventional HPS 2000K)

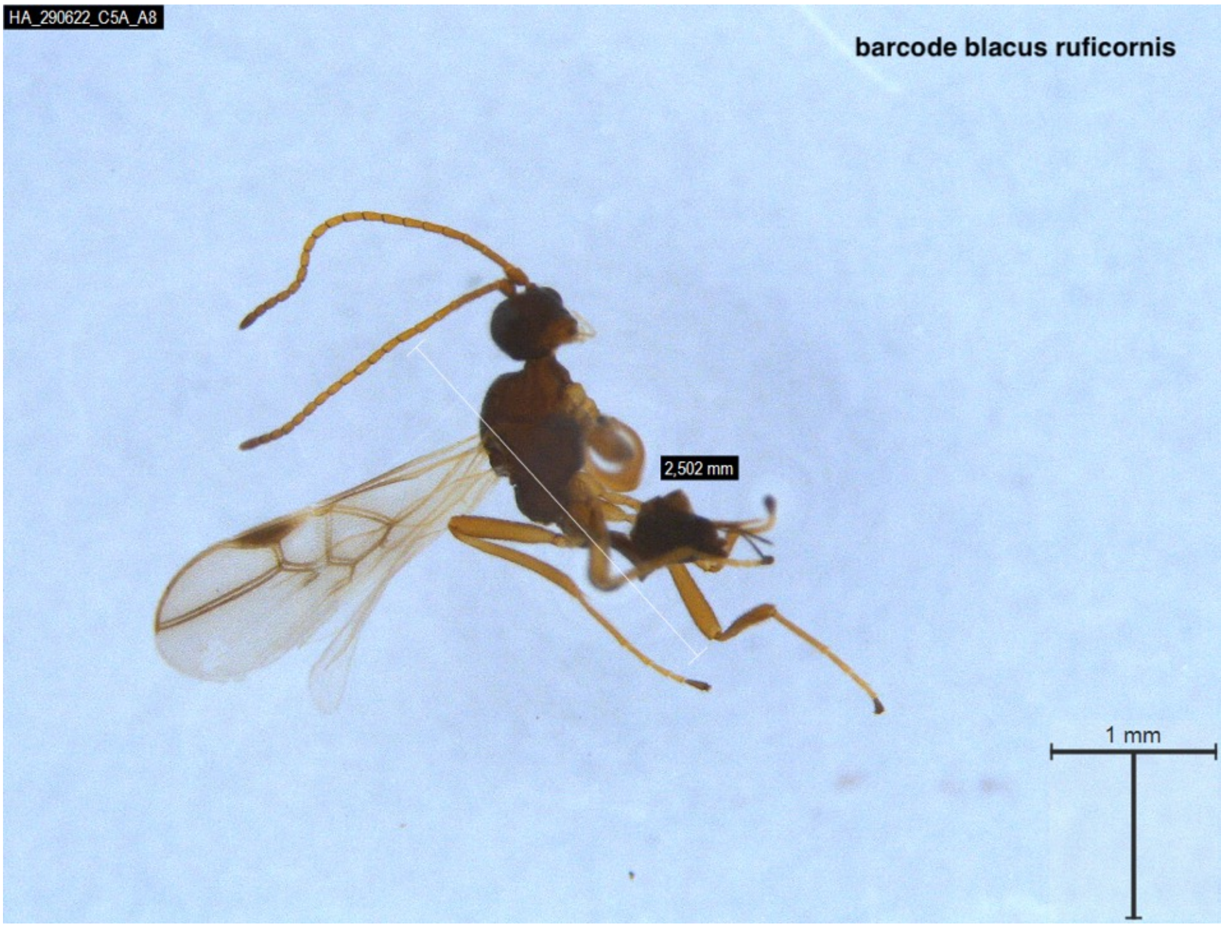

Figure S45: Blacus ruficornis

Sanger_ID: SQ_2022_057_012
Data_ID: HA_290622_C8A (conventional HPS 2000K)

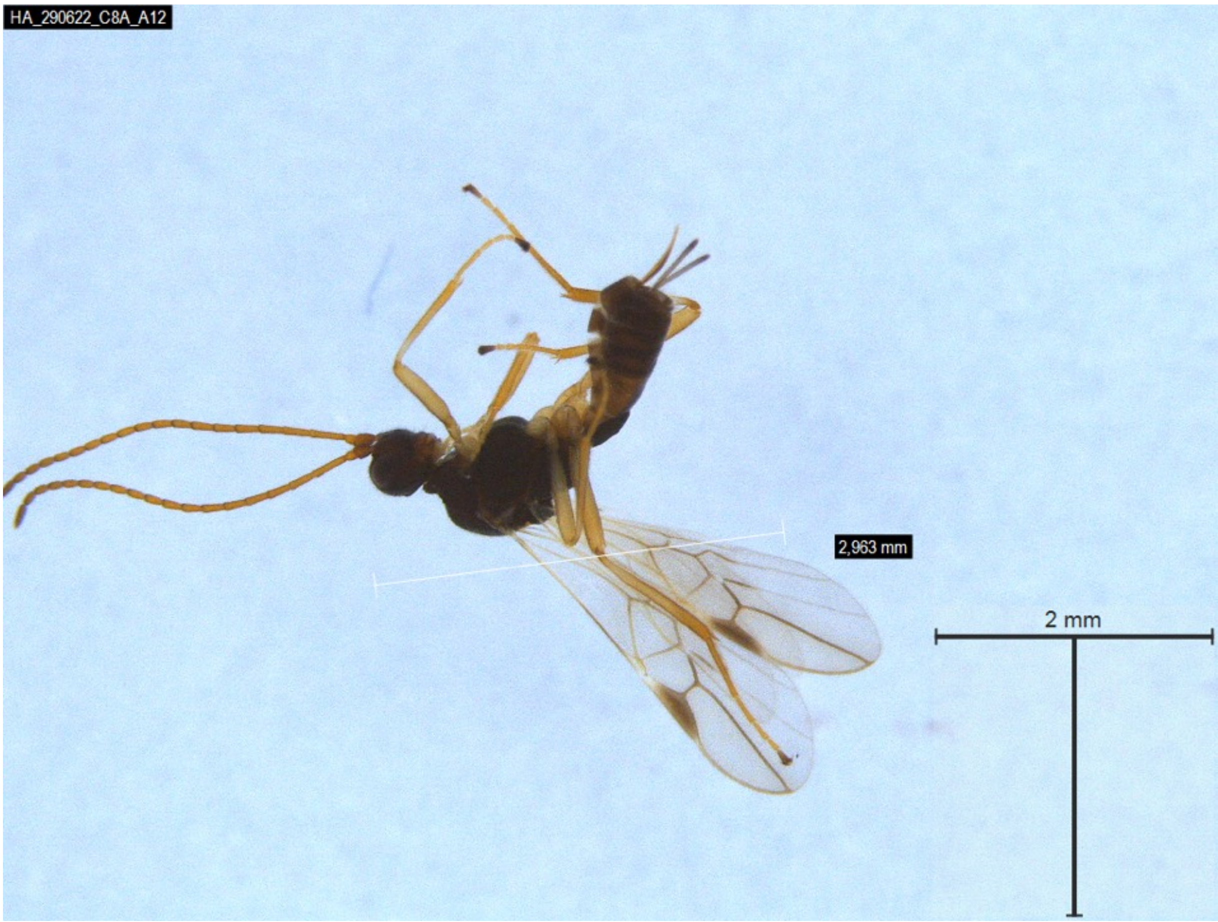

Figure S46: Blacus ruficornis

Sanger_ID: SQ_2022_057_044
Data_ID: HA_260722_C7C (conventional HPS 2000K)

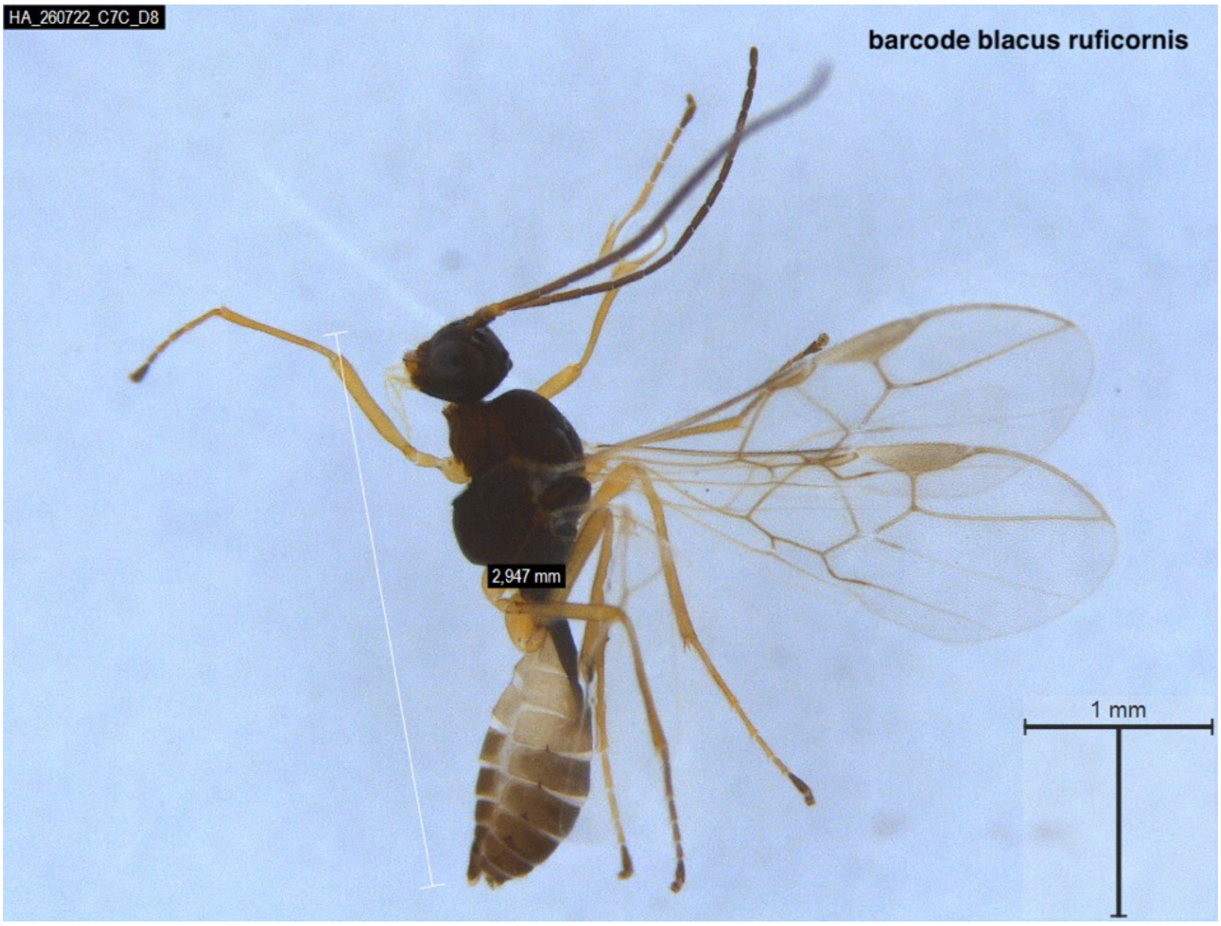

Figure S47: Blacus ruficornis

Sanger_ID: SQ_2022_057_072
Data_ID: HA_240822_C7J (conventional HPS 2000K)

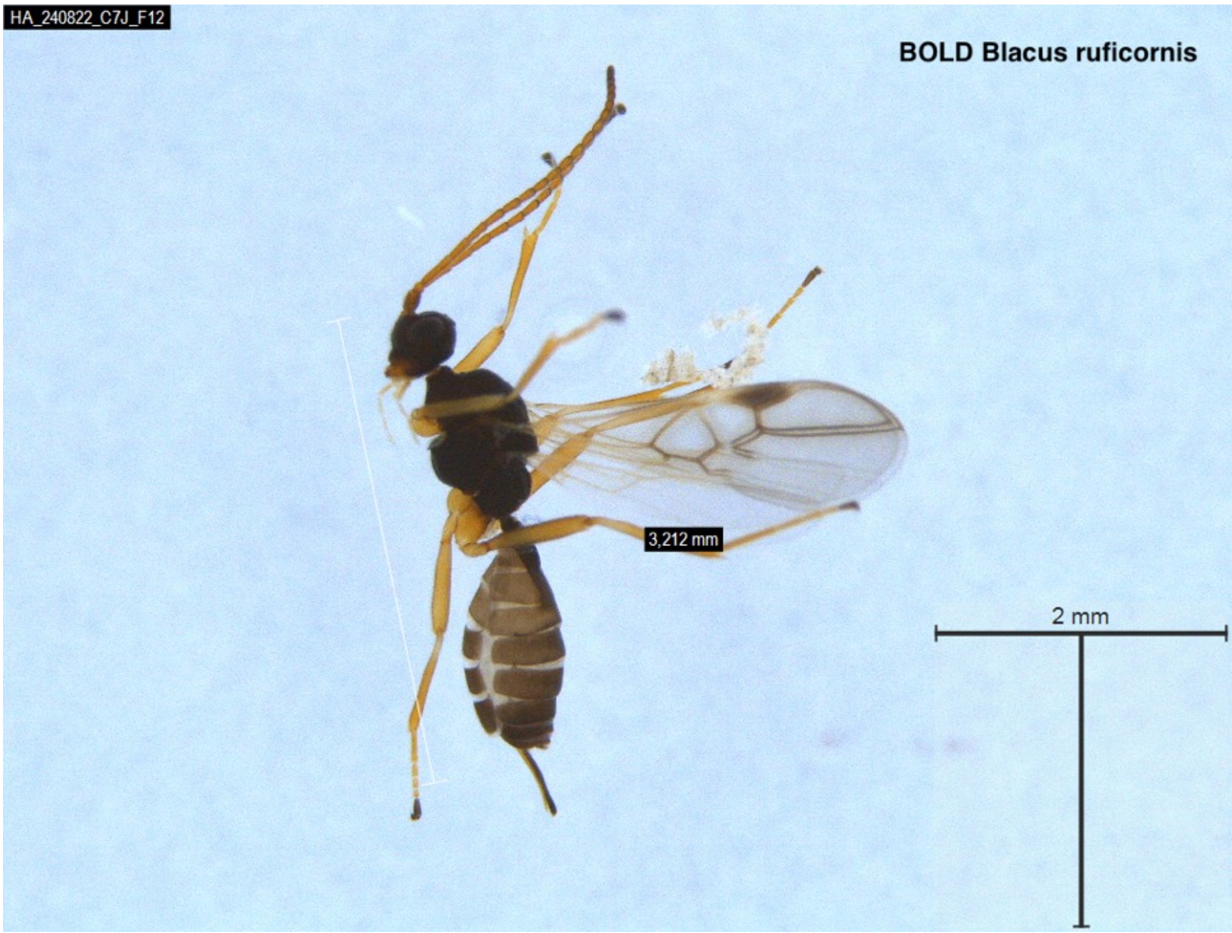

Figure S48: Blacus ruficornis

Sanger_ID: SQ_2022_057_032
Data_ID: HA_210722_C6C (conventional HPS 2000K)

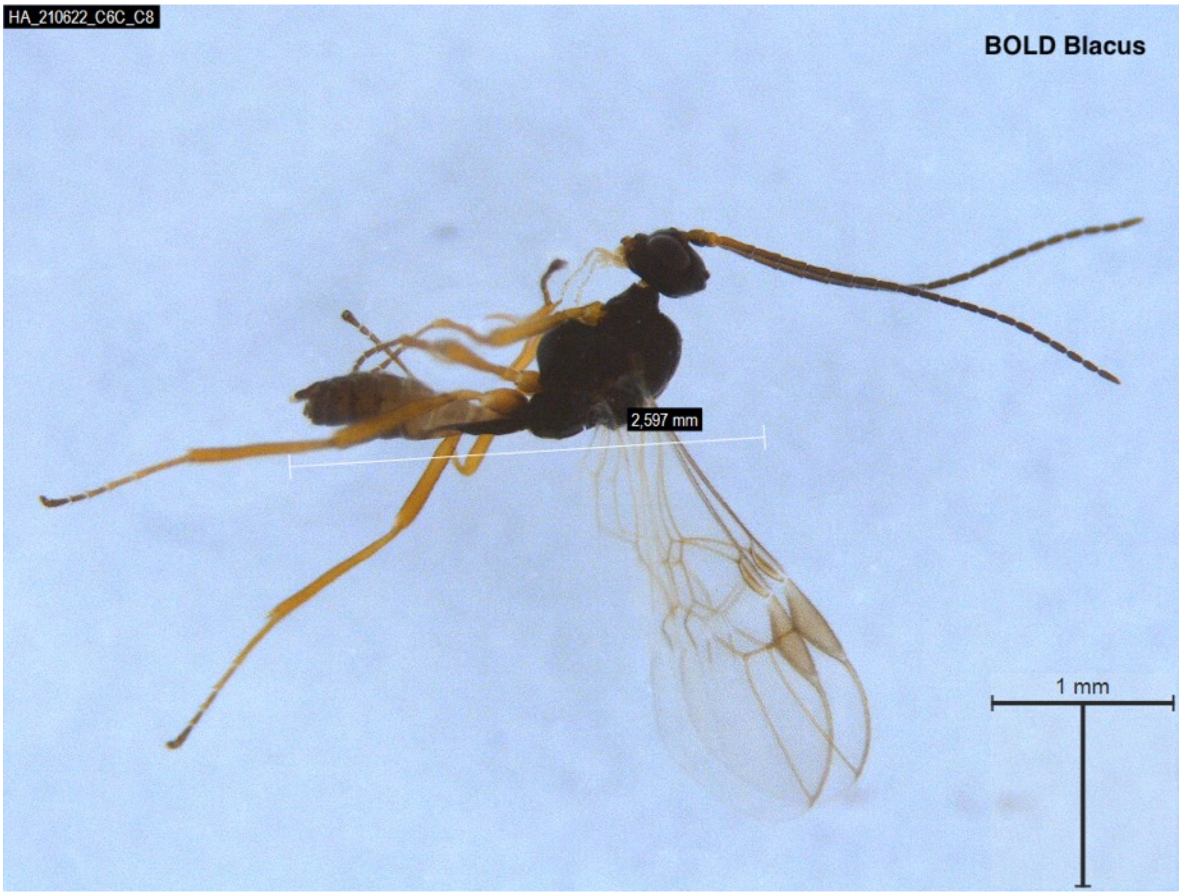

Figure S49: *Blacus ruficornis*

Sanger_ID: SQ_2022_057_043
Data_ID: HA_210722_C6A (conventional HPS 2000K)

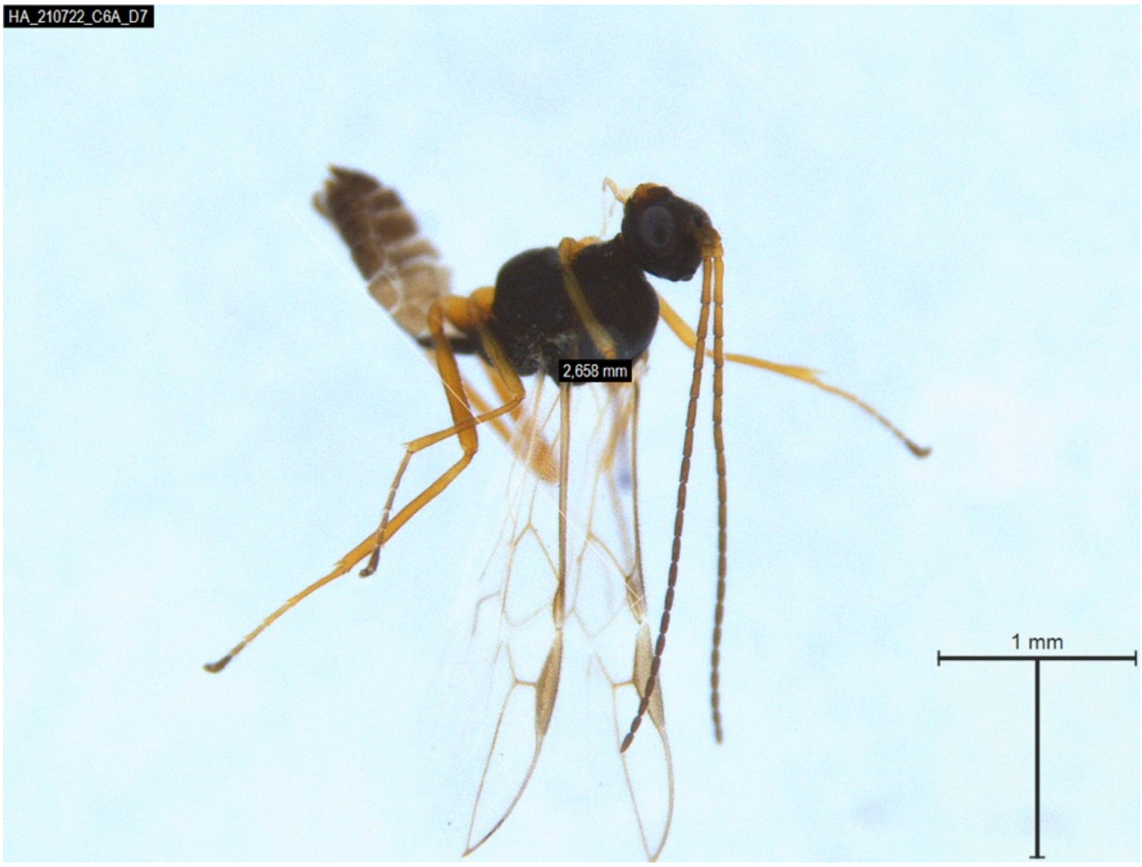

Figure S50: *Blacus ruficornis*

Sanger_ID: SQ_2022_057_067
Data_ID: HA_260722_C7B (conventional HPS 2000K)

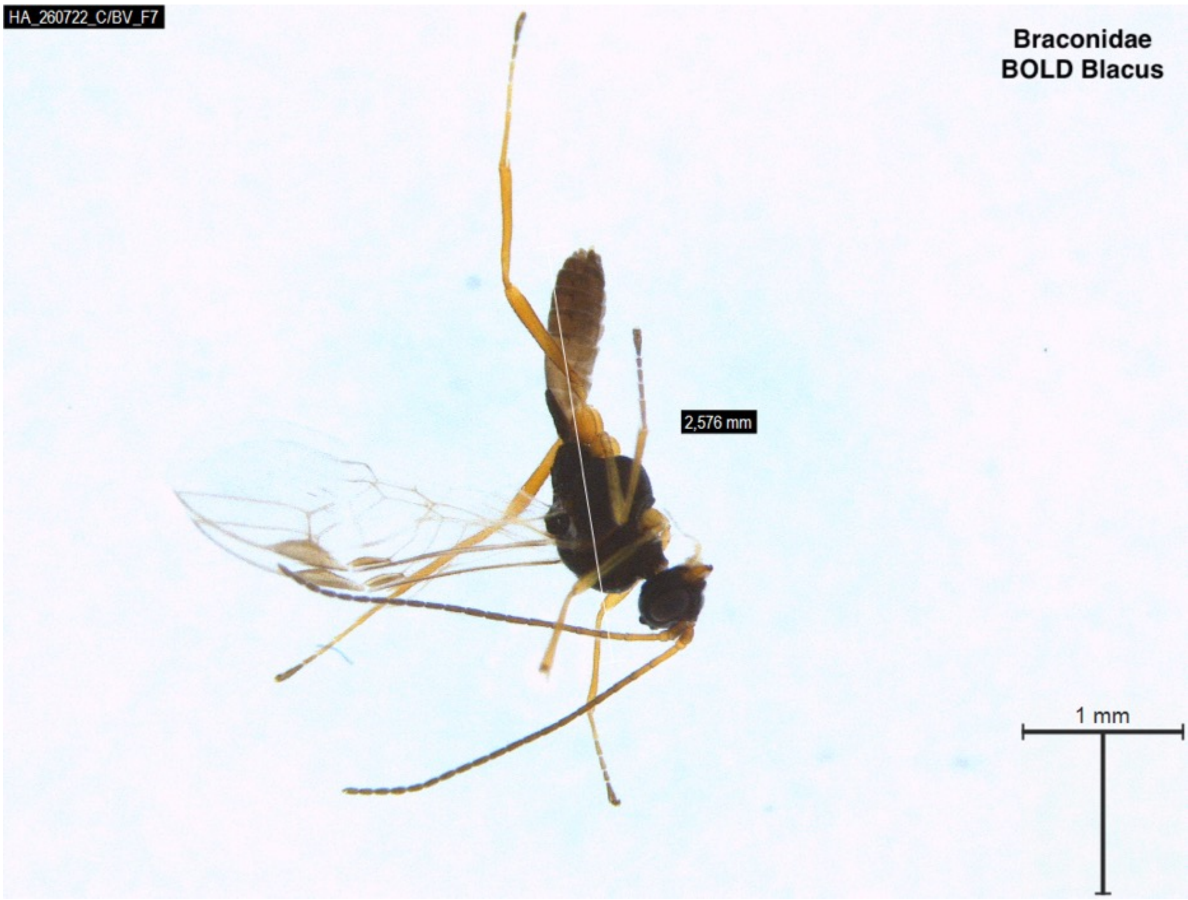

Figure S51: *Blacus ruficornis*

Sanger_ID: SQ_2022_057_079
Data_ID: HA_260722_C8B (conventional HPS 2000K)

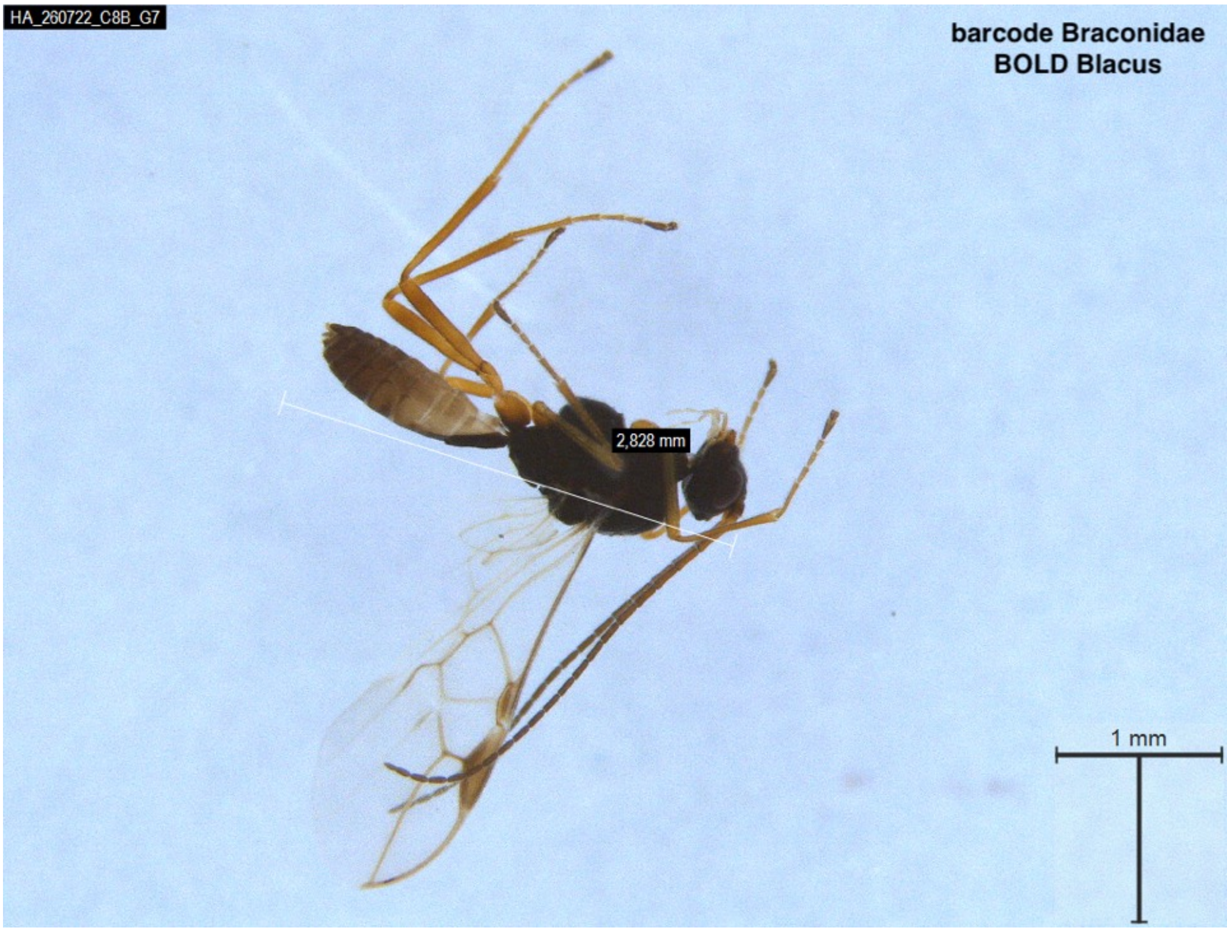

Figure S52: *Blacus ruficornis*

Sanger_ID: SQ_2022_057_091
Data_ID: HA_210722_C6B (conventional HPS 2000K)

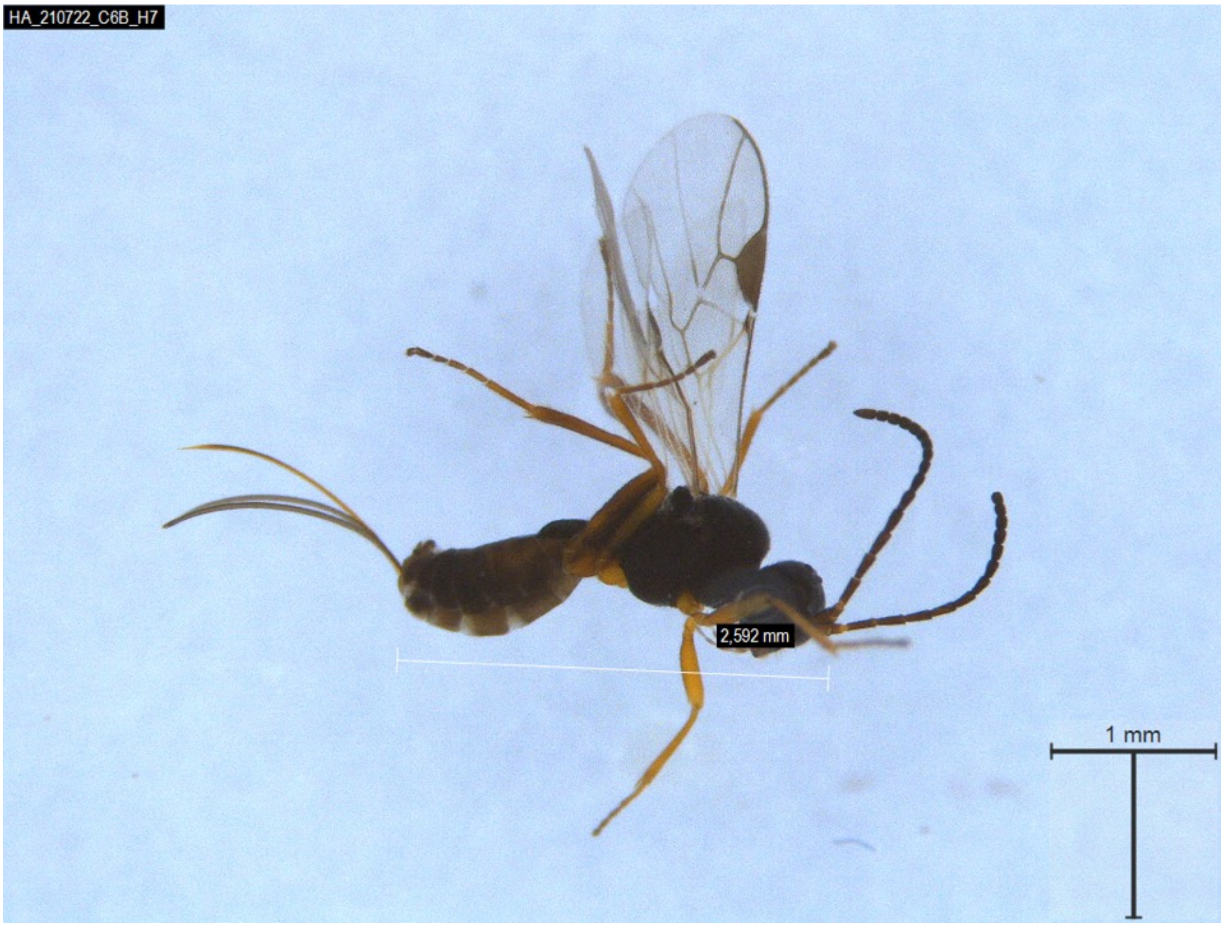

Figure S53: *Blacus ruficornis*

Sanger_ID: SQ_2022_057_098
Data_ID: HA_290622_C6B (conventional HPS 2000K)

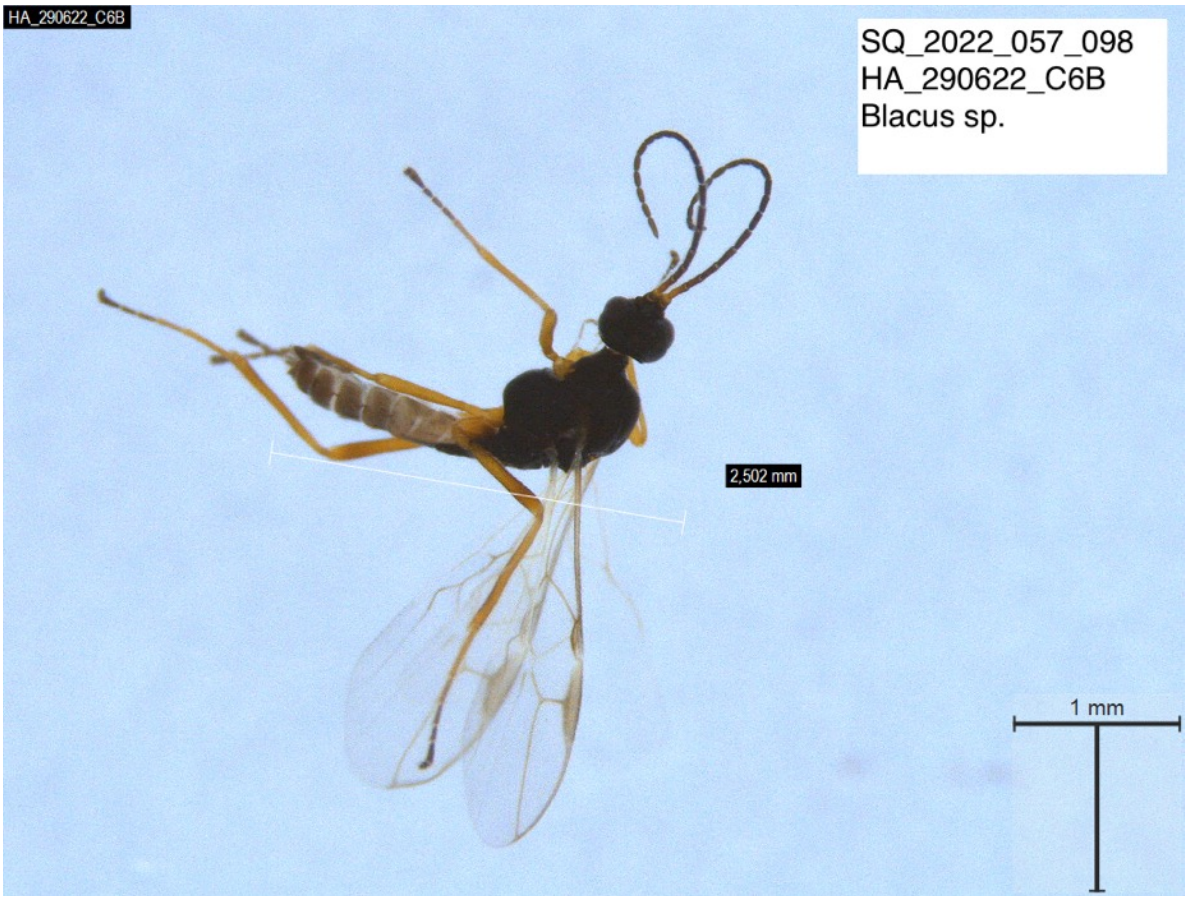

Figure S54: *Blacus ruficornis*

Blastothrix sp. (Encyrtidae)

Sanger_ID: SQ_2022_057_013
Data_ID: KA_190722_C16A (conventional LED 4000K)

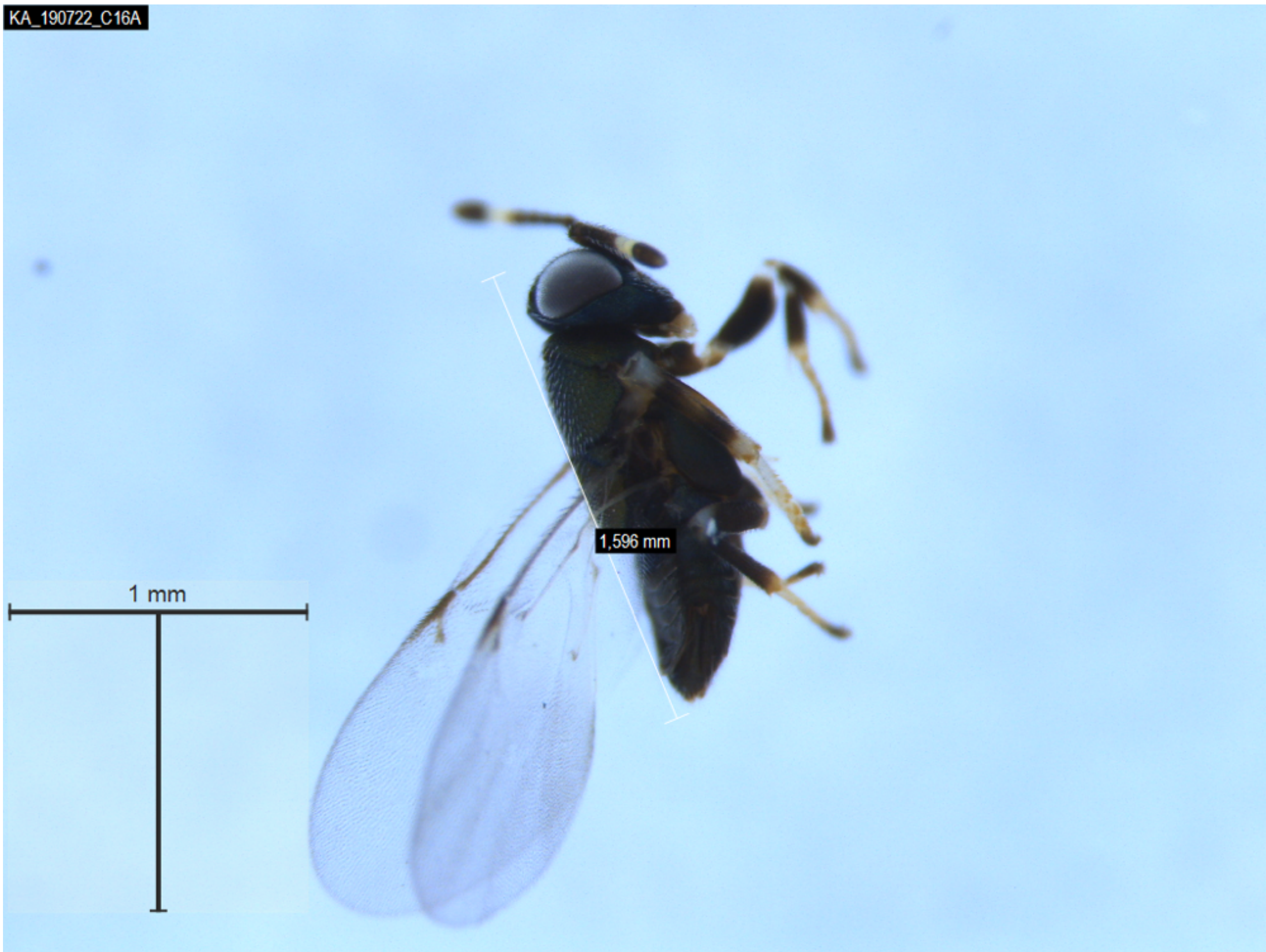

Figure S55: Blastothrix sp.

GENUS | ACCEPTED

Blastothrix Mayr, 1876

Published in: Verh. Zool.-bot. Ges. Wien, 25, Abh. source: Universal Chalcidoidea Database

324 OCCURRENCES 29 SPECIES

OVERVIEW 2 TREATMENTS METRICS REFERENCE TAXON

6 OCCURRENCES WITH IMAGES

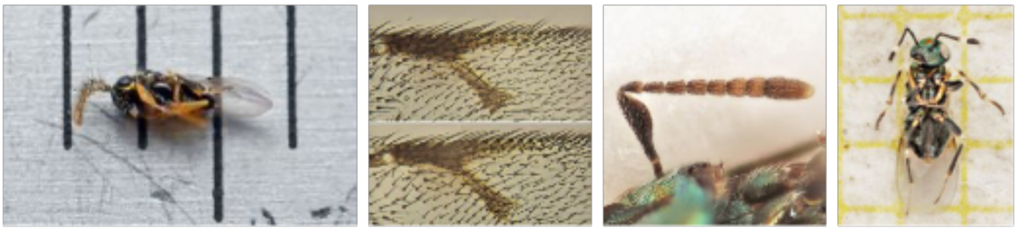

37 GEOREFERENCED RECORDS

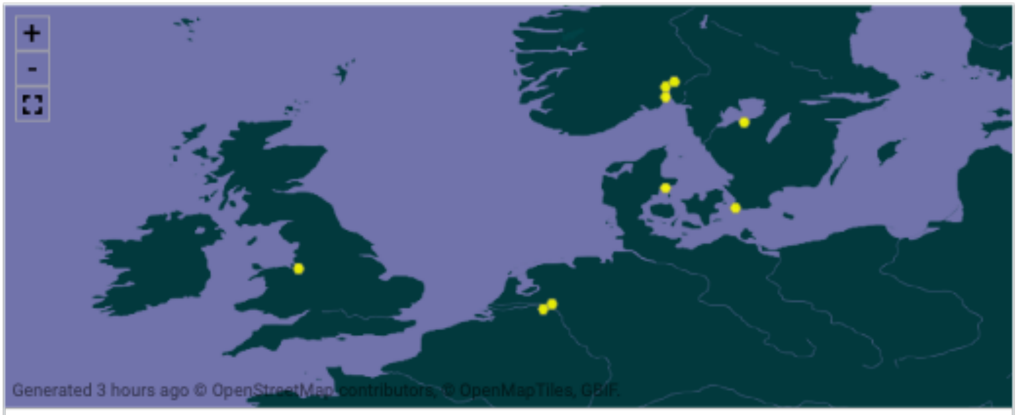

Any year 1874 - 2022 EXPLORE AREA

APPEARS IN 21 CHECKLIST DATASETS:

GBIF Backbone Taxonomy
As *Blastothrix* Mayr, 1876

Catalogue of Life Checklist
As *Blastothrix*

NCBI Taxonomy
As *Blastothrix*

Figure S56: Global Biodiversity Information Facility (GBIF) Webpage *Blastothrix*

Ceraphronidae sp1. (Ceraphronidae)

Sanger_ID: SQ_2022_057_037
Data_ID: KA_290822_C12A (tailored LED 4000K)

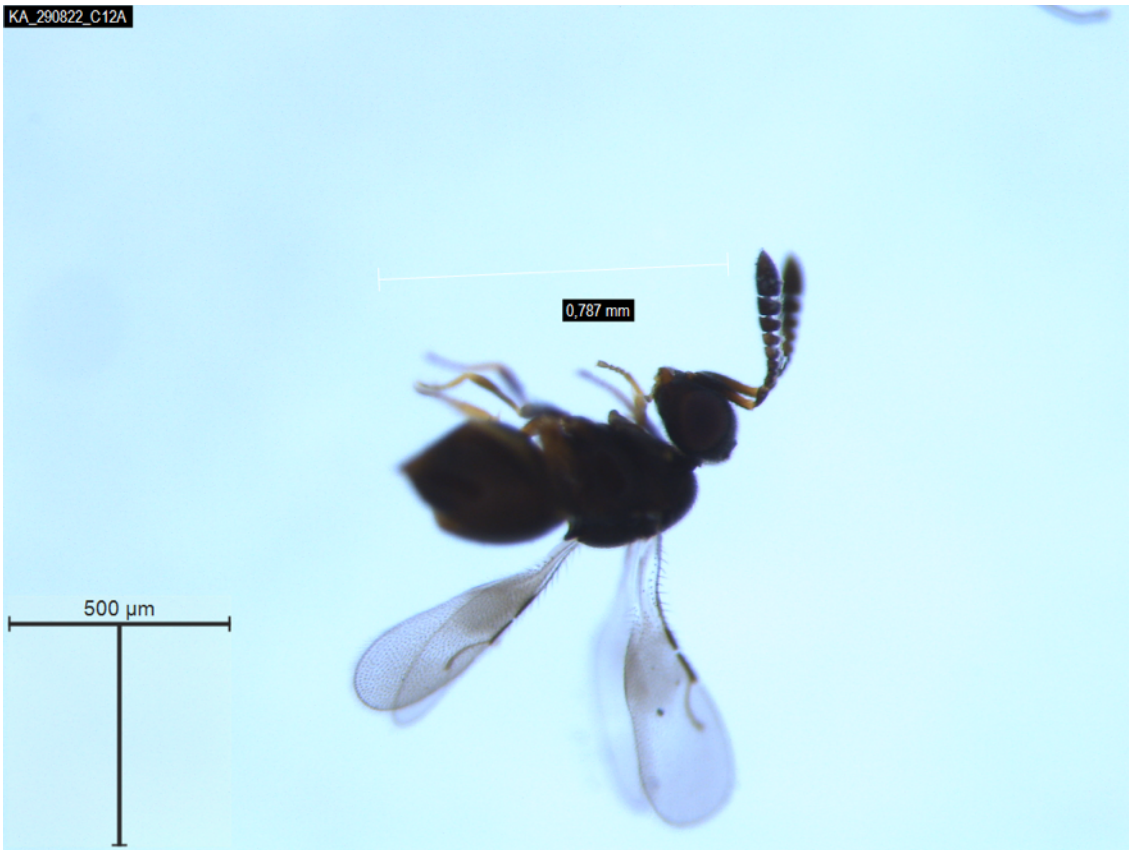

Figure S57 *Ceraphronidae sp1.*

Ceraphronidae sp2. ***(Ceraphronidae)***

Sanger_ID: SQ_2022_057_058
Data_ID: KA_270921_C9B (conventional LED 4000K)

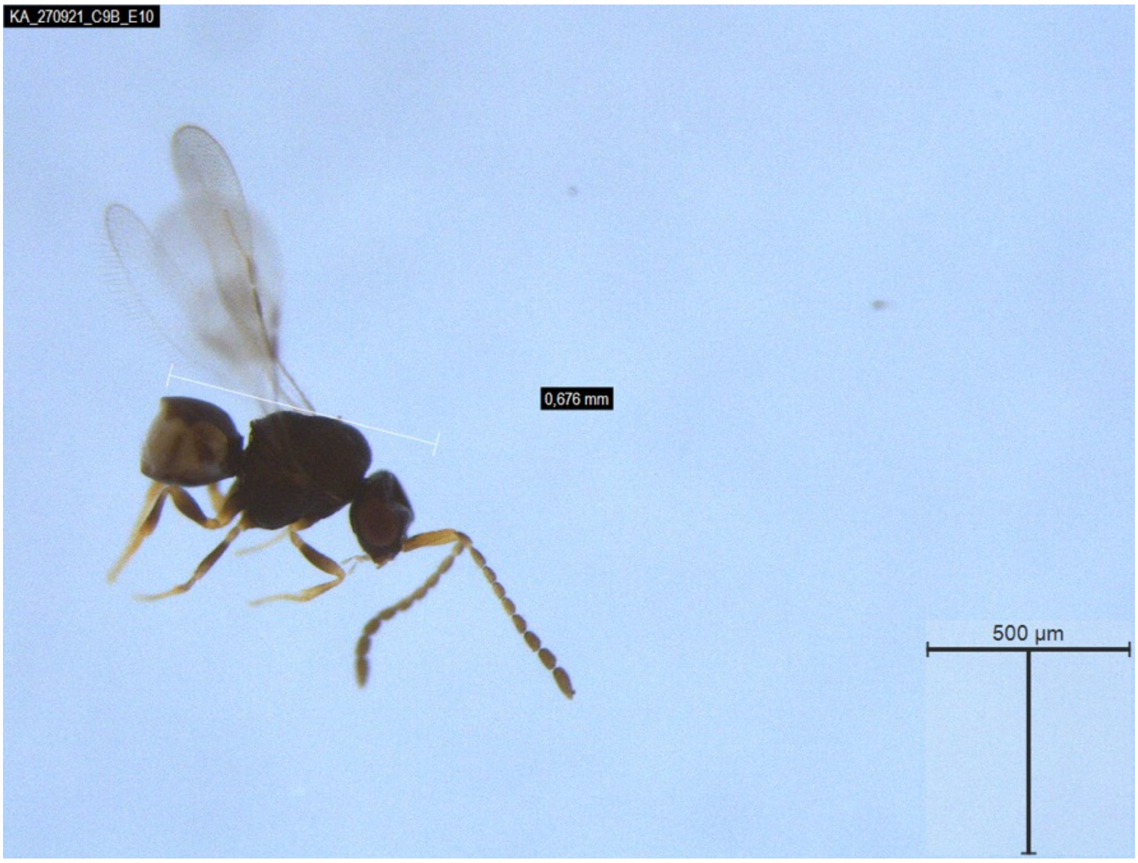

Figure S58: *Ceraphronidae sp2.*

Ceraphronidae sp3. (Ceraphronidae)

Sanger_ID: SQ_2022_057_093
Data_ID: BR_020921_C25A (conventional HPS 2000K)

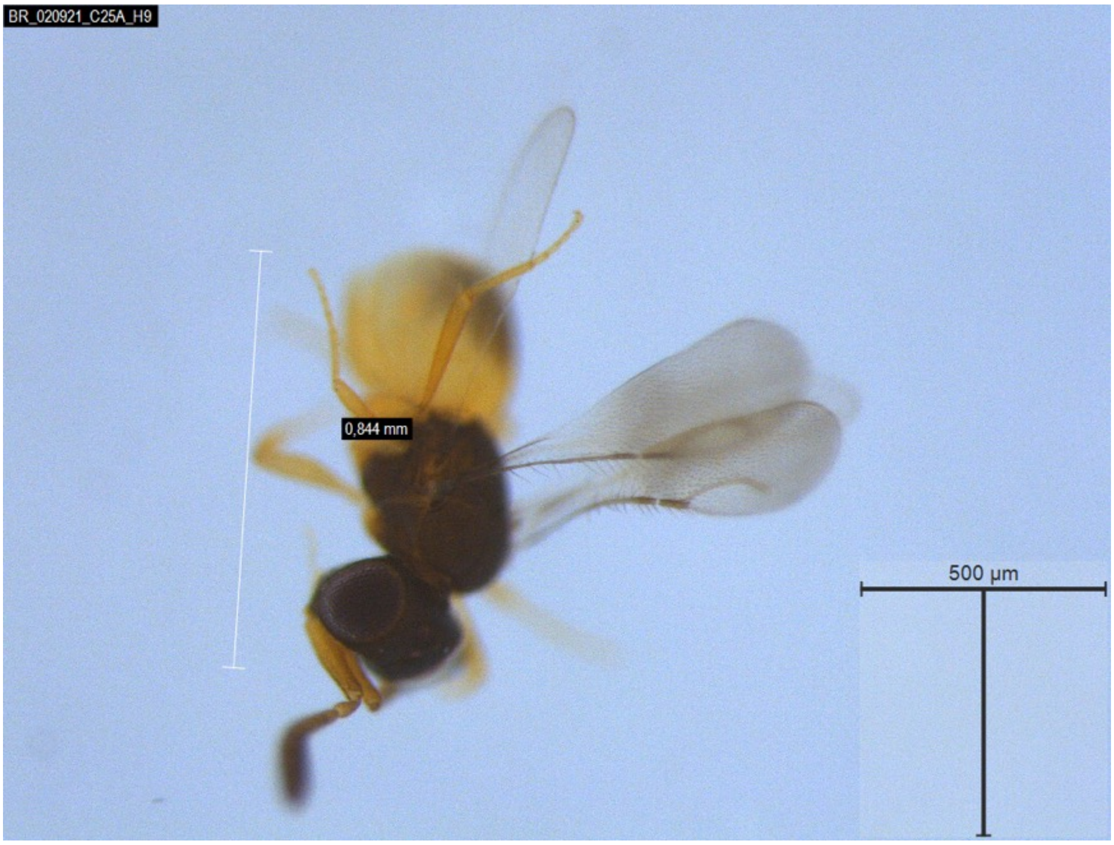

Figure S59: Ceraphronidae sp3.

FAMILY | ACCEPTED

Ceraphronidae

In: GBIF Backbone Taxonomy

Ceraphronid wasps In English

27,816 OCCURRENCES 403 SPECIES

OVERVIEW 1 TREATMENT METRICS

798 OCCURRENCES WITH IMAGES

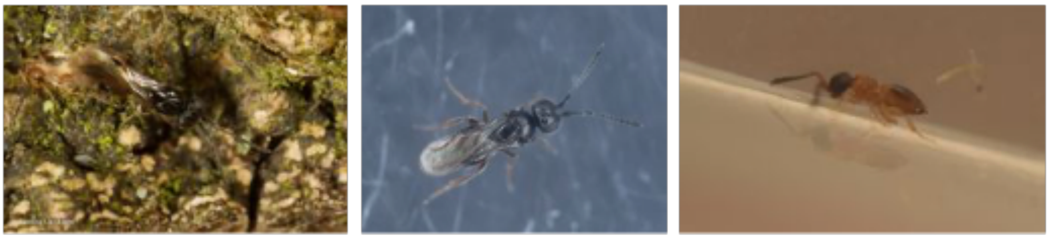

3,001 GEOREFERENCED RECORDS

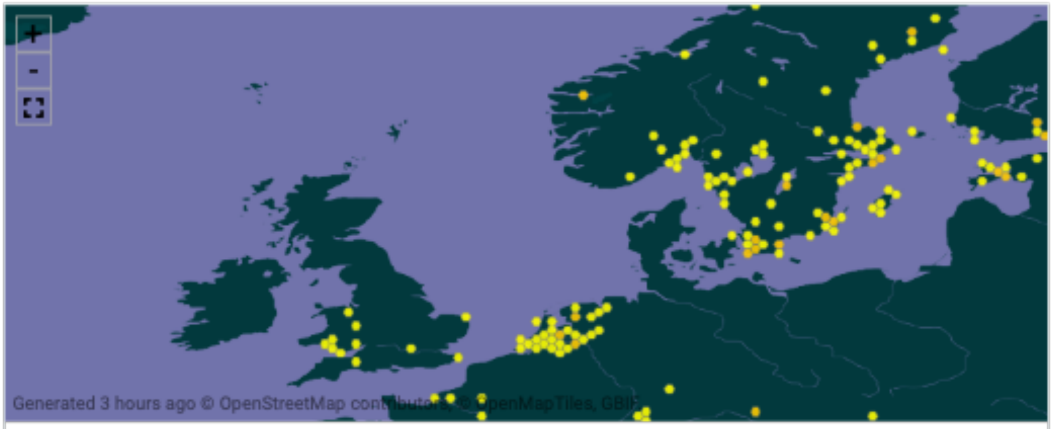

Any year 1864 - 2023 EXPLORE AREA

VERNACULAR NAMES

Ceraphronid wasps In English
sources: Catalogue of Life Checklist + 1 more dataset

Ceraphronids In English
sources: Integrated Taxonomic Information System (ITIS) + 1 more dataset

Ceraphronides In French
sources: Integrated Taxonomic Information System (ITIS) + 1 more dataset

Figure S60: Global Biodiversity Information Facility (GBIF) Webpage *Ceraphronidae*

Copidosoma sp. (Encyrtidae)

Sanger_ID: SQ_2022_057_001
Data_ID: HA_290622_C6A (conventional HPS 2000K)

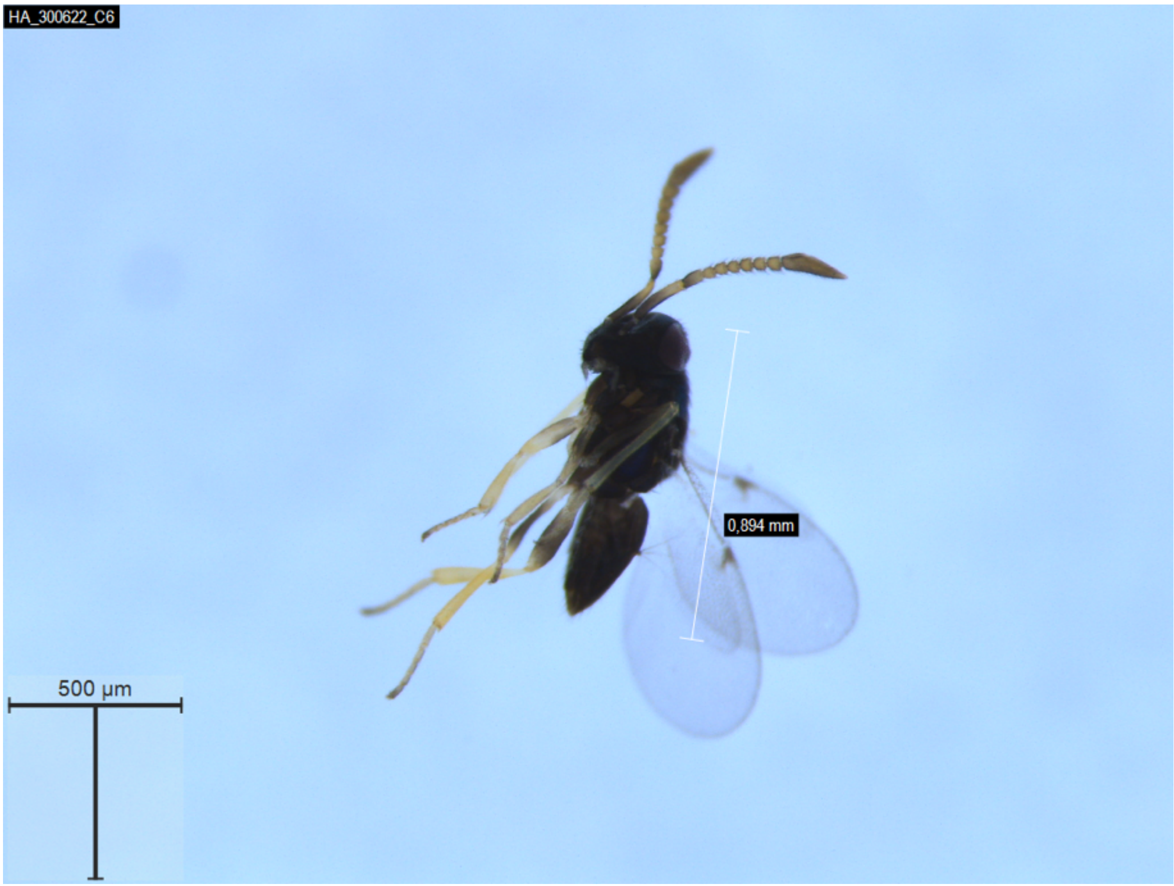

Figure S61: *Copidosoma sp.*

GENUS | ACCEPTED

Copidosoma Ratzeburg, 1844

Published in: Ratzeburg, Julius T. C. 1844. Die Ichneumonen der Forstinsecten in forstlicher und entomologischer Beziehung, als anhang zur Abbildung und Beschreibung der Forstinsecten. Nicolaischen Buchhandlung, Berlin. Vol. 1: 1-224.
source: Universal Chalcidoidea Database

5,369 OCCURRENCES 175 SPECIES

OVERVIEW 3 TREATMENTS METRICS REFERENCE TAXON

156 OCCURRENCES WITH IMAGES

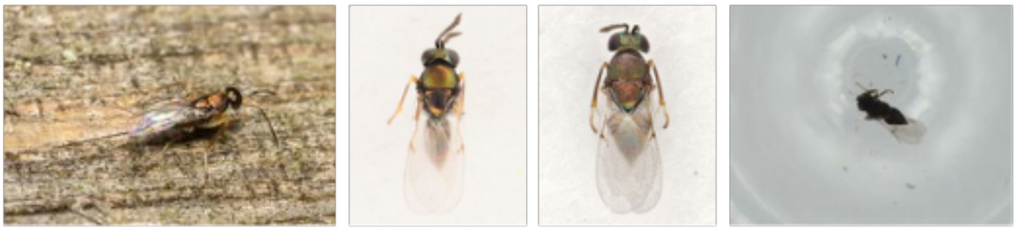

810 GEOREFERENCED RECORDS

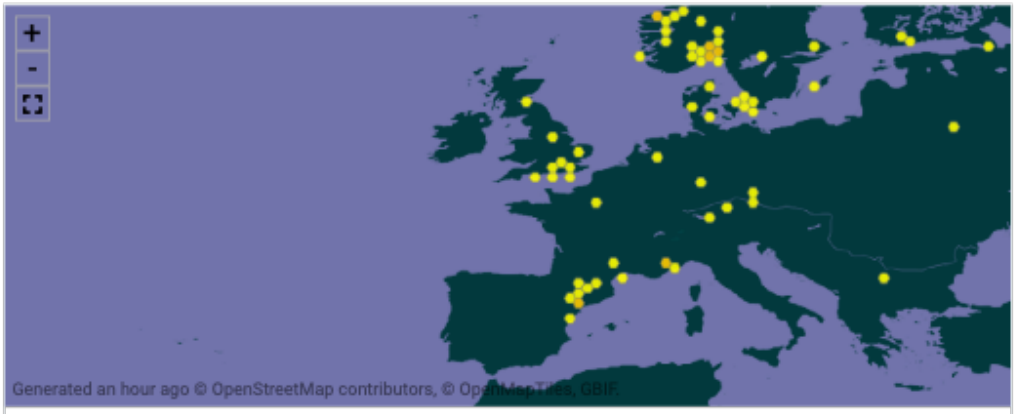

Any year 1824 - 2023 EXPLORE AREA

APPEARS IN 33 CHECKLIST DATASETS:

GBIF Backbone Taxonomy
As *Copidosoma* Ratzeburg, 1844

Catalogue of Life Checklist
As *Copidosoma*

Figure S62: Global Biodiversity Information Facility (GBIF) Webpage *Copidosoma*

Cratichneumon sp. (Ichneumonidae)

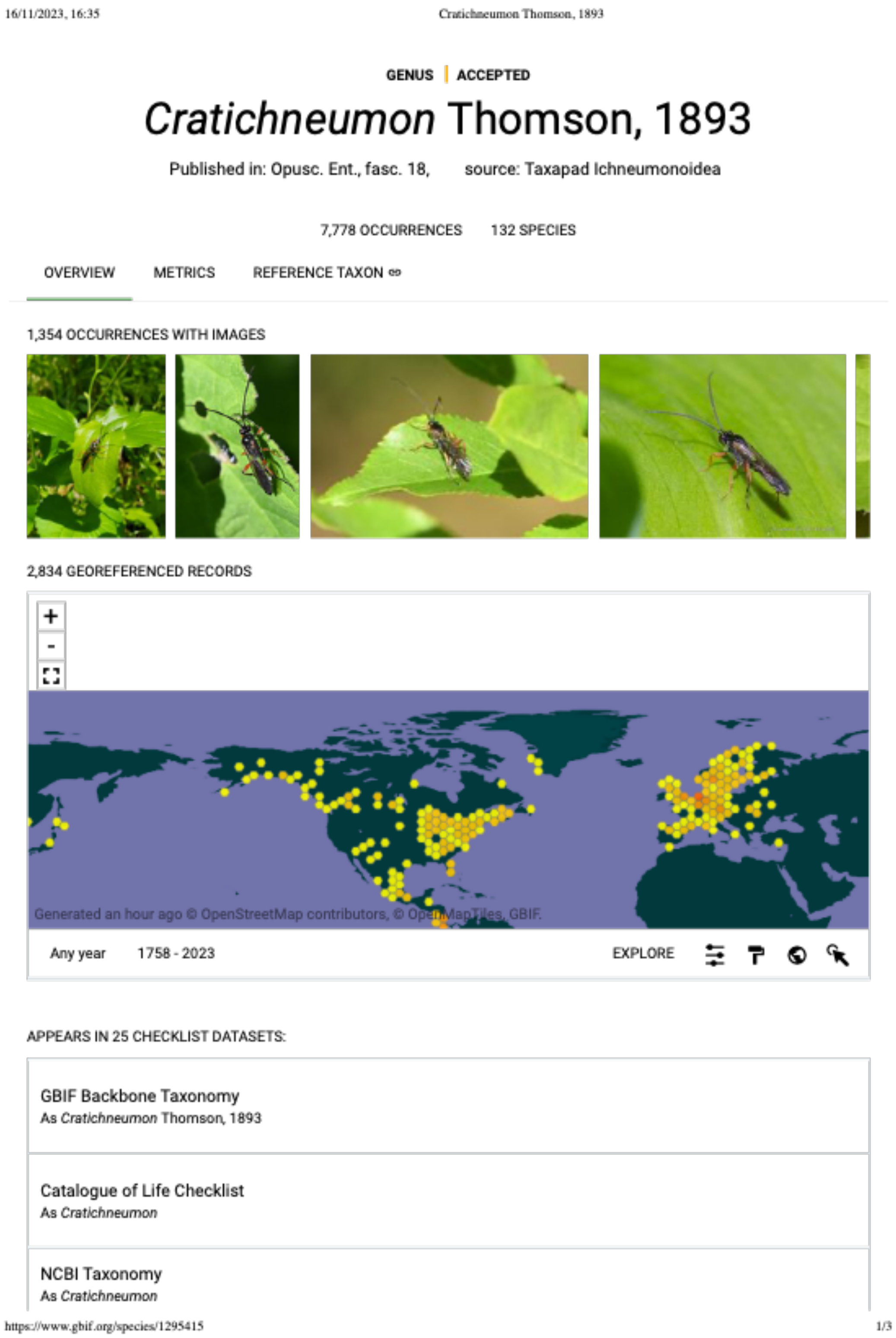
16/11/2023, 16:35 Cratichneumon Thomson, 1893

GENUS | ACCEPTED

Cratichneumon Thomson, 1893

Published in: Opusc. Ent., fasc. 18, source: Taxapad Ichneumonoidea

7,778 OCCURRENCES 132 SPECIES

OVERVIEW METRICS REFERENCE TAXON

1,354 OCCURRENCES WITH IMAGES

2,834 GEOREFERENCED RECORDS

Generated an hour ago © OpenStreetMap contributors, © OpenMapTiles, GBIF.

Any year 1758 - 2023 EXPLORE

APPEARS IN 25 CHECKLIST DATASETS:

GBIF Backbone Taxonomy
As *Cratichneumon* Thomson, 1893

Catalogue of Life Checklist
As *Cratichneumon*

NCBI Taxonomy
As *Cratichneumon*

https://www.gbif.org/species/1295415 1/3

Figure S63 Global Biodiversity Information Facility (GBIF) Webpage *Cratichneumon*

Sanger_ID: SQ_2022_057_105
Data_ID: BR_300822_C27A (conventional HPS 2000K)

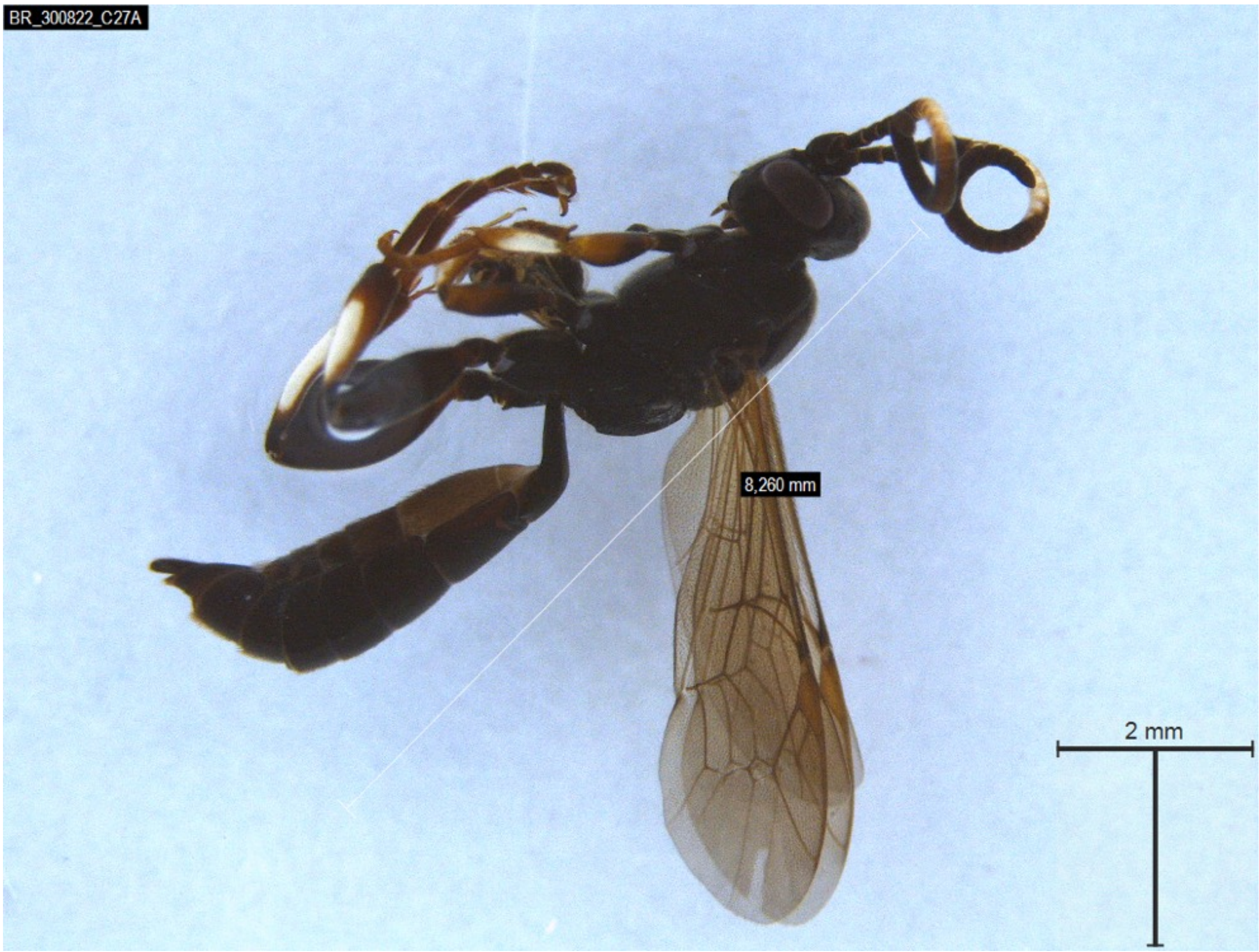

Figure S64: *Cratichneumon sp.*

Cratomus megacephalus (Pteromalidae)

SPECIES | ACCEPTED

Cratomus megacephalus (Fabricius, 1793)

Published in: Curtis, J. A guide to an arrangement of British Insects; being a catalogue of all the named species hitherto discovered. PAGES: 256pp. (1829).
source: Universal Chalcidoidea Database

Basionym: *Cynips megacephala* Fabricius, 1793

83 OCCURRENCES 1 INFRASPECIES

OVERVIEW METRICS REFERENCE TAXON

25 OCCURRENCES WITH IMAGES

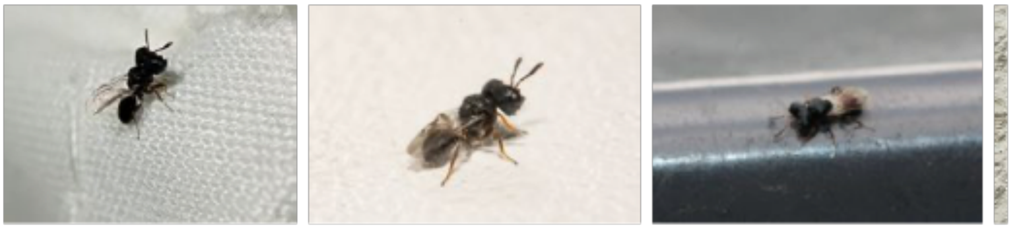

48 GEOREFERENCED RECORDS

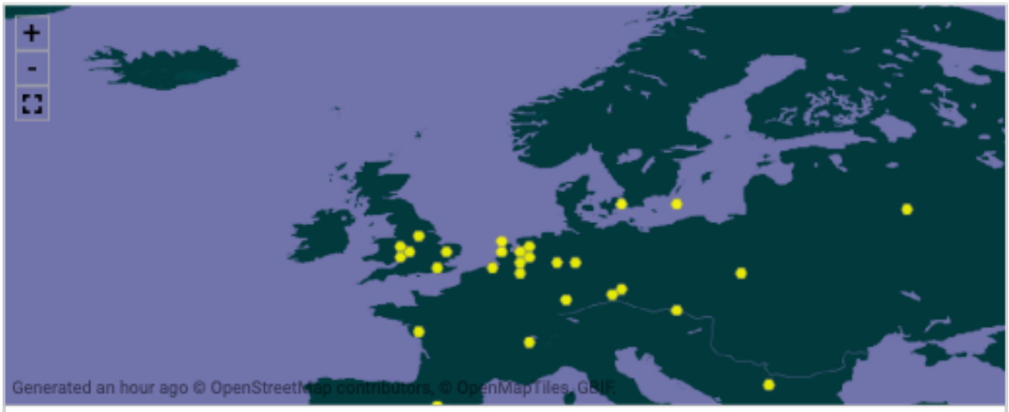

Any year 1894 - 2023 EXPLORE AREA

TYPE SPECIMENS

LECTOTYPE of Diplolepis megacephala (Fabricius, 1793) NHMD EN NHMD48561
Source: NHMD Entomology Collection

PARALECTOTYPE of Diplolepis megacephala (Fabricius, 1793) NHMD EN NHMD48562
Source: NHMD Entomology Collection

Figure S65: Global Biodiversity Information Facility (GBIF) Webpage *Cratomus megacephalus*

Sanger_ID: SQ_2022_057_010
Data_ID: BR_020621_C24A (conventional HPS 2000K)

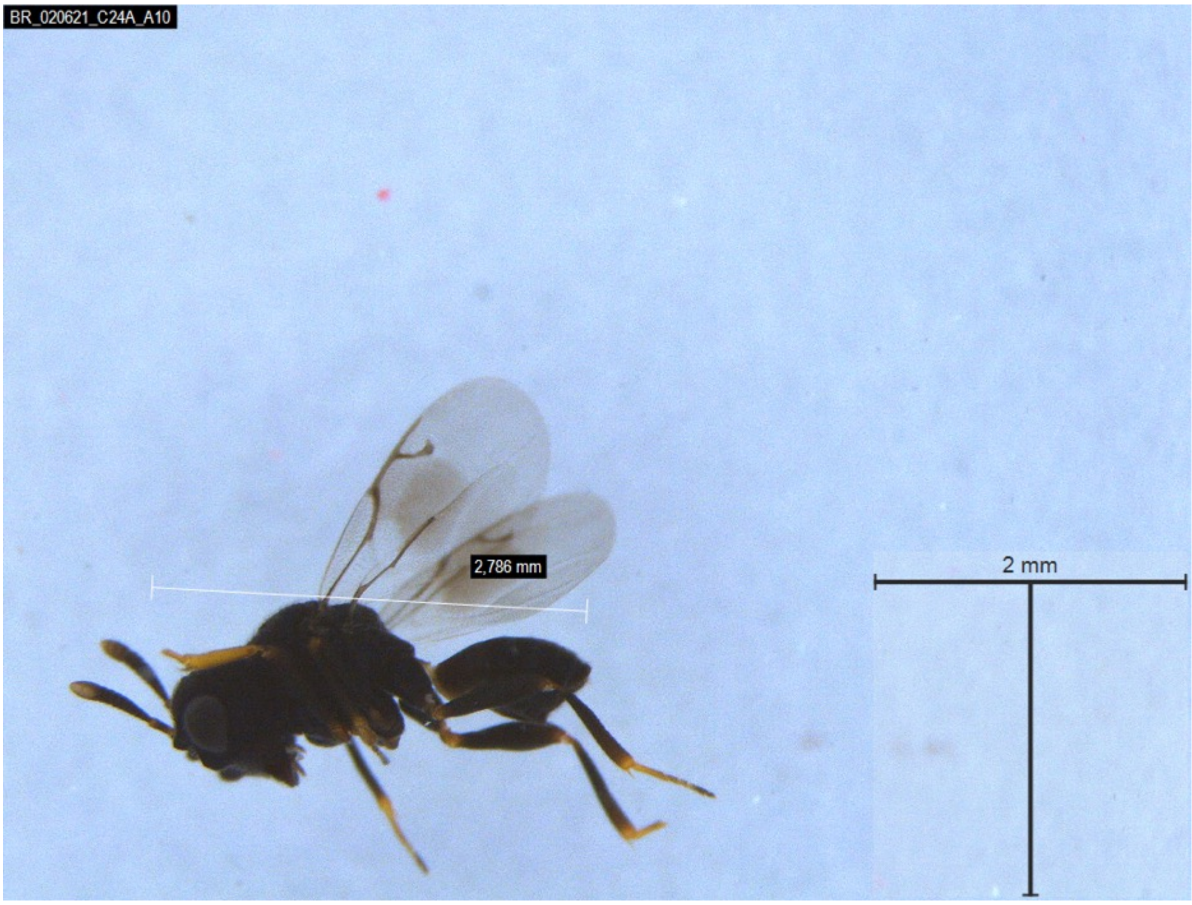

Figure S66: Cratomus megacephalus

Cyclogastrella sp. (Pteromalidae)

GENUS | ACCEPTED

Cyclogastrella Bukowskii, 1938

Published in: Rev. Ent. U. R. S. S., 27 source: Universal Chalcidoidea Database

158 OCCURRENCES 8 SPECIES

OVERVIEW METRICS REFERENCE TAXON

1 OCCURRENCE WITH IMAGES

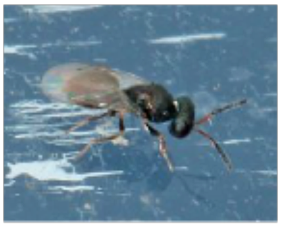

77 GEOREFERENCED RECORDS

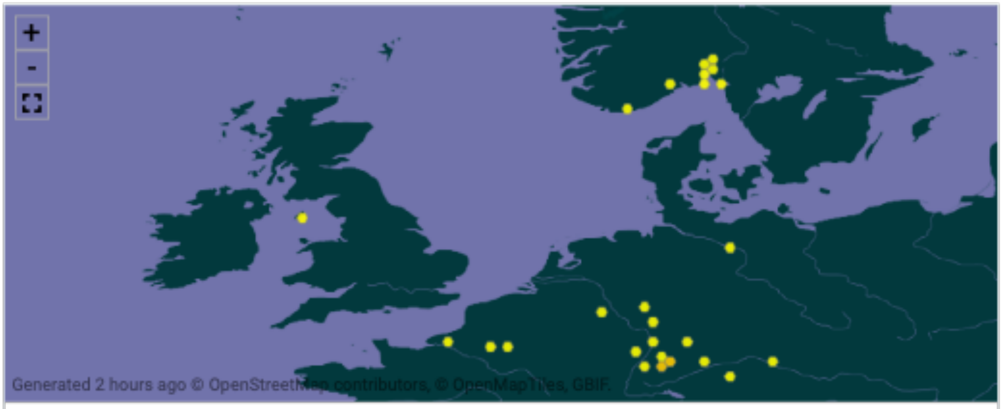

Any year 1957 - 2023 EXPLORE AREA

APPEARS IN 19 CHECKLIST DATASETS:

GBIF Backbone Taxonomy
As *Cyclogastrella* Bukowskii, 1938

Catalogue of Life Checklist
As *Cyclogastrella*

NCBI Taxonomy
As *Cyclogastrella*

Figure S67: Global Biodiversity Information Facility (GBIF) Webpage *Cyclogastrella*

Sanger_ID: SQ_2022_057_073
Data_ID: BR_160822_C27A (conventional HPS 2000K)

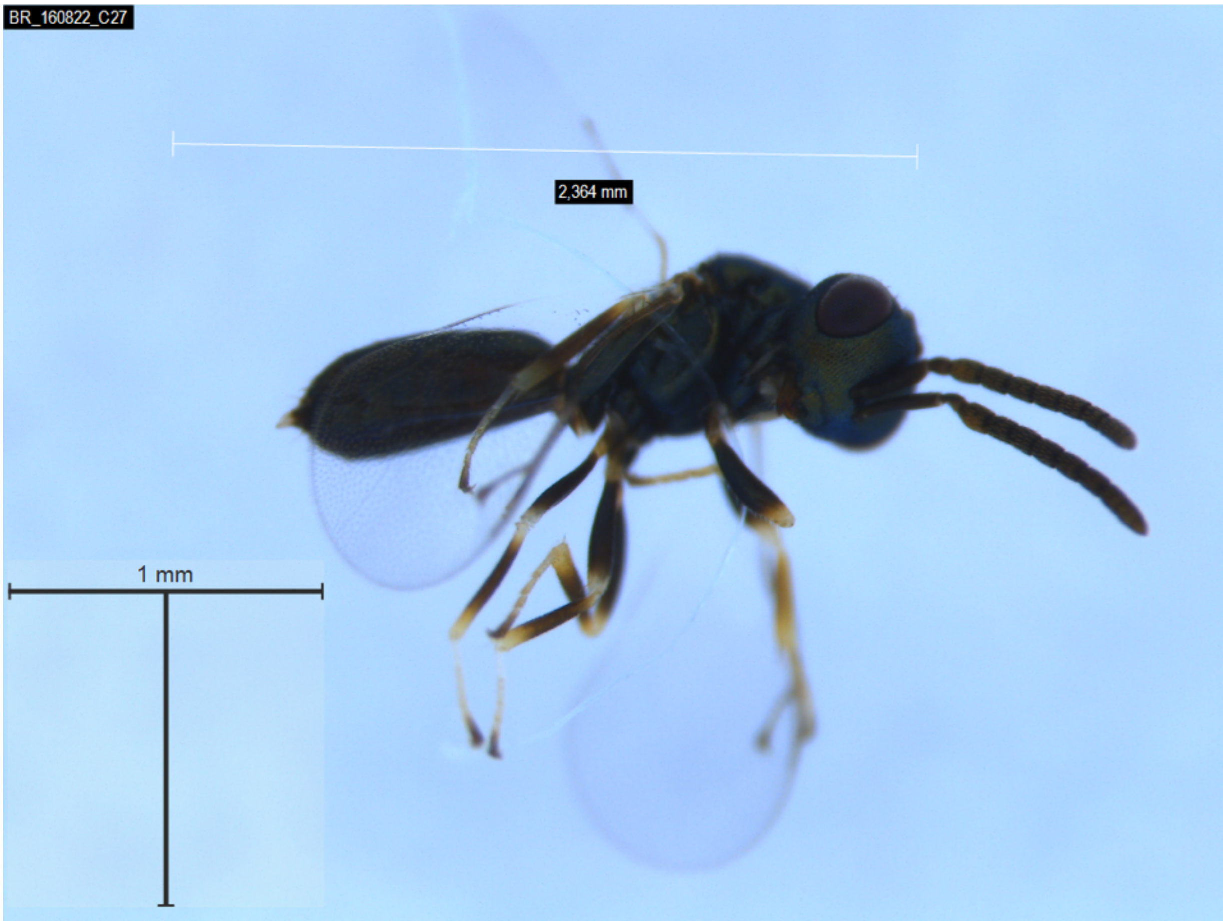

Figure S68: *Cyclogastrella sp. (cf simplex)*

Diadegma fenestrale (Ichneumonidae)

16/11/2023, 16:56 Diadegma fenestrale (Holmgren, 1860)

SPECIES | ACCEPTED

Diadegma fenestrale (Holmgren, 1860)

Published in: Holmgren, A.E. Forsok till uppstallning och beskrifning af de i Sverige funna Ophionider. (Monographia Ophionidum Sueciae). Kongliga Svenska Vetenskapsakademiens Handlingar. 2(8):1-158. (1860).

source: Taxapad Ichneumonoidea

Basionym: *Limneria fenestrale* Holmgren, 1860

201 OCCURRENCES 2 INFRASPECIES

OVERVIEW METRICS REFERENCE TAXON

2 OCCURRENCES WITH IMAGES

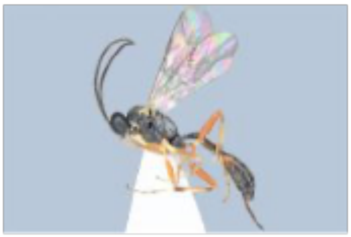

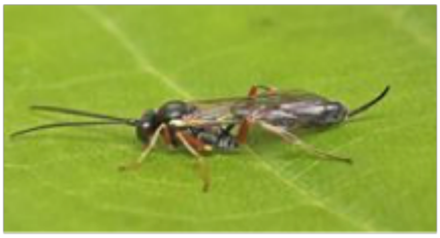

96 GEOREFERENCED RECORDS

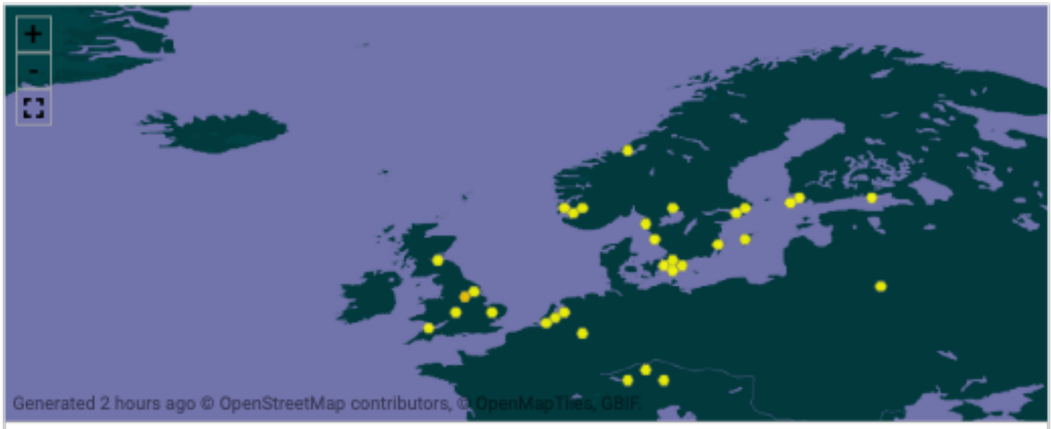

Any year 1800 - 2023 EXPLORE AREA

TYPE SPECIMENS

LECTOTYPE of Diadegma fenestrale (Holmgren, 1860) Carl Henrik Boheman Sweden. NRM NHRS NHRS-HEVA000000014

Source: Entomological Collections (NHRS), Swedish Museum of Natural History (NRM)

TYPE of Diadegma fenestrale (Holmgren, 1860) Carl Henrik Boheman Sweden. NRM NHRS NHRS-HEVA000000015

Source: Entomological Collections (NHRS), Swedish Museum of Natural History (NRM)

https://www.gbif.org/species/1291084 1/4

Figure S69: Global Biodiversity Information Facility (GBIF) Webpage *Diadegma fenestrale*

Sanger_ID: SQ_2022_057_070
Data_ID: KA_270921_C15A (conventional LED 4000K)

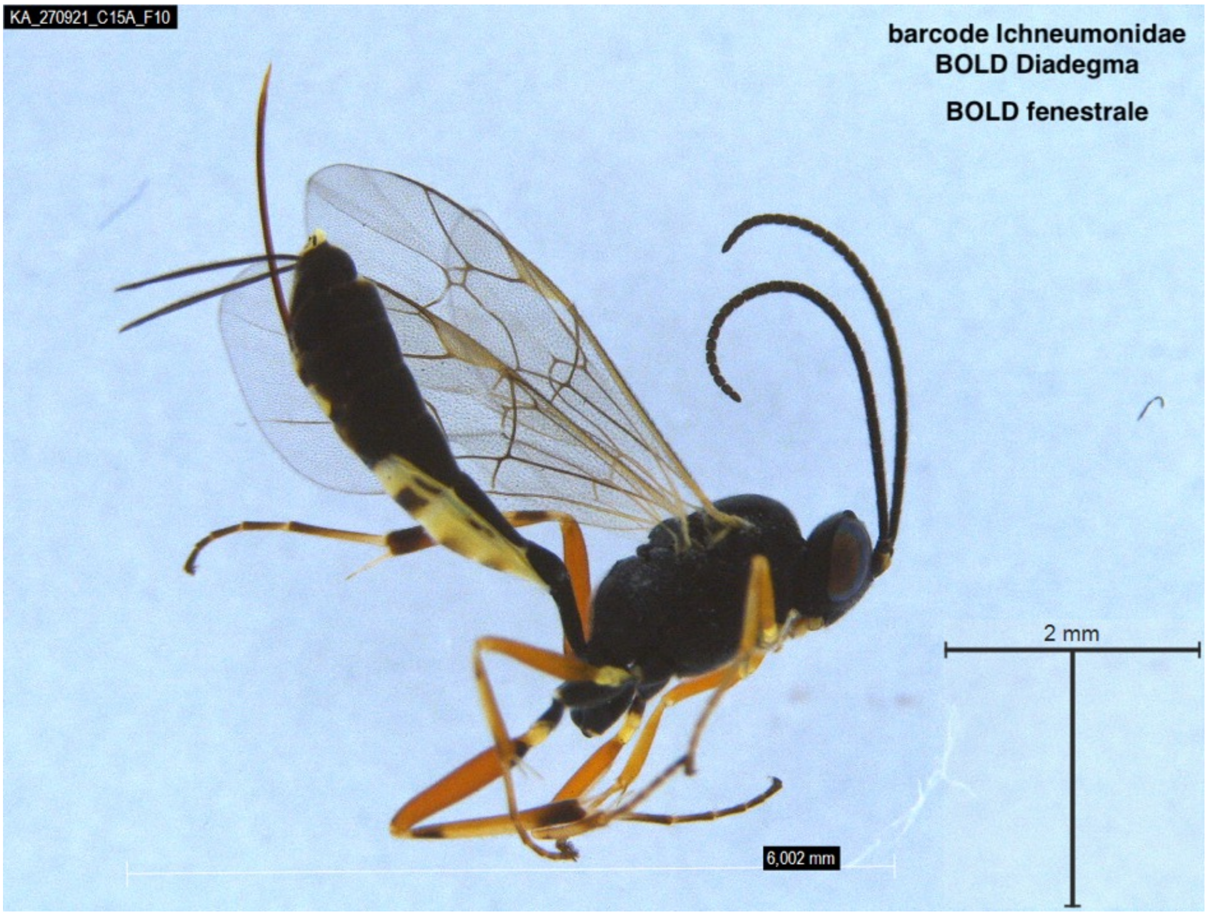

Figure S70: *Diadegma fenestrale*

Diapriidae sp. (Diapriidae)

Sanger_ID: SQ_2022_057_066
Data_ID: HA_240822_C7H (conventional HPS 2000K)

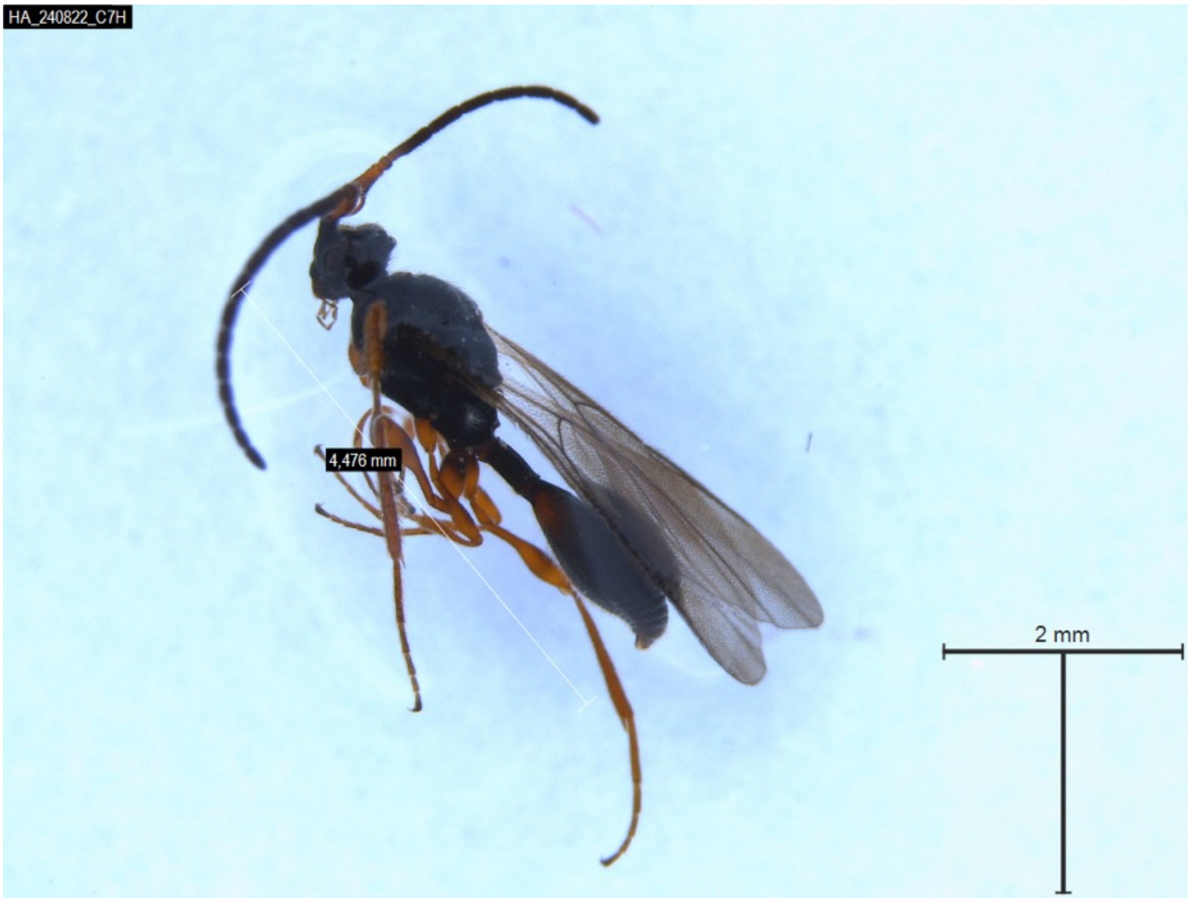

Figure S71: *Diapriidae sp1.*

Sanger-ID: SQ_2022_057_090
Data_ID: KA_260722_C2A (conventional LED 4000K)

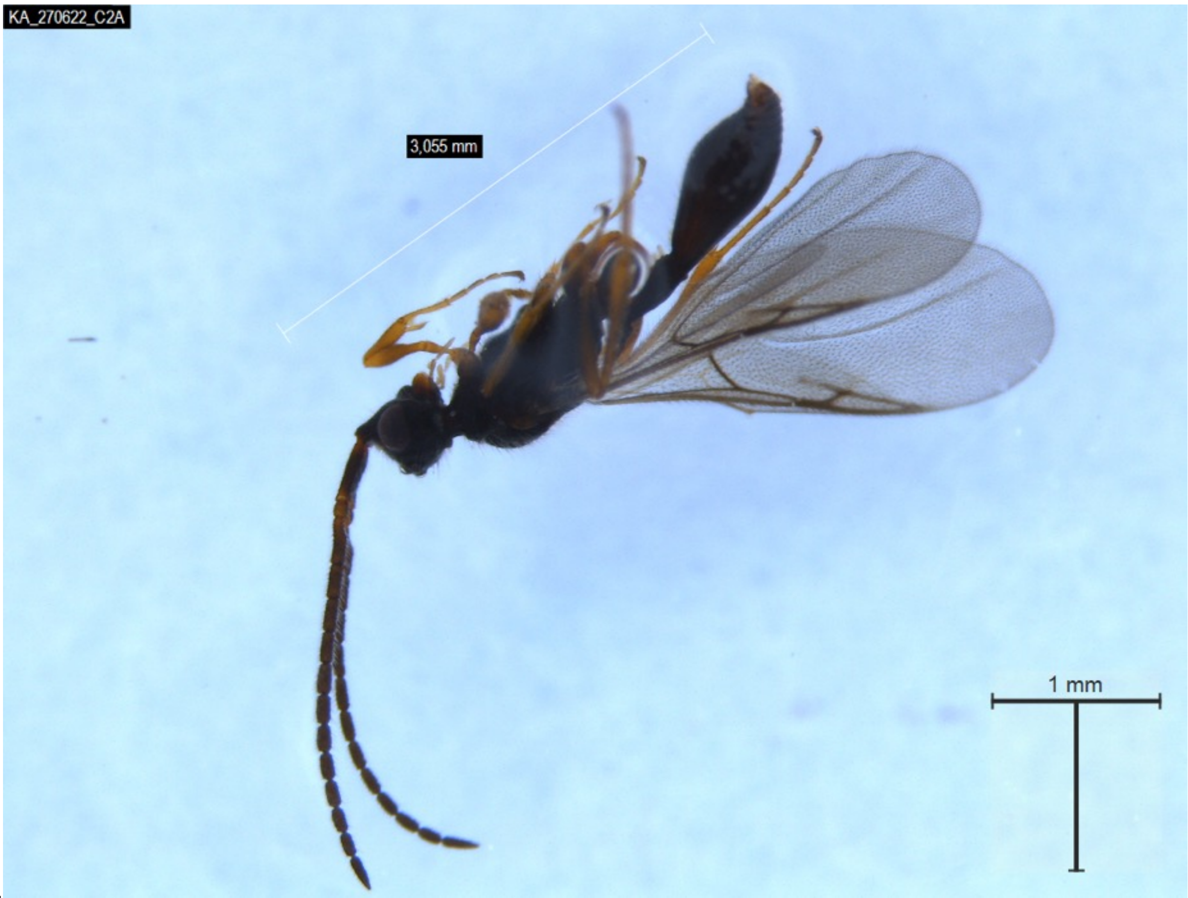

.

Figure S72: Diapriidae sp1.

Sanger_ID: SQ_2022_057_031
Data_ID: HA_240822_C7I (Conventional HPS 2000K)

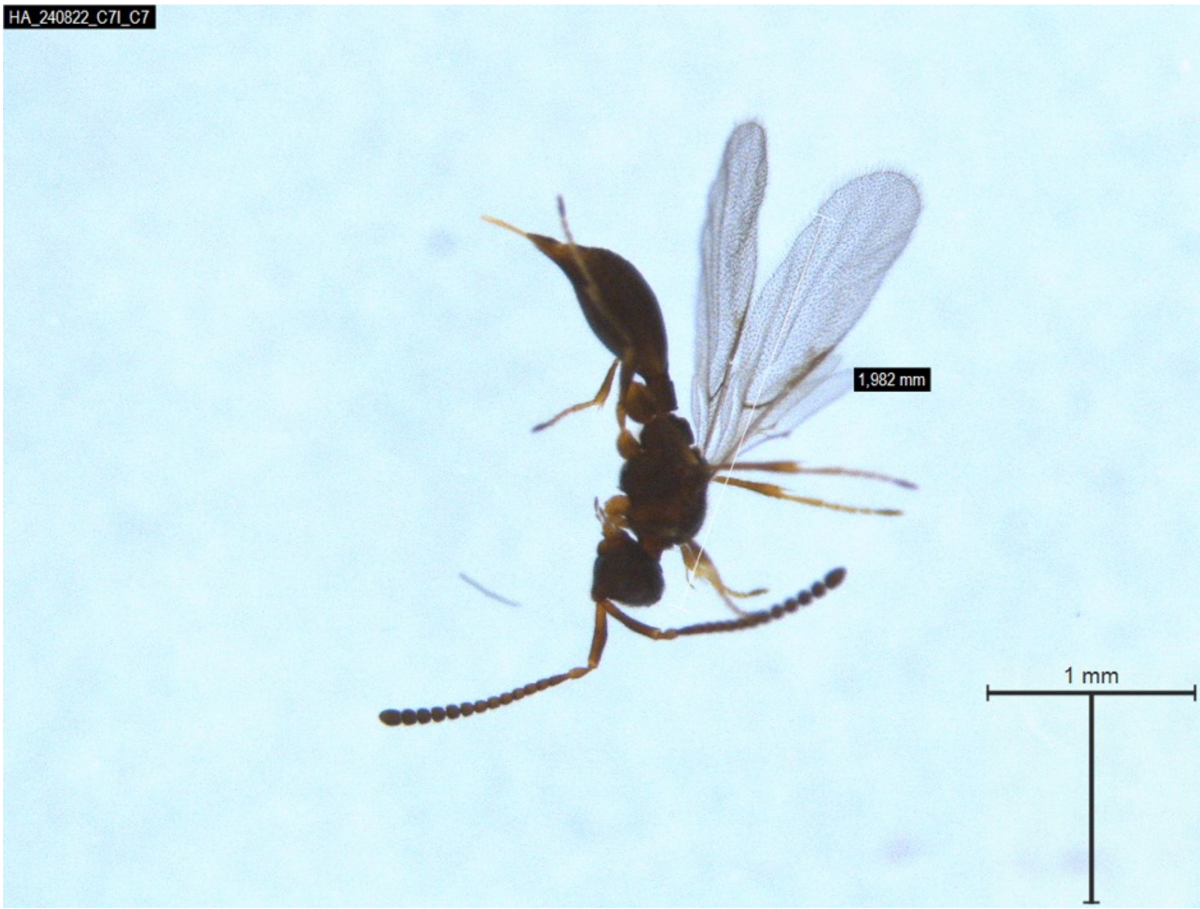

Figure S73: *Diapriidae sp2.*

Sanger_ID: SQ_2022_057_019
Data_ID: BR_160822_C21B (conventional HPS 2000K)

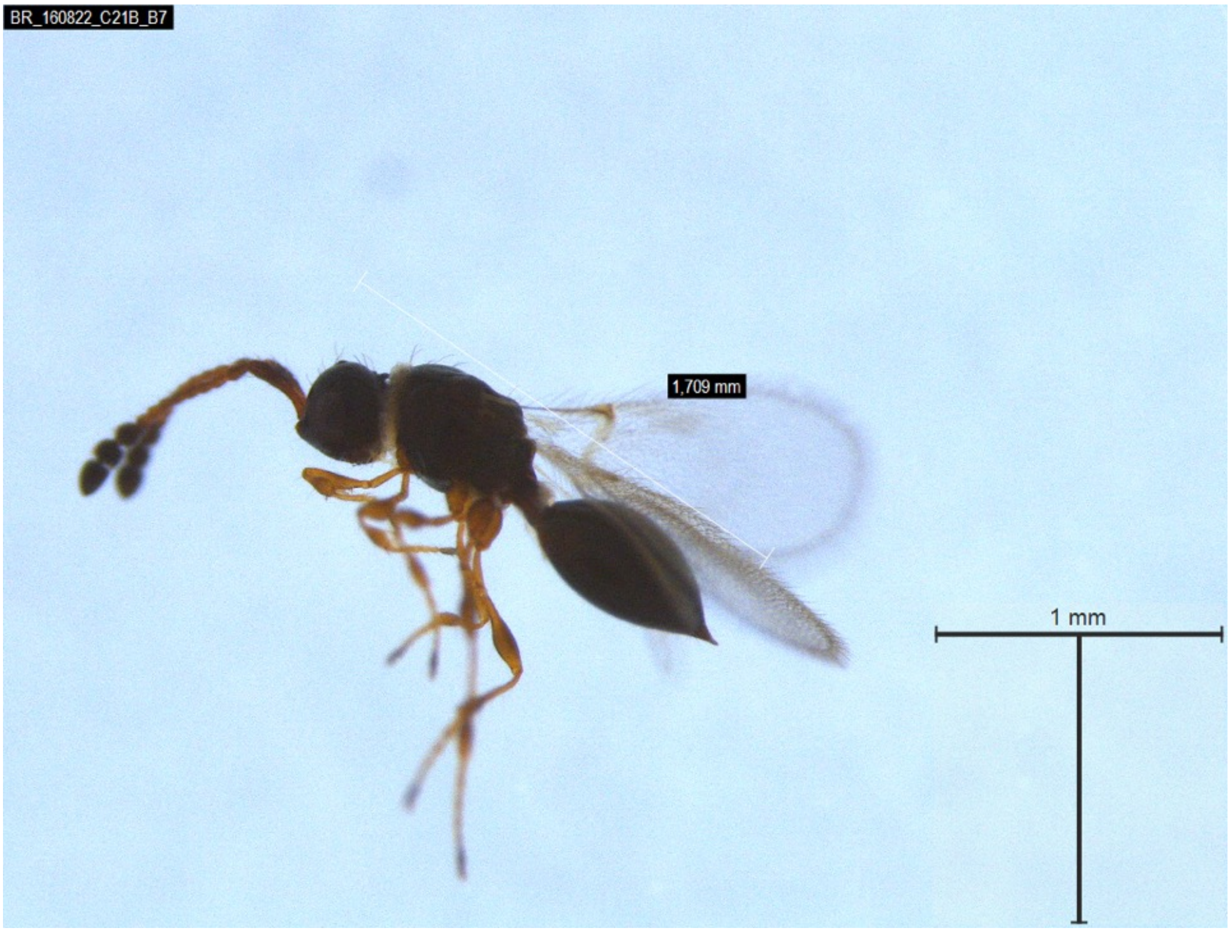

Figure S74: *Diapriidae sp2. (cf Trichopria)*

Sanger_ID: SQ_2022_057_046
Data_ID: KA_270921_C9A (conventional LED 4000K)

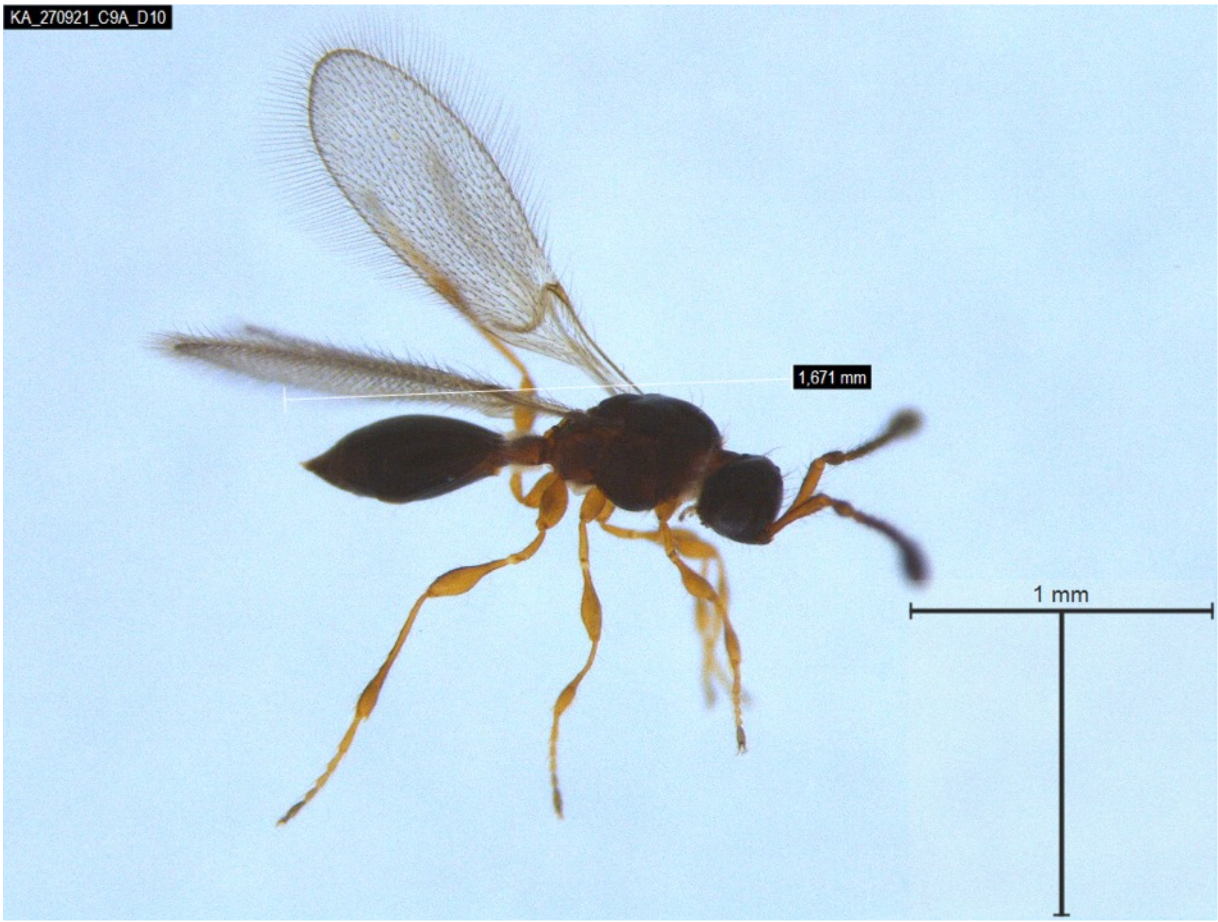

Figure S75: *Diapriidae sp2. (cf Trichopria)*

Sanger_ID: SQ_2022_057_078

Data_ID: KA_270622_C15B (conventional LED 4000K)

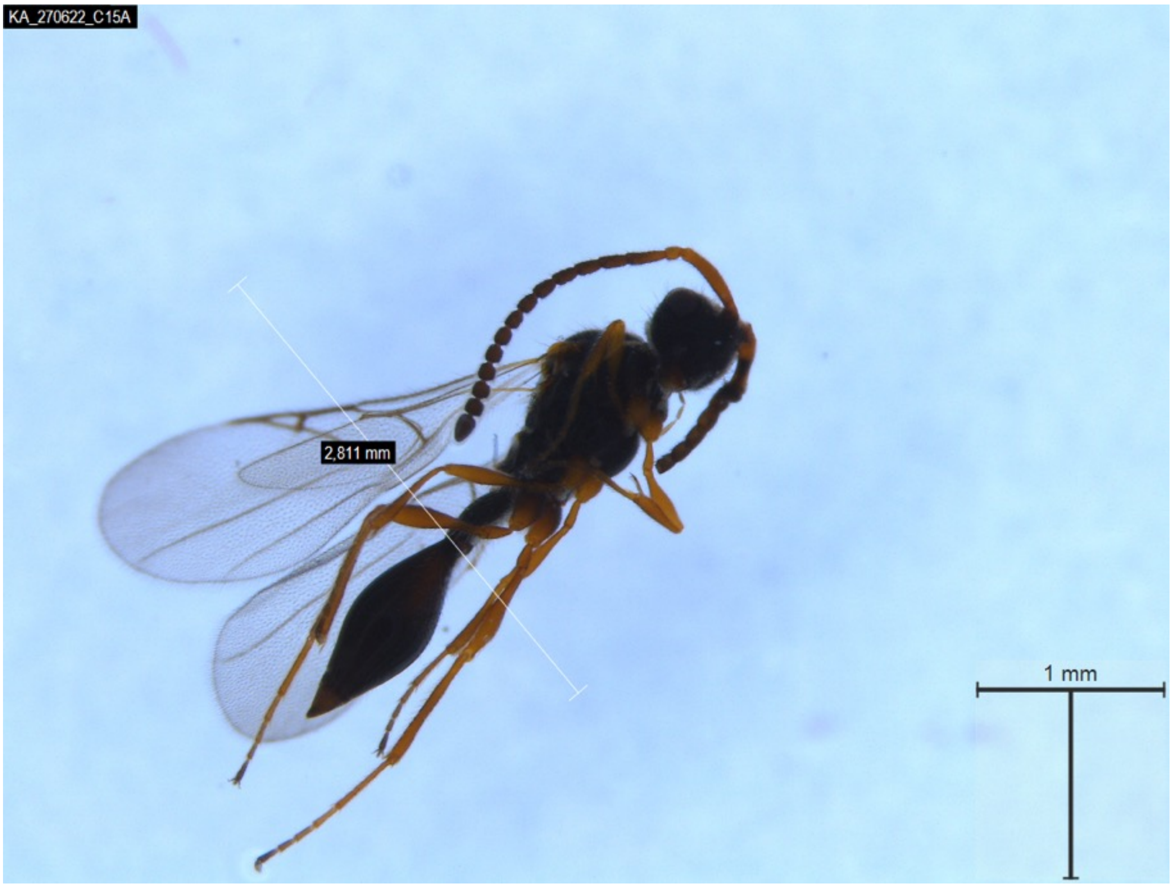

Figure S76: *Diapriidae sp3.*

Diglyphus isaea (Eulophidae)

Sanger_ID: SQ_2022_057_002
Data_ID: HA_010622_C5A (conventional HPS 2000K)

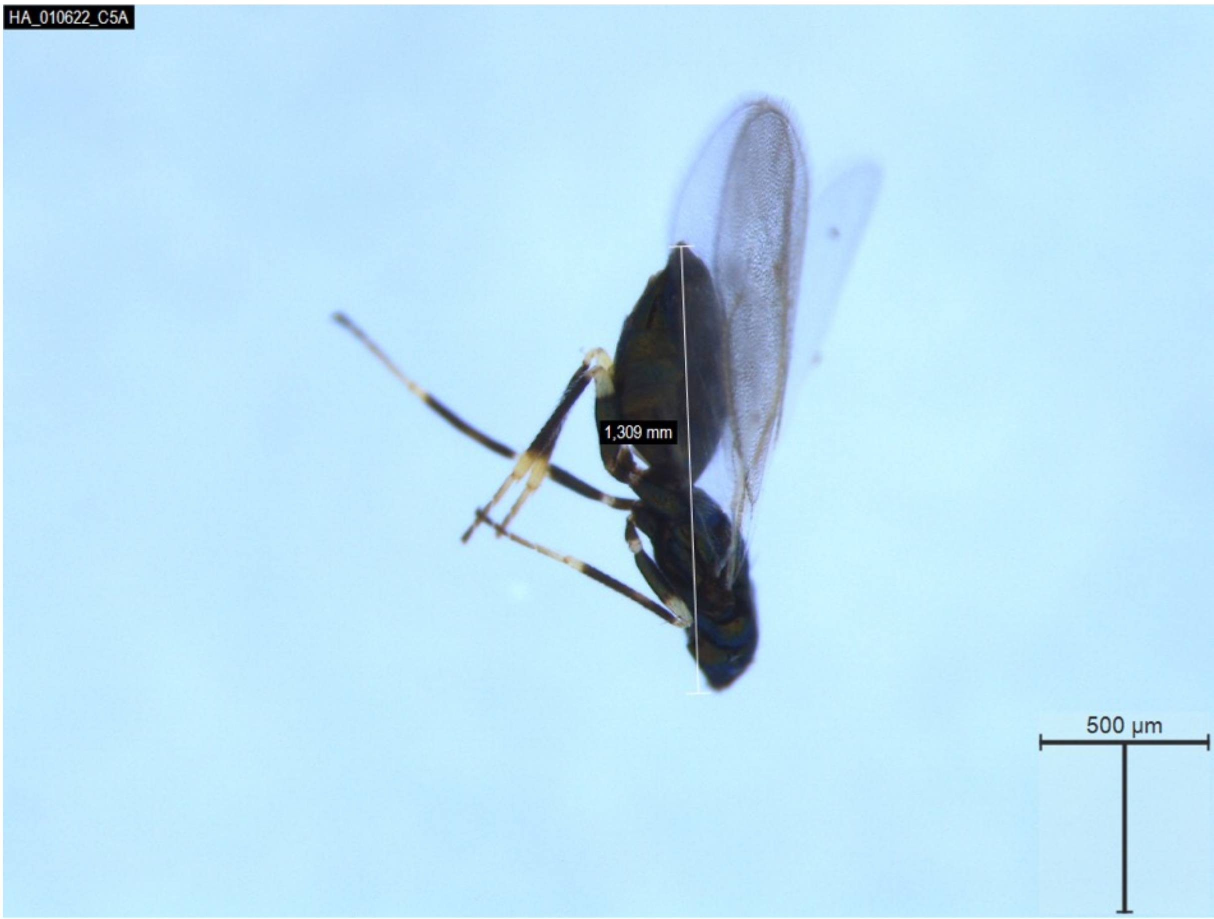

Figure S77: *Diglyphus isaea*

Dinotrema sp. (Braconidae)

GENUS | ACCEPTED

Dinotrema Förster, 1862

Published in: Foerster, Arnold. 1862. Synopsis der Familien und Gattungen der Braconen. Verhandlungen des naturhistorischen Vereines der preussischen Rheinlande und Westphalens 19: 225-288; pl.3.
source: Taxapad Ichneumonoidea

20,775 OCCURRENCES 354 SPECIES

OVERVIEW 4 TREATMENTS METRICS REFERENCE TAXON

851 OCCURRENCES WITH IMAGES

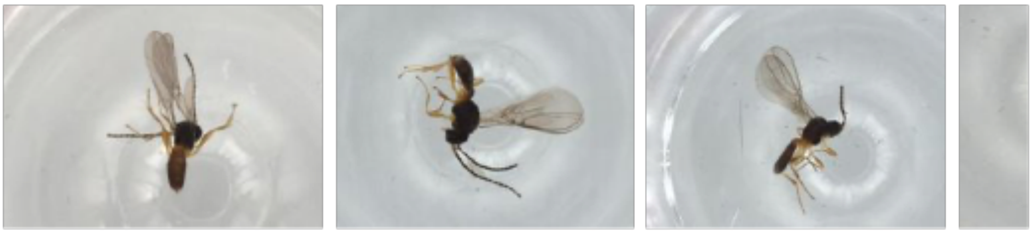

1,797 GEOREFERENCED RECORDS

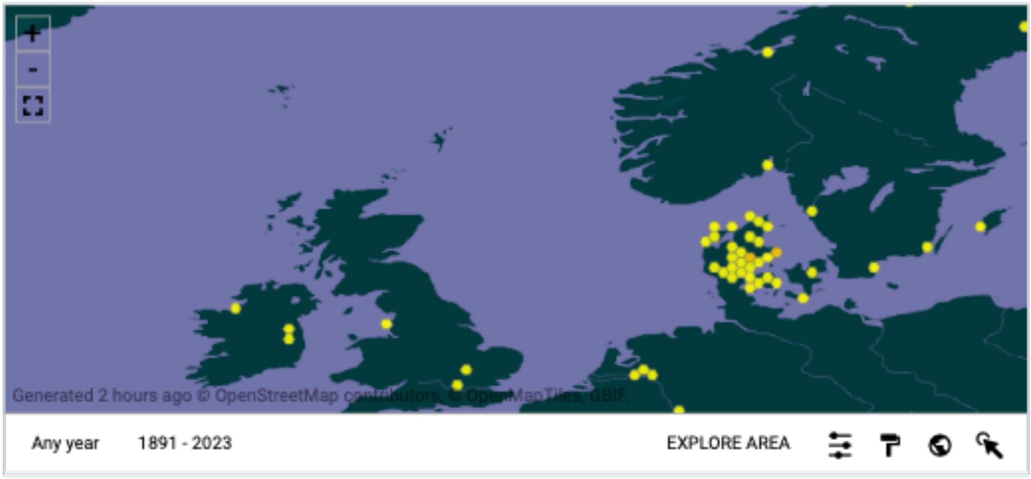

APPEARS IN 35 CHECKLIST DATASETS:

GBIF Backbone Taxonomy
As *Dinotrema* Förster, 1862

Catalogue of Life Checklist
As *Dinotrema*

Figure S78: Global Biodiversity Information Facility (GBIF) Webpage *Dinotrema*

Sanger_ID: SQ_2022_057_024
Data_ID: HA_290622_C8B (conventional HPS 2000K)

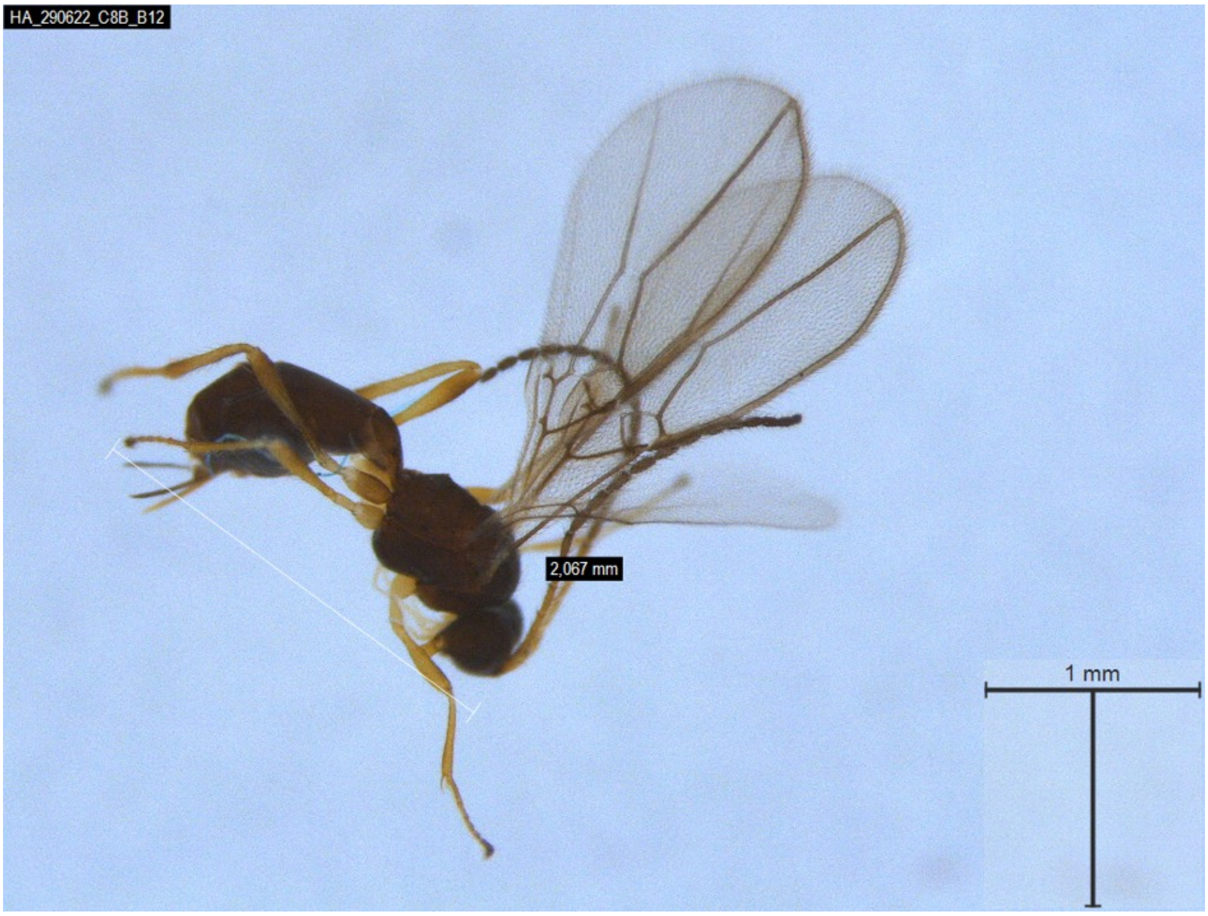

Figure S79: *Dinotrema sp.*

Sanger_ID: SQ_2022_057_092
Data_ID: KA_190722_C17A (conventional LED 4000K)

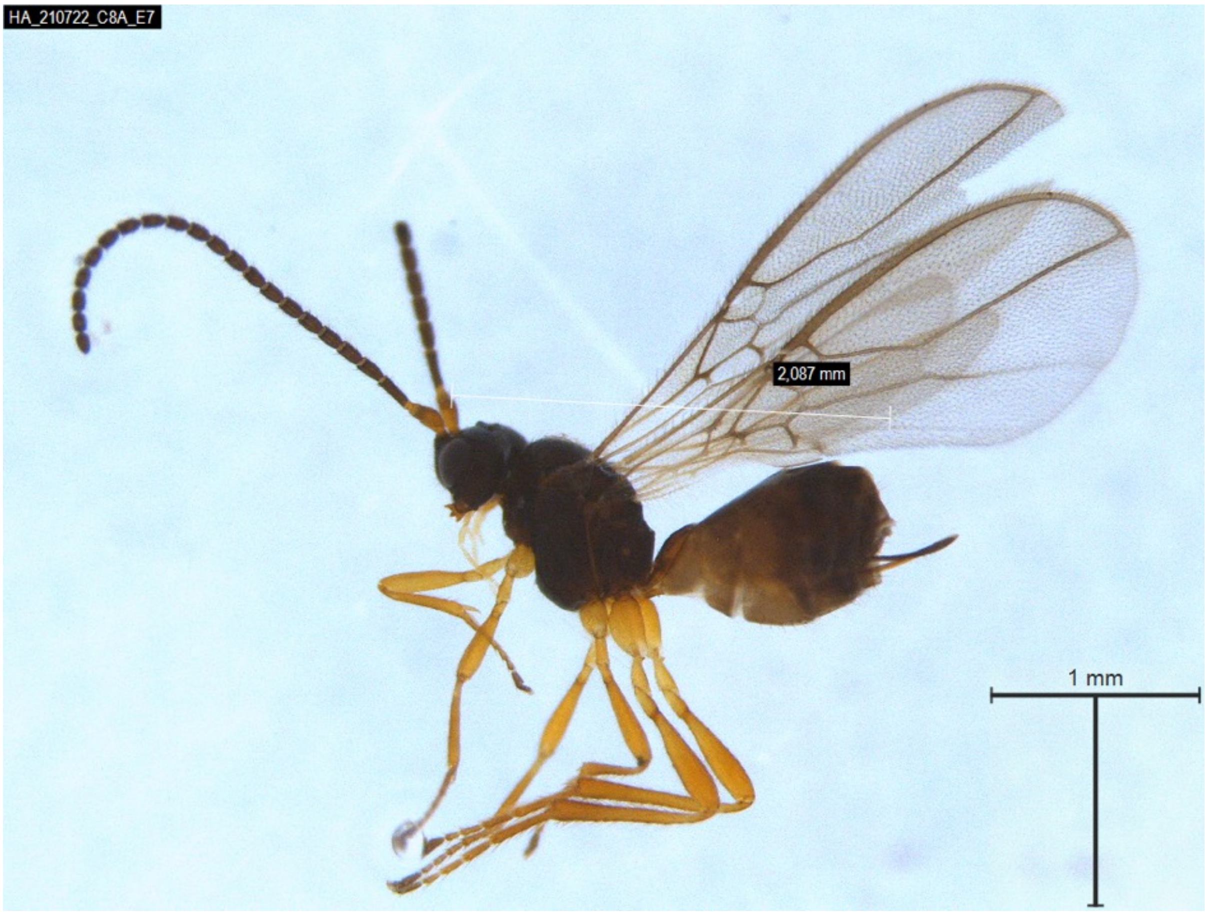

Figure S80: *Dinotrema sp.*

Sanger_ID: SQ_2022_057_055
Data_ID: HA_210722_C8A (conventional HPS 2000K)

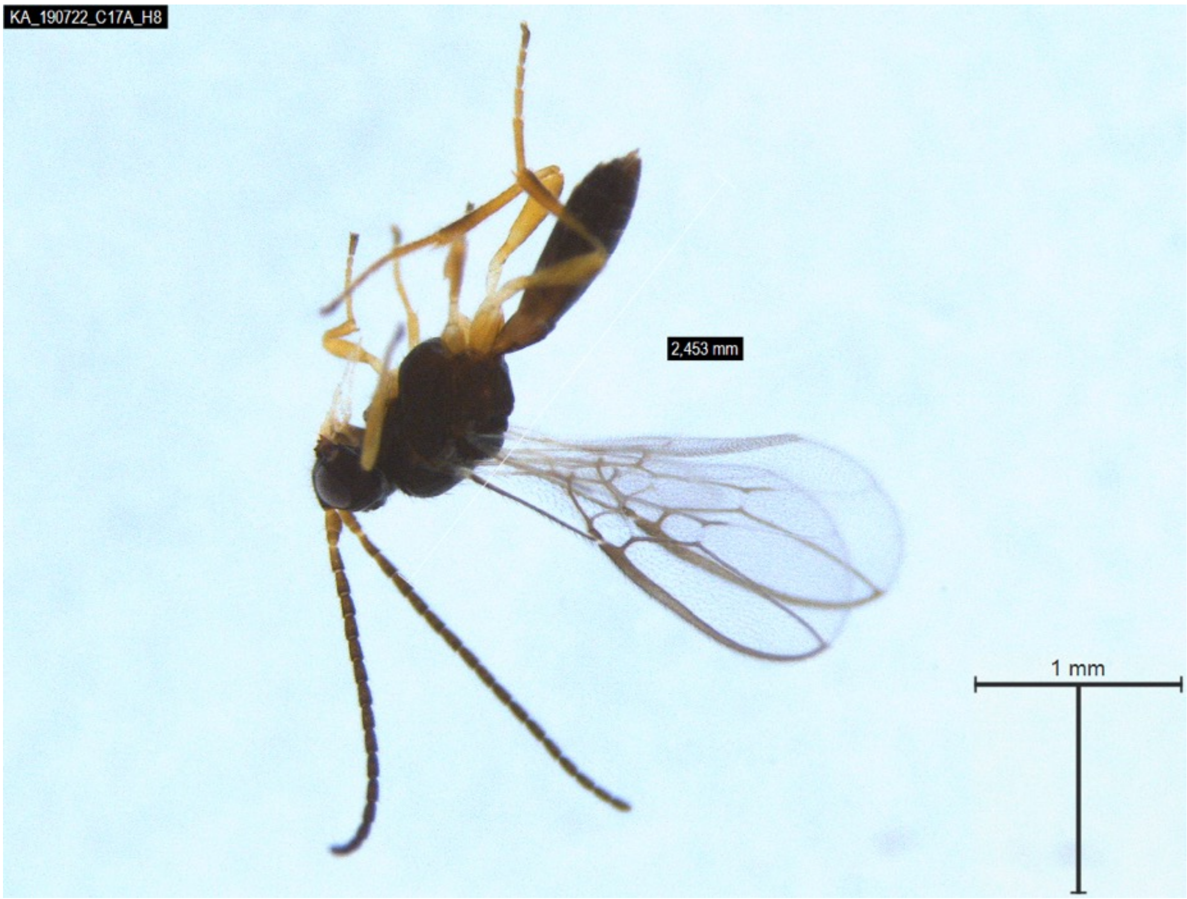

Figure S81: *Dinotrema sp.*

Dolichogenidea sp. (Braconidae)

GENUS | ACCEPTED

Dolichogenidea Viereck, 1911

Published in: Viereck, H. L. (1911). Descriptions of six new genera and thirty-one new species of ichneumon flies. *Proceedings of the United States National Museum.* 40(1812): 173-196.
source: Dyntaxa. Svensk taxonomisk databas

7,568 OCCURRENCES 120 SPECIES

OVERVIEW 5 TREATMENTS METRICS REFERENCE TAXON

141 OCCURRENCES WITH IMAGES

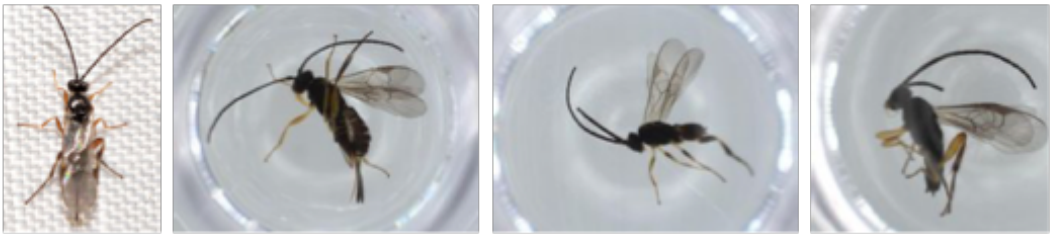

1,692 GEOREFERENCED RECORDS

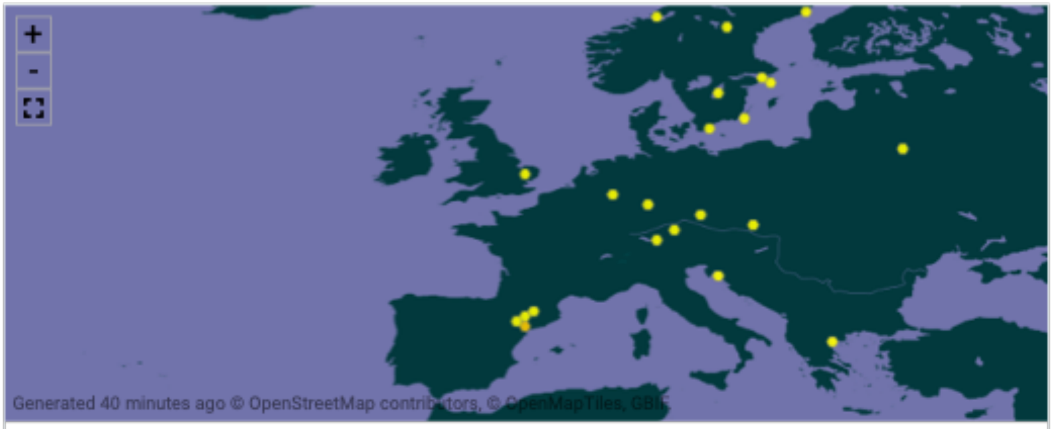

Any year 1919 - 2022 EXPLORE AREA

APPEARS IN 34 CHECKLIST DATASETS:

GBIF Backbone Taxonomy
As *Dolichogenidea* Viereck, 1911

NCBI Taxonomy
As *Dolichogenidea*

Figure S82: Global Biodiversity Information Facility (GBIF) Webpage *Doligochenidea*

Sanger_ID: SQ_2022_057_036
Data_ID: HA_290622_C8C (conventional HPS 2000K)

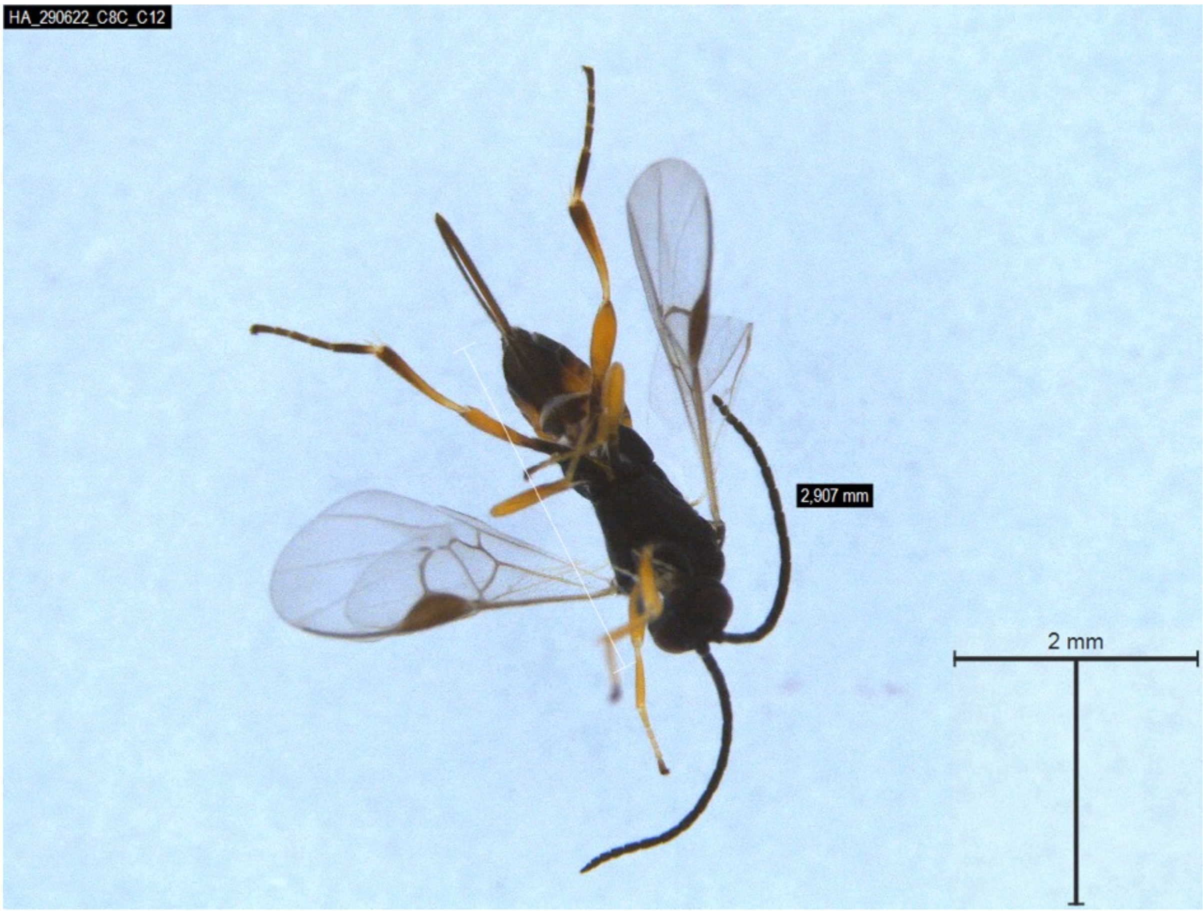

Figure S83: *Doligochenidea sp.*

Encyrtidae sp1. (Encyrtidae)

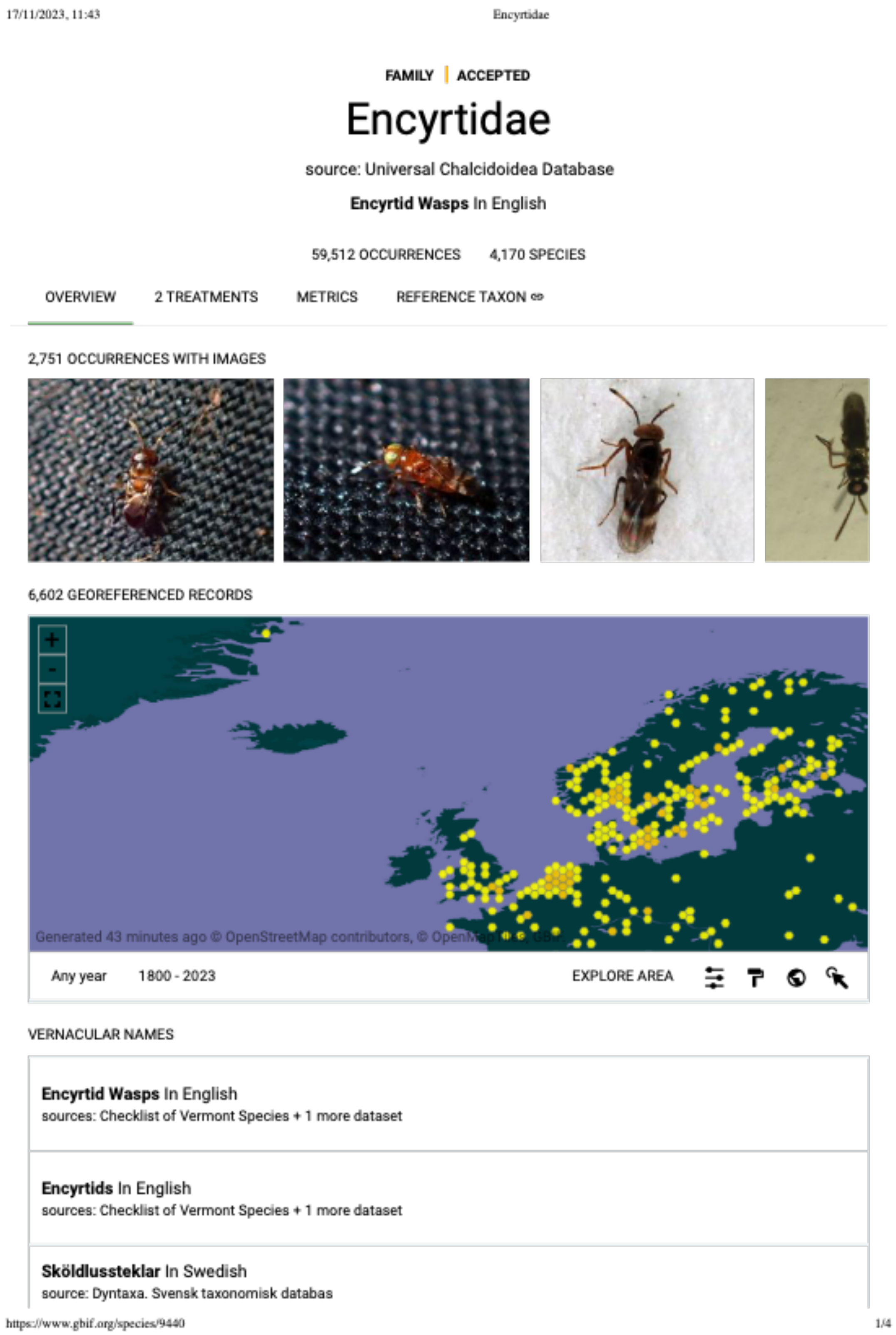

17/11/2023, 11:43 Encyrtidae

FAMILY | ACCEPTED

Encyrtidae

source: Universal Chalcidoidea Database

Encyrtid Wasps In English

59,512 OCCURRENCES 4,170 SPECIES

OVERVIEW 2 TREATMENTS METRICS REFERENCE TAXON

2,751 OCCURRENCES WITH IMAGES

6,602 GEOREFERENCED RECORDS

Generated 43 minutes ago © OpenStreetMap contributors, © OpenMapTiles, GBIF

Any year 1800 - 2023 EXPLORE AREA

VERNACULAR NAMES

Encyrtid Wasps In English
sources: Checklist of Vermont Species + 1 more dataset

Encyrtids In English
sources: Checklist of Vermont Species + 1 more dataset

Sköldlussteklar In Swedish
source: Dyntaxa. Svensk taxonomisk databas

https://www.gbif.org/species/9440 1/4

Figure S84: Global Biodiversity Information Facility (GBIF) Webpage *Encyrtidae*

Sanger_ID: SQ_2022_057_034
Data_ID: KA_270921_C17A (conventional LED 4000K)

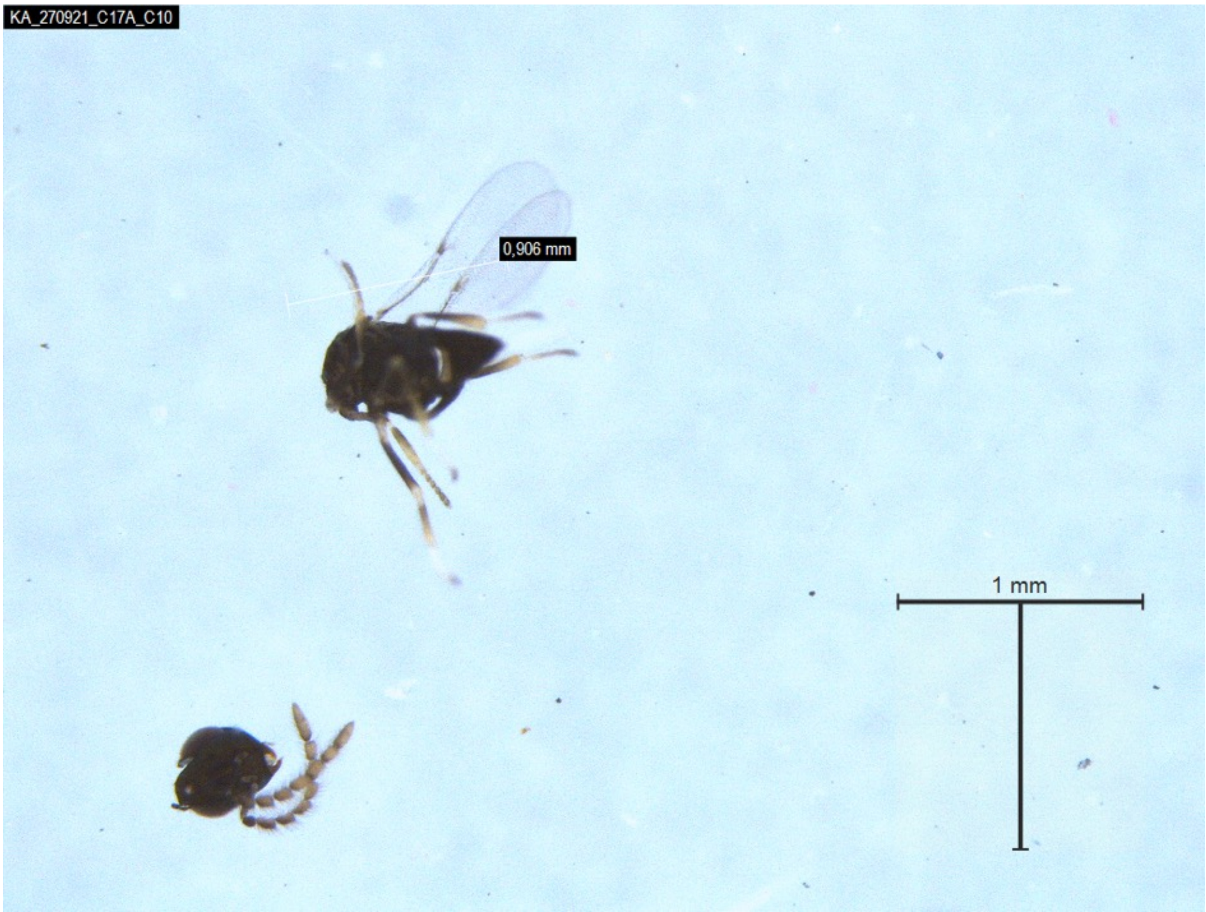

Figure S85: *Encyrtidae sp1*.

Sanger_ID: SQ_2022_057_049
Data_ID: BR_160922_C22A (tailored LED 2700K)

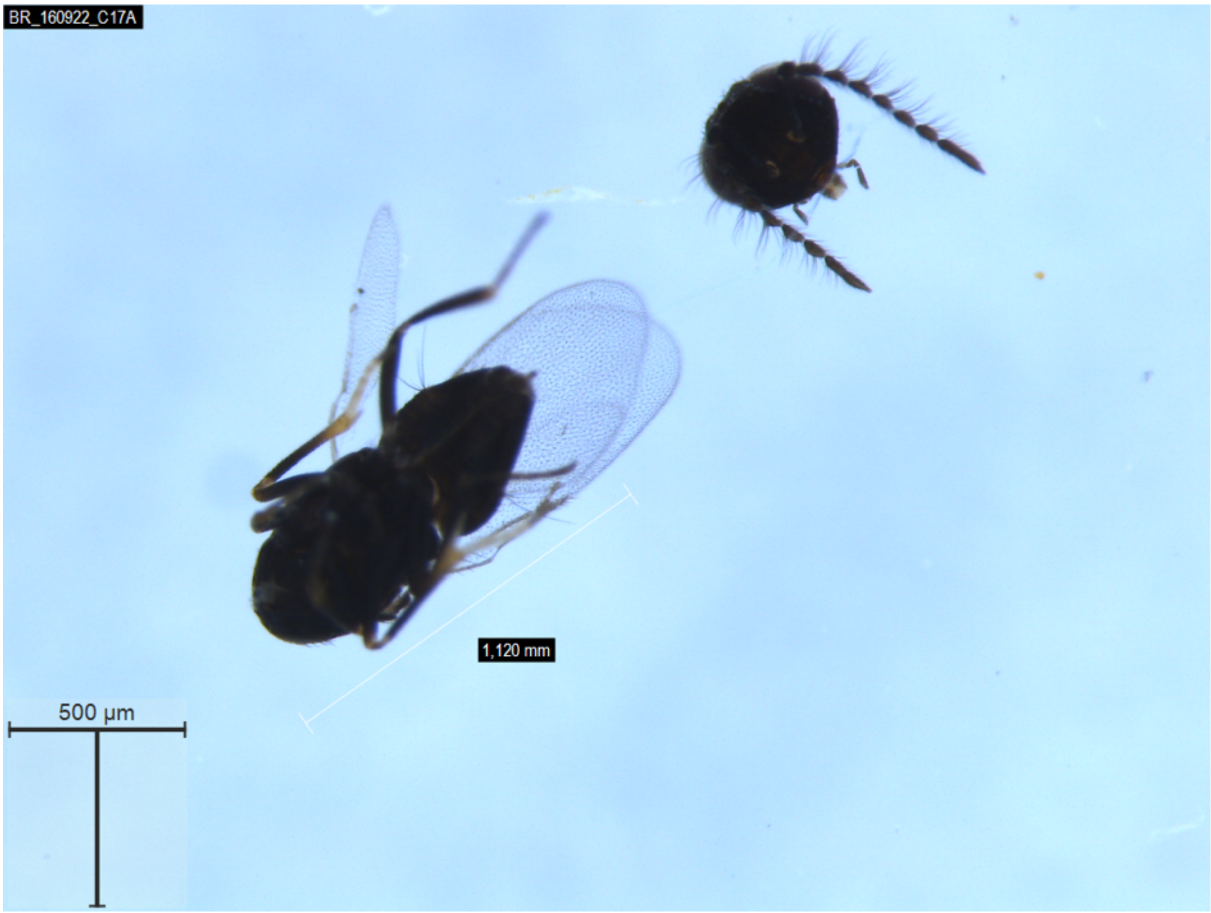

Figure S86: *Encyrtidae sp1. (Picture shows incorrect trap ID)*

Encyrtidae sp2. (Encyrtidae)

Sanger_ID: SQ_2022_057_003
Data_ID: HA_290622_C7B (conventional HPS 2000K)

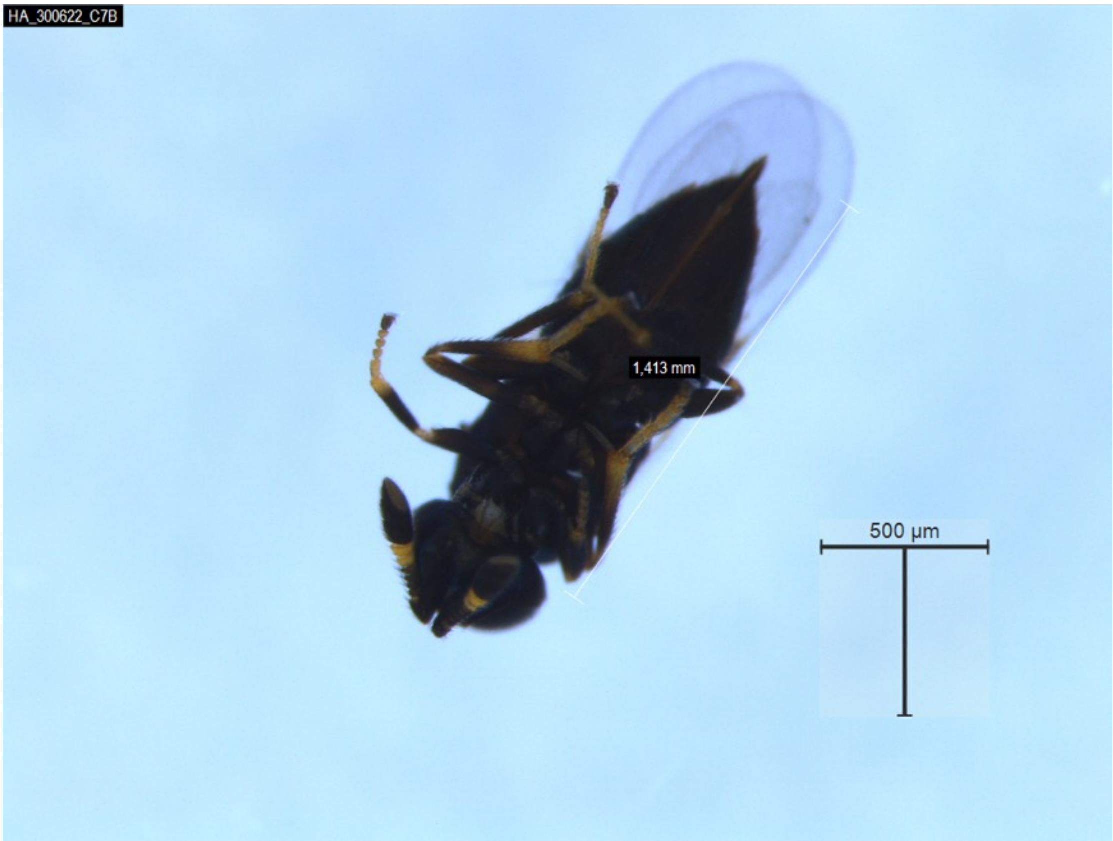

Figure S87: *Encyrtidae sp2*.

Endasys sp. (Ichneumonidae)

GENUS | ACCEPTED

Endasys Förster, 1869

Published in: Verh. Naturh. Ver. Rheinlande, 25 source: Taxapad Ichneumonoidea

996 OCCURRENCES 125 SPECIES

OVERVIEW 1 TREATMENT METRICS REFERENCE TAXON

18 OCCURRENCES WITH IMAGES

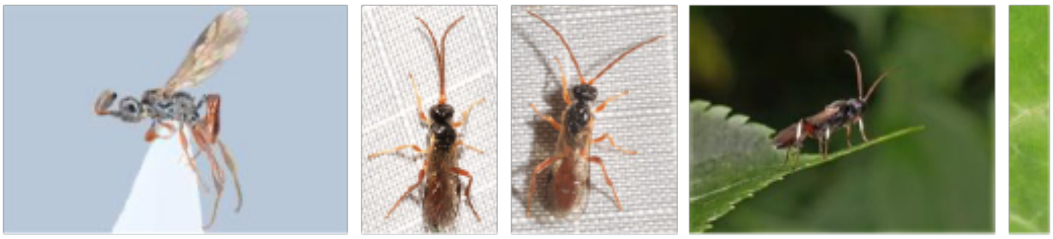

316 GEOREFERENCED RECORDS

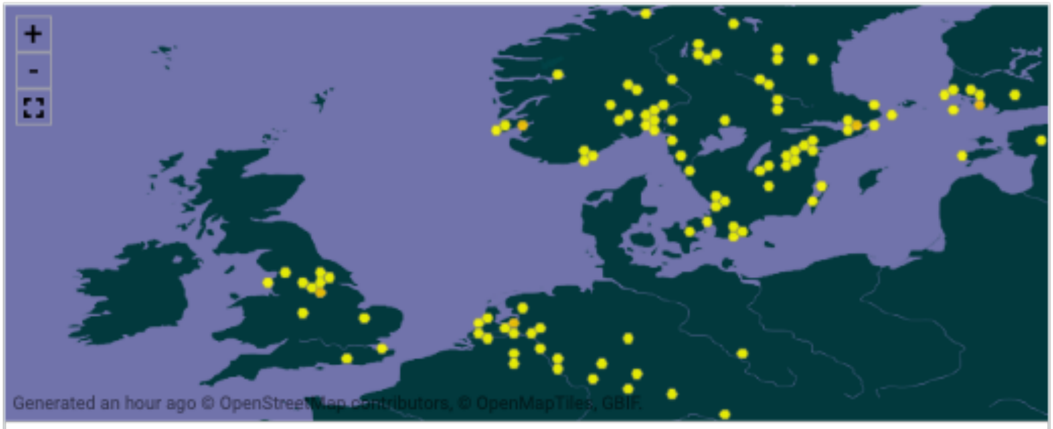

Any year 1887 - 2023 EXPLORE AREA

APPEARS IN 19 CHECKLIST DATASETS:

GBIF Backbone Taxonomy
As *Endasys* Förster, 1869

Catalogue of Life Checklist
As *Endasys*

NCBI Taxonomy
As *Endasys*

Figure S88: Global Biodiversity Information Facility (GBIF) Webpage *Endasys*

Sanger_ID: SQ_2022_057_106
Data_ID: HA_180821_C1A (conventional HPS 2000K)

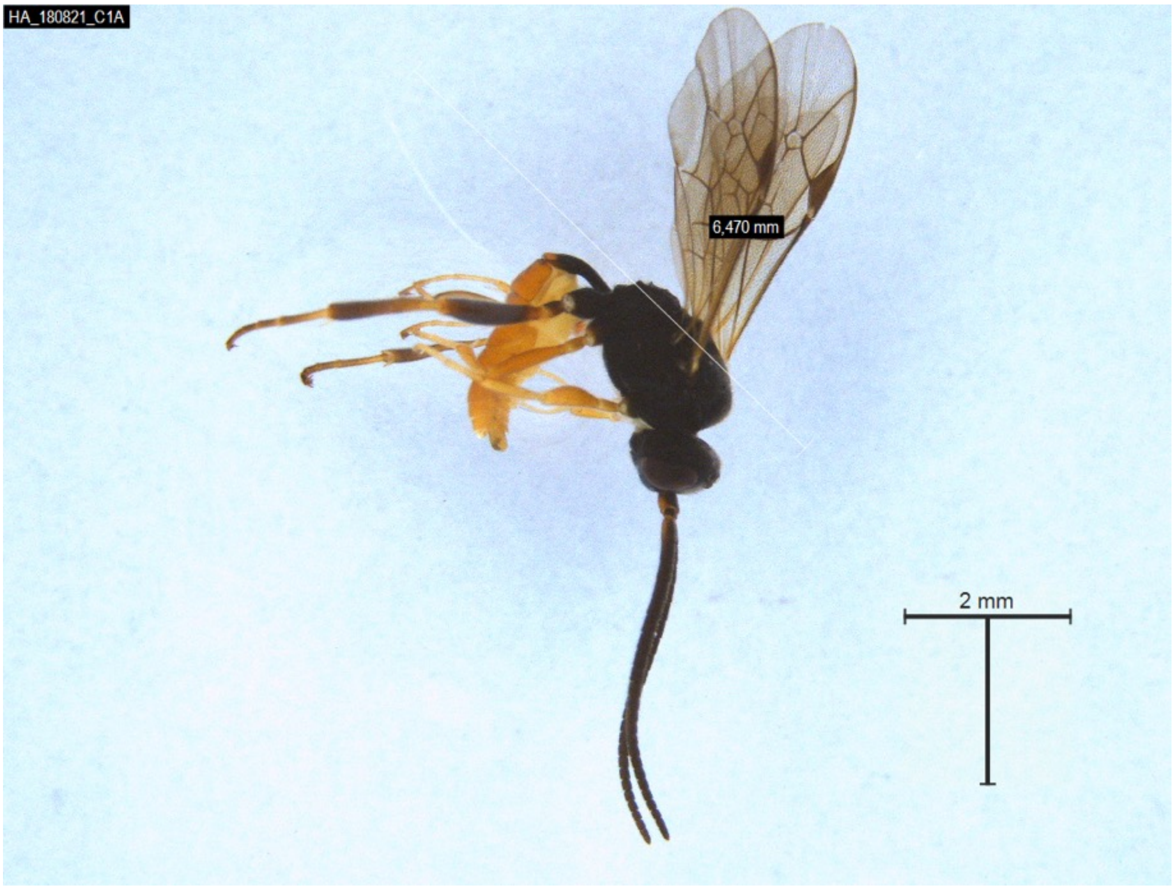

Figure S89: *Endasys sp.*

Eubazus sp. (Braconidae)

GENUS | ACCEPTED

Eubazus Nees, 1814

Published in: Nees von Esenbeck, C. G. 1812. Ichneumonides adsciti, in genera et familias divisi. Magazin für die neuesten Entdeckungen in der gesammten Naturkunde 6(3): 183-221; pl. iv.
source: Taxapad Ichneumonoidea

1,844 OCCURRENCES 153 SPECIES

OVERVIEW METRICS REFERENCE TAXON

155 OCCURRENCES WITH IMAGES

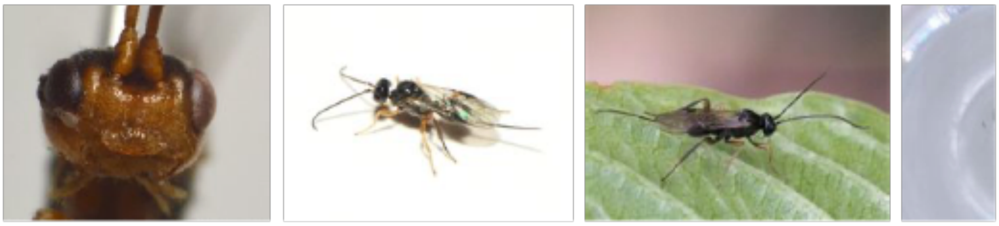

400 GEOREFERENCED RECORDS

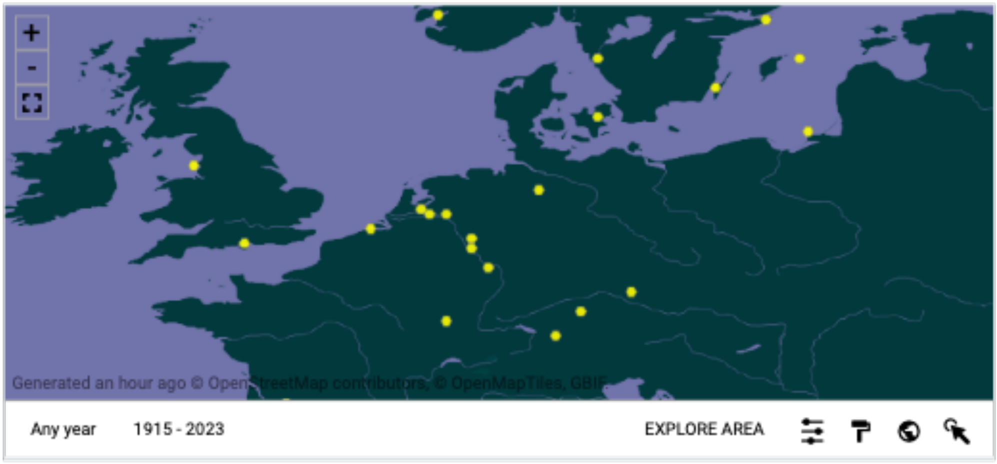

APPEARS IN 22 CHECKLIST DATASETS:

GBIF Backbone Taxonomy
As *Eubazus* Nees, 1814

Catalogue of Life Checklist
As *Eubazus*

Figure S90: Global Biodiversity Information Facility (GBIF) Webpage *Eubazus*

Sanger_ID: SQ_2022_057_082
Data_ID: KA_270921_C10A (conventional LED 4000K)

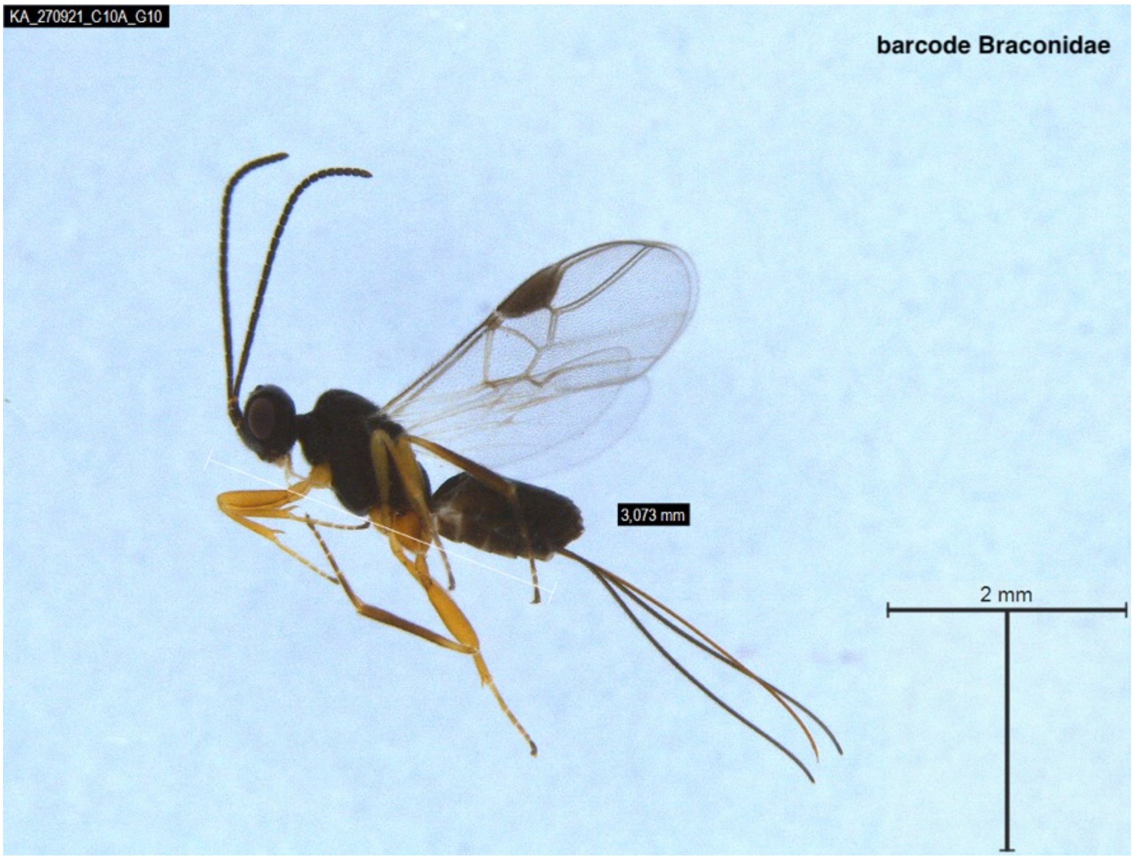

Figure S91: *Eubazus sp.*

Exallonxy nixoni (Proctotrupidae)

SPECIES | ACCEPTED

Exallonyx nixoni Townes, 1981

source: Taxon list of Hymenoptera from Germany compiled in the context of the GBOL project

71 OCCURRENCES

OVERVIEW METRICS REFERENCE TAXON

2 OCCURRENCES WITH IMAGES

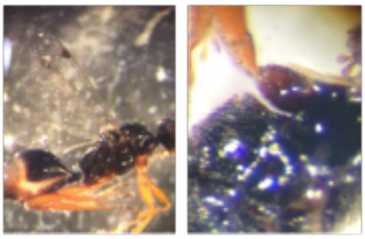

43 GEOREFERENCED RECORDS

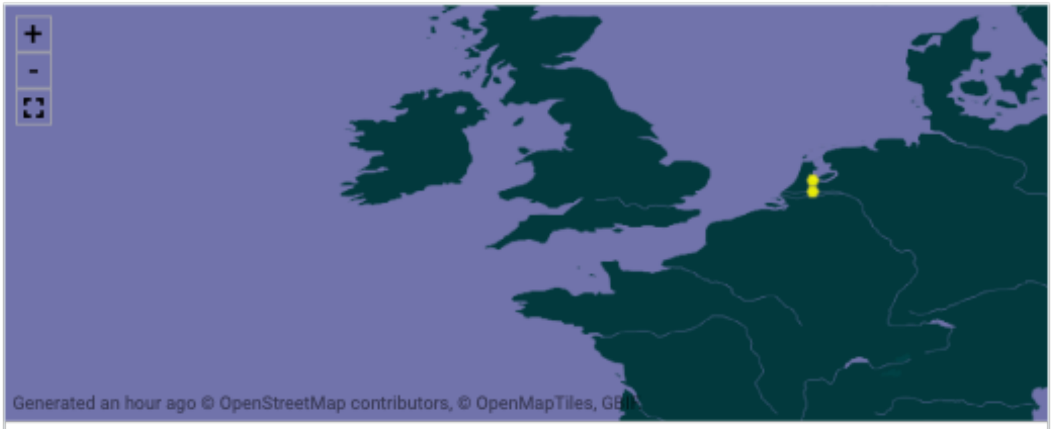

Any year 2008 - 2020 EXPLORE AREA

APPEARS IN 16 CHECKLIST DATASETS:

GBIF Backbone Taxonomy
As *Exallonyx nixoni* Townes, 1981

NCBI Taxonomy
As *Exallonyx nixoni*

The European Nucleotide Archive (ENA) taxonomy
As *Exallonyx nixoni*

Figure S92: Global Biodiversity Information Facility (GBIF) Webpage *Exallonyx nixoni*

Sanger_ID: SQ_2022_057_051
Data_ID: HA_260722_C6A (conventional HPS 2000K)

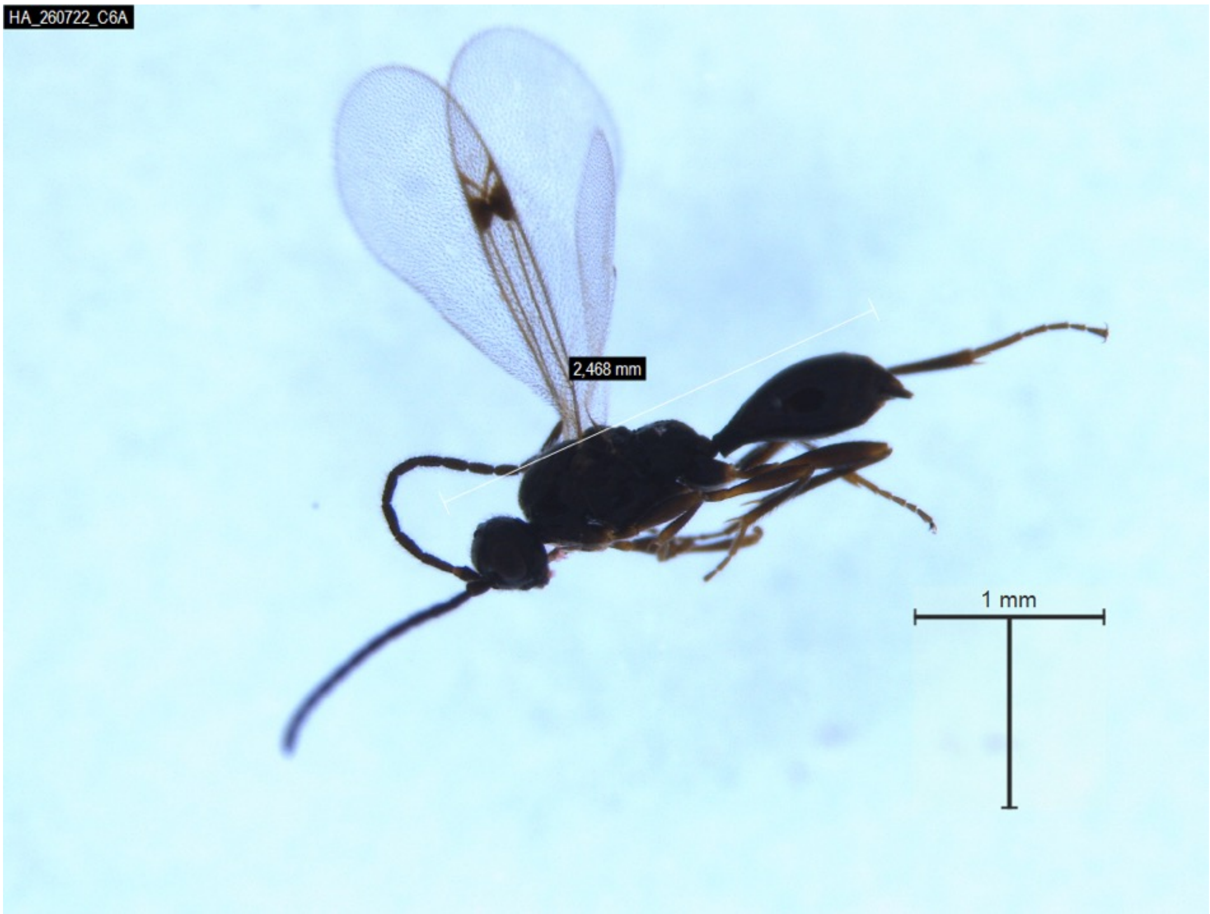

Figure S93: *Exallonxy nixoni*

Exallonxy sp. (Proctotrupidae)

17/11/2023, 12:02 Exallonyx Kieffer, 1904

GENUS | ACCEPTED

Exallonyx Kieffer, 1904

Published in: Bull. Soc. Metz, 23 source: Catalogue of Life Checklist

2,133 OCCURRENCES 26 SPECIES

OVERVIEW 1 TREATMENT METRICS REFERENCE TAXON

39 OCCURRENCES WITH IMAGES

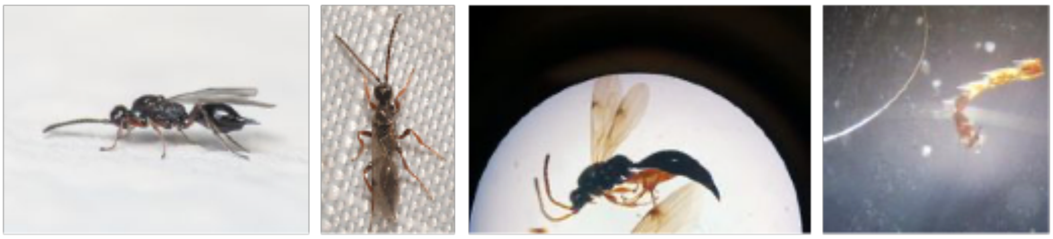

469 GEOREFERENCED RECORDS

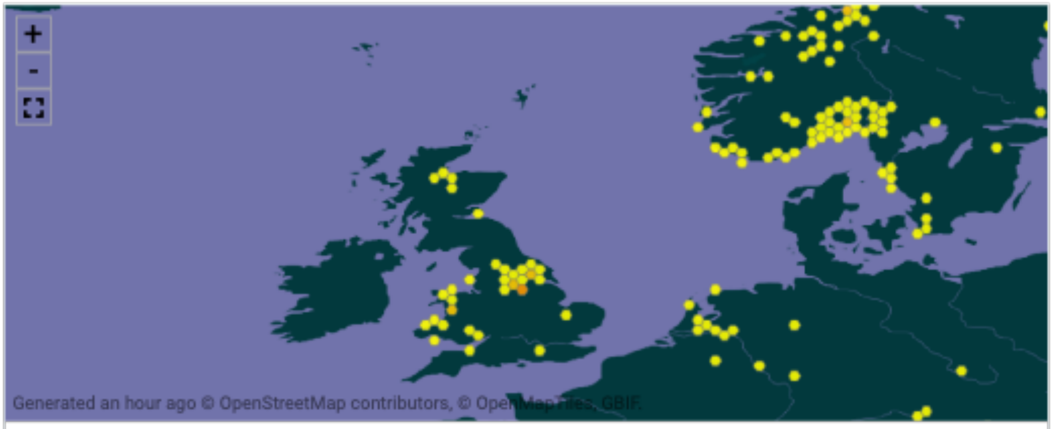

Any year 1800 - 2023 EXPLORE AREA

APPEARS IN 24 CHECKLIST DATASETS:

GBIF Backbone Taxonomy
As *Exallonyx* Kieffer, 1904

Catalogue of Life Checklist
As *Exallonyx*

NCBI Taxonomy
As *Exallonyx*

https://www.gbif.org/species/1248782 1/3

Figure S94: Global Biodiversity Information Facility (GBIF) Webpage *Exallonyx*

Sanger_ID: SQ_2022_057_007

Data_ID: HA_260722_C7A (conventional HPS 2000K)

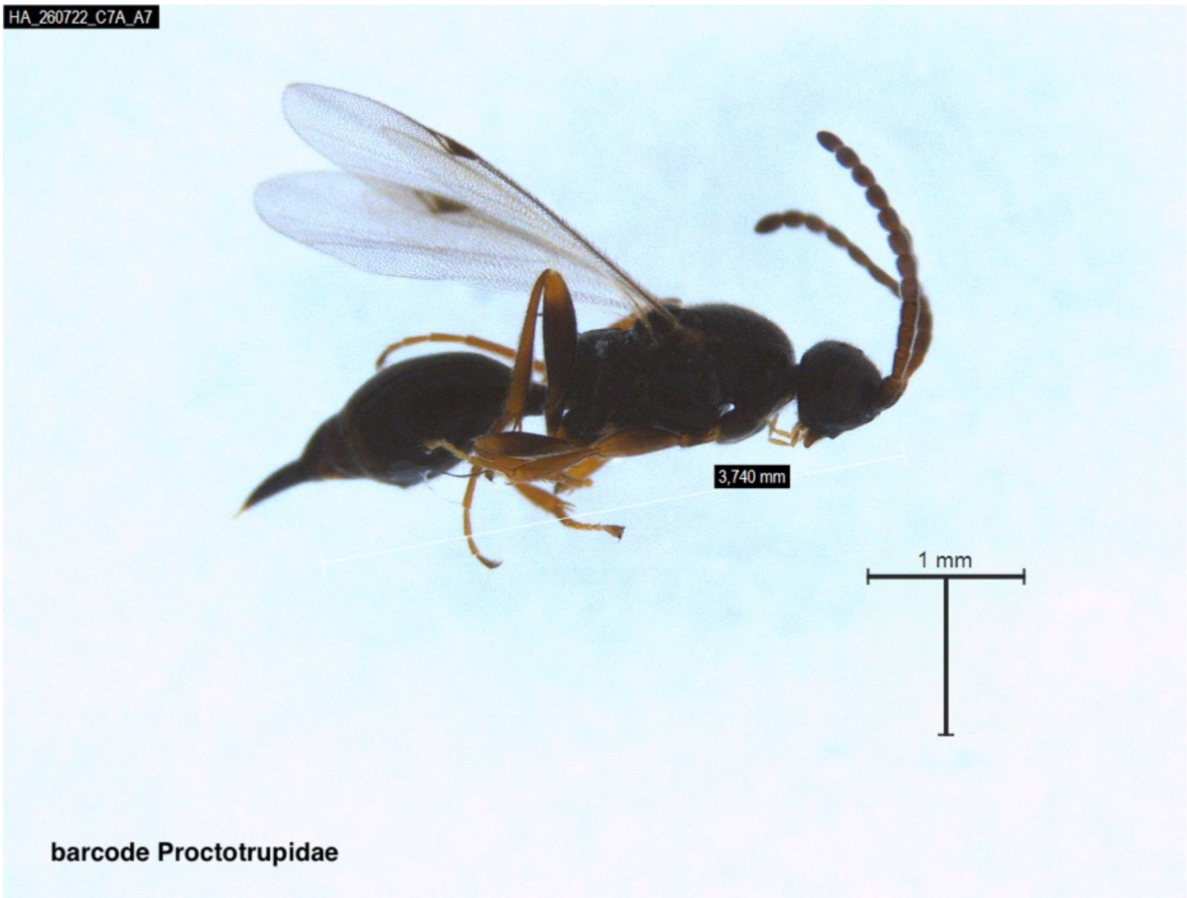

Figure S95: *Exallonyx sp.*

Gastrancistrus sp. (Pirenidae)

GENUS | ACCEPTED

Gastrancistrus Westwood, 1833

Published in: Westwood, John O. 1833. Descriptions of several new British forms amongst the parasitic hymenopterous insects. The London and Edinburgh Philosophical Magazine and Journal of Science, Series 3 2(12): 443-445.
source: Universal Chalcidoidea Database

816 OCCURRENCES 126 SPECIES

OVERVIEW METRICS REFERENCE TAXON

3 OCCURRENCES WITH IMAGES

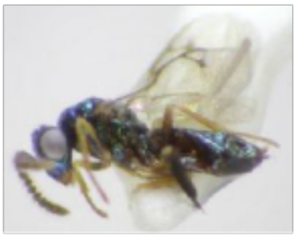

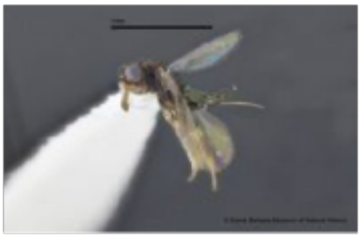

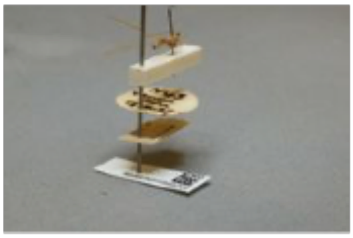

246 GEOREFERENCED RECORDS

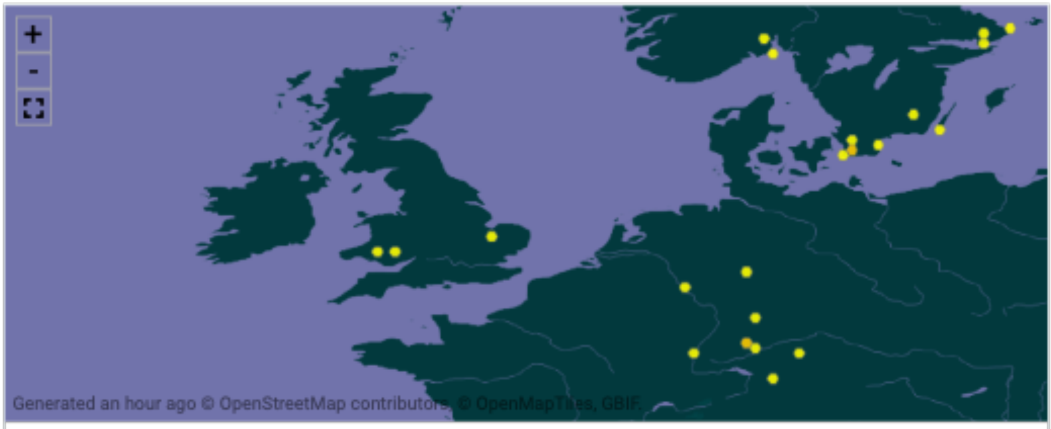

Any year 1869 - 2022 EXPLORE AREA

APPEARS IN 22 CHECKLIST DATASETS:

GBIF Backbone Taxonomy
As *Gastrancistrus* Westwood, 1833

Catalogue of Life Checklist
As *Gastrancistrus*

Figure S96: Global Biodiversity Information Facility (GBIF) Webpage *Gastrancistrus*

Sanger_ID: SQ_2022_057_035
Data_ID: BR_020921_C22B (conventional HPS 2000K)

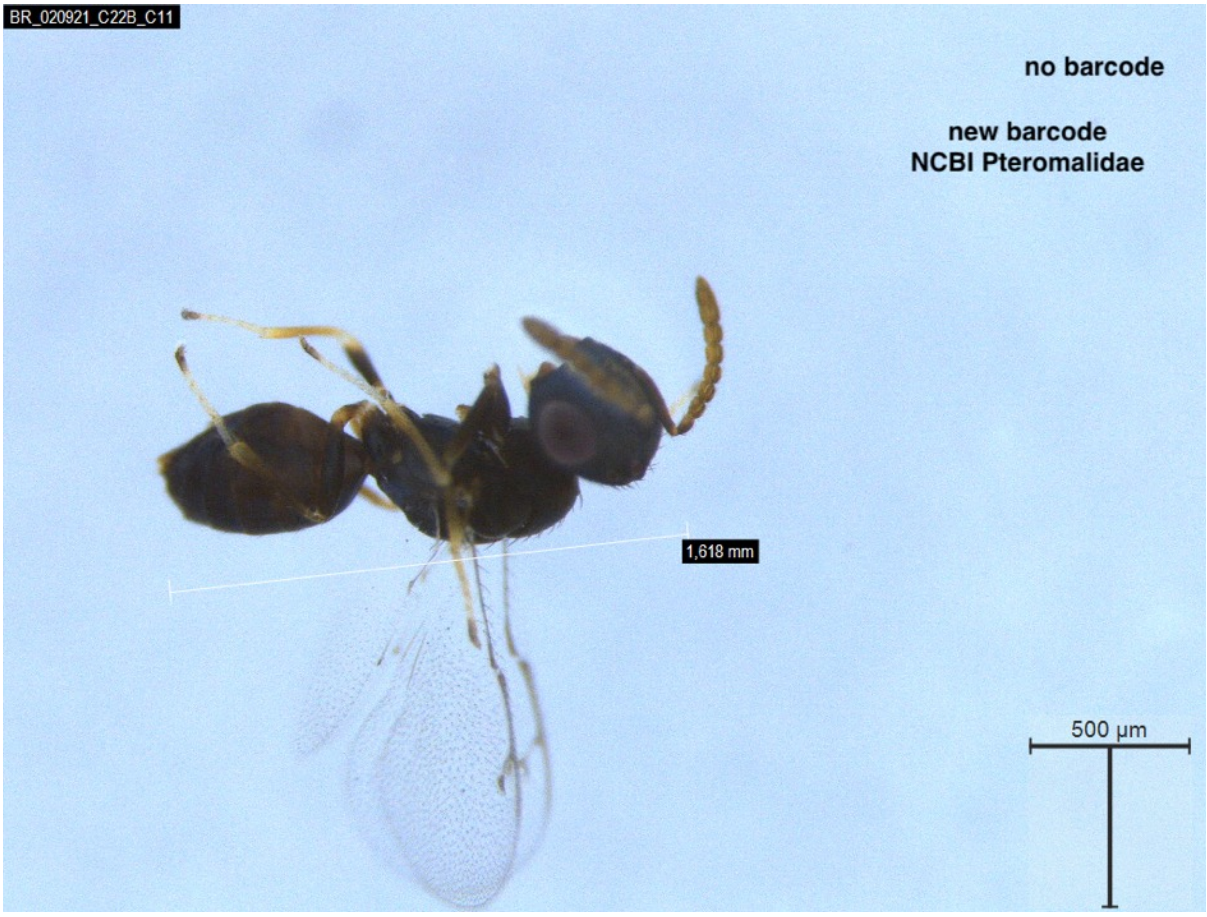

Figure S97: *Gastrancistrus sp.*

Gelis sp. (Ichneumonidae)

GENUS | ACCEPTED

Gelis Thunberg, 1827

Published in: N. Acta Soc. Sci. Upsal., 9 source: Taxapad Ichneumonoidea

8,376 OCCURRENCES 291 SPECIES

OVERVIEW 1 TREATMENT METRICS REFERENCE TAXON

1,777 OCCURRENCES WITH IMAGES

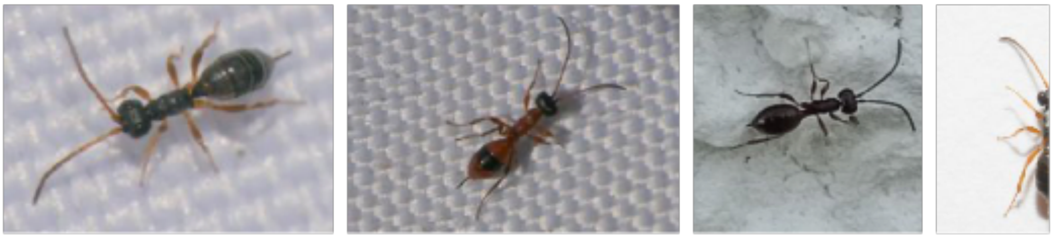

3,587 GEOREFERENCED RECORDS

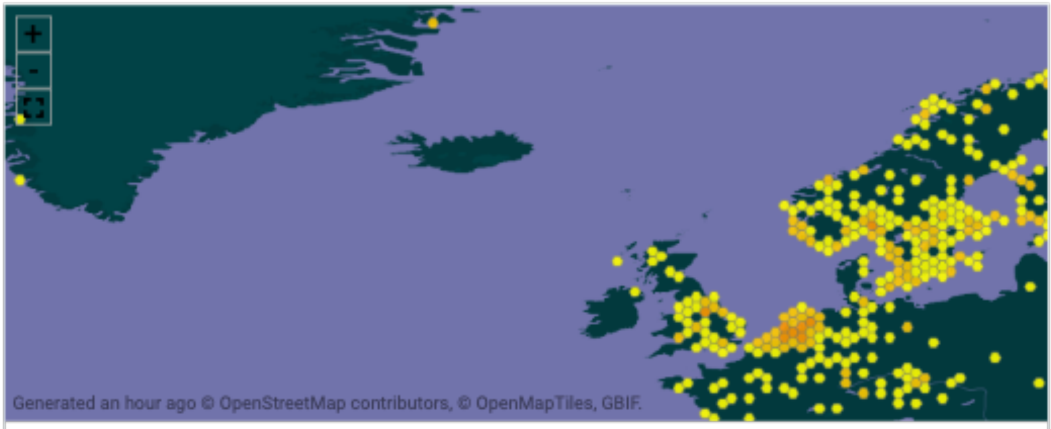

Any year 1758 - 2023 EXPLORE AREA

APPEARS IN 29 CHECKLIST DATASETS:

GBIF Backbone Taxonomy
As *Gelis* Thunberg, 1827

Catalogue of Life Checklist
As *Gelis*

NCBI Taxonomy
As *Gelis*

Figure S98: Global Biodiversity Information Facility (GBIF) Webpage *Gelis*

Sanger_ID: SQ_2022_057_009
Data_ID: HA_040821_C2A (conventional HPS 2000K)

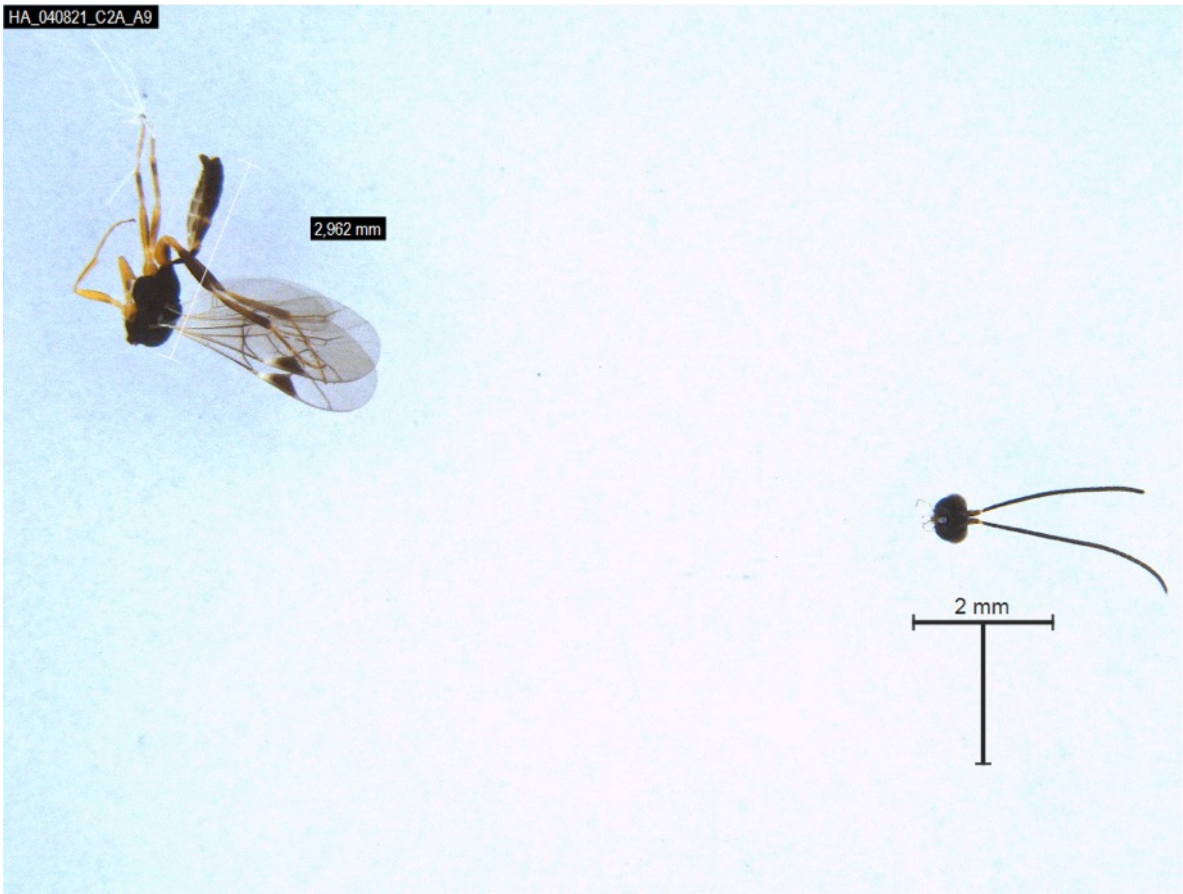

Figure S99: *Gelis sp.*

Sanger_ID: SQ_2022_057_011
Data_ID: KA_160821_C18A (conventional LED 4000K)

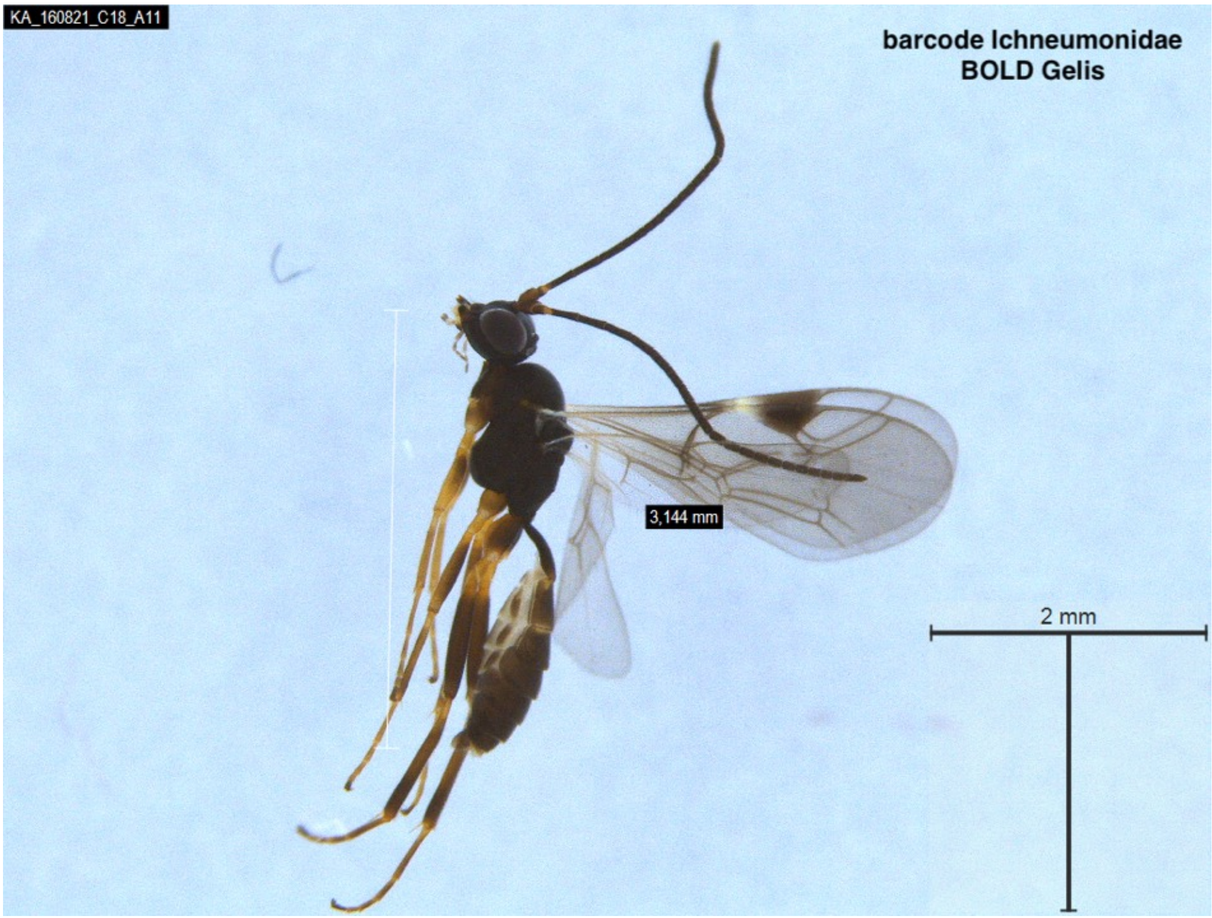

Figure S100: *Gelis sp.*

Sanger_ID: SQ_2022_057_056
Data_ID: KA_150922_C15A (conventional LED 4000K)

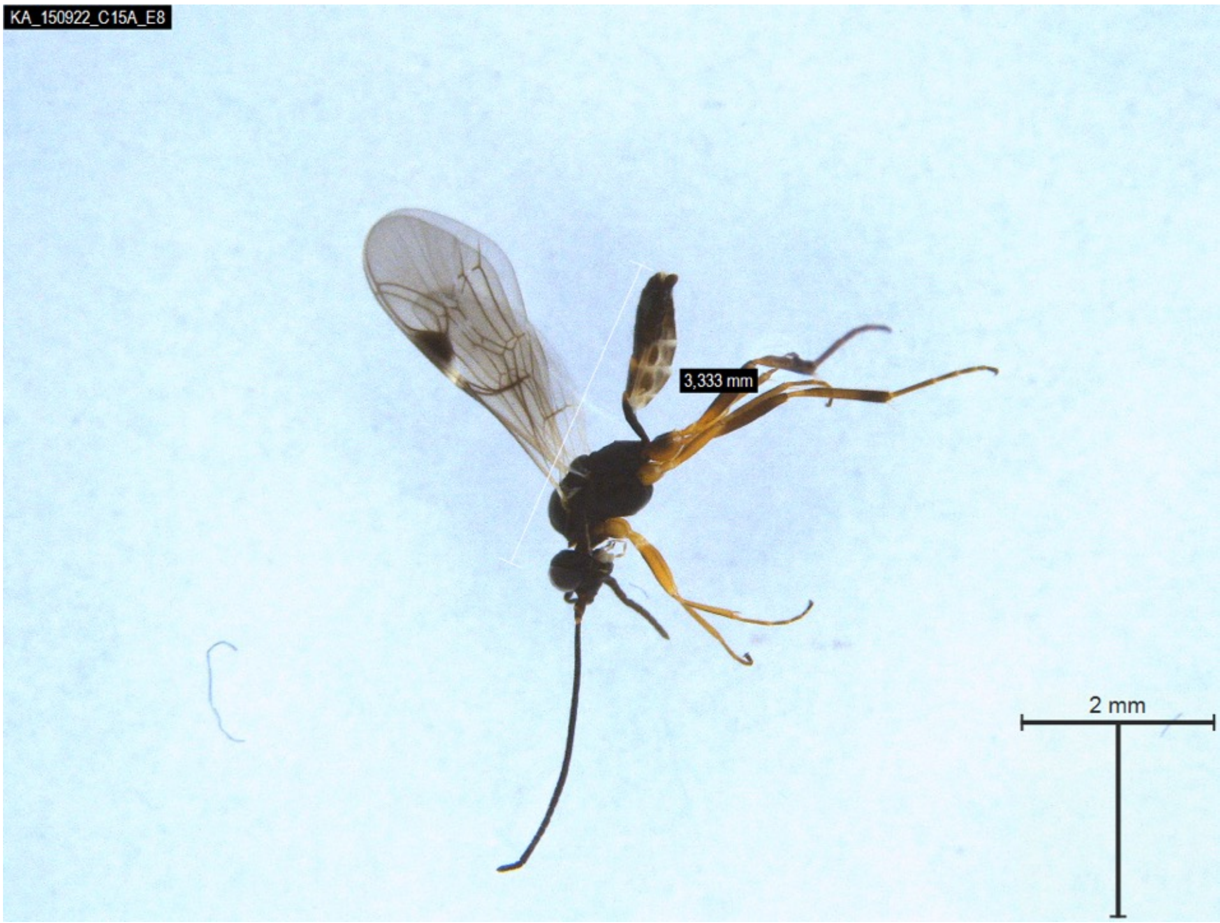

Figure S101: *Gelis sp.*

Sanger_ID: SQ_2022_057_059
Data_ID: BR_020621_C23A (conventional HPS 2000K)

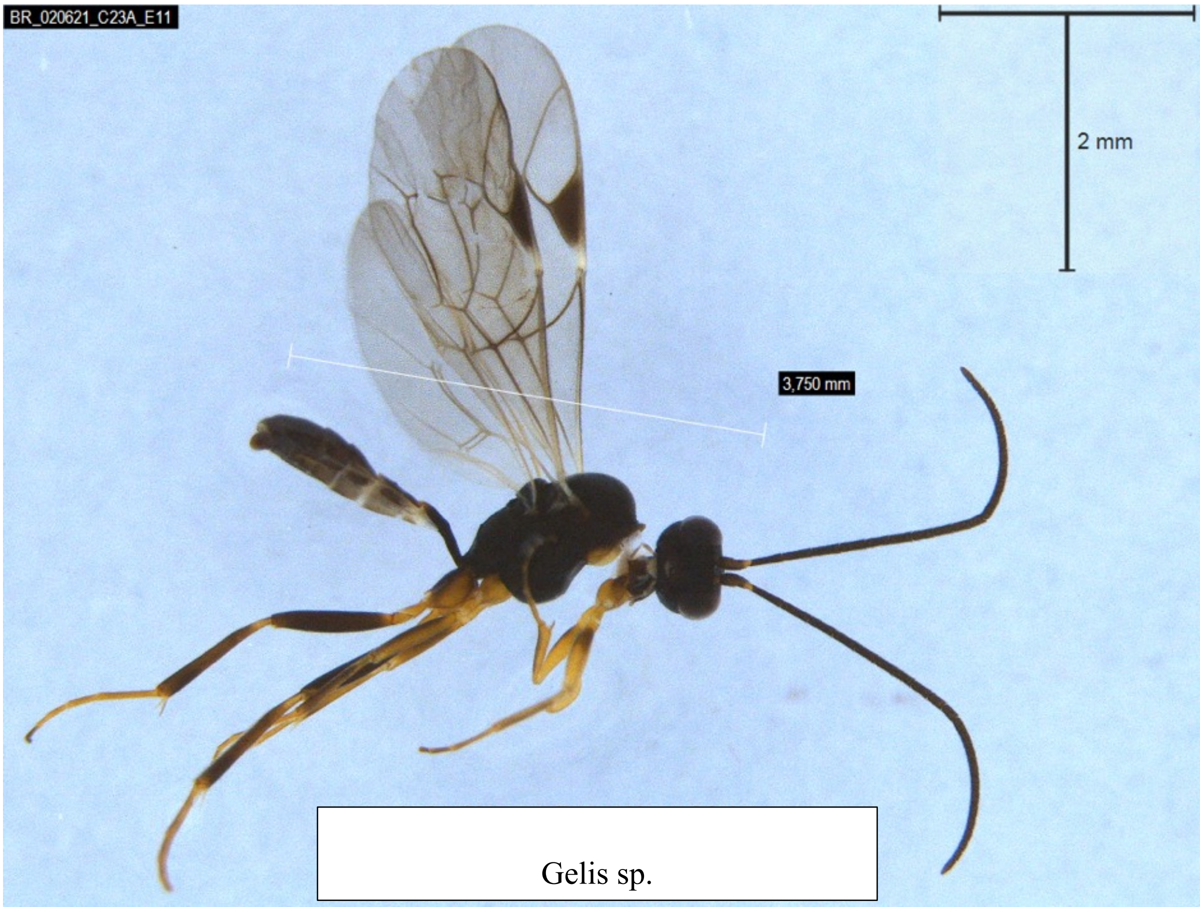

Figure S102: *Gelis sp.*
.

Inostemma sp. (Platygastridae)

GENUS | ACCEPTED

Inostemma Haliday, 1833

Published in: Haliday, A.H. (1833). Essay on the classification of the parasitic Hymenoptera of Britain, which correspond with the Ichneumones minuti of Linnaeus. *Entomological Magazine*. 1(3): 259-276.

In: GBIF Backbone Taxonomy

646 OCCURRENCES 101 SPECIES

OVERVIEW 1 TREATMENT METRICS

23 OCCURRENCES WITH IMAGES

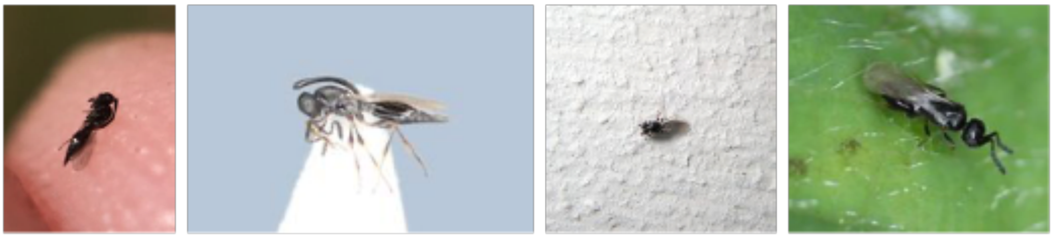

205 GEOREFERENCED RECORDS

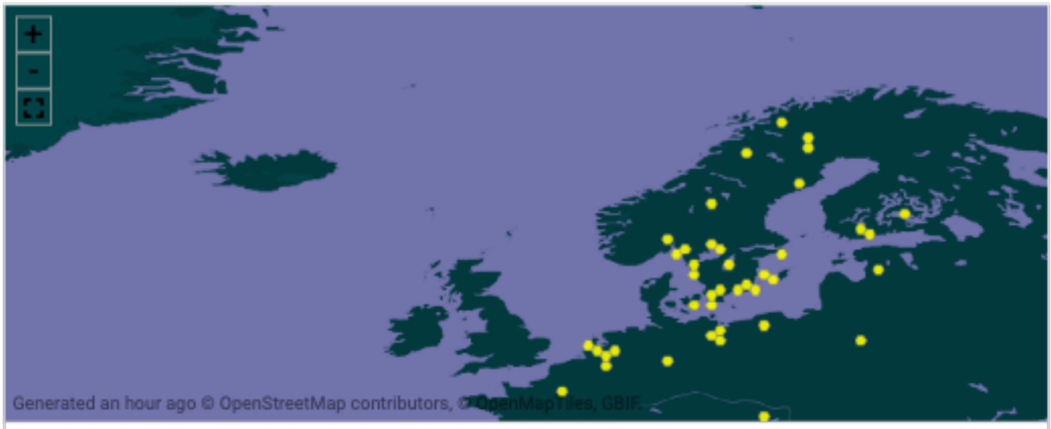

Any year 1868 - 2023 EXPLORE AREA

APPEARS IN 25 CHECKLIST DATASETS:

GBIF Backbone Taxonomy
As *Inostemma* Haliday, 1833

Catalogue of Life Checklist
As *Inostemma* Haliday, 1833

Figure S103: Global Biodiversity Information Facility (GBIF) Webpage *Inostemma*

specimen damaged, ovipositor housing lost
Sanger_ID: SQ_2022_057_052
Data_ID: HA_180822_C2A (conventional HPS 2000K)

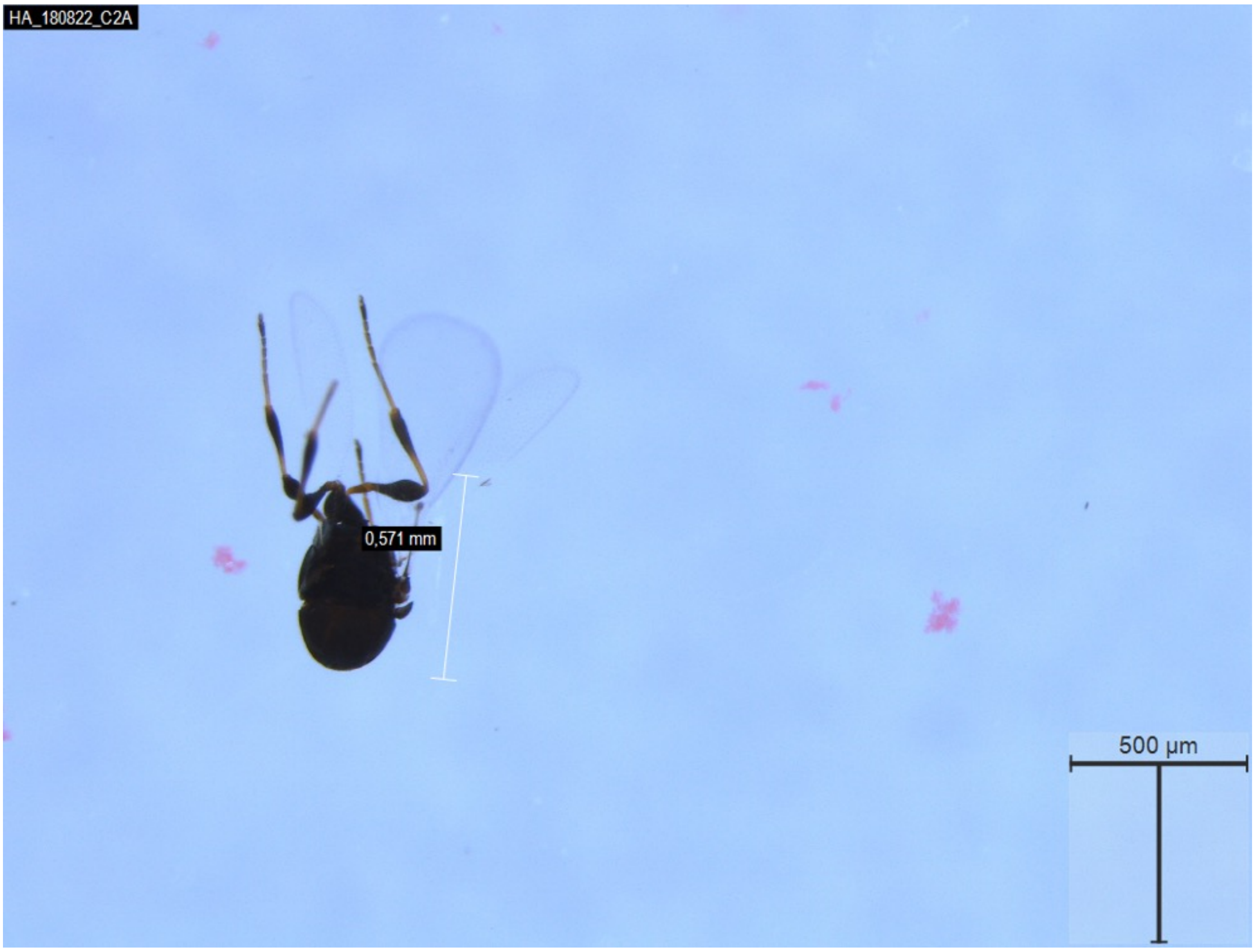

Figure S104: *Inostemma sp.*

Sanger_ID: SQ_2022_057_083
Data_ID: BR_020621_C24B (conventional HPS 2000K)

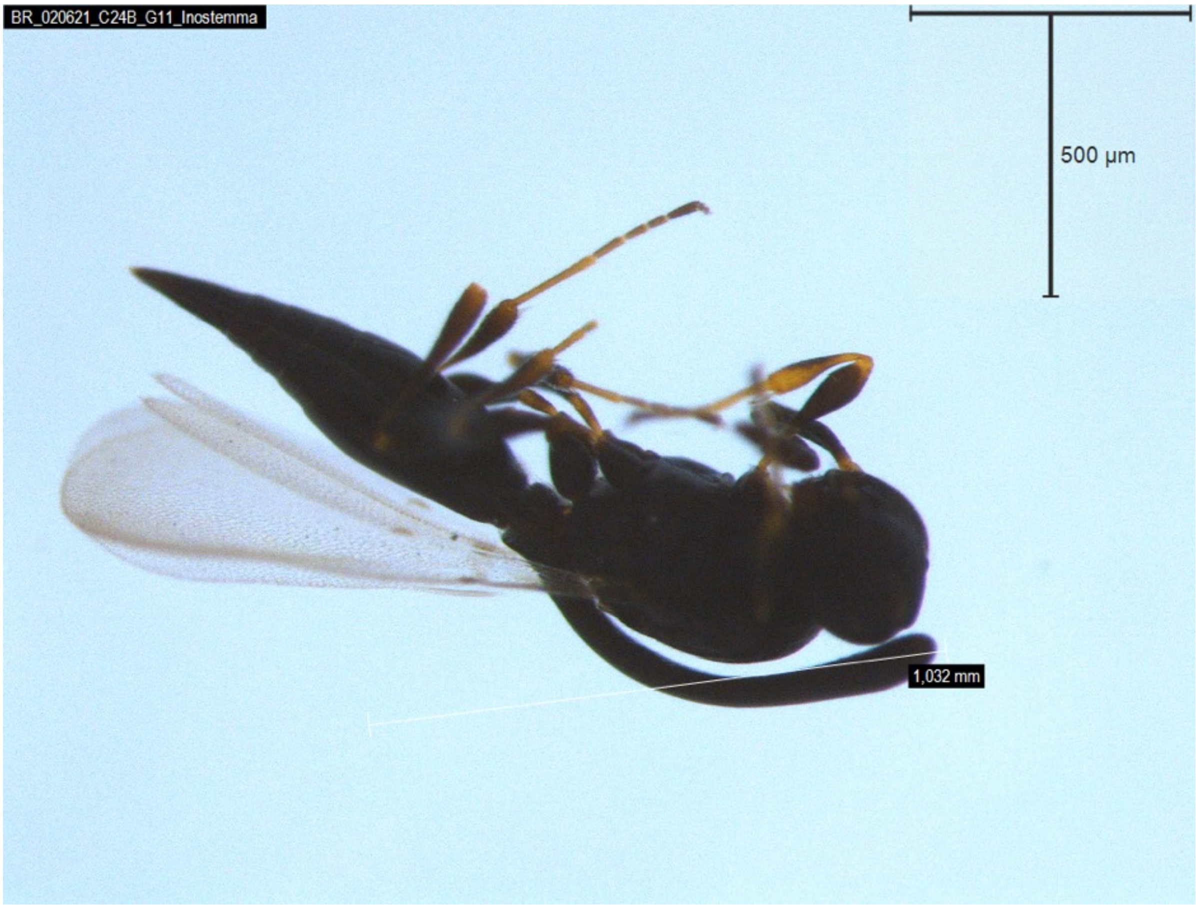

Figure S105: *Inostemma sp.*

Leptopilina japonica (Figitidae)

SPECIES | ACCEPTED

Leptopilina japonica

In: GBIF Backbone Taxonomy

26 OCCURRENCES

OVERVIEW METRICS

5 GEOREFERENCED RECORDS

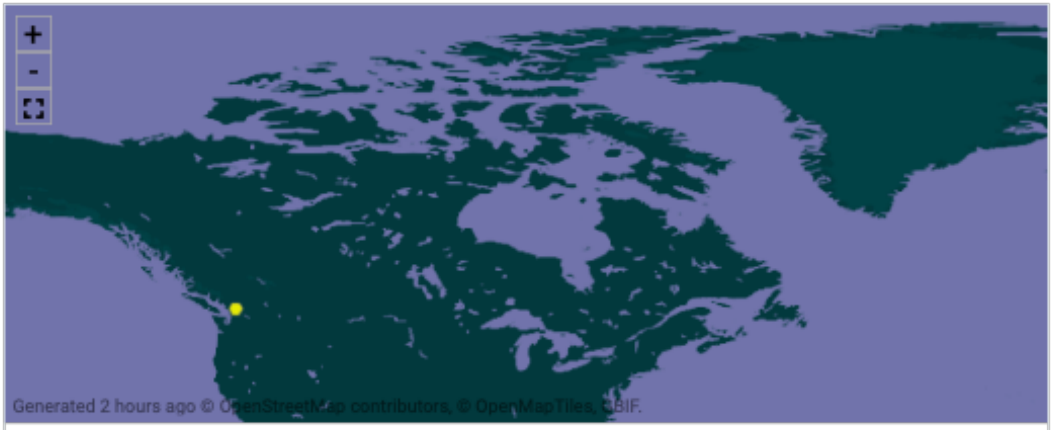

Any year 2019 - 2020 EXPLORE

APPEARS IN 5 CHECKLIST DATASETS:

GBIF Backbone Taxonomy
As *Leptopilina japonica*

NCBI Taxonomy
As *Leptopilina japonica*

The European Nucleotide Archive (ENA) taxonomy
As *Leptopilina japonica*

International Barcode of Life project (iBOL) Barcode Index Numbers (BINs)
As *Leptopilina japonica*

Cynipoidea (gall wasps) - xBio:D Cyberinfrastructure, The Ohio State University
As *Leptopilina japonica* Novković & Kimura

Figure S106: Global Biodiversity Information Facility (GBIF) Webpage *Leptopilina japonica*

Sanger_ID: SQ_2022_057_062
Data_ID: BR_160822_C24A (conventional HPS 2000K)

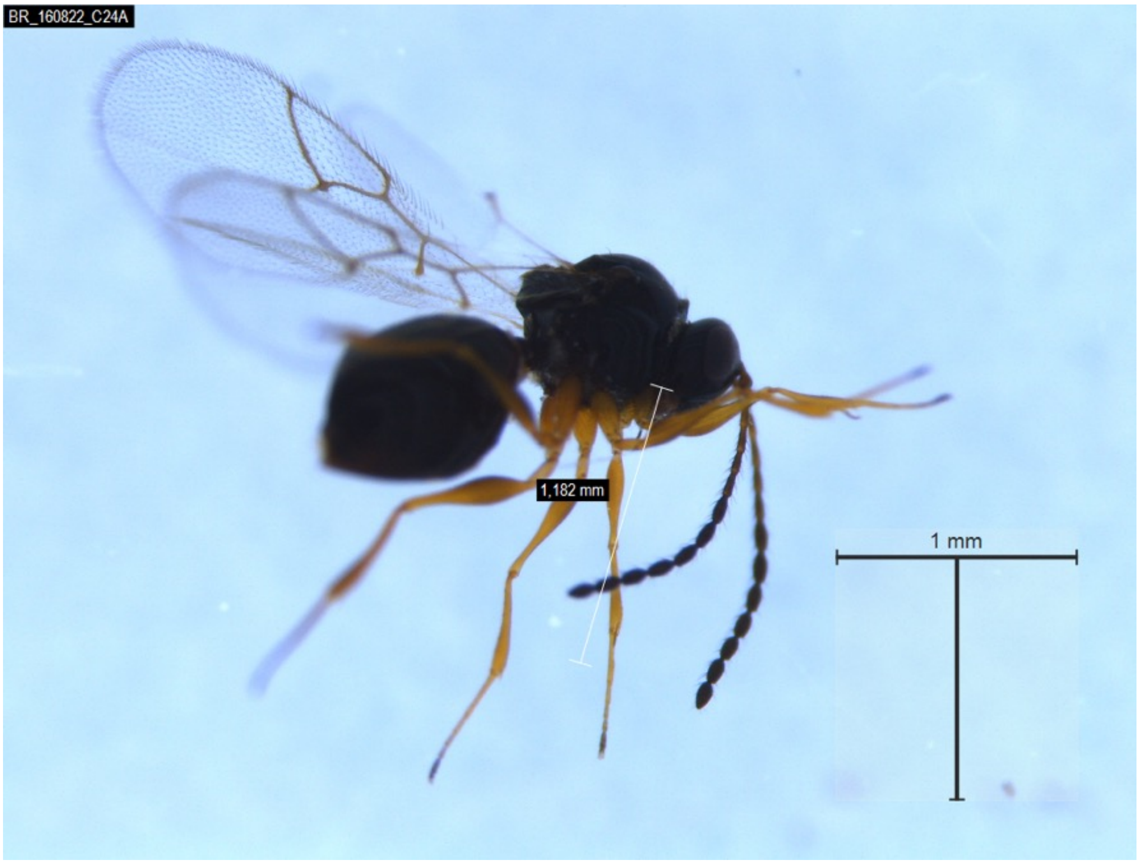

Figure S107: *Leptopilina japonica*

Lipolexis sp. (Braconidae)

17/11/2023, 13:10 Lipolexis Förster, 1862

GENUS | ACCEPTED

Lipolexis Förster, 1862

Published in: Foerster, Arnold. 1862. Synopsis der Familien und Gattungen der Braconen. Verhandlungen des naturhistorischen Vereines der preussischen Rheinlande und Westphalens 19: 225-288; pl.3.
source: Taxapad Ichneumonoidea

225 OCCURRENCES 10 SPECIES

OVERVIEW METRICS REFERENCE TAXON

35 GEOREFERENCED RECORDS

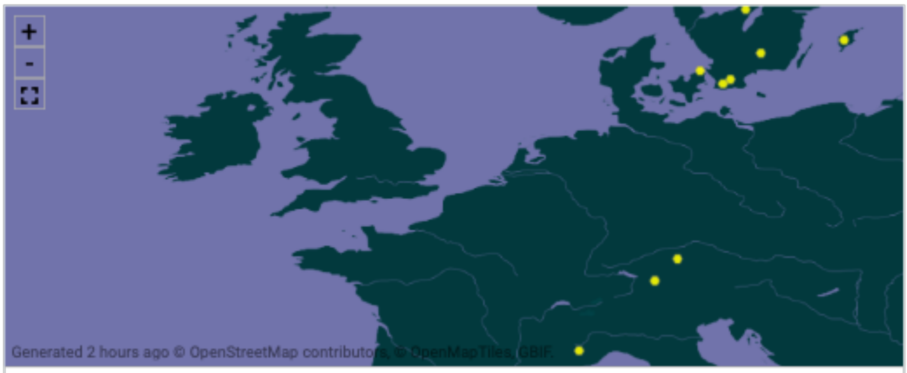

Any year 1980 - 2020 EXPLORE AREA

APPEARS IN 22 CHECKLIST DATASETS:

GBIF Backbone Taxonomy
As *Lipolexis* Förster, 1862

Catalogue of Life Checklist
As *Lipolexis*

NCBI Taxonomy
As *Lipolexis*

The European Nucleotide Archive (ENA) taxonomy
As *Lipolexis*

International Barcode of Life project (iBOL) Barcode Index Numbers (BINs)
As *Lipolexis*

https://www.gbif.org/species/1269578 1/3

Figure S108: Global Biodiversity Information Facility (GBIF) Webpage *Lipolexis*

Sanger_ID: SQ_2022_057_069
Data_ID: HA_120621_C5A (conventional HPS 2000K)

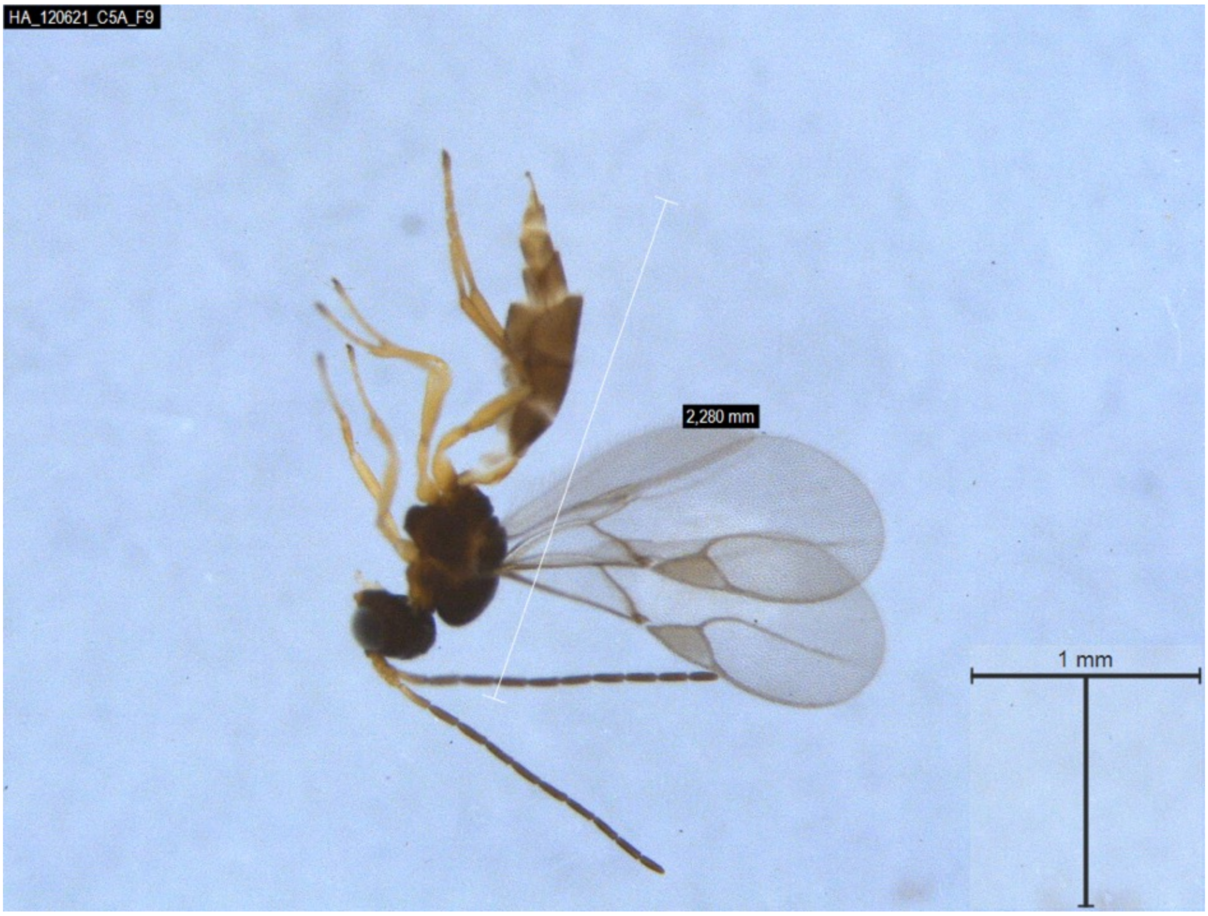

Figure S109: *Lipolexis sp. (cf gracilis)*

Ctenopelmatinae sp. (Ichneumonidae)

SPECIES | ACCEPTED

Mesoleius filicornis Holmgren, 1876

Published in: Holmgren, A.E. Dispositio Synoptica Mesoleiorum Scandinaviae. Kongliga Svenska Vetenskapsakademiens Handlingar. 13(12):1-51. (1876).
source: Taxapad Ichneumonoidea

23 OCCURRENCES

OVERVIEW METRICS REFERENCE TAXON

15 GEOREFERENCED RECORDS

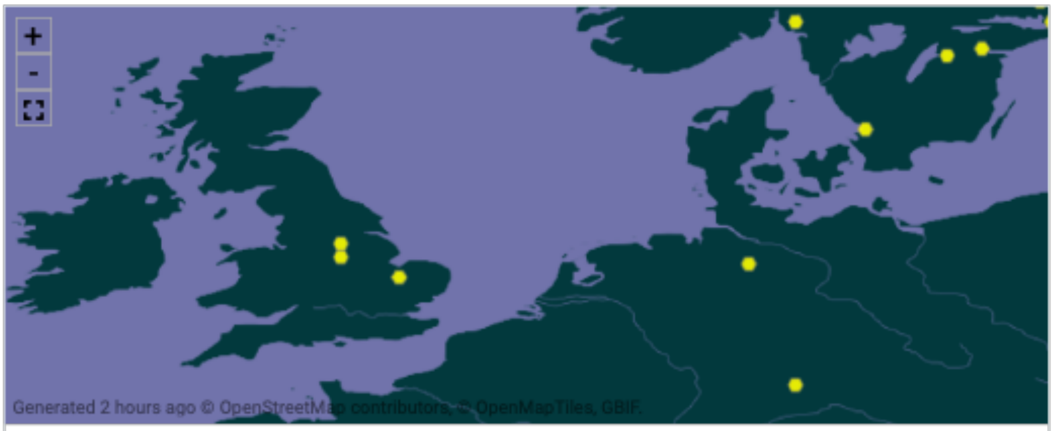

Any year 1902 - 2005 EXPLORE AREA

TYPE SPECIMENS

TYPE of Mesoleius filicornis Holmgren, 1876 Naturalis Hymenoptera BE.71342
Source: Naturalis Biodiversity Center (NL) - Museum collection digitized at storage unit level

TYPE of Mesoleius filicornis Holmgren, 1876 Naturalis Hymenoptera BE.77365
Source: Naturalis Biodiversity Center (NL) - Museum collection digitized at storage unit level

APPEARS IN 13 CHECKLIST DATASETS:

GBIF Backbone Taxonomy
As *Mesoleius filicornis* Holmgren, 1876

Catalogue of Life Checklist
As *Mesoleius filicornis* Holmgren, 1876

Figure S110: Global Biodiversity Information Facility (GBIF) Webpage *Mesoleius filicornis*

Sanger_ID: SQ_2022_057_097
Data_ID: KA_270921_C16A (conventional LED 4000K)

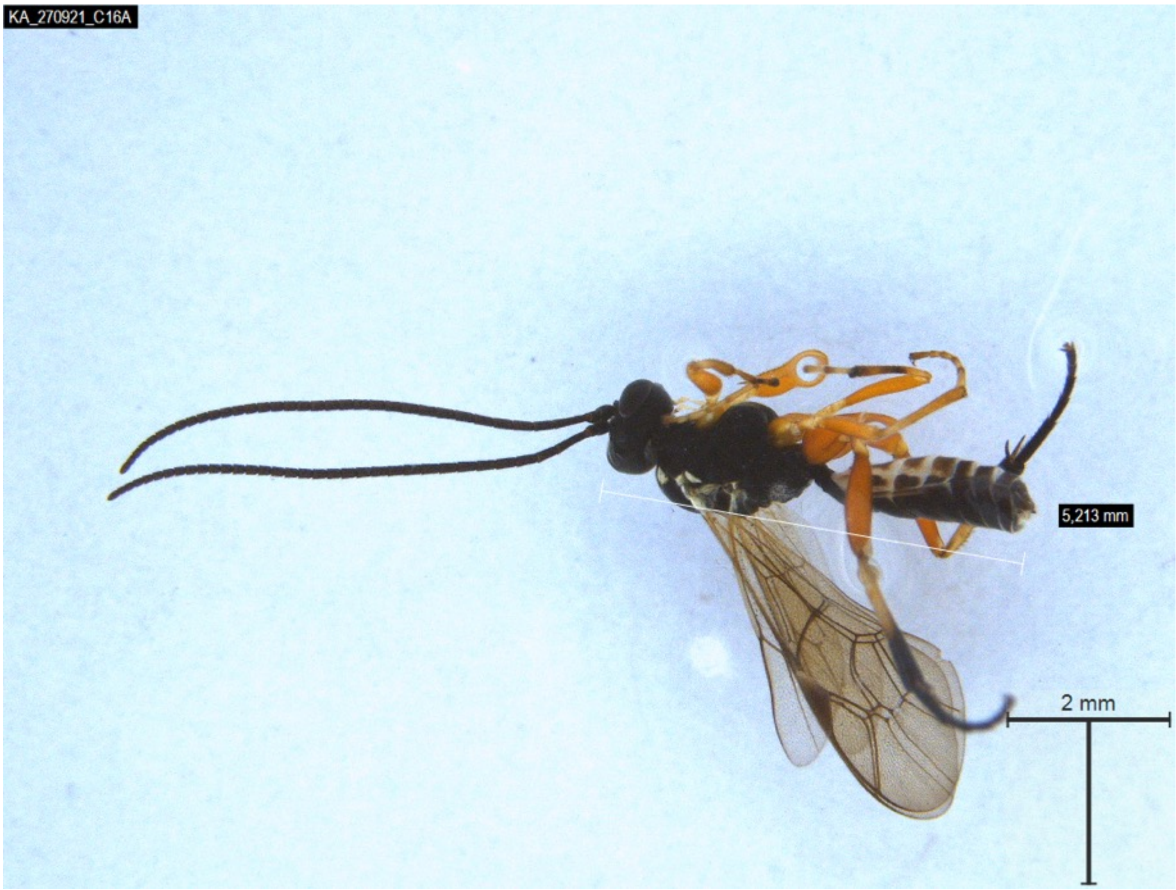

Figure S111: *Ctenopelmatinae sp.*

Metaphycus sp. (Encyrtidae)

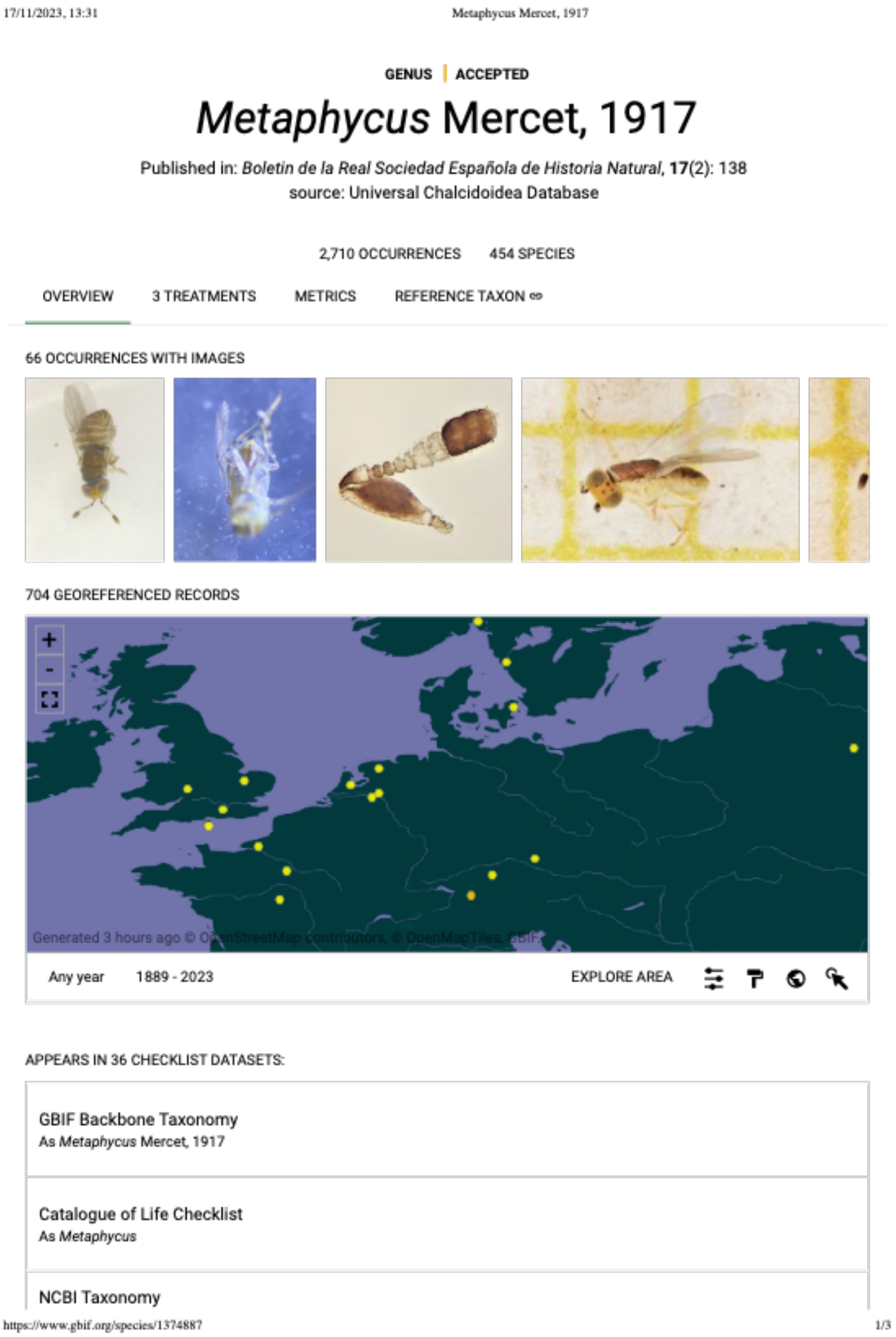

17/11/2023, 13:31 Metaphycus Mercet, 1917

GENUS | ACCEPTED

Metaphycus Mercet, 1917

Published in: *Boletin de la Real Sociedad Española de Historia Natural*, **17**(2): 138
source: Universal Chalcidoidea Database

2,710 OCCURRENCES 454 SPECIES

OVERVIEW 3 TREATMENTS METRICS REFERENCE TAXON

66 OCCURRENCES WITH IMAGES

704 GEOREFERENCED RECORDS

Generated 3 hours ago © OpenStreetMap contributors, © OpenMapTiles, GBIF.

Any year 1889 - 2023 EXPLORE AREA

APPEARS IN 36 CHECKLIST DATASETS:

GBIF Backbone Taxonomy
As *Metaphycus* Mercet, 1917

Catalogue of Life Checklist
As *Metaphycus*

NCBI Taxonomy

https://www.gbif.org/species/1374887 1/3

Figure S112: Global Biodiversity Information Facility (GBIF) Webpage *Metaphycus*

Sanger_ID: SQ_2022_057_028
Data_ID: KA_190722_C13A (tailored LED 4000K)

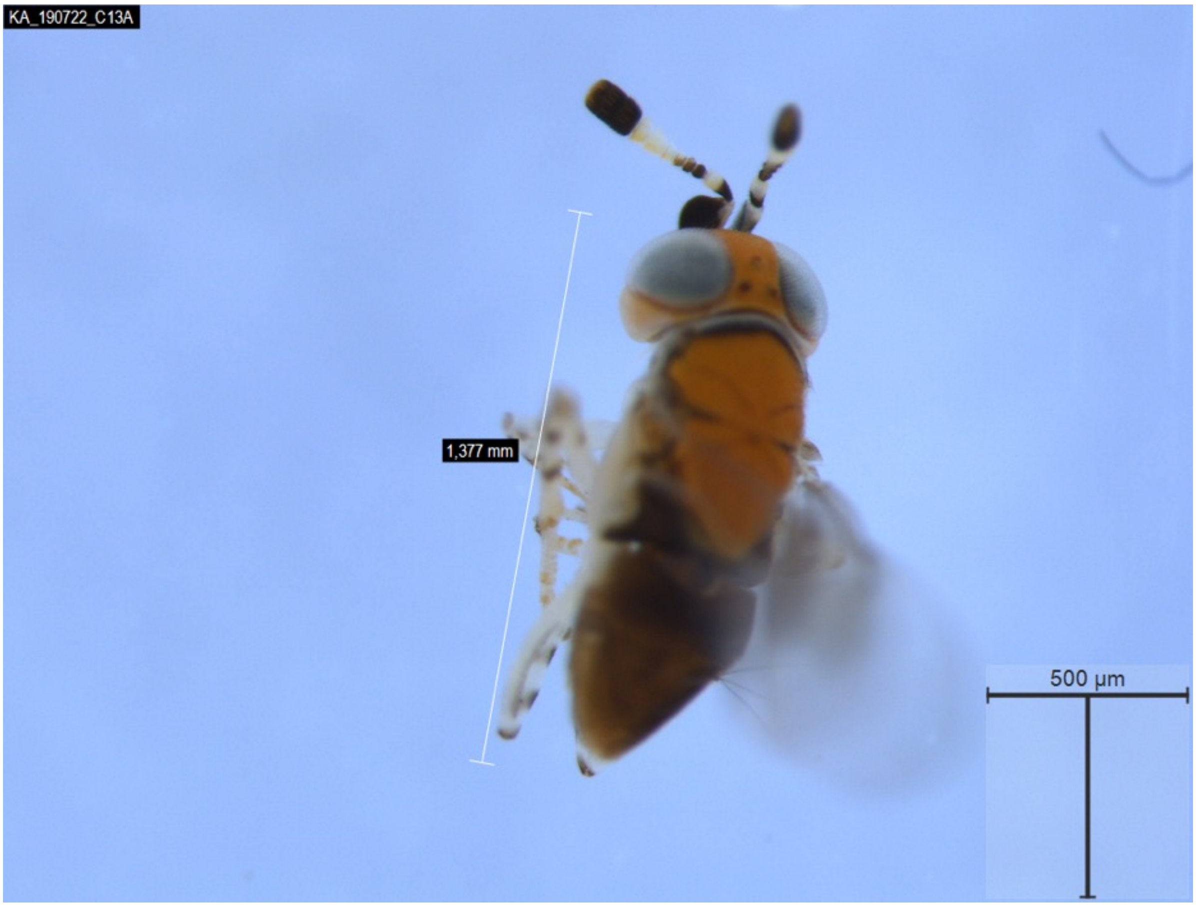

Figure S113: *Metaphycus sp. (cf flavus)*

Meteorus affinis (Braconidae)

SPECIES | **ACCEPTED**

Meteorus affinis (Wesmael, 1835)

Published in: Wesmael, C. Monographie des Braconides de Belgique. Nouveaux Memoires de l'Academie Royale des Sciences et Belles-lettres Bruxelles. 9:1-252. (1835).
source: Taxapad Ichneumonoidea

Basionym: *Perilitus affinis* Wesmael, 1835

261 OCCURRENCES 7 INFRASPECIES

OVERVIEW 1 TREATMENT METRICS REFERENCE TAXON

2 OCCURRENCES WITH IMAGES

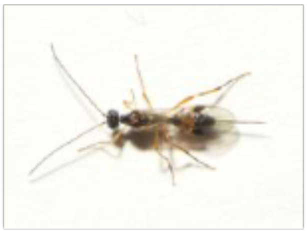
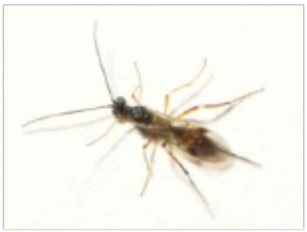

117 GEOREFERENCED RECORDS

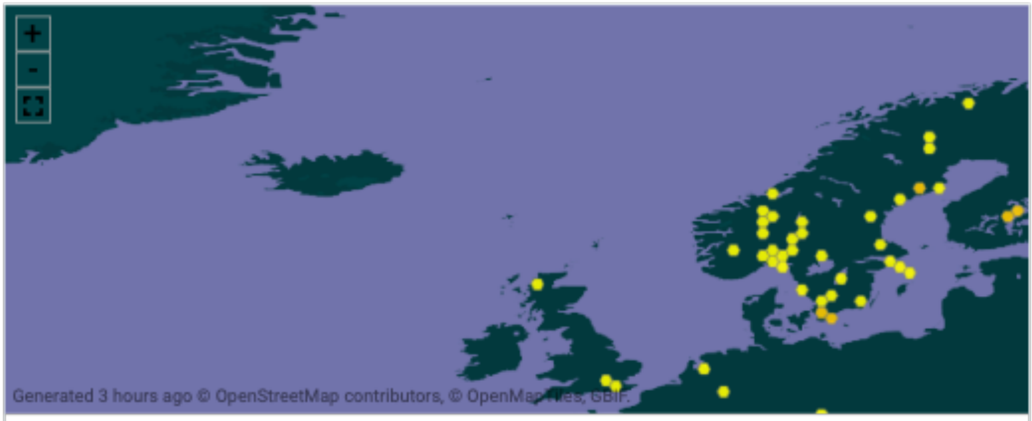

Any year 1940 - 2023 EXPLORE AREA

TYPE SPECIMENS

LECTOTYPE of Perilitus affinis Wesmael, 1835 RBINS-Scientific Heritage urn:catalog:RBINS:TVC EVCT.685
Source: RBINS DaRWIN

NAME USAGES APPLIED TO OCCURRENCES IN GBIF

Figure S114: Global Biodiversity Information Facility (GBIF) Webpage *Meteorus affinis*

Sanger_ID: SQ_2022_057_084
Data_ID: HA_260722_C5B (conventional HPS 2000K)

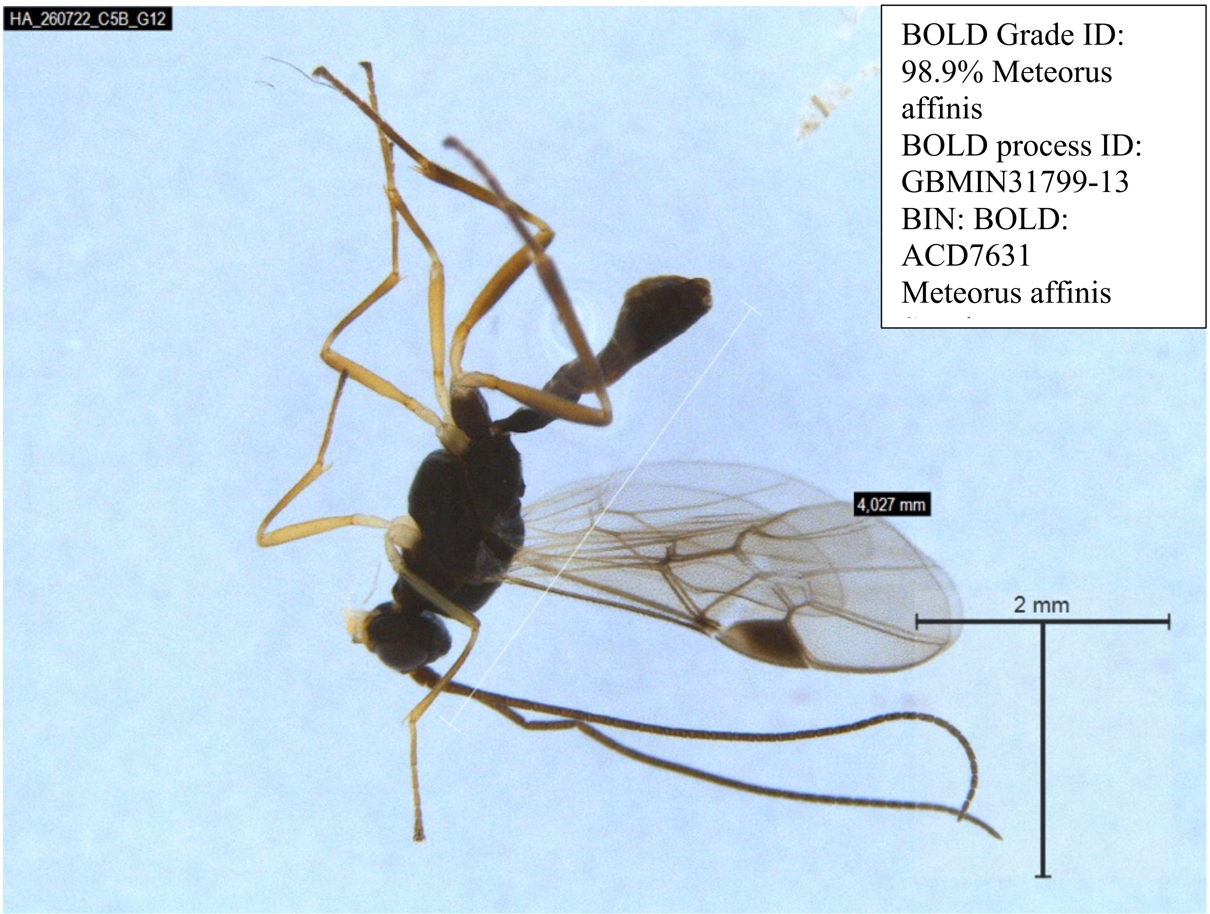

BOLD Grade ID:
98.9% Meteorus affinis
BOLD process ID:
GBMIN31799-13
BIN: BOLD:
ACD7631
Meteorus affinis

Figure S115: *Meteorus affinis*

Metopius fuscipennis (Ichneumonidae)

SPECIES | ACCEPTED

Metopius fuscipennis Wesmael, 1849

Published in: Wesmael, C. Notice sur les Ichneumonides de Belgique appartenant aux genres Metopius, Banchus et Coleocentrus. Bulletin de l'Academie Royale des Sciences, des Lettres et des Beaux-Arts de Belgique. 16(1):620-634. (1849).
source: Taxapad Ichneumonoidea

134 OCCURRENCES 1 INFRASPECIES

OVERVIEW METRICS REFERENCE TAXON

49 OCCURRENCES WITH IMAGES

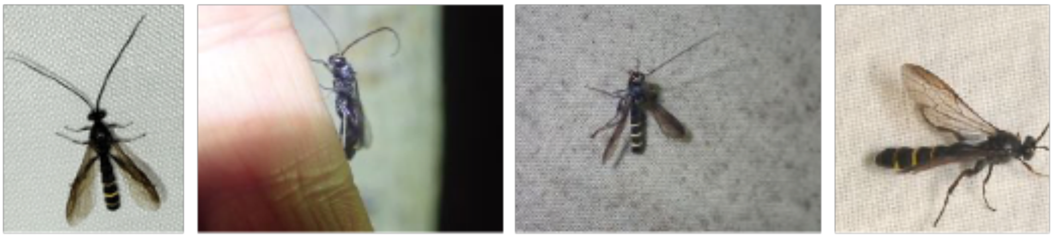

105 GEOREFERENCED RECORDS

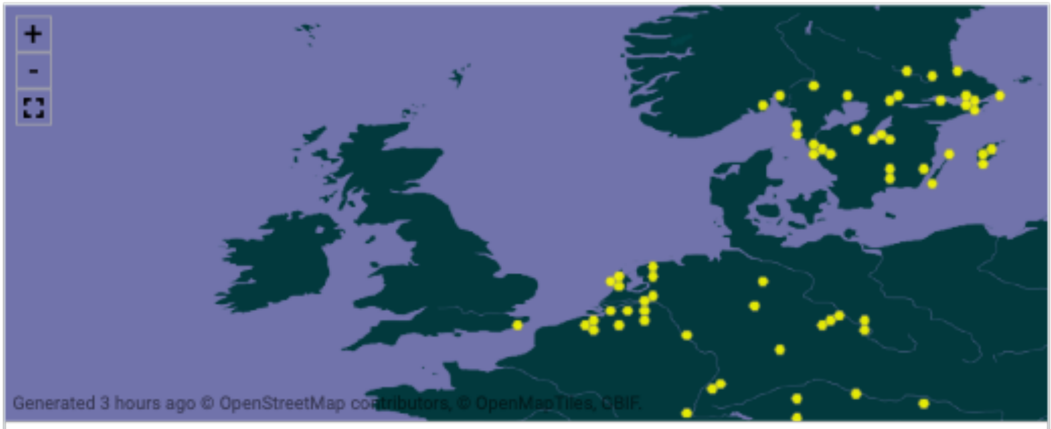

Any year 1986 - 2023 EXPLORE AREA

NAME USAGES APPLIED TO OCCURRENCES IN GBIF

Figure S116: Global Biodiversity Information Facility (GBIF) Webpage *Metopius fiscipennis*

Sanger_ID: SQ_2022_057_103
Data_ID: BR_300822_C26A (conventional HPS 2000K)

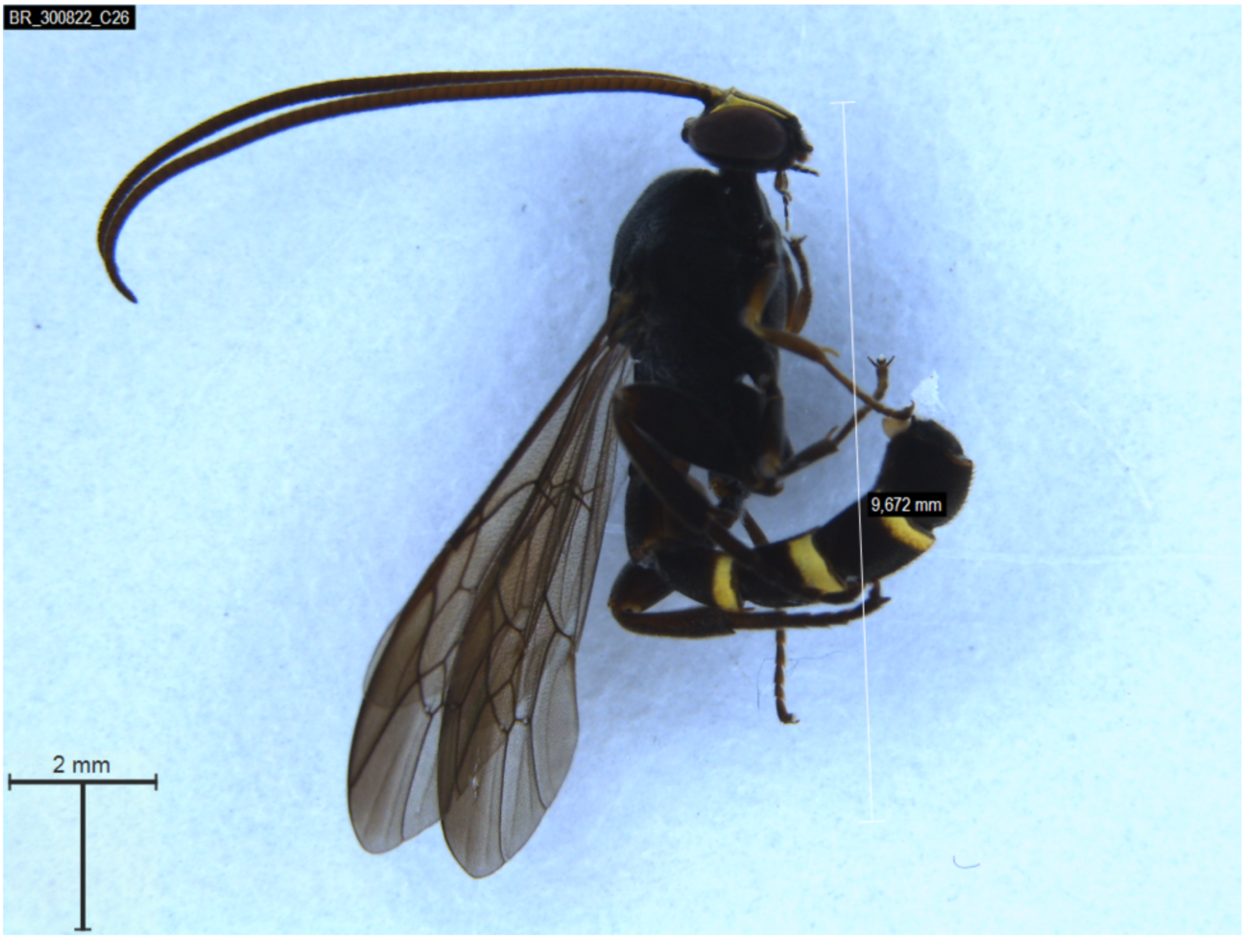

Figure S117: *Metopius fuscipennis*

Mymaridae sp.1 (Mymaridae)

FAMILY | ACCEPTED

Mymaridae

Published in: *in* Westwood, *Introd. mod. Classif. Brit. Ins.*, **2**: 173
source: Universal Chalcidoidea Database

Fairyflies In English

83,401 OCCURRENCES 1,603 SPECIES

OVERVIEW METRICS REFERENCE TAXON

4,906 OCCURRENCES WITH IMAGES

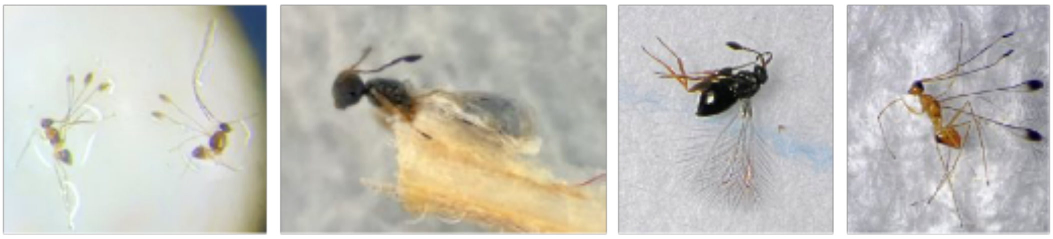

4,296 GEOREFERENCED RECORDS

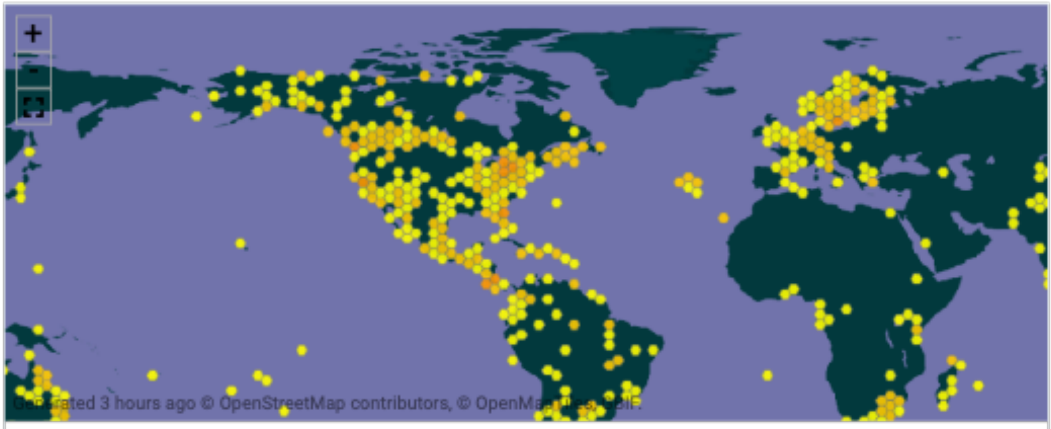

Any year 1800 - 2023 EXPLORE

VERNACULAR NAMES

Fairyflies In English
sources: Integrated Taxonomic Information System (ITIS) + 1 more dataset

Fairyfly In English
source: The Paleobiology Database

Dvärgsteklar In Swedish

Figure S118: Global Biodiversity Information Facility (GBIF) Webpage *Mymaridae*

Sanger_ID: SQ_2022_057_025
Data_ID: HA_240822_C5A (conventional HPS 2000K)

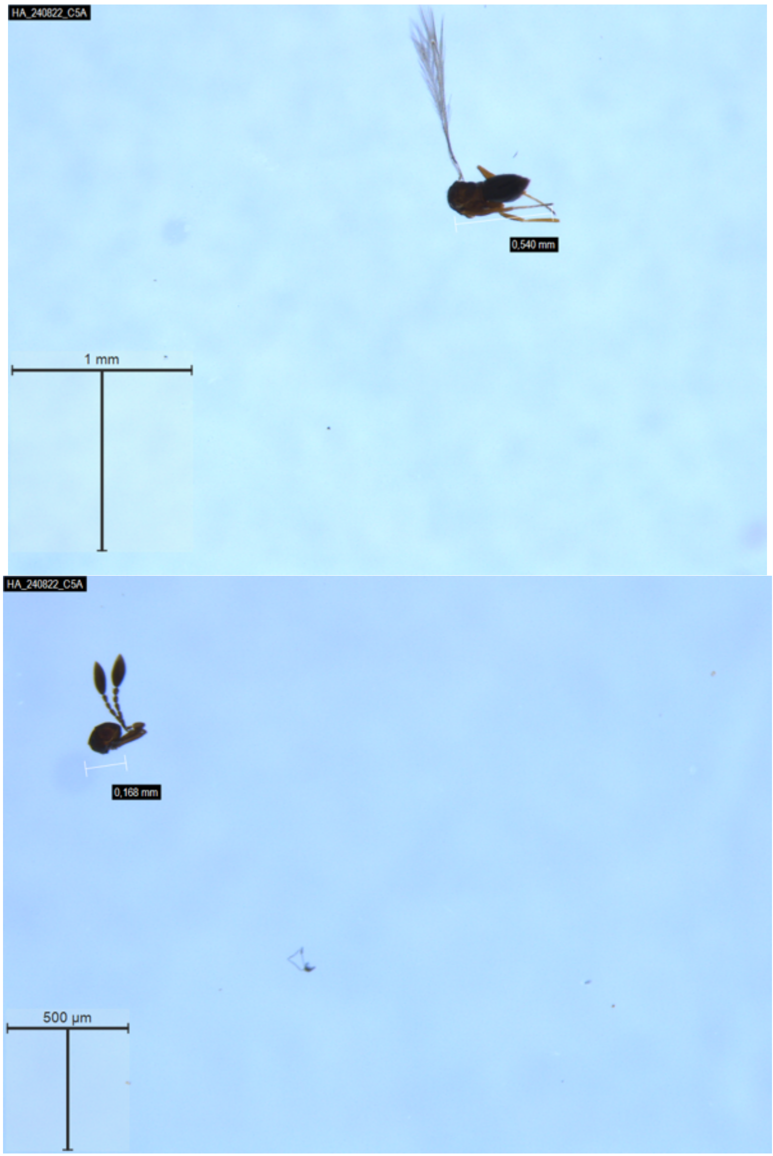

Figure S119: *Mymaridae sp1.*

Mymaridae sp.1(Mymaridae)

Sanger_ID: SQ_2022_057_086
Data_ID: HA_240822_C7A (conventional HPS 2000K)

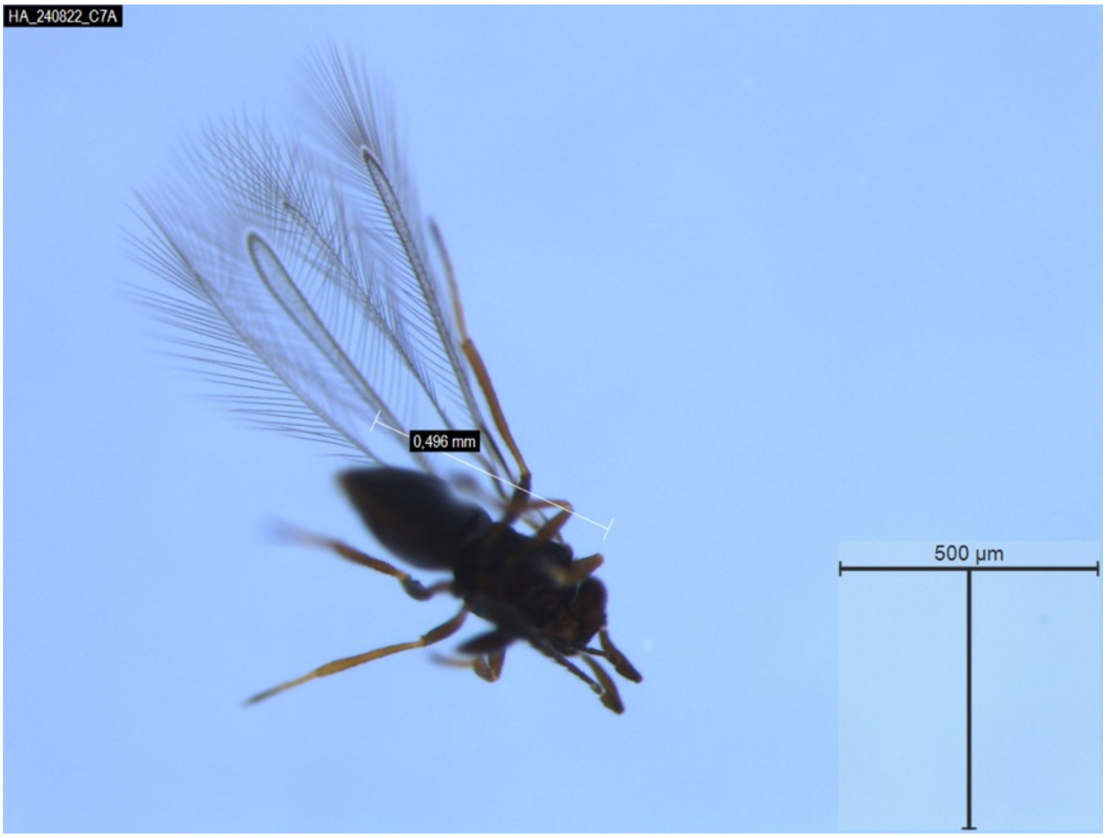

Figure S120: *Mymaridae sp1.*

Mymaridae sp.2 (Mymaridae)

Sanger_ID: SQ_2022_057_027
Data_ID: KA_160822_C22A (tailored LED 4000K)

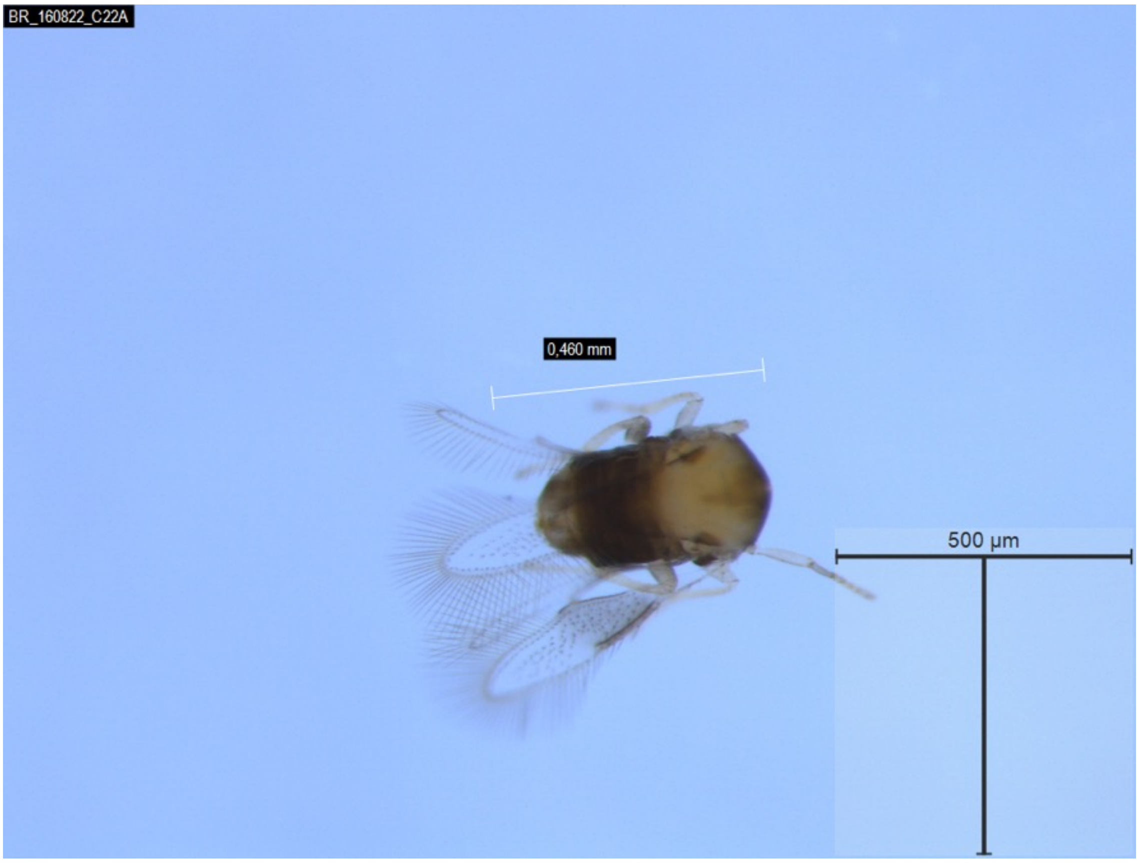

Figure S121: Mymaridae sp2.

Mymaridae sp.3 (Mymaridae)

Sanger_ID: SQ_2022_057_074
Data_ID: HA_180822_C7A (conventional HPS 2000K)

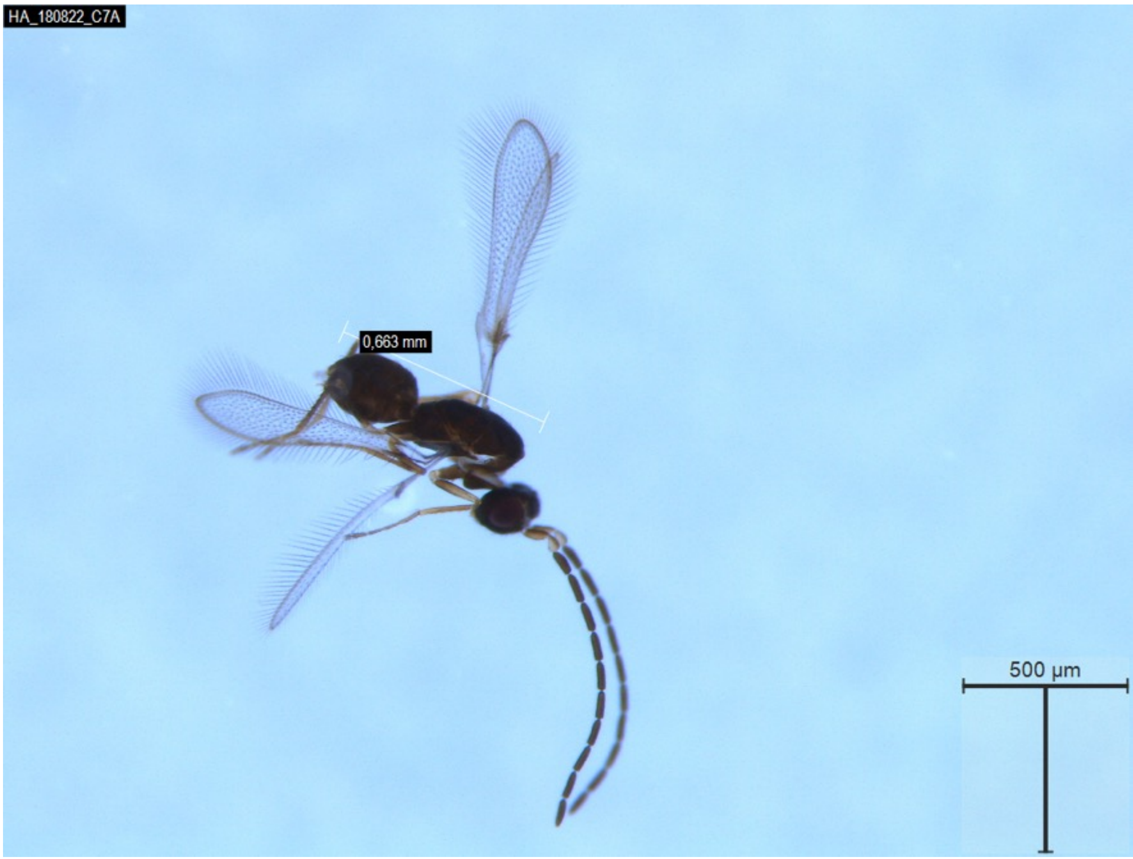

Figure S122: *Mymaridae sp3.*

Netelia sp. (Ichneumonidae)

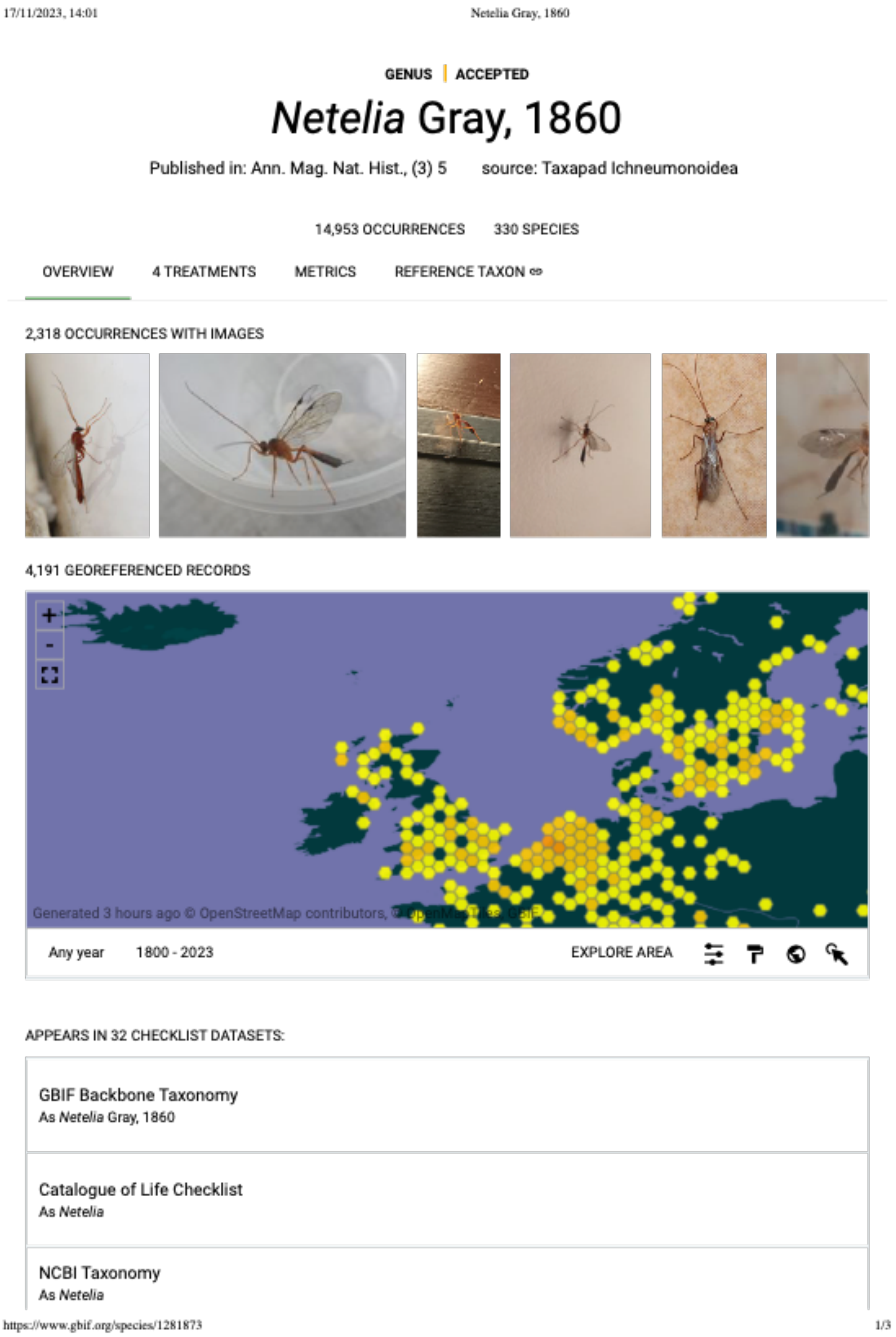

17/11/2023, 14:01 Netelia Gray, 1860

GENUS | ACCEPTED

Netelia Gray, 1860

Published in: Ann. Mag. Nat. Hist., (3) 5 source: Taxapad Ichneumonoidea

14,953 OCCURRENCES 330 SPECIES

OVERVIEW 4 TREATMENTS METRICS REFERENCE TAXON

2,318 OCCURRENCES WITH IMAGES

4,191 GEOREFERENCED RECORDS

Generated 3 hours ago © OpenStreetMap contributors, © OpenMapTiles, GBIF.

Any year 1800 - 2023 EXPLORE AREA

APPEARS IN 32 CHECKLIST DATASETS:

GBIF Backbone Taxonomy
As *Netelia* Gray, 1860

Catalogue of Life Checklist
As *Netelia*

NCBI Taxonomy
As *Netelia*

https://www.gbif.org/species/1281873 1/3

Figure S123: Global Biodiversity Information Facility (GBIF) Webpage *Netelia*

Sanger_ID: SQ_2022_057_102
Data_ID: HA_260722_C1A (tailored LED 2700K)

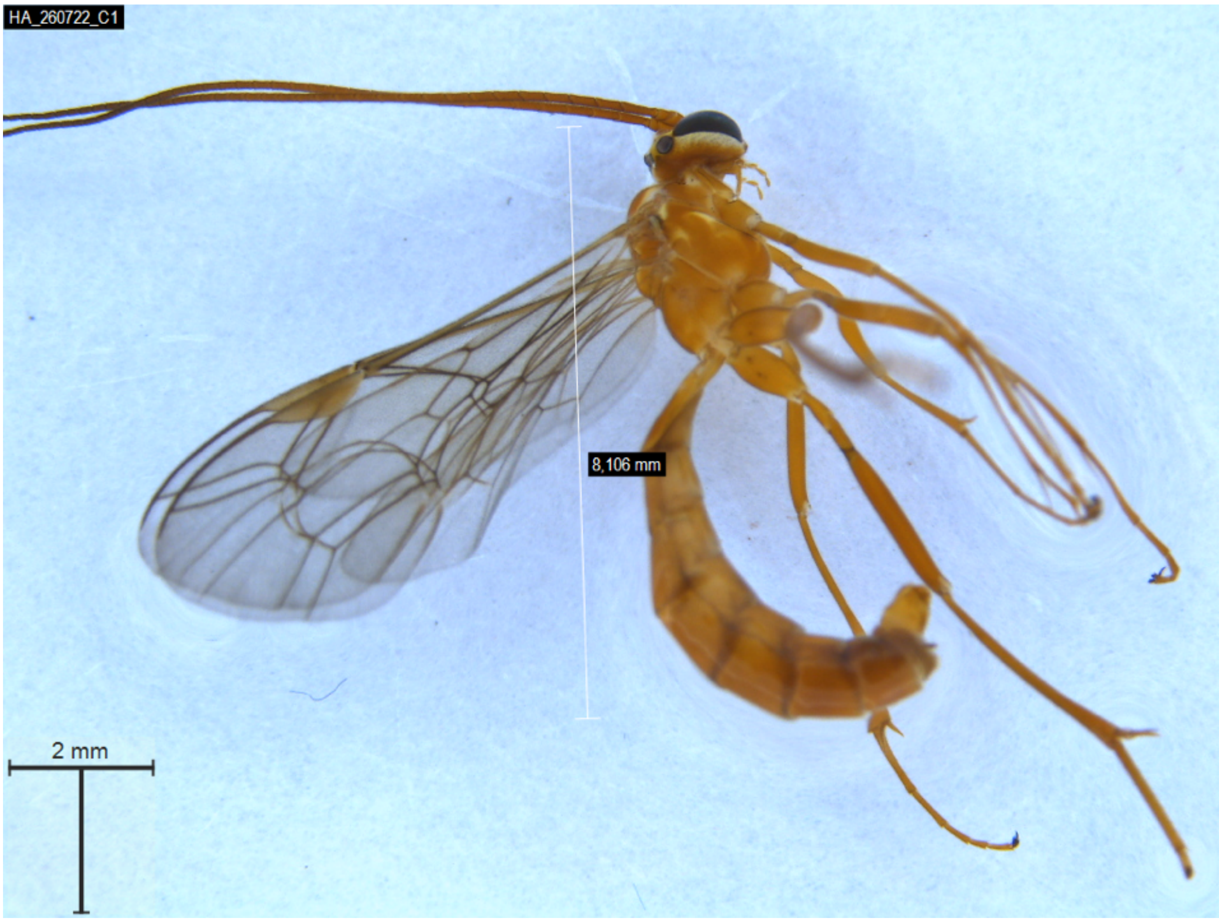

Figure S124: *Netelia sp.*

Omphale lugens (Eulophidae)

17/11/2023, 14:10 Omphale lugens (Nees, 1834)

SPECIES | ACCEPTED

Omphale lugens (Nees, 1834)

Published in: Efremova, Z.A.; Kriskovich, M.V. A new species of the genus |Omphale| (Hymenoptera, Eulophidae) from the Far East. JOURBOOK: Zoologicheskiy Zhurnal VOLUME: 73(7,8) PAGES: 245-248. (1994).
source: Universal Chalcidoidea Database

Basionym: *Eulophus lugens* Nees, 1834

38 OCCURRENCES 1 INFRASPECIES

OVERVIEW 1 TREATMENT METRICS REFERENCE TAXON

23 GEOREFERENCED RECORDS

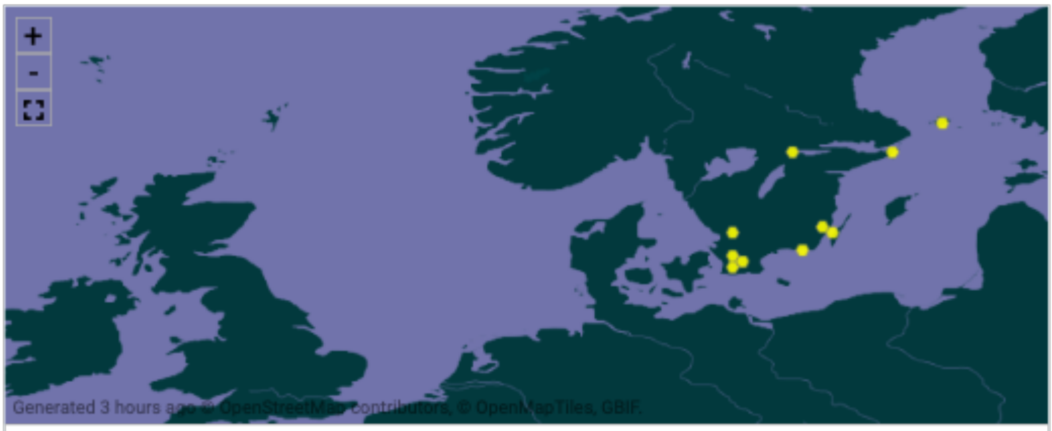

Any year 1878 - 2014 EXPLORE AREA

TYPE SPECIMENS

LECTOTYPE of Entedon navius Walker, 1839 NHMUK BMNH(E) NHMUK010838574
Source: Natural History Museum (London) Collection Specimens

NEOTYPE of Eulophus lugens Nees, 1834 Naturalis Hymenoptera BE.2270108
Source: Naturalis Biodiversity Center (NL) - Museum collection digitized at storage unit level

NAME USAGES APPLIED TO OCCURRENCES IN GBIF

https://www.gbif.org/species/1382655 1/5

Figure S125: Global Biodiversity Information Facility (GBIF) Webpage *Omphale lugens*

Sanger_ID: SQ_2022_057_045
Data_ID: HA_120621_C1B (conventional HPS 2000K)

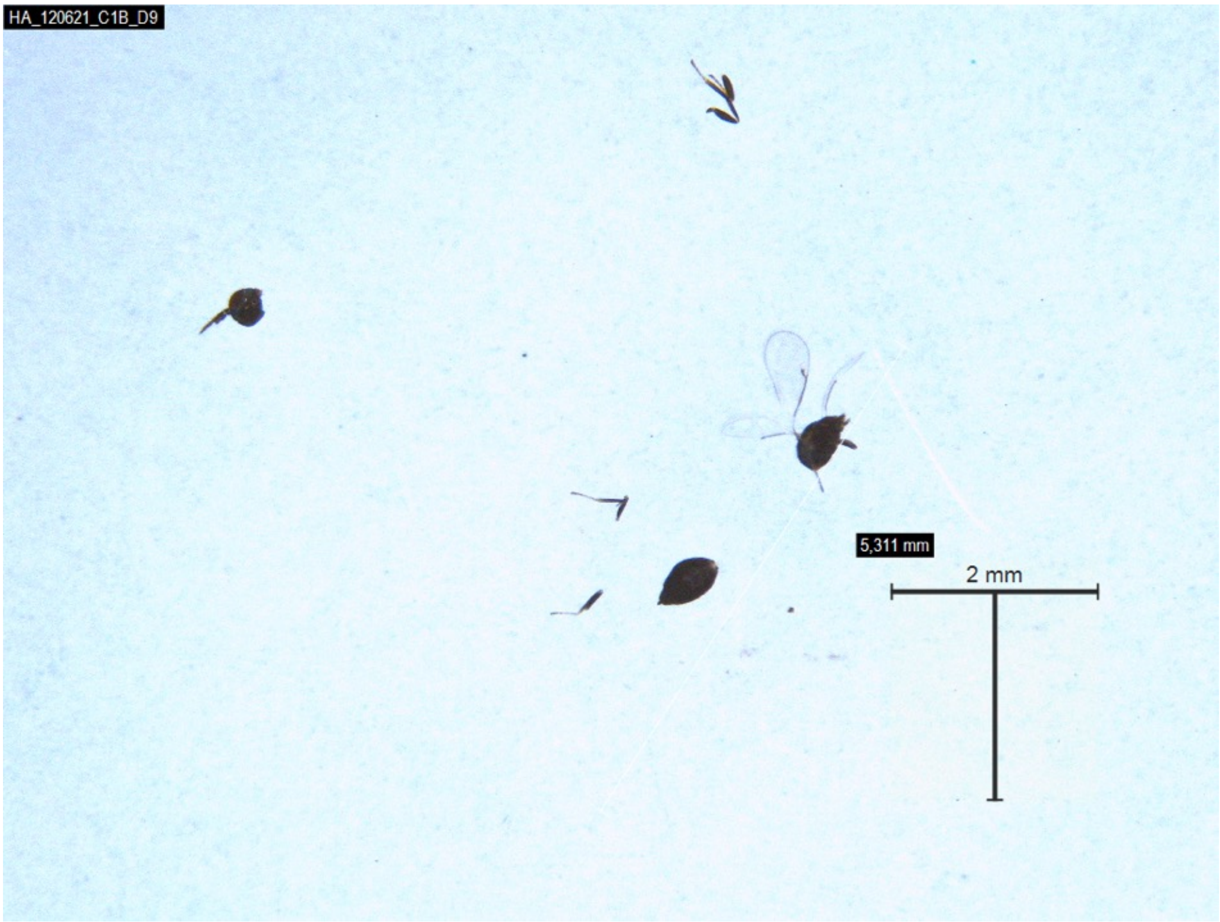

Figure S126: *Omphale lugens*

Omphale radialis (Eulophidae)

SPECIES | ACCEPTED

Omphale radialis (Thomson, 1878)

Published in: Graham, M.W.R. de V. Keys to the British genera and species of Elachertinae, Eulophinae, Entedontinae and Euderinae (Hym., Chalcidoidea). JOURBOOK: Transactions of the Society for British Entomology VOLUME: 13(10) PAGES: 169-204. (1959).
source: Universal Chalcidoidea Database

Basionym: *Derostenus radialis* Thomson, 1878

16 OCCURRENCES 1 INFRASPECIES

OVERVIEW METRICS REFERENCE TAXON

6 GEOREFERENCED RECORDS

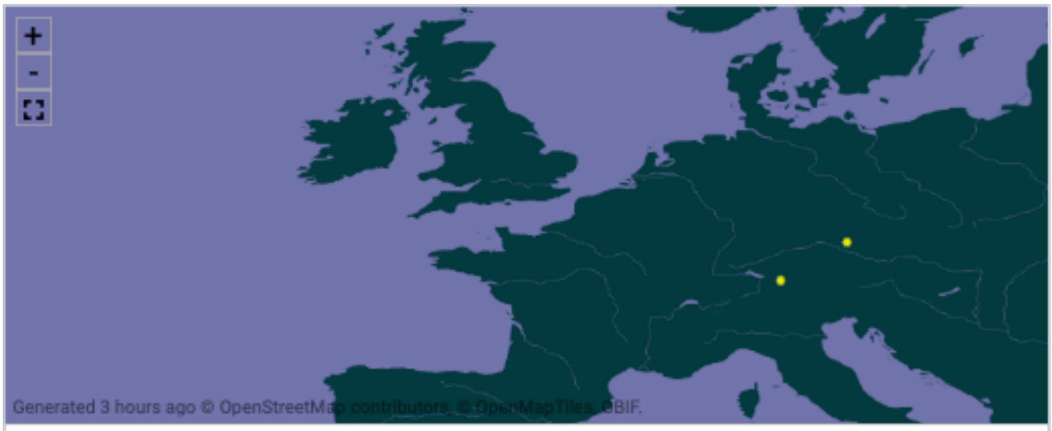

Any year 2012 - 2016 EXPLORE AREA

TYPE SPECIMENS

TYPE of Achrysocharella americana Girault, 1916 Girault. USNM ENT USNMENT01545217
Source: NMNH Extant Specimen Records (USNM, US)

NAME USAGES APPLIED TO OCCURRENCES IN GBIF

Figure S127: Global Biodiversity Information Facility (GBIF) Webpage *Omphale radialis*

Sanger_ID: SQ_2022_057_039
Data_ID: HA_240822_C8A (conventional HPS 2000K)

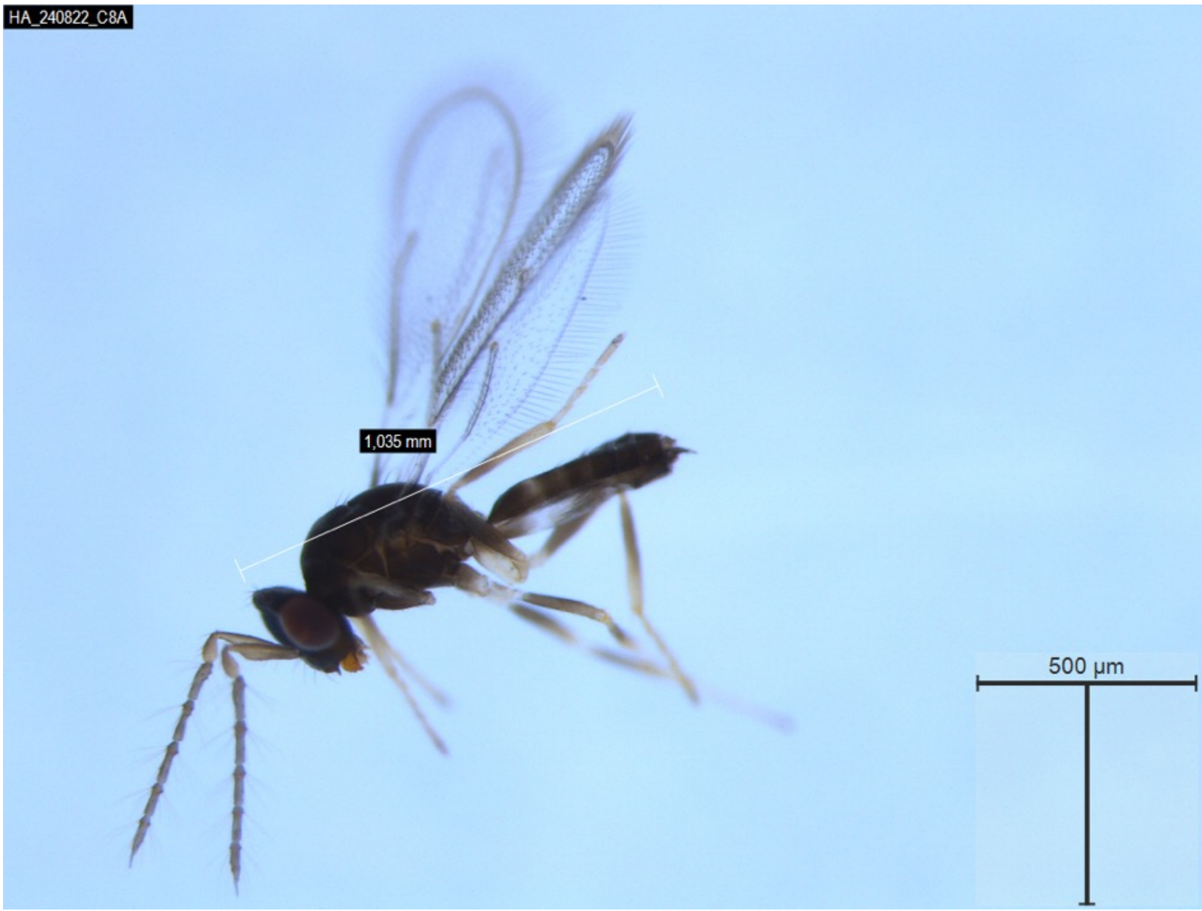

Figure S128: *Omphale radialis*

Omphale sp. (Eulophidae)

GENUS | ACCEPTED

Omphale Haliday, 1833

Published in: Entom. Mag., 1 (4) source: Universal Chalcidoidea Database

3,484 OCCURRENCES 260 SPECIES

OVERVIEW 2 TREATMENTS METRICS REFERENCE TAXON

355 GEOREFERENCED RECORDS

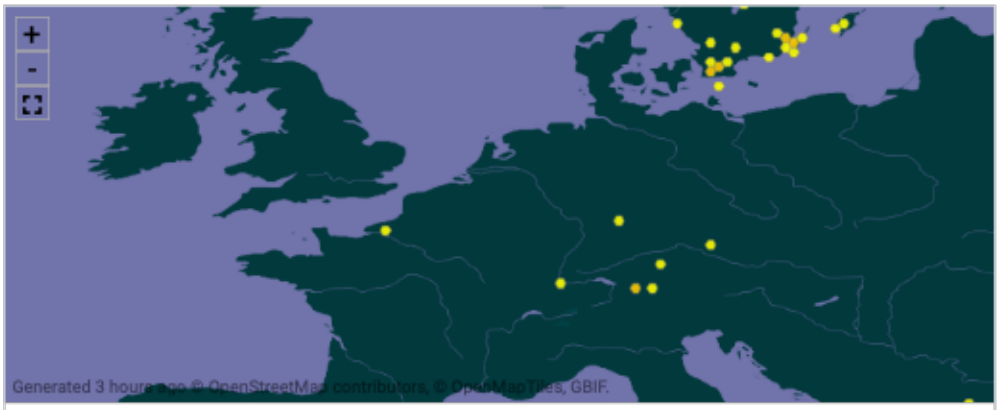

Any year 1877 - 2023 EXPLORE AREA

APPEARS IN 21 CHECKLIST DATASETS:

GBIF Backbone Taxonomy
As *Omphale* Haliday, 1833

Catalogue of Life Checklist
As *Omphale*

NCBI Taxonomy
As *Omphale*

The European Nucleotide Archive (ENA) taxonomy
As *Omphale*

International Barcode of Life project (iBOL) Barcode Index Numbers (BINs)
As *Omphale*

Figure S129: Global Biodiversity Information Facility (GBIF) Webpage *Omphale*

Sanger_ID: SQ_2022_057_022
Data_ID: KA_310521_C10A (conventional LED 4000K)

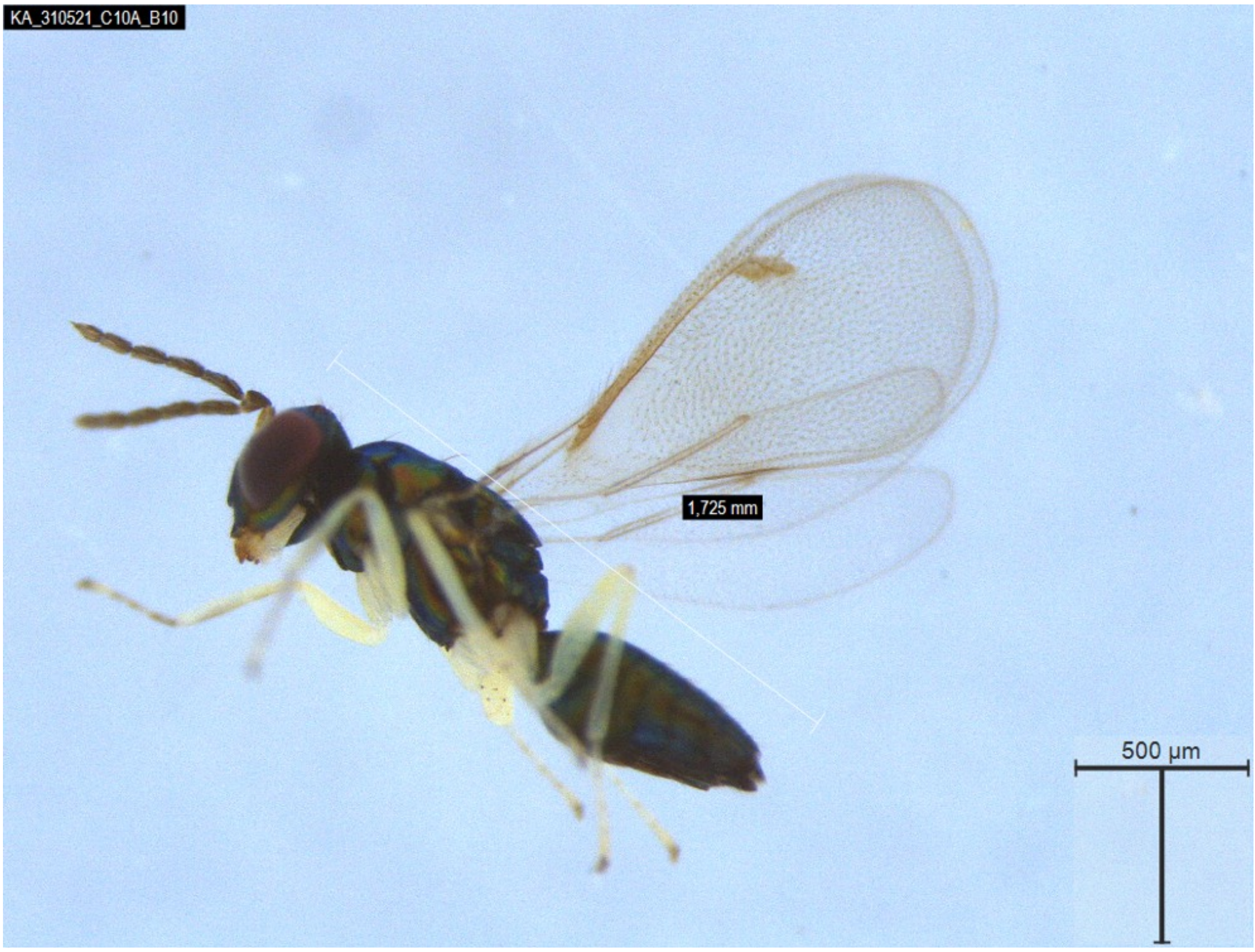

Figure S130: *Omphale sp.*

Pachycrepoideus vindemmiae (Pteromalidae)

Sanger_ID: SQ_2022_057_061
Data_ID: KA_171022_C9A (tailored LED 4000K)

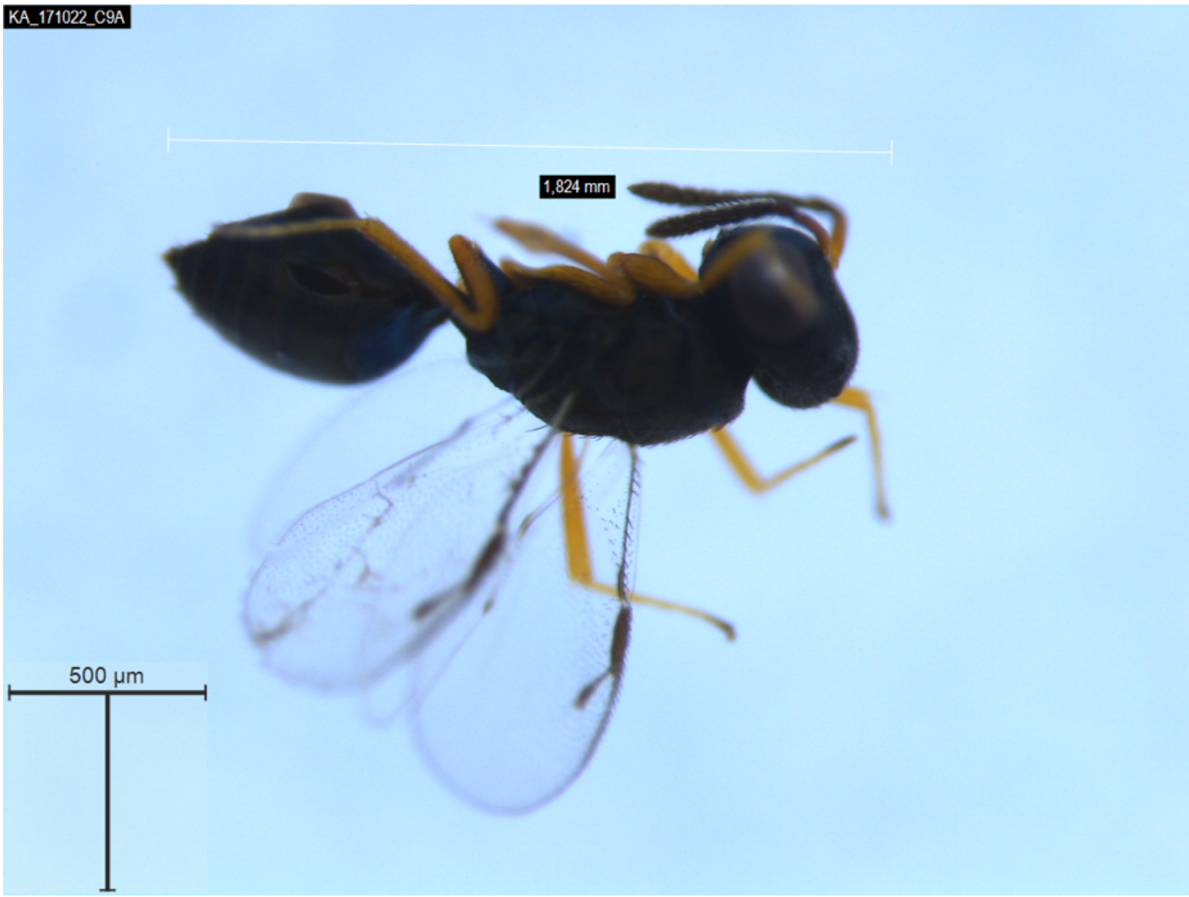

Figure S131: *Pachycrepoideus vindemmiae*

Proctotrupinae sp. (Proctotrupidae)

17/11/2023, 14:27 Parthenocodrus elongatus (Haliday, 1839)

SPECIES | ACCEPTED

Parthenocodrus elongatus (Haliday, 1839)

source: Taxon list of Hymenoptera from Germany compiled in the context of the GBOL project

Basionym: *Proctotrupes elongatus* Haliday, 1839

354 OCCURRENCES

OVERVIEW METRICS REFERENCE TAXON

1 OCCURRENCE WITH IMAGES

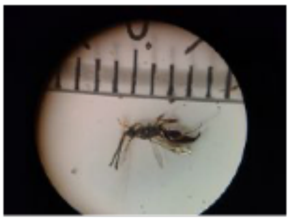

92 GEOREFERENCED RECORDS

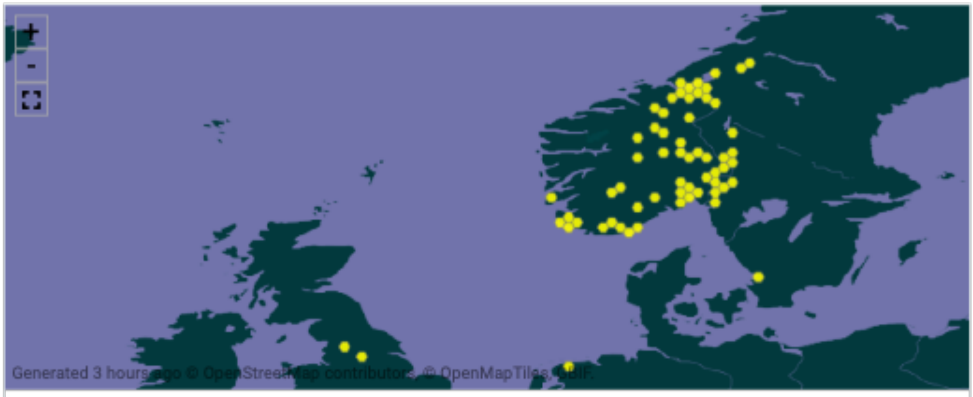

Any year 1999 - 2022 EXPLORE AREA

NAME USAGES APPLIED TO OCCURRENCES IN GBIF

https://www.gbif.org/species/4503996 1/4

Figure S132: Global Biodiversity Information Facility (GBIF) Webpage *Parthenocodrus elongatus*

SPECIES | SYNONYM

Phaenoserphus calcar (Haliday, 1839)

source: United Kingdom Species Inventory (UKSI)

Synonym of *Phaneroserphus calcar* (Haliday, 1839)

2 OCCURRENCES

OVERVIEW METRICS REFERENCE TAXON

1 GEOREFERENCED RECORD

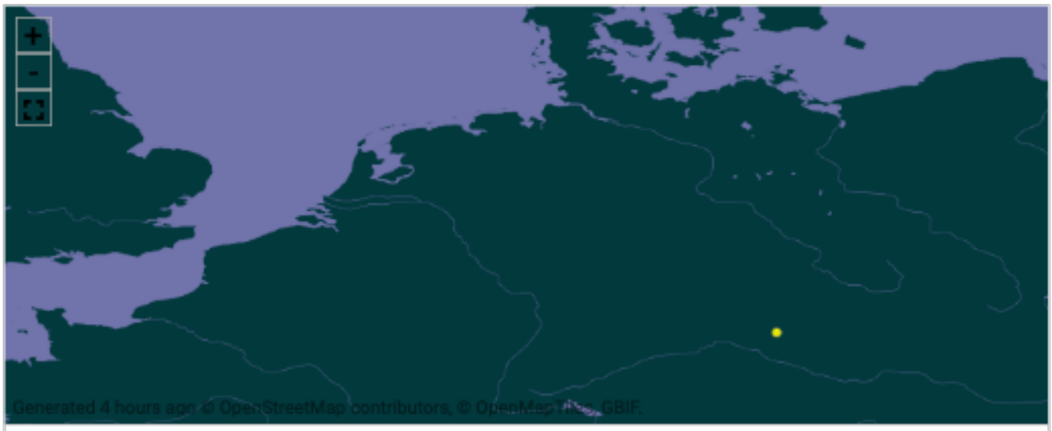

Year 2014 EXPLORE AREA

Issues: Basionym relation derived

APPEARS IN 5 CHECKLIST DATASETS:

GBIF Backbone Taxonomy
As *Phaenoserphus calcar* (Haliday, 1839)

International Barcode of Life project (iBOL) Barcode Index Numbers (BINs)
As *Phaenoserphus calcar*

TAXREF
As *Phaenoserphus calcar* (Haliday, 1839)

United Kingdom Species Inventory (UKSI)
As *Phaenoserphus calcar* (Haliday, 1839)

Figure S133: Global Biodiversity Information Facility (GBIF) Webpage *Phaenoserphus calar*

Sanger_ID: SQ_2022_057_026
Data_ID: HA_310822_C6A (conventional HPS 2000K)

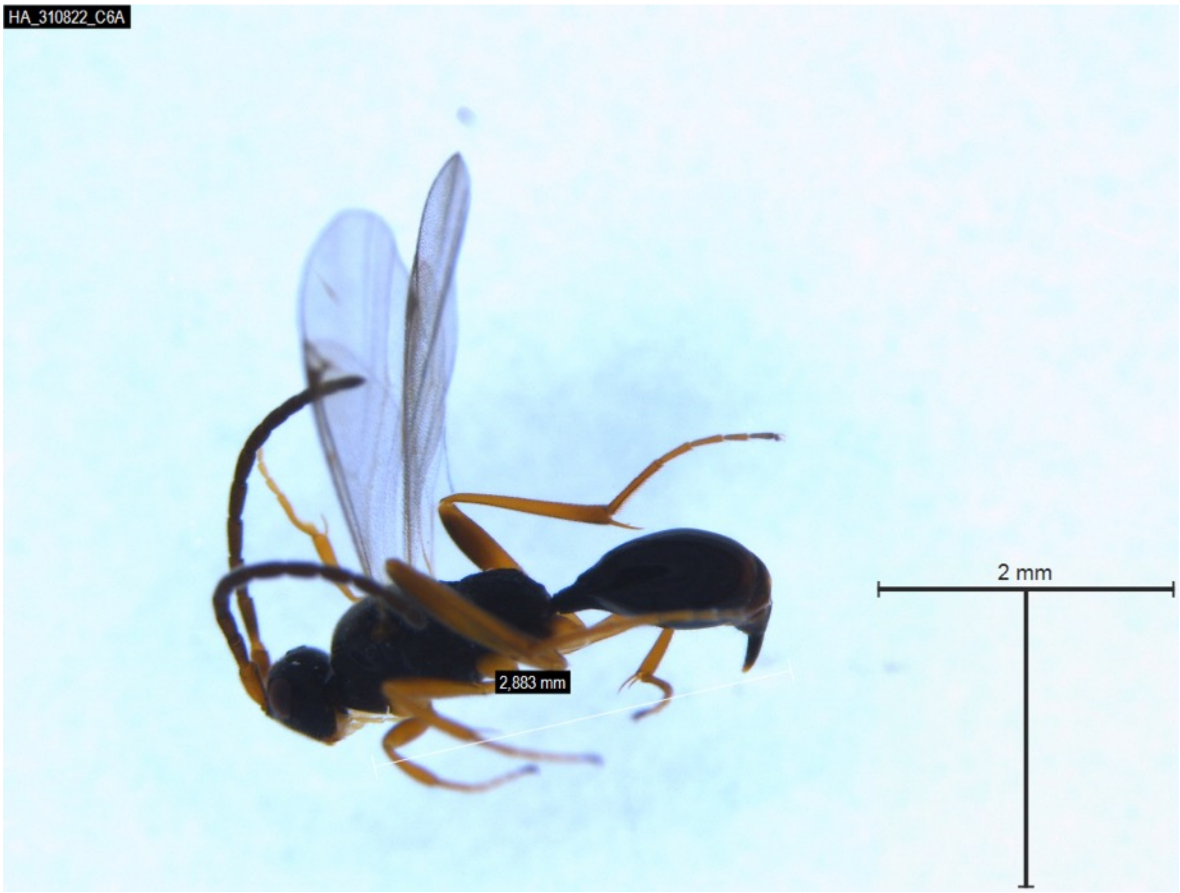

Figure S134: *Proctotrupinae sp.*

Sanger_ID: SQ_2022_057_057
Data_ID: HA_120621_C7A (conventional HPS 2000K)

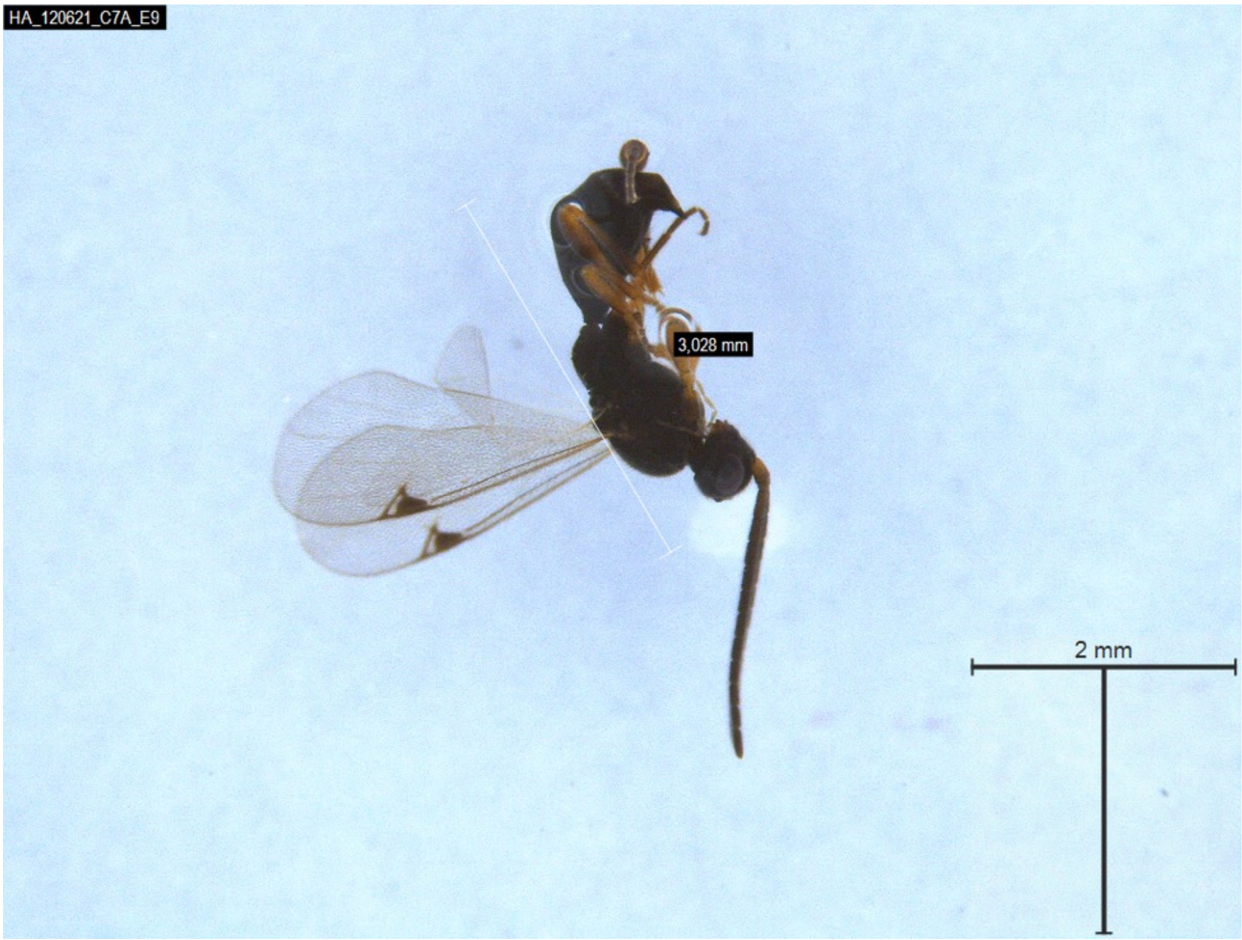

Figure S135: *Proctotrupinae sp.*

Sanger_ID: SQ_2022_057_063
Data_ID: HA_240822_C6A (conventional HPS 2000K)

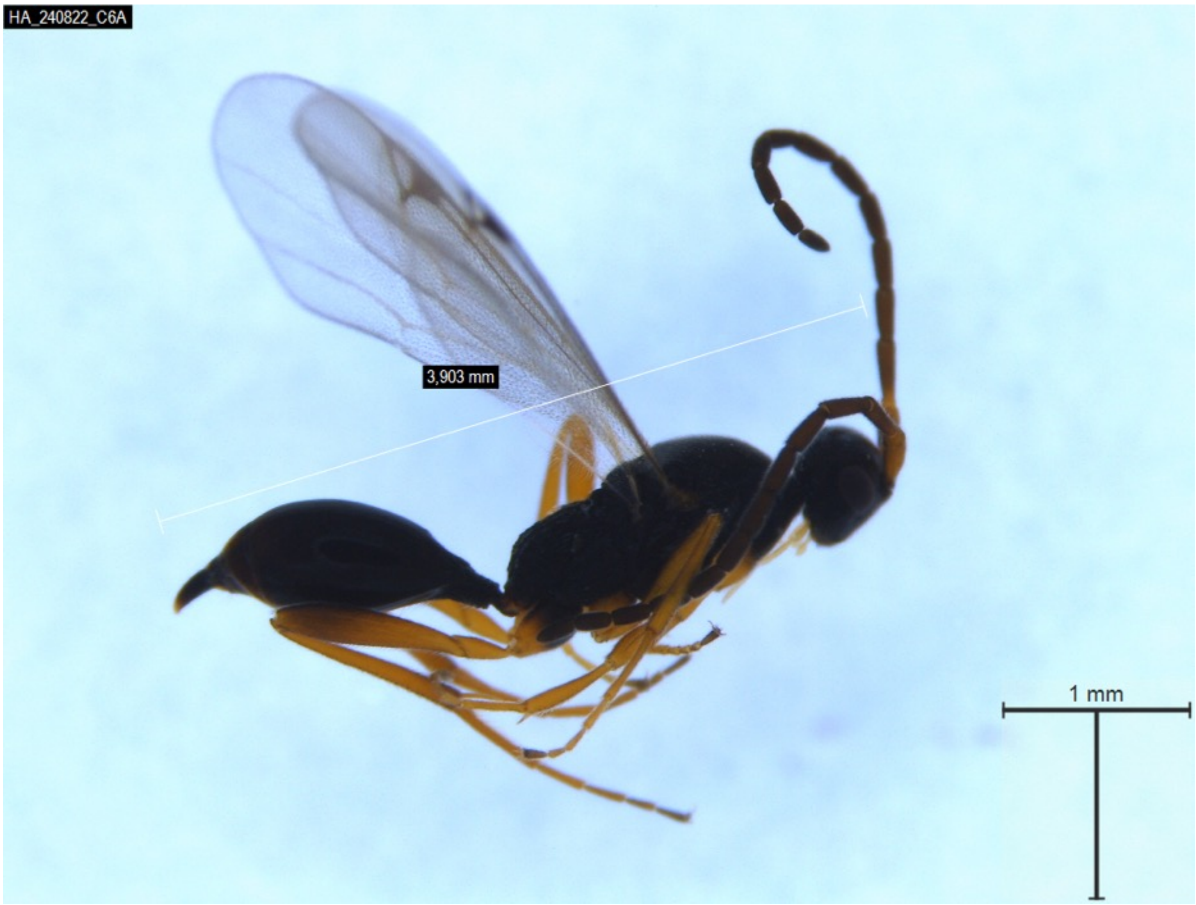

Figure S136: *Proctotrupinae sp.*

Sanger_ID: SQ_2022_057_004
Data_ID: HA_240822_C2A (conventional HPS 2000K)

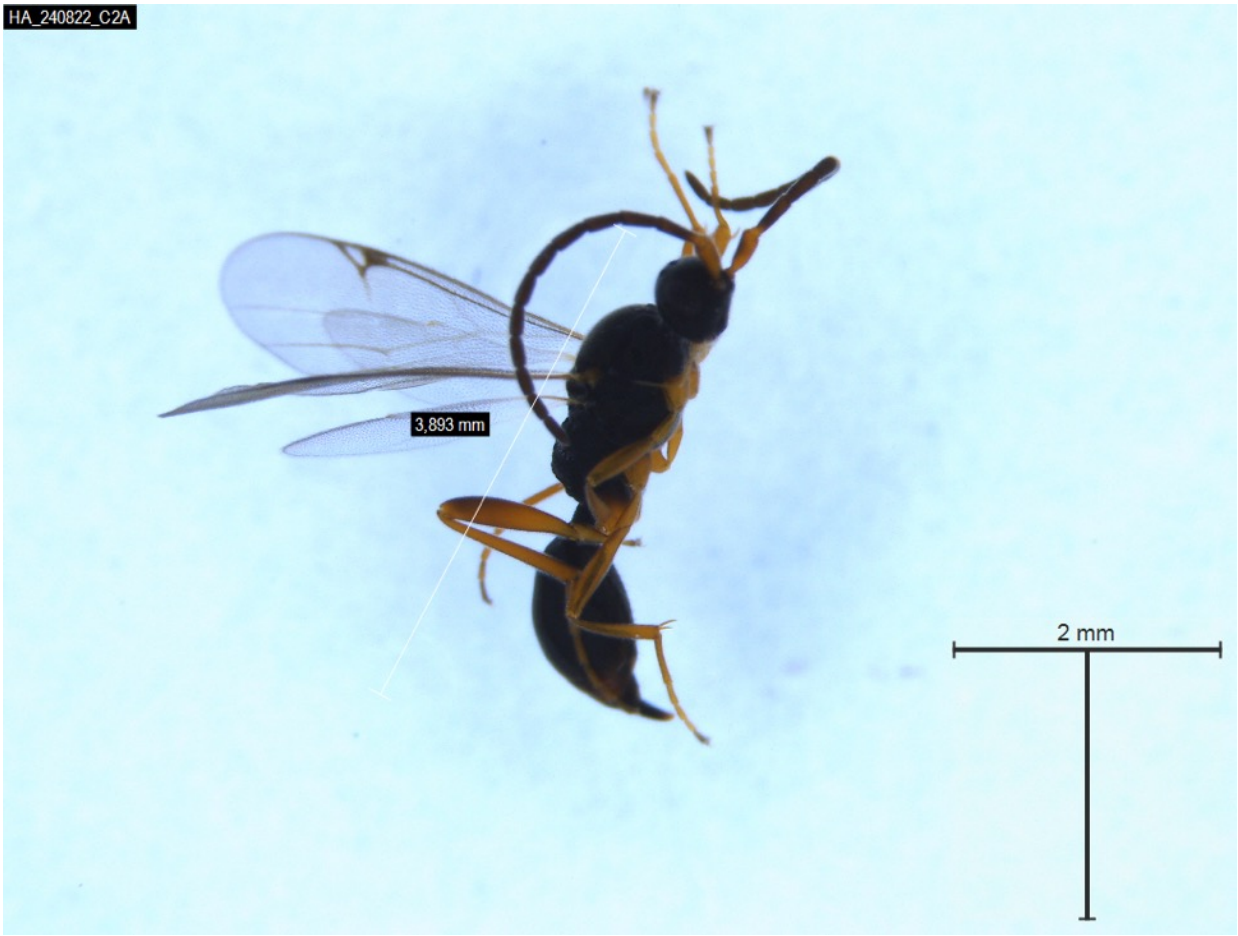

Figure S137: *Proctotrupinae sp.*

Sanger_ID: SQ_2022_057_016
Data_ID: HA_240822_C5B (conventional HPS 2000K)

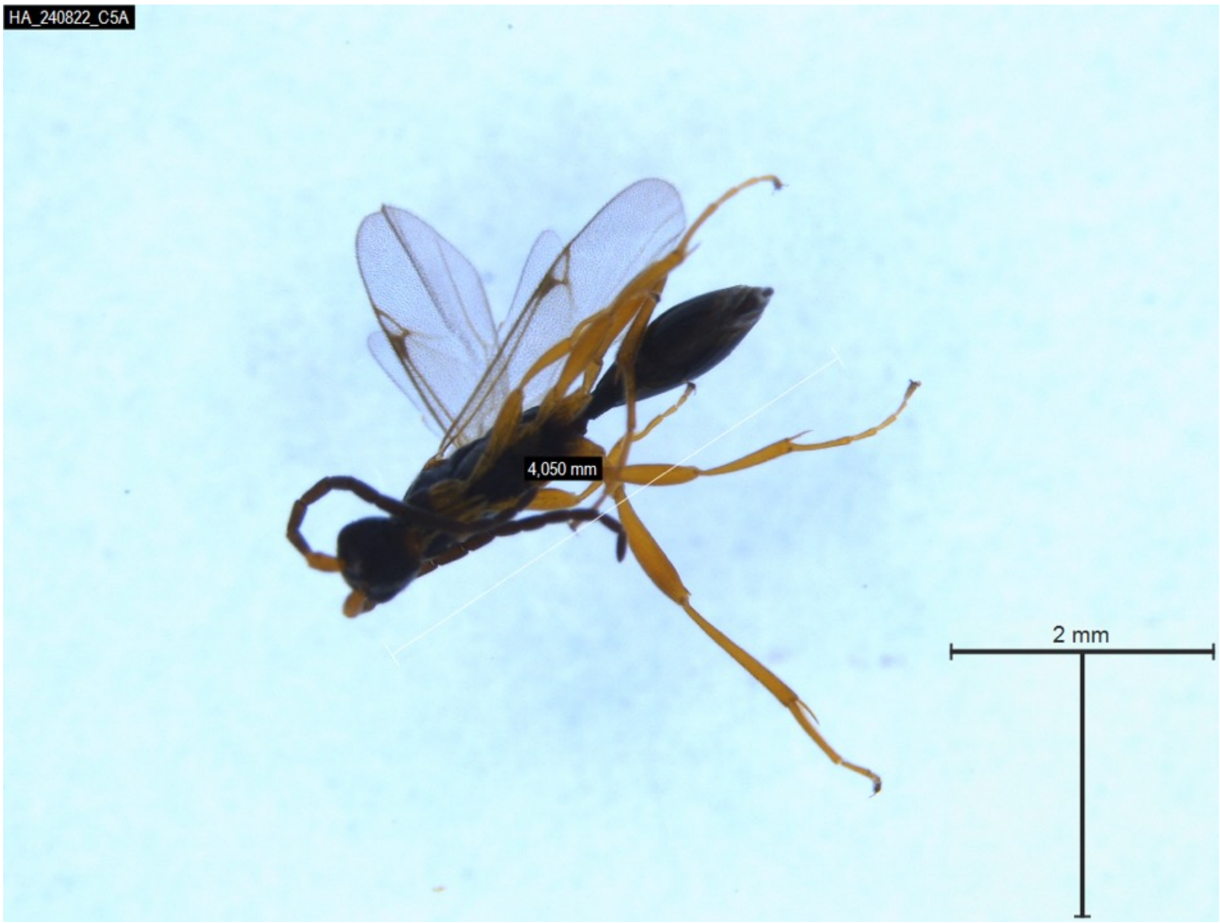

Figure S138: *Proctotrupinae sp.*

Sanger_ID: SQ_2022_057_075
Data_ID: HA_240822_C6B (conventional HPS 2000K)

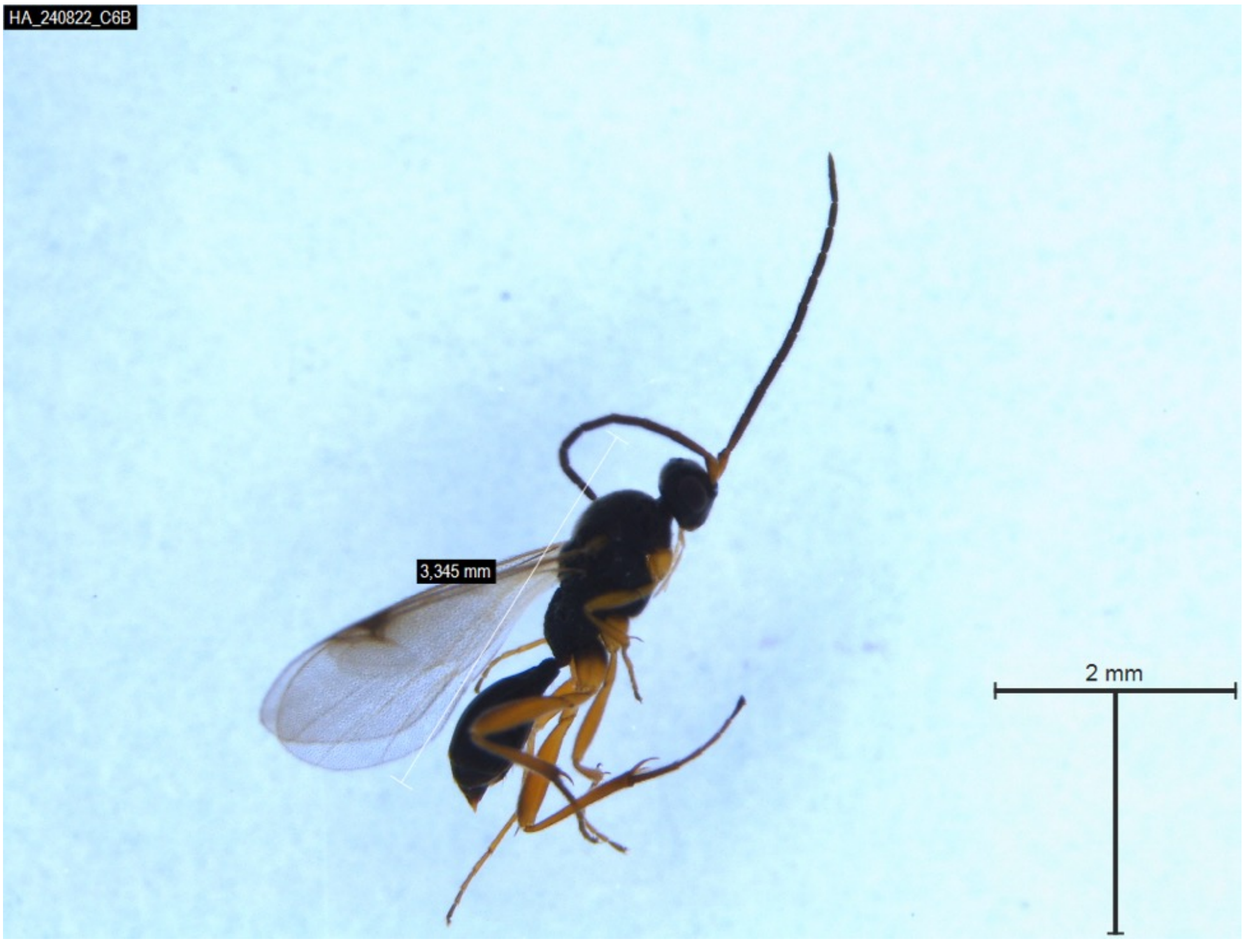

Figure S139: *Proctotrupinae sp.*

Sanger_ID: SQ_2022_057_087
Data_ID: HA_240822_C6C (conventional HPS 2000K)

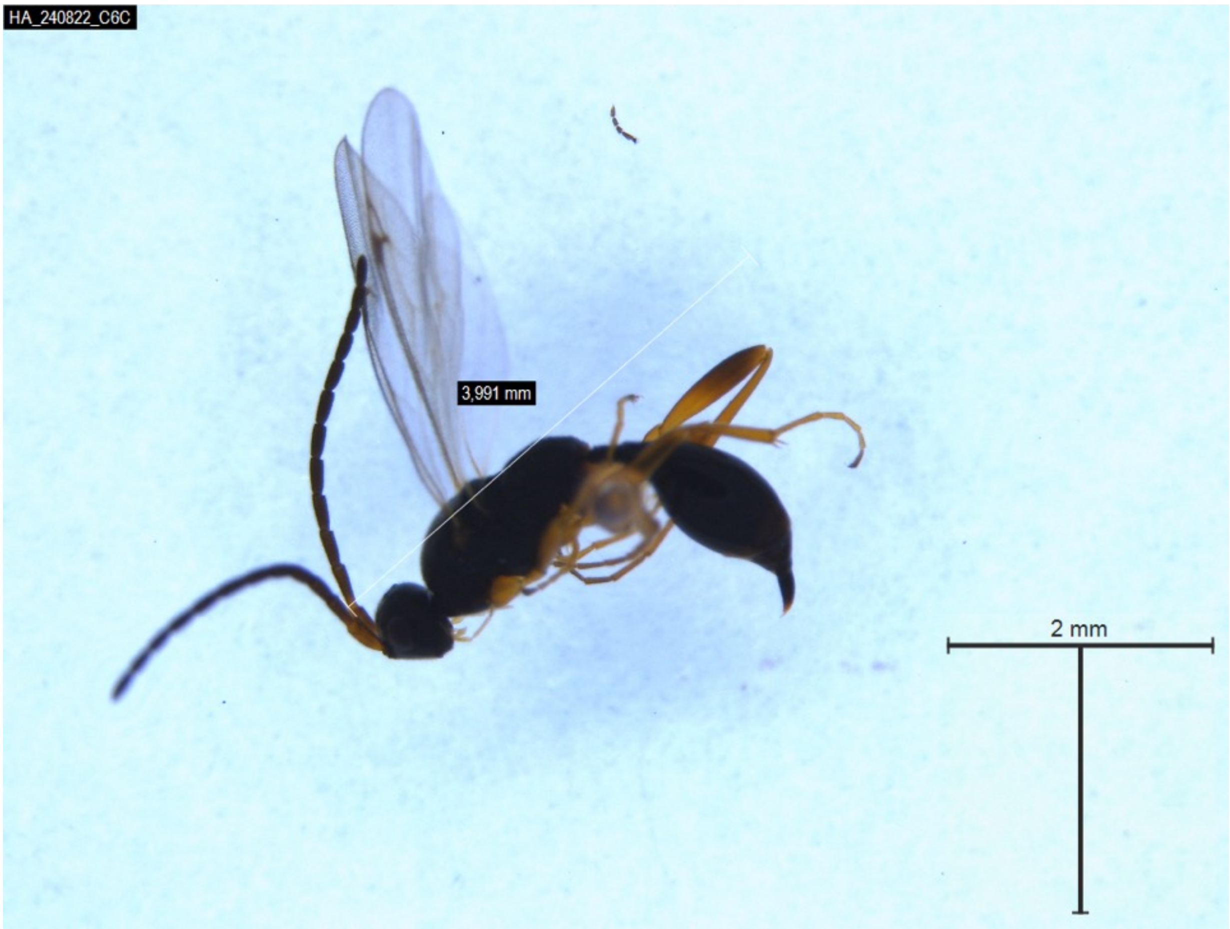

Figure S140: *Proctotrupinae sp.*

Platygastridae sp. (Platygastridae)

Data_ID: HA_180522_C1A (tailored LED 2700K)
Sanger ID: SQ_2022_057_017

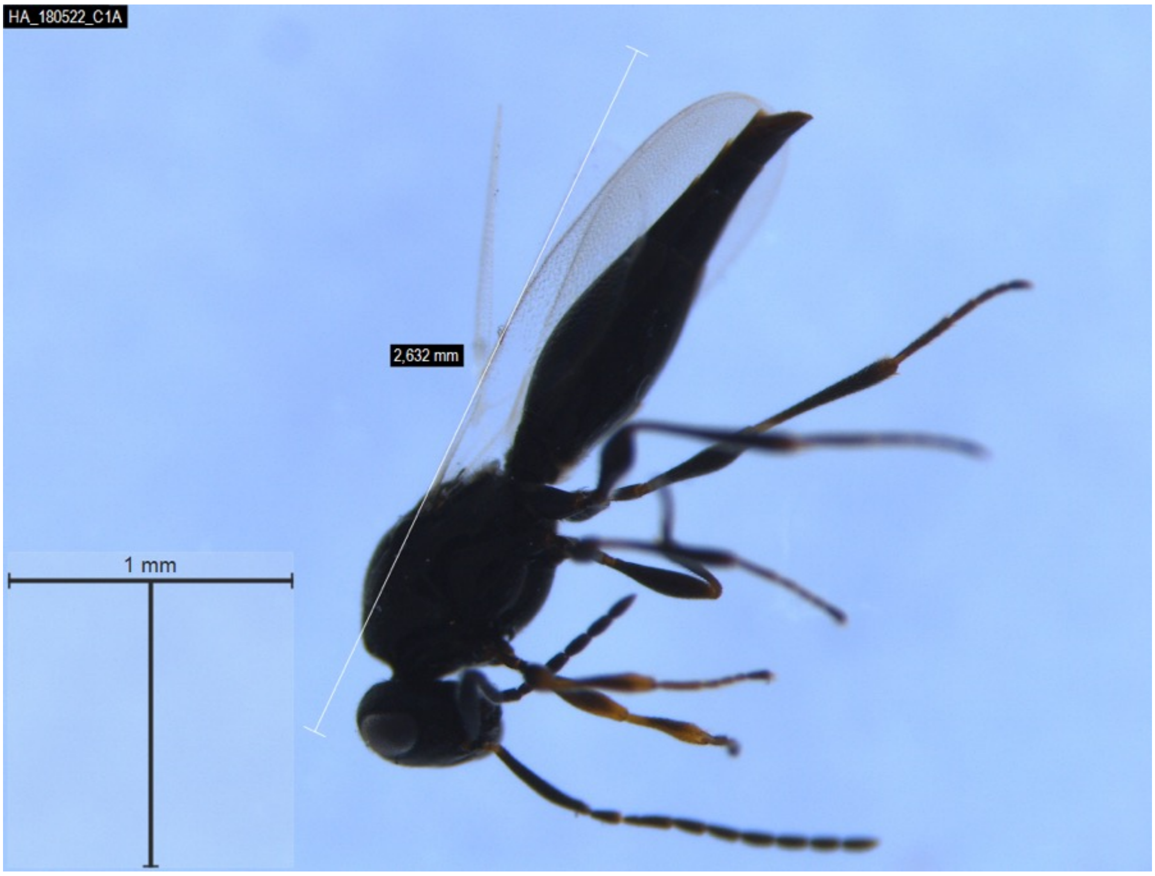

Figure S141: *Platygastridae sp.*

Proctotrupes sp. (Proctotrupidae)

GENUS | ACCEPTED

Proctotrupes Latreille, 1796

Published in: *Précis Caract. Ins.*: 108

source: Taxon list of Hymenoptera from Germany compiled in the context of the GBOL project

469 OCCURRENCES 6 SPECIES

OVERVIEW METRICS REFERENCE TAXON

48 OCCURRENCES WITH IMAGES

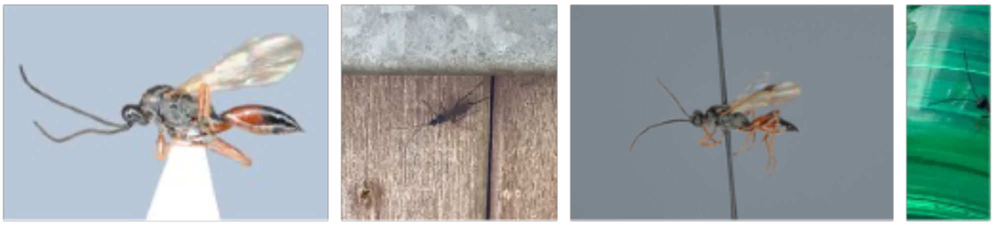

232 GEOREFERENCED RECORDS

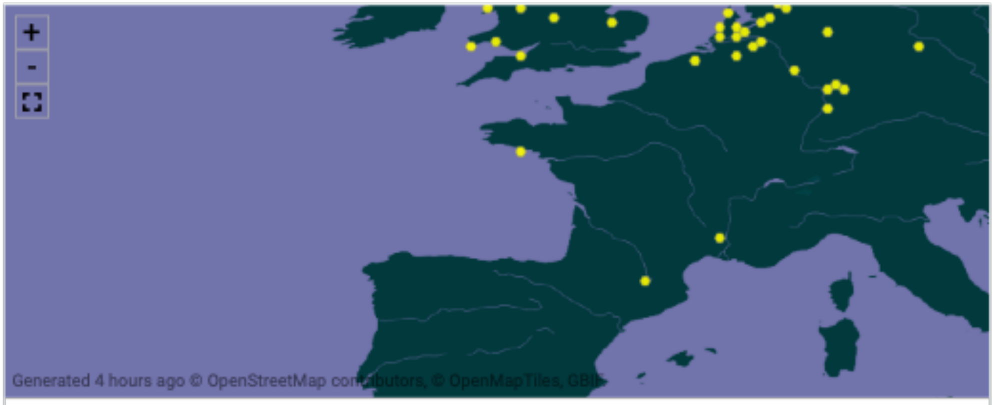

Any year 1800 - 2023 EXPLORE AREA

APPEARS IN 19 CHECKLIST DATASETS:

GBIF Backbone Taxonomy
As *Proctotrupes* Latreille, 1796

NCBI Taxonomy
As *Proctotrupes*

The European Nucleotide Archive (ENA) taxonomy

Figure S142: Global Biodiversity Information Facility (GBIF) Webpage *Proctotrupes*

Sanger_ID: SQ_2022_057_080
Data_ID: BR_280622_C23A (tailored LED 2700K)

BOLD Grade ID: 93.7%
Proctotrupidae
BOLD process ID:
JBHCI743-11
BIN: BOLD:AUU9841
NCBI Grade ID:92.9 %
Procotrupes bistratus
NCBI Accession ID:
KF936644

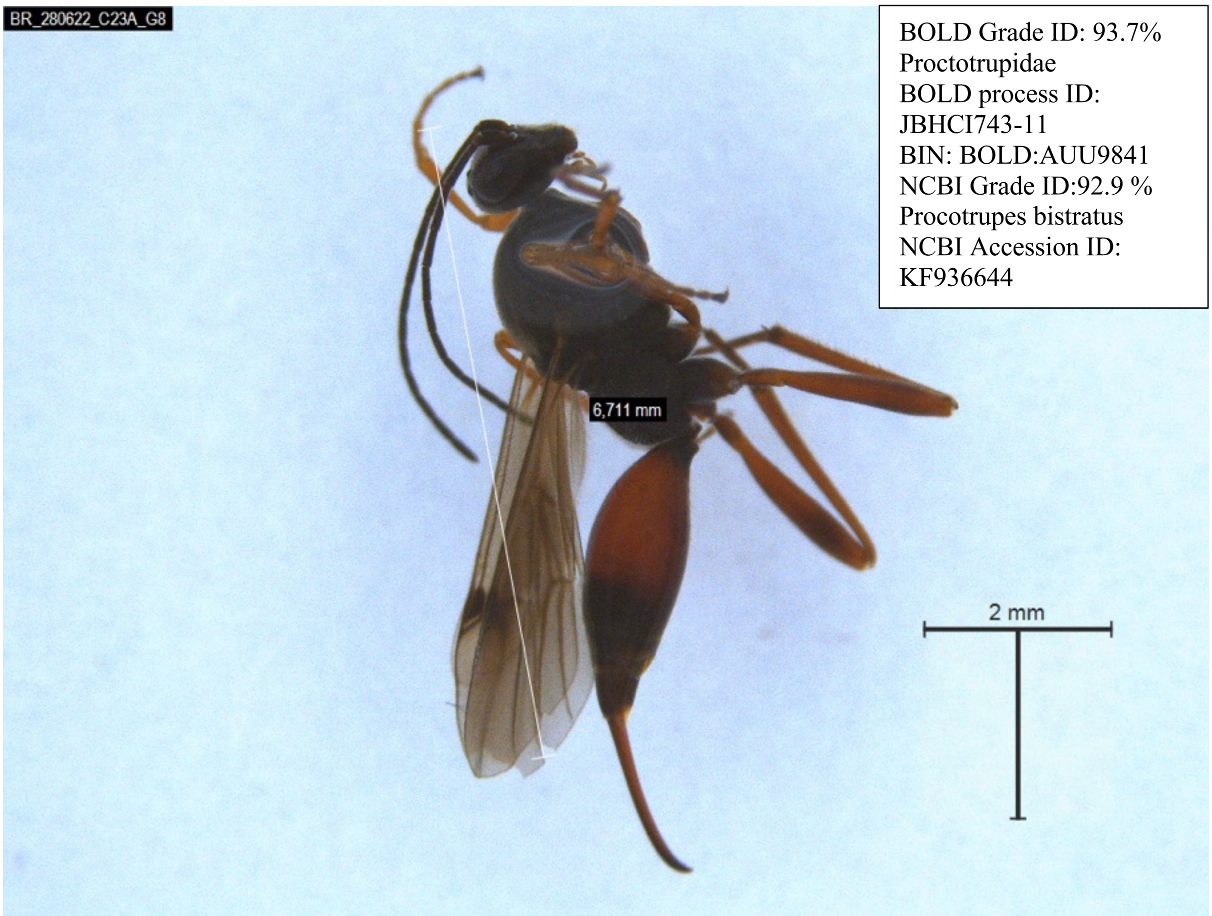

Figure S143: *Proctotrupes sp.*

Sanger_ID: SQ_2022_057_107
Data_ID: BR_280622_C19A (tailored LED 2700K)

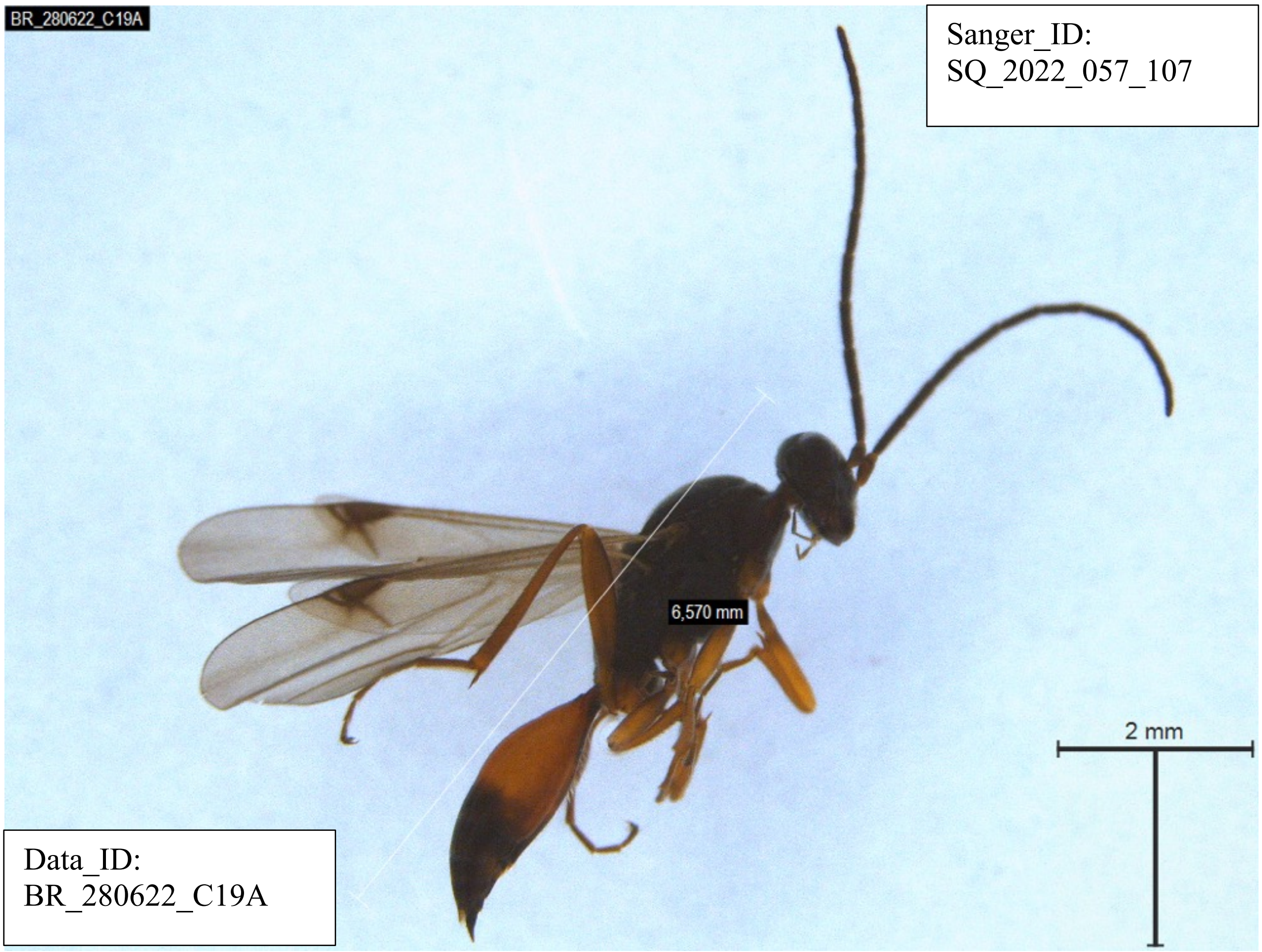

Figure S144: *Proctotrupes sp.*

Scelionidae sp1. (Scelionidae)

FAMILY | ACCEPTED

Scelionidae

In: GBIF Backbone Taxonomy

Scelionid wasps In English

314,291 OCCURRENCES 3,555 SPECIES

OVERVIEW METRICS

27,654 OCCURRENCES WITH IMAGES

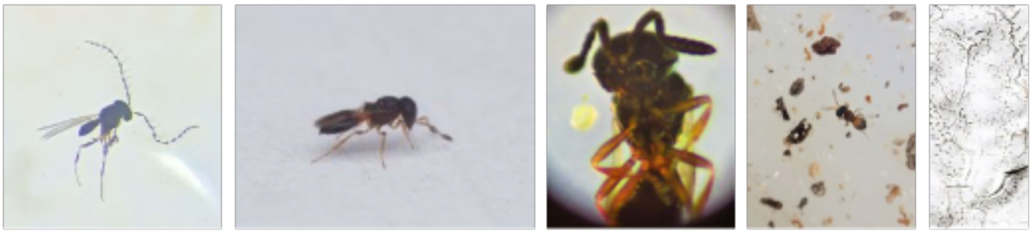

299,836 GEOREFERENCED RECORDS

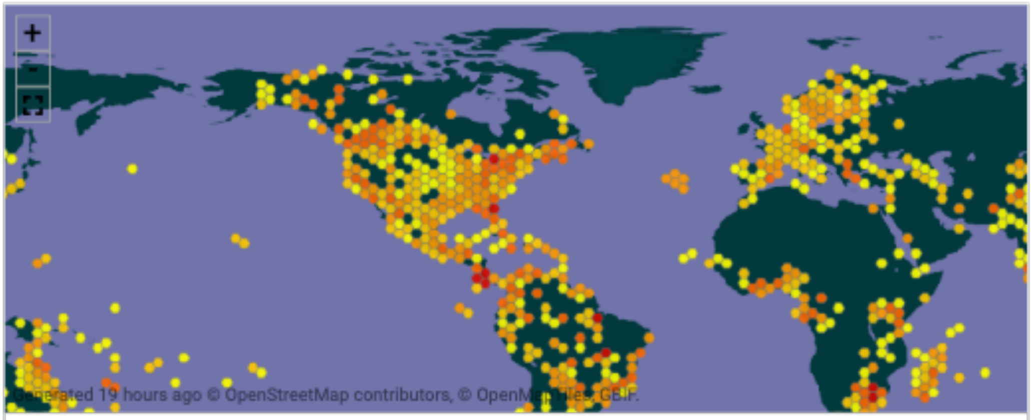

Any year 1664 - 2023 EXPLORE

VERNACULAR NAMES

Scelionid wasps In English
sources: Catalogue of Life Checklist + 1 more dataset

Scelionids In English
sources: Catalogue of Life Checklist + 1 more dataset

Äggmärkarsteklar In Swedish
source: Dyntaxa. Svensk taxonomisk databas

Figure S145: Global Biodiversity Information Facility (GBIF) Webpage *Scelionidae Succinctus*

Sanger_ID: SQ_2022_057_014
Data_ID: KA_190722_C14A (conventional LED 4000K)

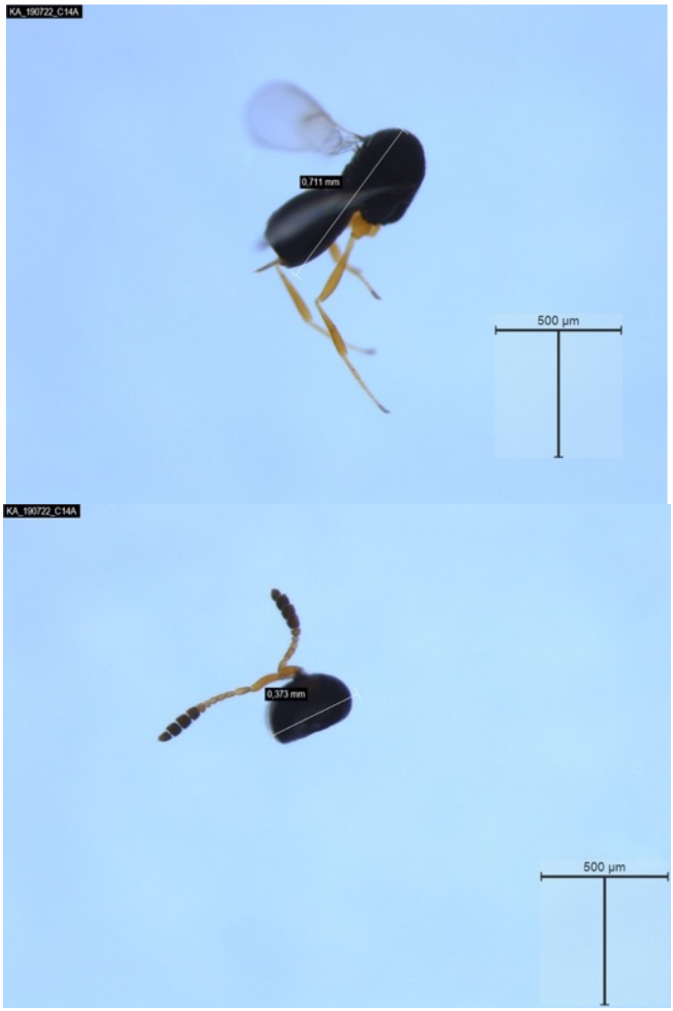

Figure S146: *Scelionidae sp1.*

Scelionidae sp2. (Scelionidae)

Sanger_ID: SQ_2022_057_094
Data_ID: KA_300621_C16A (conventional LED 4000K)

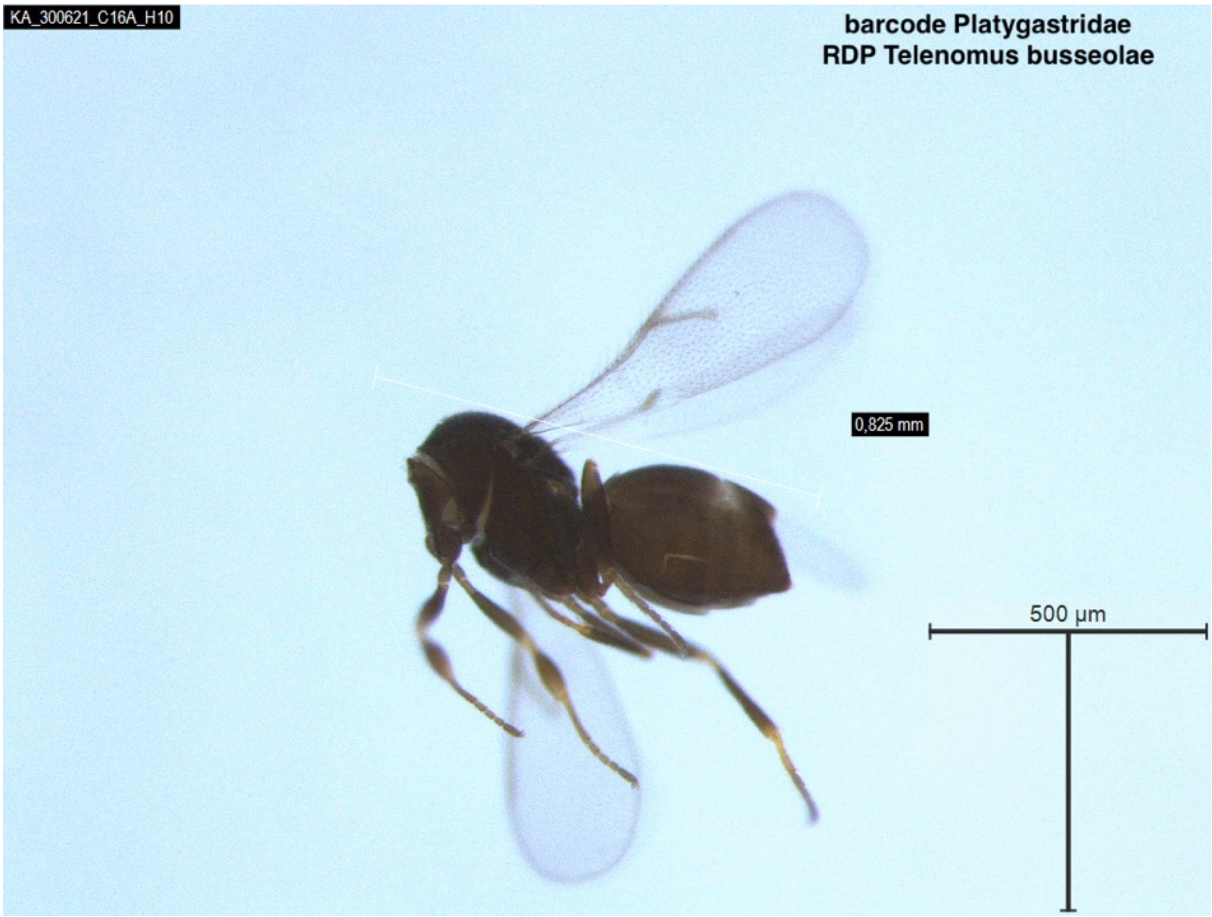

Figure S147: *Scelionidae sp2.*

Stenomacrus affinitor (Ichneumonidae)

17/11/2023, 14:57 Stenomacrus affinitor Aubert, 1981

SPECIES | ACCEPTED

Stenomacrus affinitor Aubert, 1981

Published in: Aubert, J.F. Revision des Ichneumonides Stenomacrus sensu lato. Mitteilungen Munchener Entomologischen Gesellschaft. 71:139-159. (1981).
source: Taxapad Ichneumonoidea

62 OCCURRENCES 3 INFRASPECIES

OVERVIEW METRICS REFERENCE TAXON

17 GEOREFERENCED RECORDS

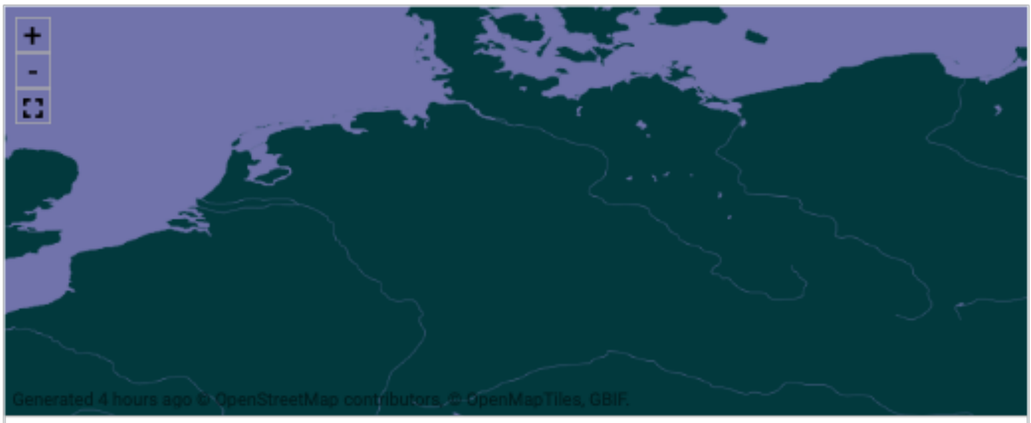

Any year 2003 - 2021 EXPLORE AREA

NAME USAGES APPLIED TO OCCURRENCES IN GBIF

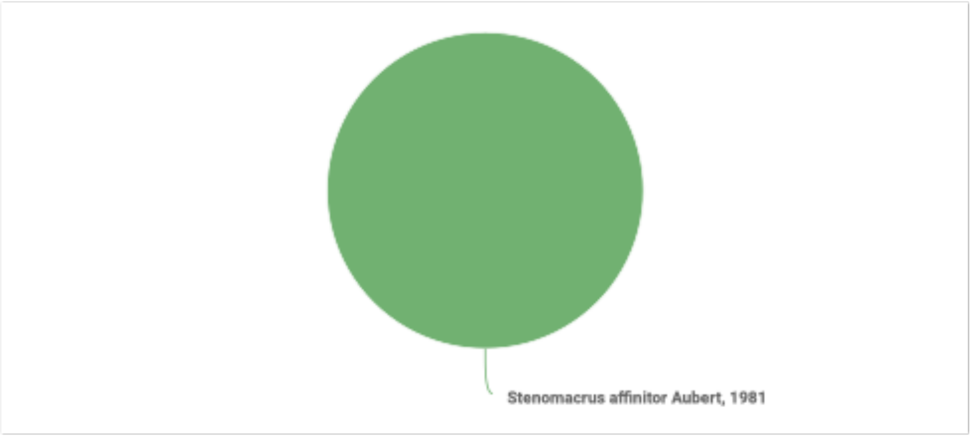

APPEARS IN 8 CHECKLIST DATASETS:

GBIF Backbone Taxonomy

https://www.gbif.org/species/1307987 1/3

Figure S148: Global Biodiversity Information Facility (GBIF) Webpage *Stenomacrus affinitor*

Sanger_ID: SQ_2022_057_060
Data_ID: BR_280622_C24A (conventional HPS 2000K)

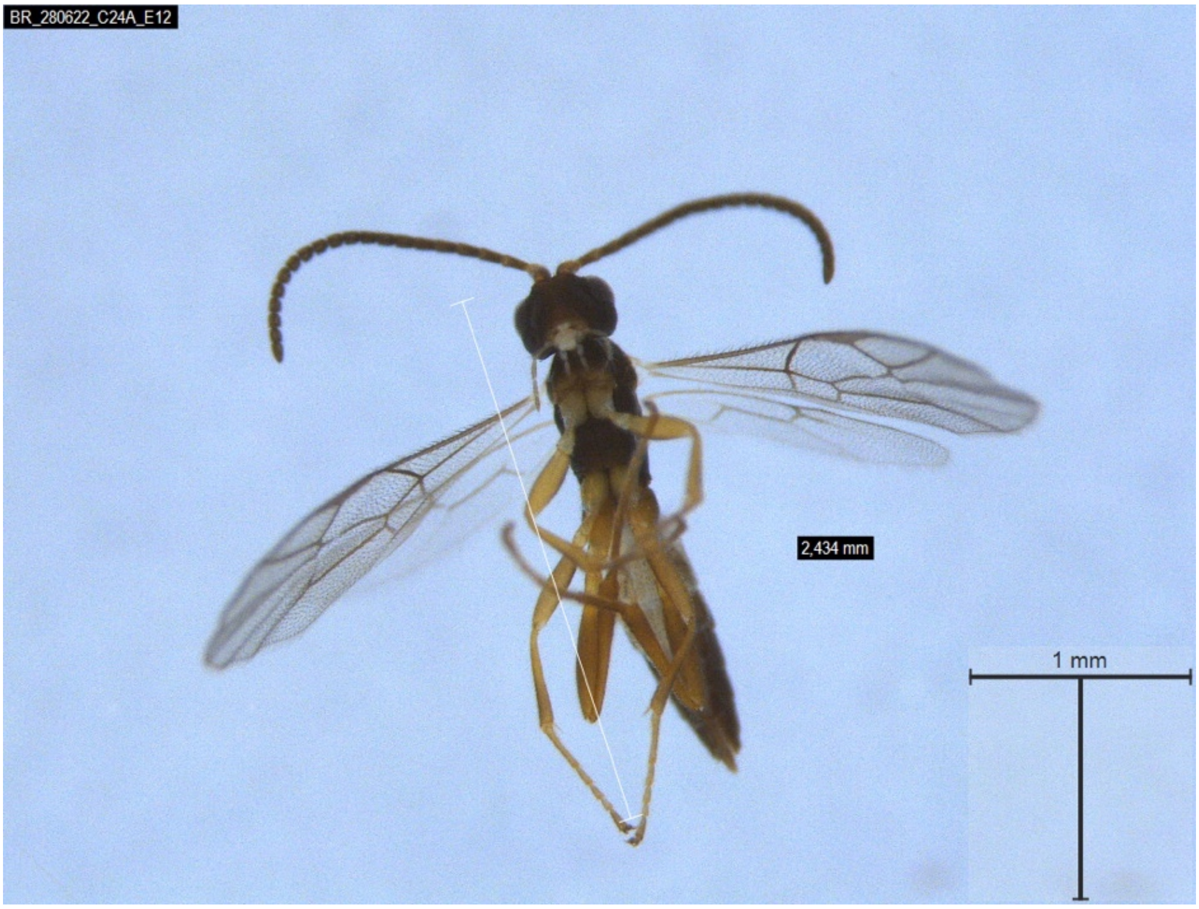

Figure S149: *Stenomacrus affinitor*

Stenomalina sp. (Pteromalidae)

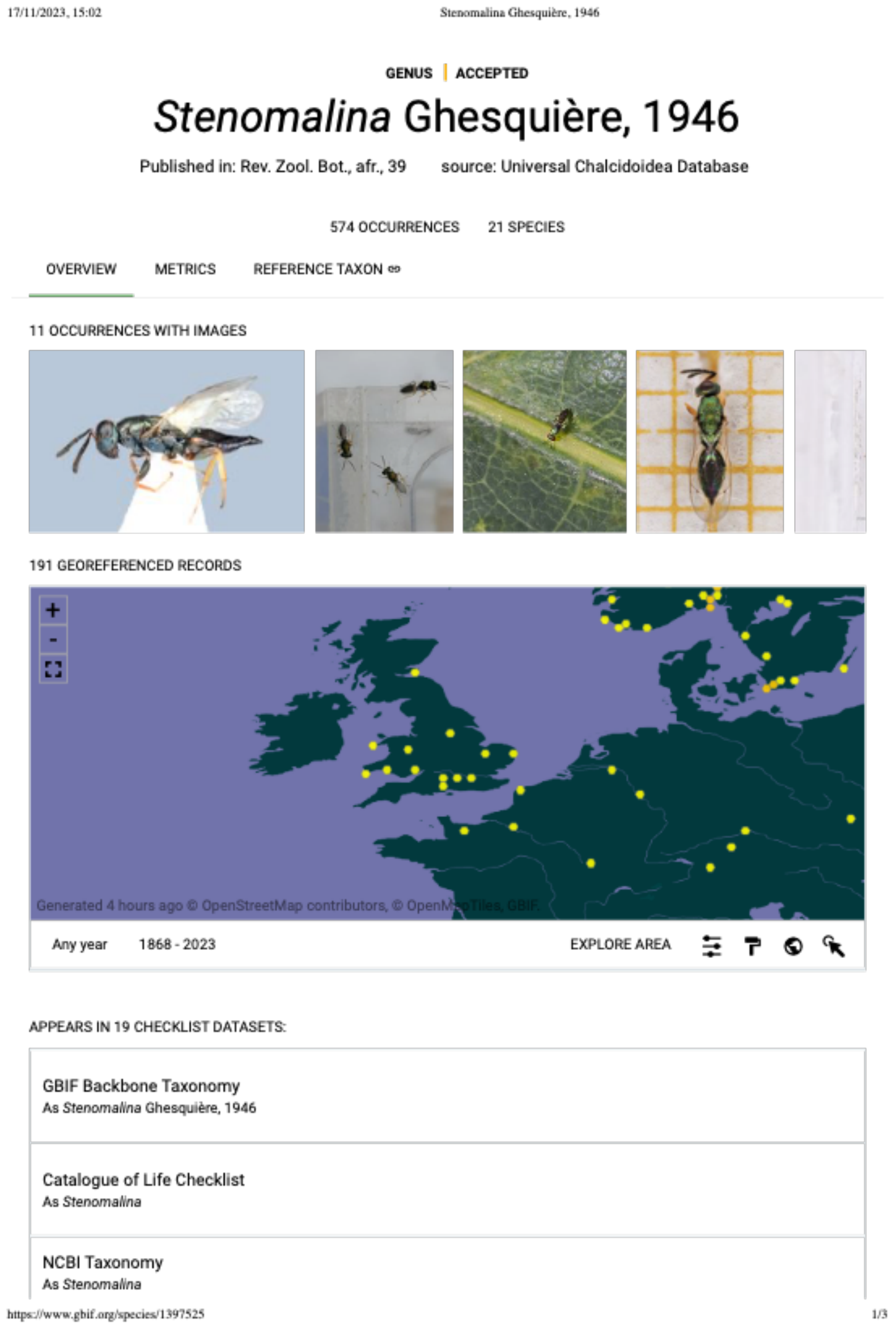
17/11/2023, 15:02 Stenomalina Ghesquière, 1946

GENUS | ACCEPTED

Stenomalina Ghesquière, 1946

Published in: Rev. Zool. Bot., afr., 39 source: Universal Chalcidoidea Database

574 OCCURRENCES 21 SPECIES

OVERVIEW METRICS REFERENCE TAXON

11 OCCURRENCES WITH IMAGES

191 GEOREFERENCED RECORDS

Generated 4 hours ago © OpenStreetMap contributors, © OpenMapTiles, GBIF.

Any year 1868 - 2023 EXPLORE AREA

APPEARS IN 19 CHECKLIST DATASETS:

GBIF Backbone Taxonomy
As *Stenomalina* Ghesquière, 1946

Catalogue of Life Checklist
As *Stenomalina*

NCBI Taxonomy
As *Stenomalina*

https://www.gbif.org/species/1397525 1/3

Figure S150: Global Biodiversity Information Facility (GBIF) Webpage *Acrotomus Succinctus*

Sanger_ID: SQ_2022_057_015
Data_ID: KA_270622_C14A (conventional LED 4000K)

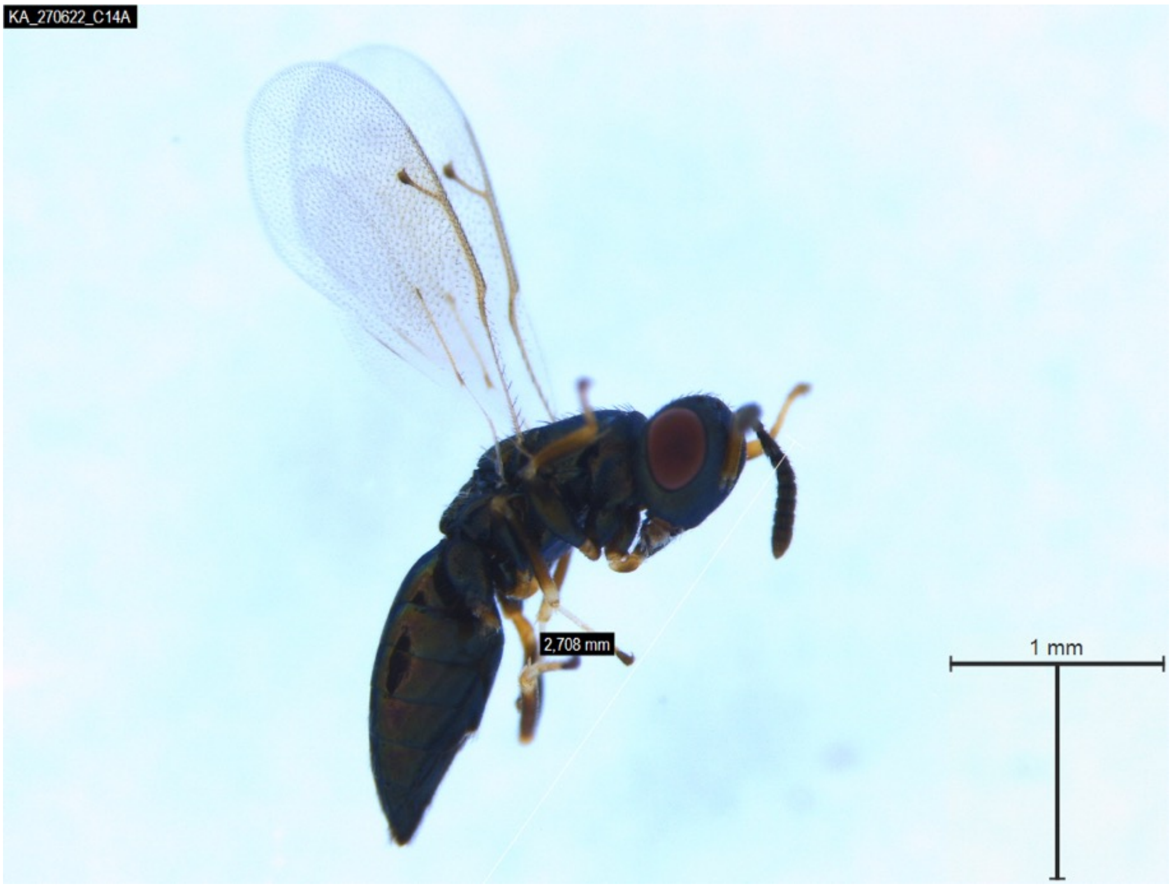

Figure S151: *Stenomalina sp.*

Synopeas sp. (Platygastridae)

GENUS | ACCEPTED

Synopeas Förster, 1856

Published in: Foerster, Arnold. 1856. Hymenopterologische Studien. II. Heft. Chalcidiae und Proctotrupii. Ernst ter Meer, Aachen.: 1-152.

In: GBIF Backbone Taxonomy

10,508 OCCURRENCES 310 SPECIES

OVERVIEW 4 TREATMENTS METRICS

52 OCCURRENCES WITH IMAGES

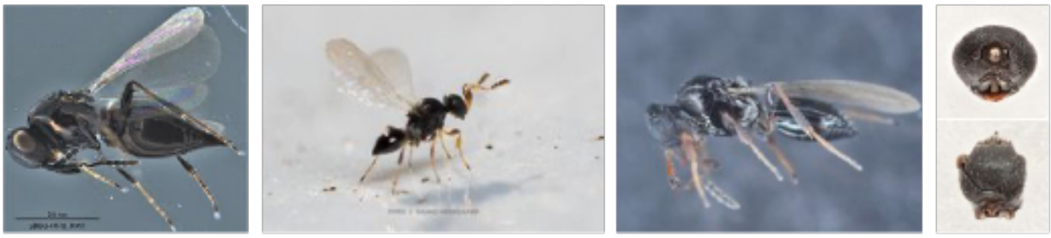

1,155 GEOREFERENCED RECORDS

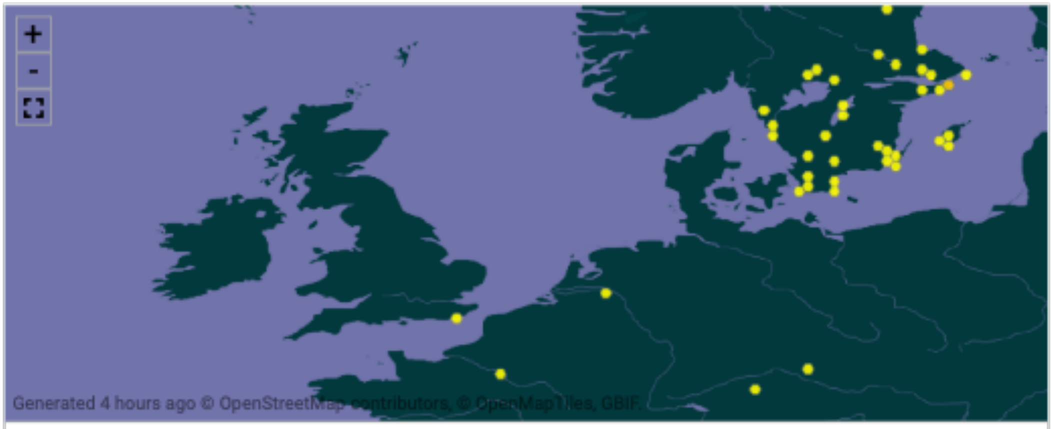

Any year 1824 - 2022 EXPLORE AREA

APPEARS IN 33 CHECKLIST DATASETS:

GBIF Backbone Taxonomy
As *Synopeas* Förster, 1856

Catalogue of Life Checklist
As *Synopeas* Förster, 1856

Figure S152: Global Biodiversity Information Facility (GBIF) Webpage *Synopeas*

Sanger_ID: SQ_2022_057_076
Data_ID: HA_240822_C3A (tailored LED 2000K)

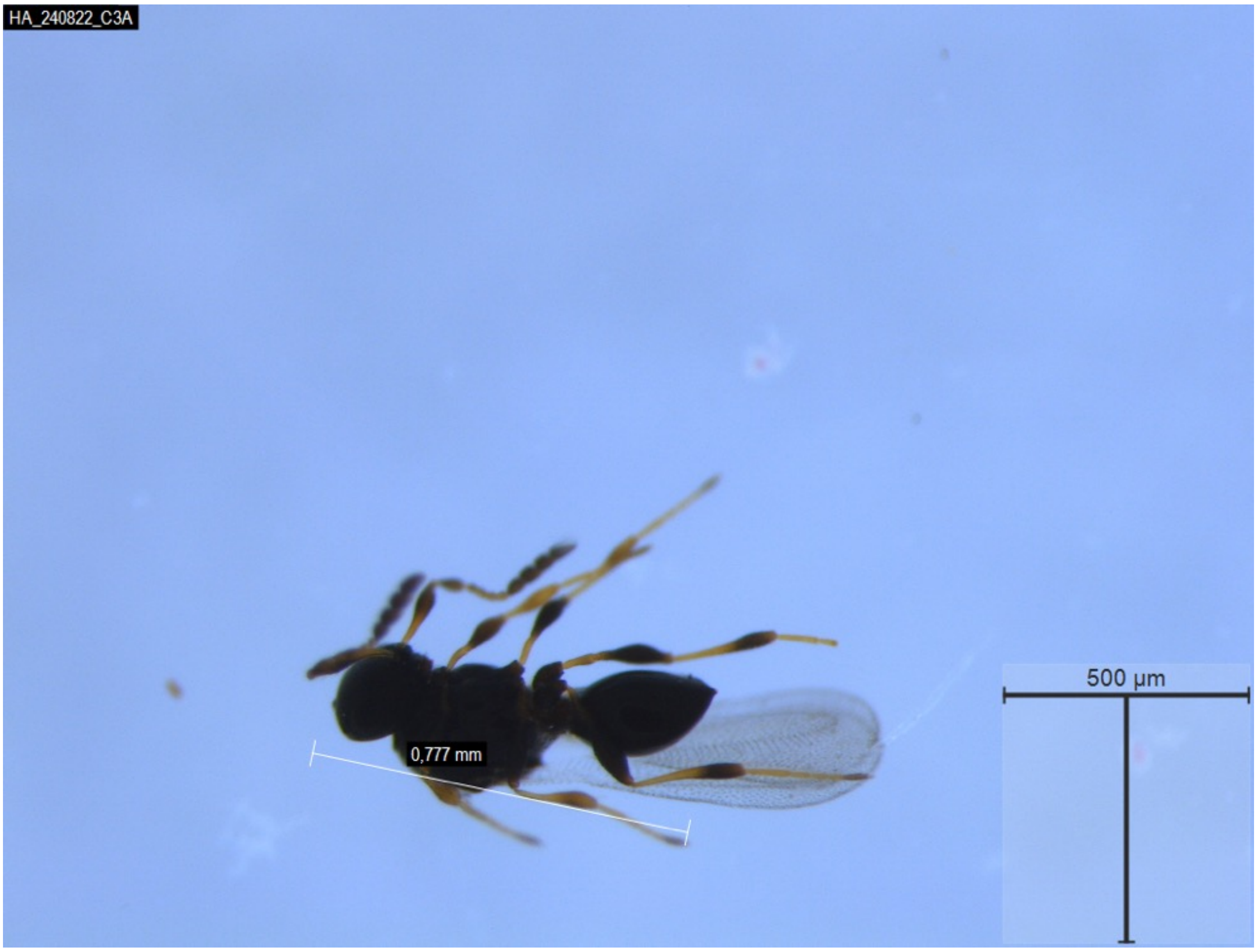

Figure S153: *Synopeas sp.*

Alysiinae sp. (Braconidae)

Sanger_ID: SQ_2022_057_095
Data_ID: HA_260722_C4A (tailored LED 2700K)

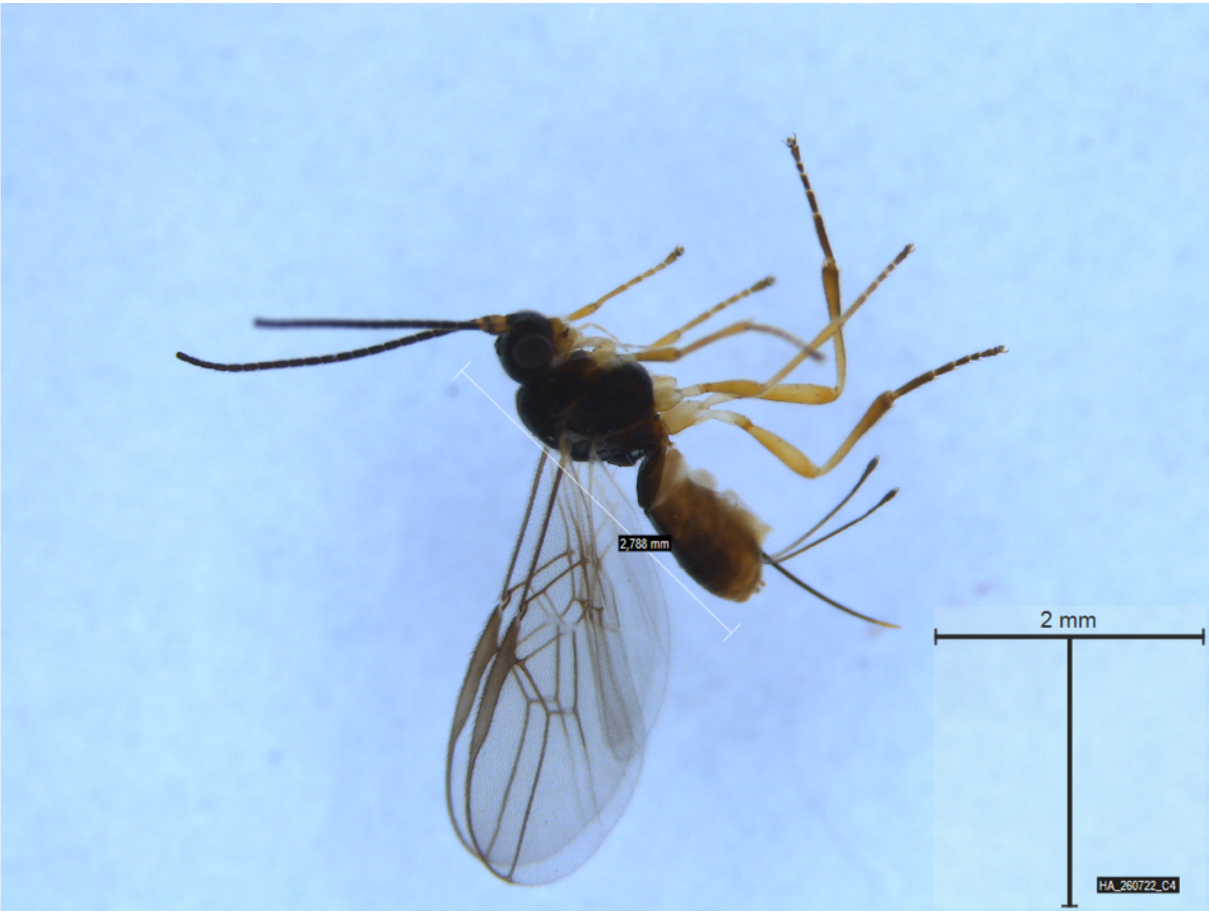

Figure S154: *Alysiinae sp.*

Temelucha (Ichneumonidae)

Sanger_ID: SQ_2022_057_099
Data_ID: KA_270622_C15A (conventional LED 4000K)

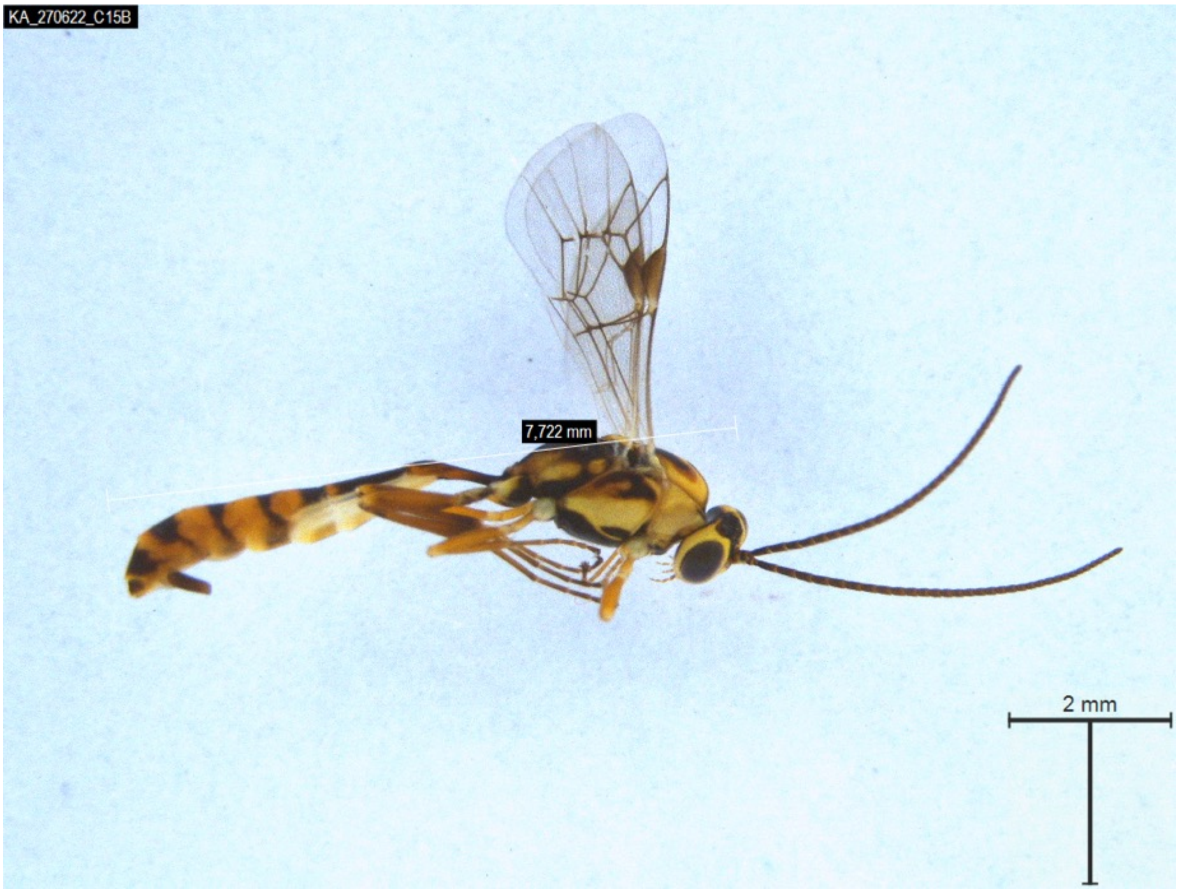

Figure S155: *Temelucha sp.*

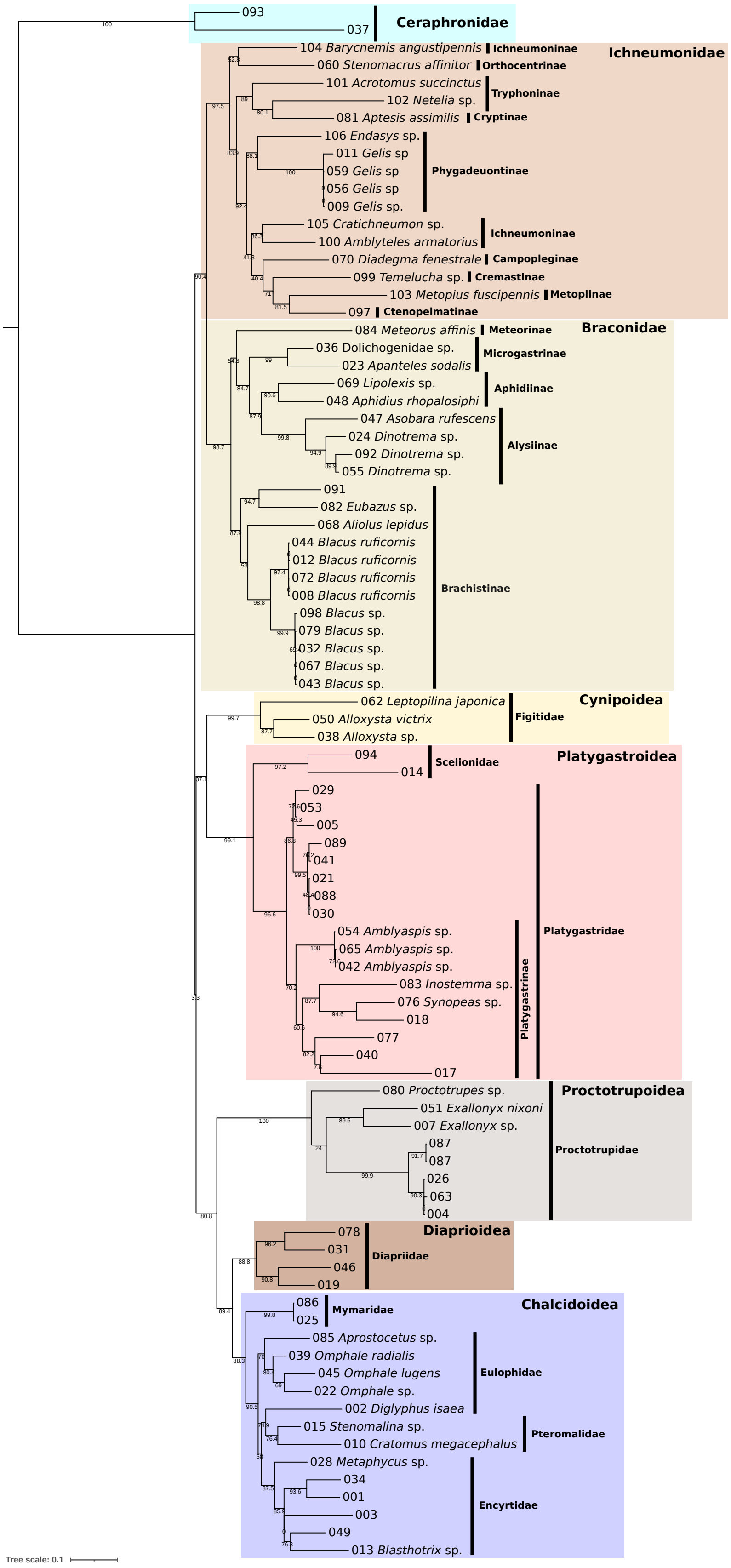

Ceraphronidae
093
037
Ichneumonidae
104 Barycnemis angustipennis
Ichneumoninae
060 Stenomacrus affinitor
Orthocentrinae
101 Acrotomus succinctus
102 Netelia sp.
Tryphoninae
081 Aptesis assimilis
Cryptinae
106 Endasys sp.
011 Gelis sp
059 Gelis sp
056 Gelis sp
009 Gelis sp.
Phygadeuontinae
105 Cratichneumon sp.
100 Amblyteles armatorius
Ichneumoninae
070 Diadegma fenestrale
Campopleginae
099 Temelucha sp.
Cremastinae
103 Metopius fuscipennis
Metopiinae
097
Ctenopelmatinae
Braconidae
084 Meteorus affinis
Meteorinae
036 Dolichogenidae sp.
023 Apanteles sodalis
Microgastrinae
069 Lipolexis sp.
048 Aphidius rhopalosiphi
Aphidiinae
047 Asobara rufescens
024 Dinotrema sp.
092 Dinotrema sp.
055 Dinotrema sp.
Alysiinae
091
082 Eubazus sp.
068 Aliolus lepidus
044 Blacus ruficornis
012 Blacus ruficornis
072 Blacus ruficornis
008 Blacus ruficornis
098 Blacus sp.
079 Blacus sp.
032 Blacus sp.
067 Blacus sp.
043 Blacus sp.
Brachistinae
Cynipoidea
062 Leptopilina japonica
050 Alloxysta victrix
038 Alloxysta sp.
Figitidae
Platygastroidea
094
014
Scelionidae
029
053
005
089
041
021
088
030
054 Amblyaspis sp.
065 Amblyaspis sp.
042 Amblyaspis sp.
083 Inostemma sp.
076 Synopeas sp.
018
077
040
017
Platygastrinae
Platygastridae
Proctotrupoidea
080 Proctotrupes sp.
051 Exallonyx nixoni
007 Exallonyx sp.
087
087
026
063
004
Proctotrupidae
Diaprioidea
078
031
046
019
Diapriidae
Chalcidoidea
086
025
Mymaridae
085 Aprostocetus sp.
039 Omphale radialis
045 Omphale lugens
022 Omphale sp.
002 Diglyphus isaea
Eulophidae
015 Stenomalina sp.
010 Cratomus megacephalus
Pteromalidae
028 Metaphycus sp.
034
001
003
049
013 Blasthotrix sp.
Encyrtidae
Tree scale: 0.1

Figure S156: Phylogenetic trees showing genetic relationships between CO1 Sequences (black numbers represent ID numbers) of parasitoid wasps. The tree was constructed sing IQ-TREE version 1.6.12, based on the maximum optimality criterion. Numbers on branches represent bootstrap support. The inferred tree was rooted by selecting two *Ceraphronidae* specimens as phylogenetic outgroup.

References

1. Woelke, J. B., Pham, K. & Humala, A. E. New species of Stenomacrus (Hymenoptera: Ichneumonidae: Orthocentrinae) reared from Bradysia impatiens (Diptera: Sciaridae) in The Netherlands. *Journal of Natural History* **54**, 1603–1616 (2020).
2. Schmidt, K. & Zmudzinski, F. 3. Beitrag zur Kenntnis der badischen Schlupfwespenfauna (Hymenoptera, Ichneumonidae). *Carolinea* **61**, 119–132 (2003).
3. Broad, G. R. Checklist of British and Irish Hymenoptera - Ichneumonidae. *Biodiversity Data Journal* **4**, (2016).
4. Klopfstein, S., Riedel, M. & Schwarz, M. Checklist of ichneumonid parasitoid wasps in Switzerland (Hymenoptera, Ichneumonidae): 470 species new for the country and an appraisal of the alpine diversity. *Alpine Entomology* **3**, 51–81 (2019).
5. Zwakhals, K., Blommers, L. & Kaaistoep, D. Tryphoninae (Hymenoptera : Ichneumonidae) reared from sawflies (Hymenoptera : Symphyta) in the Netherlands. *Entomologische Berichten* **82**, 9–17 (2022).
6. Van Achterberg, C. The European species of the subgenus Aliolus Say of the genus Eubazus Nees and of the genus Dicyrtaspis van Achterberg (Hymenoptera: Braconidae: *Zoologische Mededelingen Leiden* **77**, 301–320 (2003).
7. Belokobylskij, S. A., Taeger, A., Van Achterberg, C., Haeselbarth, E. & Riedel, M. Checklist of the Braconidae of Germany (Hymenoptera). *Beiträge zur Entomologie = Contributions to Entomology* **53**, 341–435 (2003).
8. Holler, C., Borgemeister, C., Haardt, H. & Powell, W. The Relationship between Primary Parasitoids and Hyperparasitoids of Cereal Aphids: An Analysis of Field Data. *J Anim Ecol* **62**, 12 (1993).
9. Ferrer-Suay, M., Selfa, J. & Pujade-Villar, J. Palaearctic species of charipinae (Hymenoptera, figitidae): Two new species, synthesis and identifi cation key. *European Journal of Taxonomy* **2018**, 1–110 (2018).
10. Ferrer-Suay, M. *et al.* Nuclear and mitochondrial markers suggest new species boundaries in Alloxysta (Hymenoptera: Cynipoidea: Figitidae). *Arthropod Syst Phylogeny* **76**, 463–473 (2018).
11. Sivell, O. & Broad, G. R. The genome sequence of an ichneumonid wasp , Amblyteles armatorius (Forster , 1771) [version 1 ; peer review : 1 approved] Natural History Museum Genome Acquisition Lab , Darwin Tree of Life Barcoding collective , Wellcome Sanger Institute Tree of Lif. 1–10 (2023).
12. Aydoğdu, M. Parasitoid abundance of Archips rosana (Linnaeus, 1758) (Lepidoptera: Tortricidae) in organic cherry orchards. *North West J Zool* **10**, 42–47 (2014).
13. Ye, Z., Vollhardt, I. M. G., Tomanovic, Z. & Traugott, M. Evaluation of three molecular markers for identification of European primary parasitoids of cereal aphids and their hyperparasitoids. *PLoS One* **12**, 1–20 (2017).
14. Goulet, H. & Huber, J. T. *Hymenoptera of the World: An Identification Guide to Families*. *American Entomologist* (1993). doi:10.1093/ae/40.2.115.
15. Schmidt, K., Zmudzinski, F. & Riedel, M. Beiträge zur Kenntnis der badischen Schlupfwespenfauna (Hymenoptera, Ichneumonidae) 8. Metopiinae, Tersilochinae und neun weitere Unterfamilien. *Carolinea* **68**, 61–78 (2010).
16. Abram, P. K. *et al.* New records of leptopilina, ganaspis, and asobara species associated with Drosophila suzukii in North America, including detections of L. japonica and G. brasiliensis. *J Hymenopt Res* **78**, 1–17 (2020).
17. Khalaim, A. I. A review of the Palaearctic species of the genera Barycnemis Förat., Epistathmus Först. and Spinolochus Horstm. (Hymenoptera: Ichneumonidae, Tersilochinae). *Proceedings of the Russian Entomological Society* **75**, 46–63 (2004).

18. Haeselbarth, E. Die Blacus-Arten Europas und Zentral-Asiens. *Münchner Entomologische Gesellschaft, Veröffentlichungen der zoologischen Staatssamlung München* (1974).
19. Wisniowski, B. Catalogue of Hymenoptera (Arthropoda: Insecta) of the Ojców National Park. **26**, 95–146 (2016).
20. Vidal, S., Müller, J. & Schmidt, S. Critical checklist of the Chalcidoidea and Mymarommatoidea (Insecta, Hymenoptera) of Germany. *Biodiversity Data Journal* **10**, (2022).
21. Haas, M. *et al.* Tiny wasps, huge diversity – A review of German Pteromalidae with new generic and species records (Hymenoptera: Chalcidoidea). *Biodiversity Data Journal* **9**, (2021).
22. Mitroiu, M.-D. & Andriescu, I. A preliminary faunistic review of the pteromalids (Hymenoptera: Chalcidoidea, Pteromalidae) of the Romanian protected areas. *Analele Stiintifice ale Universitatii 'Al. I. Cuza' din Iasi Sectiunea and Biologie Animala* (2006).
23. Azidah, A. A., Fitton, M. G. & Quicke, D. L. J. Identification of the Diadegma species (Hymenoptera: Ichneumonidae, Campopleginae) attacking the diamondback moth, Plutella xylostella (Lepidoptera: Plutellidae). *Bull Entomol Res* **90**, 375–389 (2000).
24. Juric, I., Salzburger, W. & Balmer, O. Spread and global population structure of the diamondback moth Plutella xylostella (Lepidoptera: Plutellidae) and its larval parasitoids Diadegma semiclausum and Diadegma fenestrale (Hymenoptera: Ichneumonidae) based on mtDNA. *Bull Entomol Res* **107**, 155–164 (2017).
25. Schmidt, K., Zmudzinski, F. & Riedel, M. Beiträge zur Kenntnis der badischen Schlupf- wespenfauna (Hymenoptera, Ichneumonidae) 9. Unterfamilie Campopleginae. 95–122 (2011).
26. Xu, X. *et al.* Molecular identification of hymenopteran parasitoids and their endosymbionts from agromyzids. *Bulletin of Entomological Research* (2023) doi:10.1017/S0007485323000160.
27. Broad, G. R. Checklist of British and Irish Hymenoptera - Proctotrupoidea. *Biodiversity Data Journal* **4**, (2016).
28. Izadizadeh, M. *et al.* Review of the Family Proctotrupidae (Hymenoptera Proctotrupoidea) in Iran. *Redia* **105**, 37–58 (2022).
29. Martin, J., Vogel, J., Peters, R. S. & Herz, A. First record of Leptopilina japonica Novković & Kimura, 2011 (Hymenoptera: Figitidae) in Germany, a parasitoid of the Spotted Wing Drosophila Drosophila suzukii (Matsumura, 1931) (Diptera: Drosophilidae). *Journal of Applied Entomology* 1–7 (2023) doi:10.1111/jen.13182.
30. Puppato, S., Grassi, A., Pedrazzoli, F., De Cristofaro, A. & Ioriatti, C. First report of leptopilina japonica in europe. *Insects* **11**, 1–13 (2020).
31. Stigenberg, J. & Ronquist, F. *Revision of the Western Palearctic Meteorini (Hymenoptera, Braconidae), with a Molecular Characterization of Hidden Fennoscandian Species Diversity*. *Zootaxa* vol. 95 (2011).
32. Hansson, C. & Shevtsova, E. Revision of the European species of Omphale Haliday (Hymenoptera, chalcidoidea, eulophidae). *ZooKeys* **232**, 1–157 (2012).
33. Gijswijt, M. J. Notes on biology and distribution of the Genus Omphale Haliday 1833, with description of two new species (Eulophidae). *Bulletin Zoologisch Museum, Universiteit van Amsterdam* **5**, 77–83 (1976).
34. Englert, C. & Herz, A. Native predators and parasitoids for biological regulation of Drosophila suzukii in Germany. *Proceedings of the Ecofruit. 17th Intern. Conference on Organic Fruit-Growing: Proceedings* 284–285 (2017).

35. Peters, R. S. New records and notes on the life history of Pachycrepoideus vindemmiae (Rondani , 1875) (Hymenoptera : Chalcidoidea : Pteromalidae). (2009).
36. Knoll, V., Ellenbroek, T., Romeis, J. & Collatz, J. Seasonal and regional presence of hymenopteran parasitoids of Drosophila in Switzerland and their ability to parasitize the invasive Drosophila suzukii. *Sci Rep* **7**, 1–11 (2017).
37. Komonen, A., Siitonen, J. & Mutanen, M. Insects inhabiting two old-growth forest polypore species. *Entomologica Fennica* **12**, 3–14 (2001).
38. Mohammadi-Khoramabadi, A., Talebi, A. A. & ... Study of three genera of the Orthocentrus genus-group (Hymenoptera: Ichneumonidae, Orthocentrinae) in northern Iran. *Journal of Entomological ...* **37**, 441–460 (2018).

Identification literature:

Azidah, A. A., Fitton, M. G., & Quicke, D. L. J. (2000). Identification of the Diadegma species (Hymenoptera: Ichneumonidae, Campopleginae) attacking the diamondback moth, Plutella xylostella (Lepidoptera: Plutellidae). Bulletin of Entomological Research, 90(5), 375–389. https://doi.org/10.1017/S0007485300000511

Boucek, Z., & Rasplus, J. Y. (1988). Illustrated Key to West-Palearctic Genera of Pteromalidae. January 1991.

Buhl, P. N. (2015). Further new or little known Neotropical species of Platygastrinae (Hymenoptera: Platygastridae). International Journal of Environmental Studies, 72(2), 316–330. https://doi.org/10.1080/00207233.2014.994275

Buhl, P. N. (2016). New European species of Platygastrinae , with an updated list of Latvian species of Platygastrinae and Sceliotrachelinae (Hymenoptera: Platygastridae). Latvijas Entomologs, 53, 3–13.

Buhl, P. N. (2019). New species and notes on variation in Platygastrinae from Germany (Hymenoptera, Platygastridae). Entomofauna, 40(1), 337–346.

Ferrer-Suay, M., Selfa, J., Notton, D. G., & Pujade-Villar, J. (2013). Revision of the types of species of Alloxysta described by Cameron and Fergusson (Hymenoptera: Figitidae: Charipinae) and deposited in the Natural History Museum (London), including a key to the fauna of Great Britain. European Journal of Taxonomy, 53, 1–27. https://doi.org/10.5852/ejt.2013.53

Ferrer-Suay, M., Selfa, J., & Pujade-Villar, J. (2015). New contribution to the knowledge of the genus Alloxysta (Insecta : Hymenoptera : Cynipoidea : Figitidae): revision of some type material. 117, 23–36.

Ferrer-Suay, M., Selfa, J., & Pujade-Villar, J. (2018). Palaearctic species of charipinae (Hymenoptera, figitidae): Two new species, synthesis and identifi cation key. European Journal of Taxonomy, 2018(427), 1–110. https://doi.org/10.5852/ejt.2018.427

Ferrer-Suay, M., Selfa, J., & Pujade-Villar, J. (2019). Keys to world charipinae (Hymenoptera, cynipoidea, figitidae). ZooKeys, 2019(822), 79–139. https://doi.org/10.3897/zookeys.822.30151

Ferrer-Suay, M., Staverløkk, A., Selfa, J., Pujade-Villar, J., Naik, S., & Ekrem, T. (2018). Nuclear and mitochondrial markers suggest new species boundaries in Alloxysta (Hymenoptera: Cynipoidea: Figitidae). Arthropod Systematics and Phylogeny, 76(3), 463–473. https://doi.org/10.3897/asp.76.e31963

Fouts, R. M. (1924). Revision of the North American wasps of the subfamily Platygasterinae. Proceedings of the United States National Museum, 63(2484), 1–145. https://doi.org/10.5479/si.00963801.63-2484.1

Gijswijt, M. J. (1976). Notes on biology and distribution of the Genus Omphale Haliday 1833, with description of two new species (Eulophidae). Bulletin Zoologisch Museum, Universiteit van Amsterdam, 5(10), 77–83.

Goulet, H., & Huber, J. T. (1993). Hymenoptera of the World: An Identification Guide to Families. In Agriculture Canada, Centre for Land and Biological Resources Research Ottawa, Ontario. https://doi.org/10.1093/ae/40.2.115

Guerrieri, E., & Noyes, J. (2005). Revision of the European species of Copidosoma Ratzeburg (Hymenoptera: Encyrtidae), parasitoids of caterpillars (Lepidoptera). Systematic Entomology, 30(1), 97–174. https://doi.org/10.1111/j.1365-3113.2005.00271.x

Haas, M., Baur, H., Schweizer, T., Monje, J. C., Moser, M., Bigalk, S., & Krogmann, L. (2021). Tiny wasps, huge diversity – A review of German Pteromalidae with new generic and species

records (Hymenoptera: Chalcidoidea). Biodiversity Data Journal, 9. https://doi.org/10.3897/BDJ.9.E77092
Hansson, C., & Shevtsova, E. (2012). Revision of the European species of Omphale Haliday (Hymenoptera, chalcidoidea, eulophidae). ZooKeys, 232, 1–157. https://doi.org/10.3897/zookeys.232.3625
Izadizadeh, M., Talebi, A. A., Kolyada, V., Farahani, S., Kazerani, F., & Ameri, A. (2022). Review of the Family Proctotrupidae (Hymenoptera Proctotrupoidea) in Iran. Redia, 105(June), 37–58. https://doi.org/10.19263/REDIA-105.22.06
Japoshvili, G. O., & Noyes, J. S. (2006). New data on the European fauna of encyrtid wasps (Hymenoptera, Chalcidoidea, Encyrtidae). Entomological Review, 86(3), 298–304. https://doi.org/10.1134/s0013873806030067
Japoshvili, G., & Soethof, R. (2011). New records of Encyrtidae (Hymenoptera: Chalcidoidea) from the Netherlands, with a description of new species. Zootaxa, 40(May), 27–40.
Khalaim, A. I. (2004). A review of the Palaearctic species of the genera Barycnemis Först., Epistathmus Först. and Spinolochus Horstm. (Hymenoptera: Ichneumonidae, Tersilochinae). Proceedings of the Russian Entomological Society, 75(1), 46–63.
Masner, L. (1980). Key To Genera of Scelionidae of the Holarctic Region, With Descriptions of New Genera and Species (Hymenoptera: Proctotrupoidea). In Memoirs of the Entomological Society of Canada (Vol. 112, Issue S113, pp. 1–54). https://doi.org/10.4039/entm112113fv
Masner, L., & García R., J. L. (2002). The genera of Diapriinae (Hymenoptera: Diapriidae) in the New World. Bulletin of the American Museum of Natural History, 268, 2–125. https://doi.org/10.1206/0003-0090(2002)268<0001:tgodhd>2.0.co;2
Mohammadi-Khoramabadi, A., Talebi, A. A. (2018). Study of three genera of the Orthocentrus genus-group (Hymenoptera: Ichneumonidae, Orthocentrinae) in northern Iran. Journal of Entomological, 37(February), 441–460. https://doi.org/10.22117/jesi.2018.116455.1163
Pricop, E. (2013). Identification key to European genera of the Mymaridae (Hymenoptera: Chalcidoidea), with additional notes. ELBA Bioflux, 5(1), undefined-69-81.
Riedel, M., Schmidt, K., & Zmudzinski, F. (2013). Beiträge zur Kenntnis der badischen Schlupfwespenfauna (Hymenoptera, Ichneumonidae) 11. Nachträge und Korrekturen Matthias. 25–53.
Schaefer, M. (2009). Brohmer - Fauna von Deutschland. Quelle & Meyers. ISBN:9783494014722
Schmidt, K., & Zmudzinski, F. (2003). 3. Beitrag zur Kenntnis der badischen Schlupfwespenfauna (Hymenoptera, Ichneumonidae). Carolinea, 61, 119–132.
Schmidt, K., Zmudzinski, F., & Riedel, M. (2010). Beiträge zur Kenntnis der badischen Schlupfwespenfauna (Hymenoptera, Ichneumonidae) 8. Metopiinae, Tersilochinae und neun weitere Unterfamilien. Carolinea, 68, 61–78. www.zobodat.at
Schmidt, K., Zmudzinski, F., & Riedel, M. (2011). Beiträge zur Kenntnis der badischen Schlupfwespenfauna (Hymenoptera, Ichneumonidae) 9. Unterfamilie Campopleginae. 1924, 95–122.
Shimbori, E. M., Costa, V. A., & Zucchi, R. A. (2020). Annotated checklist and illustrated key to parasitoids (Hymenoptera: Diapriidae, Eulophidae and Pteromalidae) of fruit flies (Diptera, Tephritidae) in Brazil. Zootaxa, 4858(1), 53–70. https://doi.org/10.11646/zootaxa.4858.1.3
Stigenberg, J., & Peris-Felipo, F. J. (2019). Contribution to the knowledge of Swedish Dacnusini (Hymenoptera, Braconidae: Alysiinae): checklist and seven new species records. Journal of Insect Biodiversity and Systematics, 5(3), 221–230. https://doi.org/10.52547/jibs.5.3.221
Stigenberg, J., & Ronquist, F. (2011). Revision of the western Palearctic meteorini (Hymenoptera, Braconidae), with a molecular characterization of hidden Fennoscandian species diversity. In Zootaxa (Vol. 95, Issue 3084). https://doi.org/10.11646/zootaxa.3084.1.1
Stigenberg, J., & Shaw, M. R. (2013). Western palaearctic meteorinae (Hymenoptera: Braconidae) in the national museums of Scotland, with rearing, phenological and distributional data,

including six species new to Britain, and a discussion of a potential route to speciation. Entomologist's Gazette, 64(4), 251–268.
Van Achterberg, C. (2003). The European species of the subgenus Aliolus Say of the genus Eubazus Nees and of the genus Dicyrtaspis van Achterberg (Hymenoptera: Braconidae: Zoologische Mededelingen Leiden, 77(17), 301–320. http://www.repository.naturalis.nl/document/44277
Verheyde, F., Hoekstra2, P., Libert, P.-N., Meijer, H., De Ketelaere, A., Vandaudenard, T., Belgers, D., & Brosens, E. (2021). Belgian Journal of Entomology Two hundred and five ichneumonid wasps reported for the first time in Belgium and the Netherlands (Hymenoptera: Ichneumonidae). Belgian Journal of Entomology, 122, 1–142. www.srbe-kbve.be
Yu, F., Chen, F. Q., Yen, S. H., Tu, L. H., Zhu, C. D., Guerrieri, E., & Zhang, Y. Z. (2014). Preliminary phylogeny of the genus Copidosoma (Hymenoptera, Encyrtidae), polyembryonic parasitoids of Lepidoptera. Systematic Entomology, 39(2), 325–334. https://doi.org/10.1111/syen.12057
